# Supplementary material for: Risk of lead exposure, subcortical brain structure, and cognition in a large cohort of 9- to 10-year-old children
Source: PLoS One. 2021 Oct 14;16(10):e0258469. doi: 10.1371/journal.pone.0258469 (PMC8516269; doi:10.1371/journal.pone.0258469)
Supplement: S1 Appendix — (DOCX) [file pone.0258469.s001.docx]

**Supporting Information**


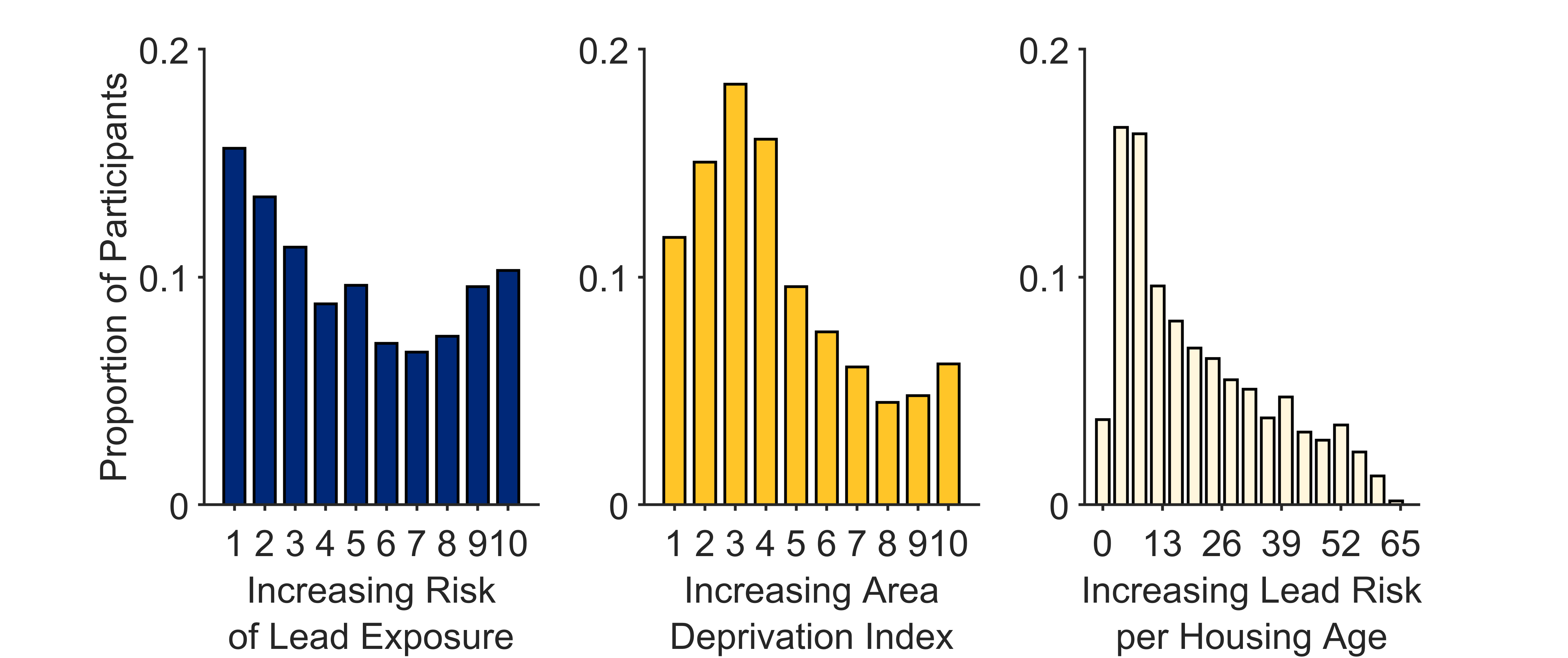


**C**

**B**

**A**

**Supplementary Figure 1**. Proportion of children in the analyzed sample of the ABCD cohort by (**A**) risk of lead exposure, (**B**) area deprivation index, and (**C**) the risk of lead exposure per the subcomponent of housing age (i.e., estimated proportion of houses in each census tract with lead-based paint hazards, based on age of the houses). Each of these indices were geocoded to participants’ primary residential addresses at the census-tract level.

**Supplementary Table 1. NDA Data Release 2.0.1 Variables**

| Data Tables and Variables (NDA Release 2.0.1) | Variable Labels in Current Report |
| --- | --- |
| **Demographics (pdem02)** |  |
| demo_comb_income_v2 | Family Income |
| demo_prnt_ed_v2 | Caregiver (Parent) 1 Education Level |
| demo_prtnr_ed_v2 | Caregiver (Parent) 2 Education Level |
| demo_race_a_p___10 | Child Race (White) |
| demo_race_a_p___11 | Child Race (Black) |
| demo_race_a_p___12 | Child Race (American Indian) |
| demo_race_a_p___13 | Child Race (Alaska Native) |
| demo_race_a_p___14 | Child Race (Native Hawaiian) |
| demo_race_a_p___15 | Child Race (Guamanian) |
| demo_race_a_p___16 | Child Race (Samoan) |
| demo_race_a_p___17 | Child Race (Other Pacific Islander) |
| demo_race_a_p___18 | Child Race (Asian Indian) |
| demo_race_a_p___19 | Child Race (Chinese) |
| demo_race_a_p___20 | Child Race (Filipino) |
| demo_race_a_p___21 | Child Race (Japanese) |
| demo_race_a_p___22 | Child Race (Korean) |
| demo_race_a_p___23 | Child Race (Vietnamese) |
| demo_race_a_p___24 | Child Race (Other Asian) |
| demo_race_a_p___25 | Child Race (Other Race) |
| demo_ethn_v2 | Child Ethnicity (Hispanic) |
|  |  |
| **NIH Toolbox (abcd_tbss01)** |  |
| nihtbx_picvocab_uncorrected | Picture Vocabulary Test Score |
| nihtbx_flanker_uncorrected | Flanker Test Score |
| nihtbx_list_uncorrected | List Sorting Test Score |
| nihtbx_cardsort_uncorrected | Dimensional Change Card Sort Test Score |
| nihtbx_pattern_uncorrected | Pattern Comparison Test Score |
| nihtbx_picture_uncorrected | Picture Sequence Memory Test Score |
| nihtbx_reading_uncorrected | Oral Reading Test Score |
|  |  |
| **Structural MRI (abcd_smrip201)** |  |
| smri_vol_scs_tplh | Left Thalamus Proper (Volume) |
| smri_vol_scs_tprh | Right Thalamus Proper (Volume) |
| smri_vol_scs_caudatelh | Left Caudate (Volume) |
| smri_vol_scs_caudaterh | Right Caudate (Volume) |
| smri_vol_scs_putamenlh | Left Putamen (Volume) |
| smri_vol_scs_putamenrh | Right Putamen (Volume) |
| smri_vol_scs_pallidumlh | Left Pallidum (Volume) |
| smri_vol_scs_pallidumrh | Right Pallidum (Volume) |
| smri_vol_scs_hpuslh | Left Hippocampus (Volume) |
| smri_vol_scs_hpusrh | Right Hippocampus (Volume) |
| smri_vol_scs_amygdalalh | Left Amygdala (Volume) |
| smri_vol_scs_amygdalarh | Right Amygdala (Volume) |
| smri_vol_scs_aal | Left Accumbens Area (Volume) |
| smri_vol_scs_aar | Right Accumbens Area (Volume) |
| smri_vol_scs_vedclh | Left Ventral Diencephalon (Volume) |
| smri_vol_scs_vedcrh | Right Ventral Diencephalon (Volume) |
| smri_vol_scs_crbcortexlh | Left Cerebellum Cortex (Volume) |
| smri_vol_scs_crbcortexrh | Right Cerebellum Cortex (Volume) |
| smri_vol_scs_crbwmatterlh | Left Cerebellum White Matter (Volume) |
| smri_vol_scs_crbwmatterrh | Right Cerebellum White Matter (Volume) |
| smri_vol_scs_ltventriclelh | Left Lateral Ventricle (Volume) |
| smri_vol_scs_ltventriclerh | Right Lateral Ventricle (Volume) |
| smri_vol_scs_inflatventlh | Left Inferior Lateral Ventricle (Volume) |
| smri_vol_scs_inflatventrh | Right Interior Lateral Ventricle (Volume) |
| smri_vol_scs_ccat | Anterior Corpus Callosum (Volume) |
| smri_vol_scs_ccmidat | Mid-Anterior Corpus Callosum (Volume) |
| smri_vol_scs_ccct | Central Corpus Callosum (Volume) |
| smri_vol_scs_ccmidps | Mid-Posterior Corpus Callosum (Volume) |
| smri_vol_scs_ccps | Posterior Corpus Callosum (Volume) |
| smri_vol_scs_3rdventricle | 3^rd^ Ventricle (Volume) |
| smri_vol_scs_4thventricle | 4^th^ Ventricle (Volume) |
| smri_vol_scs_bstem | Brain Stem (Volume) |
| smri_vol_scs_subcorticalgv | Subcortical Gray Matter Volume (Volume) |
| smri_vol_scs_intracranialv | Intracranial Volume |
|  |  |
| **MRI Scanner Information (abcd_mri01)** |  |
| mri_info_deviceserialnumber | MRI Device Serial Number |
|  |  |
| **FreeSurfer Quality Control (freesqc01)** |  |
| fsqc_qc | FreeSurfer Reconstruction Quality Control Score |
|  |  |
| **Neuroradiology Reports (abcd_mrfindings01)** |  |
| mrif_score | MRI Report Score |
|  |  |
| **MRI T_1_ Quality Control (mriqcrp102)** |  |
| iqc_t1_1_qc_score | MRI Quality Control T_1_ #1 |
| iqc_t1_2_qc_score | MRI Quality Control T_1_ #2 |
| iqc_t1_3_qc_score | MRI Quality Control T_1_ #3 |
|  |  |
| **Residential History (abcd_rhds01)** |  |
| reshist_addr1_valid | Validity of Primary Residential Address |
| reshist_addr1_leadrisk | Lead Risk (Composite Score) |
| reshist_addr1_leadrisk_housing | Lead Risk (Age of Housing Score) |
| reshist_addr1_adi_wsum | Area Deprivation Index (ADI) |
|  |  |
| **Longitudinal Tracking (abcd_lt01)** |  |
| site_id_l | Site ID Number |
|  |  |
| **American Community Survey Post Stratification Weights (acspsw03)** | |
| interview_age | Age |
| sex | Sex |
| rel_family_id | Family ID Number |

**Supplementary Table 2. Linear mixed-effects model output for the analysis of lead risk and volume of the thalamus proper, collapsed across hemispheres.**

|  | *t*(8508) | *p* | *b* | 95% CI |
| --- | --- | --- | --- | --- |
| Intercept | 229.13 | < .001 | 7530.03 | [7465.61, 7594.45] |
| Maximum Parental Education | 0.18 | .854 | 1.03 | [-9.89, 11.94] |
| Family Income (High) | 0.31 | .759 | 2.64 | [-14.21, 19.48] |
| Family Income (Low) | -1.02 | .307 | -9.61 | [-28.06, 8.84] |
| Sex | -3.97 | < .001 | -21.02 | [-31.38, -10.65] |
| Child Race (American Indian/Alaska Native) | -0.36 | .722 | -21.99 | [-143.04, 99.06] |
| Child Race (Asian) | -0.31 | .755 | -12.10 | [-88.12, 63.92] |
| Child Race (Black) | -0.51 | .609 | -15.88 | [-76.68, 44.93] |
| Child Race (Native Hawaiian / Pacific Islander) | 0.48 | .633 | 60.51 | [-188.11, 309.13] |
| Child Race (Other) | 0.42 | .677 | 12.49 | [-46.27, 71.24] |
| Child Ethnicity | -0.98 | .329 | -7.37 | [-22.16, 7.43] |
| Age | 6.31 | < .001 | 3.84 | [2.65, 5.04] |
| Lead Risk | -0.41 | .678 | -0.78 | [-4.48, 2.91] |
| Intracranial Volume | 93.80 | < .001 | 0.004 | [0.004, 0.004] |
| Family Income (High) × Lead Risk | 0.33 | .744 | 0.76 | [-3.79, 5.31] |
| Family Income (Low) × Lead Risk | -0.43 | .666 | -1.08 | [-5.99, 3.83] |

**Note**: The linear mixed-effects model incorporates testing the statistical significance of coefficients against a *t*-distribution. Family Income was a categorical, effects-coded factor, in which the level “Mid” served as the reference level. Family Income was operationally defined as the self-reported combined family income and partitioned into three levels: Low Income: ≤ $50K; Mid Income: $50K-$100K; High Income: ≥ $100K. Sex was also a categorical factor, effect coded with Male/Female as ‑1/+1. Child Ethnicity was also a categorical factor, effect coded with Hispanic/Non-Hispanic as ‑1/+1. Child Race was also a categorical factor, in which “White” served as the reference level. Age, Maximum Parental Education (i.e., highest education level between parents/caregivers), Lead Risk, and Intracranial Volume were centered continuous factors. The random effects structure included a random intercept for magnetic resonance imaging (MRI) scanner serial number and family identification number. Random effects were restricted to be uncorrelated. Analysis included 8,524 data points. The model accounted for 85.9% of the variance in the data (*R*^2^ = .859, adjusted *R*^2^ = .859).

**Supplementary Table 3. Linear mixed-effects model output for the analysis of lead risk and volume of the caudate, collapsed across hemispheres.**

|  | *t*(8508) | *p* | *b* | 95% CI |
| --- | --- | --- | --- | --- |
| Intercept | 94.78 | < .001 | 4028.18 | [3944.87, 4111.49] |
| Maximum Parental Education | 2.17 | .030 | 11.90 | [1.16, 22.64] |
| Family Income (High) | -0.23 | .820 | -1.93 | [-18.53, 14.67] |
| Family Income (Low) | -0.87 | .385 | -8.05 | [-26.23, 10.12] |
| Sex | 7.61 | < .001 | 39.36 | [29.22, 49.50] |
| Child Race (American Indian/Alaska Native) | -2.44 | .015 | -148.12 | [-266.95, -29.30] |
| Child Race (Asian) | -0.28 | .783 | -10.54 | [-85.42, 64.34] |
| Child Race (Black) | 1.43 | .154 | 43.68 | [-16.36, 103.72] |
| Child Race (Native Hawaiian / Pacific Islander) | 0.04 | .968 | 5.08 | [-240.70, 250.86] |
| Child Race (Other) | 1.54 | .124 | 45.46 | [-12.49, 103.42] |
| Child Ethnicity | 1.27 | .205 | 9.51 | [-5.20, 24.22] |
| Age | -3.51 | < .001 | -2.08 | [-3.24, -0.92] |
| Lead Risk | -1.60 | .110 | -2.99 | [-6.66, 0.68] |
| Intracranial Volume | 58.53 | < .001 | 0.002 | [0.002, 0.002] |
| Family Income (High) × Lead Risk | 0.96 | .338 | 2.19 | [-2.29, 6.67] |
| Family Income (Low) × Lead Risk | -0.30 | .765 | -0.74 | [-5.58, 4.10] |

**Note**: The linear mixed-effects model incorporates testing the statistical significance of coefficients against a *t*-distribution. Family Income was a categorical, effects-coded factor, in which the level “Mid” served as the reference level. Family Income was operationally defined as the self-reported combined family income and partitioned into three levels: Low Income: ≤ $50K; Mid Income: $50K-$100K; High Income: ≥ $100K. Sex was also a categorical factor, effect coded with Male/Female as ‑1/+1. Child Ethnicity was also a categorical factor, effect coded with Hispanic/Non-Hispanic as ‑1/+1. Child Race was also a categorical factor, in which “White” served as the reference level. Age, Maximum Parental Education (i.e., highest education level between parents/caregivers), Lead Risk, and Intracranial Volume were centered continuous factors. The random effects structure included a random intercept for magnetic resonance imaging (MRI) scanner serial number and family identification number. Random effects were restricted to be uncorrelated. Analysis included 8,524 data points. The model accounted for 76.4% of the variance in the data (*R*^2^ = .764, adjusted *R*^2^ = .763).

**Supplementary Table 4. Linear mixed-effects model output for the analysis of lead risk and volume of the putamen, collapsed across hemispheres.**

|  | *t*(8508) | *p* | *b* | 95% CI |
| --- | --- | --- | --- | --- |
| Intercept | 148.13 | < .001 | 5892.44 | [5814.46, 5970.41] |
| Maximum Parental Education | 0.22 | .829 | 1.45 | [-11.73, 14.62] |
| Family Income (High) | 1.04 | .300 | 10.74 | [-9.59, 31.08] |
| Family Income (Low) | -2.32 | .021 | -26.33 | [-48.61, -4.05] |
| Sex | -9.60 | < .001 | -61.00 | [-73.46, -48.54] |
| Child Race (American Indian/Alaska Native) | -1.59 | .112 | -118.41 | [-264.39, 27.56] |
| Child Race (Asian) | 3.23 | .001 | 151.19 | [59.37, 243.01] |
| Child Race (Black) | -1.86 | .063 | -69.82 | [-143.37, 3.72] |
| Child Race (Native Hawaiian / Pacific Islander) | 0.30 | .767 | 45.54 | [-255.61, 346.70] |
| Child Race (Other) | -0.04 | .970 | -1.38 | [-72.45, 69.69] |
| Child Ethnicity | -1.36 | .175 | -12.36 | [-30.21, 5.50] |
| Age | -3.86 | < .001 | -2.81 | [-4.24, -1.38] |
| Lead Risk | -1.78 | .074 | -4.06 | [-8.52, 0.40] |
| Intracranial Volume | 51.08 | < .001 | 0.002 | [0.002, 0.003] |
| Family Income (High) × Lead Risk | -0.11 | .910 | -0.32 | [-5.81, 5.18] |
| Family Income (Low) × Lead Risk | 1.19 | .232 | 3.61 | [-2.32, 9.54] |

**Note**: The linear mixed-effects model incorporates testing the statistical significance of coefficients against a *t*-distribution. Family Income was a categorical, effects-coded factor, in which the level “Mid” served as the reference level. Family Income was operationally defined as the self-reported combined family income and partitioned into three levels: Low Income: ≤ $50K; Mid Income: $50K-$100K; High Income: ≥ $100K. Sex was also a categorical factor, effect coded with Male/Female as ‑1/+1. Child Ethnicity was also a categorical factor, effect coded with Hispanic/Non-Hispanic as ‑1/+1. Child Race was also a categorical factor, in which “White” served as the reference level. Age, Maximum Parental Education (i.e., highest education level between parents/caregivers), Lead Risk, and Intracranial Volume were centered continuous factors. The random effects structure included a random intercept for magnetic resonance imaging (MRI) scanner serial number and family identification number. Random effects were restricted to be uncorrelated. Analysis included 8,524 data points. The model accounted for 75.3% of the variance in the data (*R*^2^ = .753, adjusted *R*^2^ = .753).

**Supplementary Table 5. Linear mixed-effects model output for the analysis of lead risk and volume of the pallidum, collapsed across hemispheres.**

|  | *t*(8508) | *p* | *b* | 95% CI |
| --- | --- | --- | --- | --- |
| Intercept | 111.20 | < .001 | 1785.46 | [1753.98, 1816.93] |
| Maximum Parental Education | 1.64 | .101 | 3.33 | [-0.64, 7.29] |
| Family Income (High) | 1.77 | .077 | 5.54 | [-0.60, 11.67] |
| Family Income (Low) | -1.25 | .212 | -4.28 | [-10.99, 2.44] |
| Sex | -10.66 | < .001 | -20.71 | [-24.52, -16.91] |
| Child Race (American Indian/Alaska Native) | 0.01 | .991 | 0.26 | [-43.82, 44.34] |
| Child Race (Asian) | 0.20 | .845 | 2.75 | [-24.86, 30.35] |
| Child Race (Black) | 0.73 | .464 | 8.22 | [-13.78, 30.21] |
| Child Race (Native Hawaiian / Pacific Islander) | 0.08 | .933 | 3.82 | [-85.63, 93.27] |
| Child Race (Other) | -0.33 | .738 | -3.62 | [-24.84, 17.60] |
| Child Ethnicity | -1.76 | .079 | -4.88 | [-10.33, 0.56] |
| Age | -7.48 | < .001 | -1.69 | [-2.13, -1.24] |
| Lead Risk | -0.91 | .365 | -0.63 | [-1.98, 0.73] |
| Intracranial Volume | 53.11 | < .001 | 0.001 | [0.001, 0.001] |
| Family Income (High) × Lead Risk | 1.26 | .206 | 1.07 | [-0.59, 2.72] |
| Family Income (Low) × Lead Risk | 0.21 | .837 | 0.19 | [-1.60, 1.98] |

**Note**: The linear mixed-effects model incorporates testing the statistical significance of coefficients against a *t*-distribution. Family Income was a categorical, effects-coded factor, in which the level “Mid” served as the reference level. Family Income was operationally defined as the self-reported combined family income and partitioned into three levels: Low Income: ≤ $50K; Mid Income: $50K-$100K; High Income: ≥ $100K. Sex was also a categorical factor, effect coded with Male/Female as ‑1/+1. Child Ethnicity was also a categorical factor, effect coded with Hispanic/Non-Hispanic as ‑1/+1. Child Race was also a categorical factor, in which “White” served as the reference level. Age, Maximum Parental Education (i.e., highest education level between parents/caregivers), Lead Risk, and Intracranial Volume were centered continuous factors. The random effects structure included a random intercept for magnetic resonance imaging (MRI) scanner serial number and family identification number. Random effects were restricted to be uncorrelated. Analysis included 8,524 data points. The model accounted for 64.7% of the variance in the data (*R*^2^ = .647, adjusted *R*^2^ = .646).

**Supplementary Table 6. Linear mixed-effects model output for the analysis of lead risk and volume of the hippocampus, collapsed across hemispheres.**

|  | *t*(8508) | *p* | *b* | 95% CI |
| --- | --- | --- | --- | --- |
| Intercept | 187.98 | < .001 | 4089.54 | [4046.90, 4132.19] |
| Maximum Parental Education | 2.64 | .008 | 10.43 | [2.69, 18.17] |
| Family Income (High) | 2.98 | .003 | 18.10 | [6.20, 30.01] |
| Family Income (Low) | -2.47 | .014 | -16.44 | [-29.50, -3.38] |
| Sex | -3.35 | .001 | -12.49 | [-19.80, -5.18] |
| Child Race (American Indian/Alaska Native) | 0.68 | .497 | 29.78 | [-56.12, 115.69] |
| Child Race (Asian) | -0.25 | .799 | -7.00 | [-60.90, 46.90] |
| Child Race (Black) | -4.79 | < .001 | -105.29 | [-148.42, -62.17] |
| Child Race (Native Hawaiian / Pacific Islander) | 1.44 | .150 | 129.95 | [-47.09, 307.00] |
| Child Race (Other) | -1.65 | .099 | -35.22 | [-77.03, 6.58] |
| Child Ethnicity | -1.07 | .287 | -5.56 | [-15.78, 4.67] |
| Age | 1.91 | .056 | 0.82 | [-0.02, 1.67] |
| Lead Risk | 1.18 | .240 | 1.54 | [-1.03, 4.11] |
| Intracranial Volume | 62.50 | < .001 | 0.002 | [0.002, 0.002] |
| Family Income (High) × Lead Risk | 0.09 | .925 | 0.15 | [-3.07, 3.38] |
| Family Income (Low) × Lead Risk | 0.76 | .448 | 1.35 | [-2.13, 4.82] |

**Note**: The linear mixed-effects model incorporates testing the statistical significance of coefficients against a *t*-distribution. Family Income was a categorical, effects-coded factor, in which the level “Mid” served as the reference level. Family Income was operationally defined as the self-reported combined family income and partitioned into three levels: Low Income: ≤ $50K; Mid Income: $50K-$100K; High Income: ≥ $100K. Sex was also a categorical factor, effect coded with Male/Female as ‑1/+1. Child Ethnicity was also a categorical factor, effect coded with Hispanic/Non-Hispanic as ‑1/+1. Child Race was also a categorical factor, in which “White” served as the reference level. Age, Maximum Parental Education (i.e., highest education level between parents/caregivers), Lead Risk, and Intracranial Volume were centered continuous factors. The random effects structure included a random intercept for magnetic resonance imaging (MRI) scanner serial number and family identification number. Random effects were restricted to be uncorrelated. Analysis included 8,524 data points. The model accounted for 78.4% of the variance in the data (*R*^2^ = .784, adjusted *R*^2^ = .784).

**Supplementary Table 7. Linear mixed-effects model output for the analysis of lead risk and volume of the amygdala, collapsed across hemispheres.**

|  | *t*(8508) | *p* | *b* | 95% CI |
| --- | --- | --- | --- | --- |
| Intercept | 100.91 | < .001 | 1590.15 | [1559.26, 1621.04] |
| Maximum Parental Education | 1.45 | .148 | 2.84 | [-1.01, 6.68] |
| Family Income (High) | 1.98 | .048 | 6.00 | [0.06, 11.94] |
| Family Income (Low) | -1.63 | .102 | -5.42 | [-11.92, 1.08] |
| Sex | -12.71 | < .001 | -23.93 | [-27.62, -20.24] |
| Child Race (American Indian/Alaska Native) | 1.91 | .056 | 41.59 | [-1.11, 84.28] |
| Child Race (Asian) | 1.09 | .277 | 14.83 | [-11.90, 41.55] |
| Child Race (Black) | -3.61 | < .001 | -39.17 | [-60.46, -17.89] |
| Child Race (Native Hawaiian / Pacific Islander) | 0.07 | .944 | 3.10 | [-83.41, 89.61] |
| Child Race (Other) | -1.56 | .119 | -16.34 | [-36.87, 4.19] |
| Child Ethnicity | -1.07 | .284 | -2.88 | [-8.16, 2.39] |
| Age | 1.31 | .189 | 0.29 | [-0.14, 0.72] |
| Lead Risk | -0.03 | .977 | -0.02 | [-1.33, 1.29] |
| Intracranial Volume | 54.99 | < .001 | 0.001 | [0.001, 0.001] |
| Family Income (High) × Lead Risk | -0.33 | .740 | -0.27 | [-1.87, 1.33] |
| Family Income (Low) × Lead Risk | 0.30 | .767 | 0.26 | [-1.47, 1.99] |

**Note**: The linear mixed-effects model incorporates testing the statistical significance of coefficients against a *t*-distribution. Family Income was a categorical, effects-coded factor, in which the level “Mid” served as the reference level. Family Income was operationally defined as the self-reported combined family income and partitioned into three levels: Low Income: ≤ $50K; Mid Income: $50K-$100K; High Income: ≥ $100K. Sex was also a categorical factor, effect coded with Male/Female as ‑1/+1. Child Ethnicity was also a categorical factor, effect coded with Hispanic/Non-Hispanic as ‑1/+1. Child Race was also a categorical factor, in which “White” served as the reference level. Age, Maximum Parental Education (i.e., highest education level between parents/caregivers), Lead Risk, and Intracranial Volume were centered continuous factors. The random effects structure included a random intercept for magnetic resonance imaging (MRI) scanner serial number and family identification number. Random effects were restricted to be uncorrelated. Analysis included 8,524 data points. The model accounted for 73.0% of the variance in the data (*R*^2^ = .730, adjusted *R*^2^ = .730).

**Supplementary Table 8. Linear mixed-effects model output for the analysis of lead risk and volume of the accumbens area, collapsed across hemispheres.**

|  | *t*(8508) | *p* | *b* | 95% CI |
| --- | --- | --- | --- | --- |
| Intercept | 56.59 | < .001 | 597.6 | [576.90, 618.31] |
| Maximum Parental Education | 0.09 | .925 | 0.09 | [-1.80, 1.98] |
| Family Income (High) | 0.82 | .412 | 1.22 | [-1.70, 4.14] |
| Family Income (Low) | -1.14 | .252 | -1.87 | [-5.06, 1.33] |
| Sex | -0.38 | .701 | -0.36 | [-2.17, 1.46] |
| Child Race (American Indian/Alaska Native) | 0.93 | .354 | 9.91 | [-11.06, 30.88] |
| Child Race (Asian) | -1.94 | .052 | -13.00 | [-26.13, 0.14] |
| Child Race (Black) | -0.06 | .954 | -0.31 | [-10.78, 10.16] |
| Child Race (Native Hawaiian / Pacific Islander) | 0.63 | .526 | 13.76 | [-28.80, 56.32] |
| Child Race (Other) | -1.41 | .160 | -7.24 | [-17.33, 2.86] |
| Child Ethnicity | -0.18 | .858 | -0.24 | [-2.83, 2.36] |
| Age | -6.35 | < .001 | -0.68 | [-0.89, -0.47] |
| Lead Risk | -1.30 | .193 | -0.43 | [-1.07, 0.22] |
| Intracranial Volume | 53.68 | < .001 | 0.0004 | [0.0004, 0.0004] |
| Family Income (High) × Lead Risk | 0.51 | .607 | 0.21 | [-0.58, 0.99] |
| Family Income (Low) × Lead Risk | 0.66 | .512 | 0.28 | [-0.57, 1.14] |

**Note**: The linear mixed-effects model incorporates testing the statistical significance of coefficients against a *t*-distribution. Family Income was a categorical, effects-coded factor, in which the level “Mid” served as the reference level. Family Income was operationally defined as the self-reported combined family income and partitioned into three levels: Low Income: ≤ $50K; Mid Income: $50K-$100K; High Income: ≥ $100K. Sex was also a categorical factor, effect coded with Male/Female as ‑1/+1. Child Ethnicity was also a categorical factor, effect coded with Hispanic/Non-Hispanic as ‑1/+1. Child Race was also a categorical factor, in which “White” served as the reference level. Age, Maximum Parental Education (i.e., highest education level between parents/caregivers), Lead Risk, and Intracranial Volume were centered continuous factors. The random effects structure included a random intercept for magnetic resonance imaging (MRI) scanner serial number and family identification number. Random effects were restricted to be uncorrelated. Analysis included 8,524 data points. The model accounted for 67.5% of the variance in the data (*R*^2^ = .675, adjusted *R*^2^ = .674).

**Supplementary Table 9. Linear mixed-effects model output for the analysis of lead risk and volume of the ventral diencephalon, collapsed across hemispheres.**

|  | *t*(8508) | *p* | *b* | 95% CI |
| --- | --- | --- | --- | --- |
| Intercept | 155.05 | < .001 | 3936.54 | [3886.77, 3986.31] |
| Maximum Parental Education | 1.67 | .095 | 5.46 | [-0.96, 11.87] |
| Family Income (High) | 1.48 | .140 | 7.47 | [-2.45, 17.38] |
| Family Income (Low) | -1.09 | .275 | -6.04 | [-16.90, 4.81] |
| Sex | -1.96 | .050 | -6.13 | [-12.27, 0.00] |
| Child Race (American Indian/Alaska Native) | 0.78 | .437 | 28.26 | [-42.95, 99.46] |
| Child Race (Asian) | -2.69 | .007 | -61.36 | [-106.01, -16.71] |
| Child Race (Black) | 3.59 | < .001 | 65.20 | [29.56, 100.84] |
| Child Race (Native Hawaiian / Pacific Islander) | -1.29 | .197 | -95.59 | [-240.77, 49.59] |
| Child Race (Other) | 1.78 | .075 | 31.26 | [-3.13, 65.64] |
| Child Ethnicity | -0.16 | .869 | -0.74 | [-9.54, 8.06] |
| Age | 10.12 | < .001 | 3.67 | [2.96, 4.38] |
| Lead Risk | 0.93 | .355 | 1.03 | [-1.16, 3.23] |
| Intracranial Volume | 86.20 | < .001 | 0.002 | [0.002, 0.002] |
| Family Income (High) × Lead Risk | 0.72 | .473 | 0.98 | [-1.70, 3.66] |
| Family Income (Low) × Lead Risk | 0.43 | .667 | 0.63 | [-2.26, 3.52] |

**Note**: The linear mixed-effects model incorporates testing the statistical significance of coefficients against a *t*-distribution. Family Income was a categorical, effects-coded factor, in which the level “Mid” served as the reference level. Family Income was operationally defined as the self-reported combined family income and partitioned into three levels: Low Income: ≤ $50K; Mid Income: $50K-$100K; High Income: ≥ $100K. Sex was also a categorical factor, effect coded with Male/Female as ‑1/+1. Child Ethnicity was also a categorical factor, effect coded with Hispanic/Non-Hispanic as ‑1/+1. Child Race was also a categorical factor, in which “White” served as the reference level. Age, Maximum Parental Education (i.e., highest education level between parents/caregivers), Lead Risk, and Intracranial Volume were centered continuous factors. The random effects structure included a random intercept for magnetic resonance imaging (MRI) scanner serial number and family identification number. Random effects were restricted to be uncorrelated. Analysis included 8,524 data points. The model accounted for 80.8% of the variance in the data (*R*^2^ = .808, adjusted *R*^2^ = .808).

**Supplementary Table 10. Linear mixed-effects model output for the analysis of lead risk and volume of the cerebellum cortex, collapsed across hemispheres.**

|  | *t*(8508) | *p* | *b* | 95% CI |
| --- | --- | --- | --- | --- |
| Intercept | 141.30 | < .001 | 52980.65 | [52245.67, 53715.64] |
| Maximum Parental Education | 3.93 | < .001 | 200.05 | [100.33, 299.76] |
| Family Income (High) | 1.94 | .052 | 152.91 | [-1.22, 307.04] |
| Family Income (Low) | -2.24 | .025 | -193.08 | [-361.83, -24.34] |
| Sex | -18.78 | < .001 | -894.03 | [-987.35, -800.70] |
| Child Race (American Indian/Alaska Native) | 0.14 | .891 | 77.19 | [-1023.72, 1178.10] |
| Child Race (Asian) | -1.98 | .048 | -703.19 | [-1398.99, -7.40] |
| Child Race (Black) | -0.42 | .675 | -119.54 | [-678.70, 439.62] |
| Child Race (Native Hawaiian / Pacific Islander) | -0.91 | .364 | -1062.57 | [-3357.61, 1232.46] |
| Child Race (Other) | 2.19 | .029 | 602.97 | [63.09, 1142.85] |
| Child Ethnicity | 5.92 | < .001 | 412.01 | [275.65, 548.37] |
| Age | -1.54 | .122 | -8.34 | [-18.92, 2.24] |
| Lead Risk | -0.37 | .712 | -6.41 | [-40.48, 27.66] |
| Intracranial Volume | 56.96 | < .001 | 0.02 | [0.02, 0.02] |
| Family Income (High) × Lead Risk | 2.07 | .038 | 44.03 | [2.40, 85.67] |
| Family Income (Low) × Lead Risk | -0.24 | .811 | -5.48 | [-50.38, 39.43] |

**Note**: The linear mixed-effects model incorporates testing the statistical significance of coefficients against a *t*-distribution. Family Income was a categorical, effects-coded factor, in which the level “Mid” served as the reference level. Family Income was operationally defined as the self-reported combined family income and partitioned into three levels: Low Income: ≤ $50K; Mid Income: $50K-$100K; High Income: ≥ $100K. Sex was also a categorical factor, effect coded with Male/Female as ‑1/+1. Child Ethnicity was also a categorical factor, effect coded with Hispanic/Non-Hispanic as ‑1/+1. Child Race was also a categorical factor, in which “White” served as the reference level. Age, Maximum Parental Education (i.e., highest education level between parents/caregivers), Lead Risk, and Intracranial Volume were centered continuous factors. The random effects structure included a random intercept for magnetic resonance imaging (MRI) scanner serial number and family identification number. Random effects were restricted to be uncorrelated. Analysis included 8,524 data points. The model accounted for 86.2% of the variance in the data (*R*^2^ = .862, adjusted *R*^2^ = .862).

**Supplementary Table 11. Linear mixed-effects model output for the analysis of lead risk and volume of cerebellar white matter, collapsed across hemispheres.**

|  | *t*(8508) | *p* | *b* | 95% CI |
| --- | --- | --- | --- | --- |
| Intercept | 133.50 | < .001 | 17101.83 | [16850.72, 17352.94] |
| Maximum Parental Education | 2.04 | .041 | 44.33 | [1.80, 86.85] |
| Family Income (High) | 1.48 | .140 | 49.43 | [-16.20, 115.07] |
| Family Income (Low) | -1.85 | .064 | -67.93 | [-139.83, 3.98] |
| Sex | -2.84 | .005 | -57.94 | [-97.99, -17.90] |
| Child Race (American Indian/Alaska Native) | 1.07 | .283 | 258.03 | [-212.66, 728.72] |
| Child Race (Asian) | -1.31 | .191 | -197.82 | [-494.34, 98.69] |
| Child Race (Black) | 2.64 | .008 | 320.71 | [82.90, 558.51] |
| Child Race (Native Hawaiian / Pacific Islander) | -0.91 | .363 | -452.40 | [-1427.66, 522.87] |
| Child Race (Other) | 0.69 | .490 | 80.99 | [-148.87, 310.84] |
| Child Ethnicity | 1.63 | .104 | 47.80 | [-9.77, 105.37] |
| Age | 7.20 | < .001 | 16.82 | [12.24, 21.40] |
| Lead Risk | -0.22 | .829 | -1.59 | [-15.99, 12.81] |
| Intracranial Volume | 50.19 | < .001 | 0.01 | [0.01, 0.01] |
| Family Income (High) × Lead Risk | 0.25 | .801 | 2.29 | [-15.45, 20.02] |
| Family Income (Low) × Lead Risk | 0.64 | .521 | 6.27 | [-12.87, 25.40] |

**Note**: The linear mixed-effects model incorporates testing the statistical significance of coefficients against a *t*-distribution. Family Income was a categorical, effects-coded factor, in which the level “Mid” served as the reference level. Family Income was operationally defined as the self-reported combined family income and partitioned into three levels: Low Income: ≤ $50K; Mid Income: $50K-$100K; High Income: ≥ $100K. Sex was also a categorical factor, effect coded with Male/Female as ‑1/+1. Child Ethnicity was also a categorical factor, effect coded with Hispanic/Non-Hispanic as ‑1/+1. Child Race was also a categorical factor, in which “White” served as the reference level. Age, Maximum Parental Education (i.e., highest education level between parents/caregivers), Lead Risk, and Intracranial Volume were centered continuous factors. The random effects structure included a random intercept for magnetic resonance imaging (MRI) scanner serial number and family identification number. Random effects were restricted to be uncorrelated. Analysis included 8,524 data points. The model accounted for 78.0% of the variance in the data (*R*^2^ = .780, adjusted *R*^2^ = .779).

**Supplementary Table 12. Linear mixed-effects model output for the analysis of lead risk and volume of the brain stem.**

|  | *t*(8508) | *p* | *b* | 95% CI |
| --- | --- | --- | --- | --- |
| Intercept | 138.70 | < .001 | 19164.9 | [18894.04, 19435.76] |
| Maximum Parental Education | 1.47 | .143 | 26.37 | [-8.91, 61.64] |
| Family Income (High) | 1.81 | .071 | 50.28 | [-4.25, 104.81] |
| Family Income (Low) | -1.16 | .245 | -35.42 | [-95.12, 24.27] |
| Sex | -8.88 | < .001 | -150.45 | [-183.64, -117.25] |
| Child Race (American Indian/Alaska Native) | 1.01 | .311 | 201.55 | [-188.43, 591.54] |
| Child Race (Asian) | -2.30 | .021 | -289.29 | [-535.34, -43.24] |
| Child Race (Black) | 3.86 | < .001 | 389.00 | [191.54, 586.47] |
| Child Race (Native Hawaiian / Pacific Islander) | -1.13 | .259 | -466.19 | [-1275.39, 343.01] |
| Child Race (Other) | 1.16 | .245 | 113.09 | [-77.53, 303.71] |
| Child Ethnicity | -0.67 | .504 | -16.47 | [-64.76, 31.82] |
| Age | 12.72 | < .001 | 24.56 | [20.77, 28.34] |
| Lead Risk | 0.21 | .835 | 1.28 | [-10.78, 13.33] |
| Intracranial Volume | 74.20 | < .001 | 0.01 | [0.01, 0.01] |
| Family Income (High) × Lead Risk | 0.18 | .858 | 1.35 | [-13.38, 16.08] |
| Family Income (Low) × Lead Risk | 2.11 | .035 | 17.11 | [1.22, 33.00] |

**Note**: The linear mixed-effects model incorporates testing the statistical significance of coefficients against a *t*-distribution. Family Income was a categorical, effects-coded factor, in which the level “Mid” served as the reference level. Family Income was operationally defined as the self-reported combined family income and partitioned into three levels: Low Income: ≤ $50K; Mid Income: $50K-$100K; High Income: ≥ $100K. Sex was also a categorical factor, effect coded with Male/Female as ‑1/+1. Child Ethnicity was also a categorical factor, effect coded with Hispanic/Non-Hispanic as ‑1/+1. Child Race was also a categorical factor, in which “White” served as the reference level. Age, Maximum Parental Education (i.e., highest education level between parents/caregivers), Lead Risk, and Intracranial Volume were centered continuous factors. The random effects structure included a random intercept for magnetic resonance imaging (MRI) scanner serial number and family identification number. Random effects were restricted to be uncorrelated. Analysis included 8,524 data points. The model accounted for 85.5% of the variance in the data (*R*^2^ = .855, adjusted *R*^2^ = .855).

**Supplementary Table 13. Linear mixed-effects model output for the analysis of lead risk and volume of the posterior corpus callosum.**

|  | *t*(8508) | *p* | *b* | 95% CI |
| --- | --- | --- | --- | --- |
| Intercept | 90.47 | < .001 | 796.13 | [778.88, 813.38] |
| Maximum Parental Education | 0.98 | .328 | 1.54 | [-1.54, 4.62] |
| Family Income (High) | 0.02 | .988 | 0.04 | [-4.71, 4.78] |
| Family Income (Low) | -0.53 | .595 | -1.41 | [-6.61, 3.79] |
| Sex | 1.99 | .046 | 2.96 | [0.05, 5.86] |
| Child Race (American Indian/Alaska Native) | 0.47 | .639 | 8.16 | [-25.99, 42.32] |
| Child Race (Asian) | 0.72 | .471 | 7.89 | [-13.58, 29.35] |
| Child Race (Black) | -0.05 | .962 | -0.42 | [-17.62, 16.77] |
| Child Race (Native Hawaiian / Pacific Islander) | -0.61 | .542 | -21.95 | [-92.51, 48.61] |
| Child Race (Other) | 0.95 | .340 | 8.10 | [-8.54, 24.75] |
| Child Ethnicity | -1.86 | .063 | -3.90 | [-8.01, 0.21] |
| Age | 7.39 | < .001 | 1.26 | [0.93, 1.59] |
| Lead Risk | -2.03 | .043 | -1.06 | [-2.10, -0.03] |
| Intracranial Volume | 28.68 | < .001 | 0.0003 | [0.0003, 0.0003] |
| Family Income (High) × Lead Risk | 0.51 | .607 | 0.34 | [-0.95, 1.62] |
| Family Income (Low) × Lead Risk | 0.74 | .460 | 0.52 | [-0.86, 1.91] |

**Note**: The linear mixed-effects model incorporates testing the statistical significance of coefficients against a *t*-distribution. Family Income was a categorical, effects-coded factor, in which the level “Mid” served as the reference level. Family Income was operationally defined as the self-reported combined family income and partitioned into three levels: Low Income: ≤ $50K; Mid Income: $50K-$100K; High Income: ≥ $100K. Sex was also a categorical factor, effect coded with Male/Female as ‑1/+1. Child Ethnicity was also a categorical factor, effect coded with Hispanic/Non-Hispanic as ‑1/+1. Child Race was also a categorical factor, in which “White” served as the reference level. Age, Maximum Parental Education (i.e., highest education level between parents/caregivers), Lead Risk, and Intracranial Volume were centered continuous factors. The random effects structure included a random intercept for magnetic resonance imaging (MRI) scanner serial number and family identification number. Random effects were restricted to be uncorrelated. Analysis included 8,524 data points. The model accounted for 60.2% of the variance in the data (*R*^2^ = .602, adjusted *R*^2^ = .602).

**Supplementary Table 14. Linear mixed-effects model output for the analysis of lead risk and volume of the mid-posterior corpus callosum.**

|  | *t*(8508) | *p* | *b* | 95% CI |
| --- | --- | --- | --- | --- |
| Intercept | 70.73 | < .001 | 375.83 | [365.42, 386.25] |
| Maximum Parental Education | 0.79 | .430 | 0.76 | [-1.13, 2.65] |
| Family Income (High) | -0.94 | .348 | -1.39 | [-4.30, 1.52] |
| Family Income (Low) | -0.24 | .812 | -0.39 | [-3.58, 2.80] |
| Sex | 1.88 | .061 | 1.72 | [-0.08, 3.53] |
| Child Race (American Indian/Alaska Native) | 1.15 | .251 | 12.31 | [-8.71, 33.33] |
| Child Race (Asian) | -0.56 | .576 | -3.75 | [-16.89, 9.40] |
| Child Race (Black) | -1.46 | .145 | -7.80 | [-18.28, 2.69] |
| Child Race (Native Hawaiian / Pacific Islander) | -0.81 | .419 | -17.65 | [-60.49, 25.19] |
| Child Race (Other) | 1.47 | .142 | 7.61 | [-2.54, 17.76] |
| Child Ethnicity | 0.65 | .514 | 0.84 | [-1.67, 3.35] |
| Age | 7.59 | < .001 | 0.81 | [0.60, 1.02] |
| Lead Risk | -3.21 | .001 | -1.03 | [-1.66, -0.40] |
| Intracranial Volume | 19.27 | < .001 | 0.0001 | [0.0001, 0.0001] |
| Family Income (High) × Lead Risk | 0.31 | .758 | 0.12 | [-0.66, 0.91] |
| Family Income (Low) × Lead Risk | 1.04 | .296 | 0.45 | [-0.40, 1.30] |

**Note**: The linear mixed-effects model incorporates testing the statistical significance of coefficients against a *t*-distribution. Family Income was a categorical, effects-coded factor, in which the level “Mid” served as the reference level. Family Income was operationally defined as the self-reported combined family income and partitioned into three levels: Low Income: ≤ $50K; Mid Income: $50K-$100K; High Income: ≥ $100K. Sex was also a categorical factor, effect coded with Male/Female as ‑1/+1. Child Ethnicity was also a categorical factor, effect coded with Hispanic/Non-Hispanic as ‑1/+1. Child Race was also a categorical factor, in which “White” served as the reference level. Age, Maximum Parental Education (i.e., highest education level between parents/caregivers), Lead Risk, and Intracranial Volume were centered continuous factors. The random effects structure included a random intercept for magnetic resonance imaging (MRI) scanner serial number and family identification number. Random effects were restricted to be uncorrelated. Analysis included 8,524 data points. The model accounted for 43.0% of the variance in the data (*R*^2^ = .430, adjusted *R*^2^ = .429).

**Supplementary Table 15. Linear mixed-effects model output for the analysis of lead risk and volume of the central corpus callosum.**

|  | *t*(8508) | *p* | *b* | 95% CI |
| --- | --- | --- | --- | --- |
| Intercept | 66.63 | < .001 | 405.60 | [393.67, 417.53] |
| Maximum Parental Education | -0.70 | .481 | -0.75 | [-2.85, 1.34] |
| Family Income (High) | -1.07 | .284 | -1.77 | [-5.00, 1.47] |
| Family Income (Low) | -0.07 | .946 | -0.12 | [-3.67, 3.42] |
| Sex | 3.37 | .001 | 3.46 | [1.45, 5.46] |
| Child Race (American Indian/Alaska Native) | 0.30 | .766 | 3.53 | [-19.78, 26.85] |
| Child Race (Asian) | 1.63 | .103 | 12.12 | [-2.46, 26.69] |
| Child Race (Black) | 0.73 | .462 | 4.35 | [-7.26, 15.96] |
| Child Race (Native Hawaiian / Pacific Islander) | -1.44 | .151 | -34.65 | [-81.95, 12.65] |
| Child Race (Other) | 1.77 | .077 | 10.14 | [-1.08, 21.36] |
| Child Ethnicity | 1.27 | .204 | 1.83 | [-0.99, 4.66] |
| Age | 4.99 | < .001 | 0.59 | [0.36, 0.83] |
| Lead Risk | -3.47 | .001 | -1.25 | [-1.96, -0.54] |
| Intracranial Volume | 17.60 | < .001 | 0.0001 | [0.0001, 0.0002] |
| Family Income (High) × Lead Risk | 0.99 | .322 | 0.44 | [-0.43, 1.32] |
| Family Income (Low) × Lead Risk | -0.28 | .778 | -0.14 | [-1.08, 0.81] |

**Note**: The linear mixed-effects model incorporates testing the statistical significance of coefficients against a *t*-distribution. Family Income was a categorical, effects-coded factor, in which the level “Mid” served as the reference level. Family Income was operationally defined as the self-reported combined family income and partitioned into three levels: Low Income: ≤ $50K; Mid Income: $50K-$100K; High Income: ≥ $100K. Sex was also a categorical factor, effect coded with Male/Female as ‑1/+1. Child Ethnicity was also a categorical factor, effect coded with Hispanic/Non-Hispanic as ‑1/+1. Child Race was also a categorical factor, in which “White” served as the reference level. Age, Maximum Parental Education (i.e., highest education level between parents/caregivers), Lead Risk, and Intracranial Volume were centered continuous factors. The random effects structure included a random intercept for magnetic resonance imaging (MRI) scanner serial number and family identification number. Random effects were restricted to be uncorrelated. Analysis included 8,524 data points. The model accounted for 36.9% of the variance in the data (*R*^2^ = .369, adjusted *R*^2^ = .368).

**Supplementary Table 16. Linear mixed-effects model output for the analysis of lead risk and volume of the mid-anterior corpus callosum.**

|  | *t*(8508) | *p* | *b* | 95% CI |
| --- | --- | --- | --- | --- |
| Intercept | 63.04 | < .001 | 434.01 | [420.51, 447.50] |
| Maximum Parental Education | -2.26 | .024 | -2.73 | [-5.11, -0.36] |
| Family Income (High) | -1.26 | .208 | -2.35 | [-6.01, 1.31] |
| Family Income (Low) | 0.63 | .526 | 1.30 | [-2.72, 5.31] |
| Sex | -0.12 | .902 | -0.14 | [-2.42, 2.13] |
| Child Race (American Indian/Alaska Native) | 0.56 | .578 | 7.50 | [-18.91, 33.91] |
| Child Race (Asian) | 2.54 | .011 | 21.40 | [4.90, 37.91] |
| Child Race (Black) | -1.29 | .196 | -8.67 | [-21.82, 4.47] |
| Child Race (Native Hawaiian / Pacific Islander) | -1.35 | .177 | -36.91 | [-90.45, 16.63] |
| Child Race (Other) | 1.67 | .094 | 10.85 | [-1.85, 23.56] |
| Child Ethnicity | 0.64 | .524 | 1.04 | [-2.16, 4.24] |
| Age | 0.05 | .962 | 0.01 | [-0.26, 0.27] |
| Lead Risk | -3.73 | < .001 | -1.52 | [-2.32, -0.72] |
| Intracranial Volume | 23.68 | < .001 | 0.0002 | [0.0002, 0.0002] |
| Family Income (High) × Lead Risk | 0.66 | .509 | 0.33 | [-0.66, 1.32] |
| Family Income (Low) × Lead Risk | 0.23 | .819 | 0.12 | [-0.94, 1.19] |

**Note**: The linear mixed-effects model incorporates testing the statistical significance of coefficients against a *t*-distribution. Family Income was a categorical, effects-coded factor, in which the level “Mid” served as the reference level. Family Income was operationally defined as the self-reported combined family income and partitioned into three levels: Low Income: ≤ $50K; Mid Income: $50K-$100K; High Income: ≥ $100K. Sex was also a categorical factor, effect coded with Male/Female as ‑1/+1. Child Ethnicity was also a categorical factor, effect coded with Hispanic/Non-Hispanic as ‑1/+1. Child Race was also a categorical factor, in which “White” served as the reference level. Age, Maximum Parental Education (i.e., highest education level between parents/caregivers), Lead Risk, and Intracranial Volume were centered continuous factors. The random effects structure included a random intercept for magnetic resonance imaging (MRI) scanner serial number and family identification number. Random effects were restricted to be uncorrelated. Analysis included 8,524 data points. The model accounted for 39.9% of the variance in the data (*R*^2^ = .399, adjusted *R*^2^ = .398).

**Supplementary Table 17. Linear mixed-effects model output for the analysis of lead risk and volume of the anterior corpus callosum.**

|  | *t*(8508) | *p* | *b* | 95% CI |
| --- | --- | --- | --- | --- |
| Intercept | 79.46 | < .001 | 778.43 | [759.22, 797.63] |
| Maximum Parental Education | -0.52 | .600 | -0.83 | [-3.91, 2.26] |
| Family Income (High) | -0.46 | .648 | -1.11 | [-5.88, 3.66] |
| Family Income (Low) | -0.23 | .819 | -0.61 | [-5.83, 4.61] |
| Sex | 4.22 | < .001 | 6.30 | [3.38, 9.23] |
| Child Race (American Indian/Alaska Native) | -0.80 | .425 | -13.93 | [-48.13, 20.28] |
| Child Race (Asian) | 0.32 | .749 | 3.51 | [-18.01, 25.02] |
| Child Race (Black) | 1.54 | .123 | 13.54 | [-3.69, 30.77] |
| Child Race (Native Hawaiian / Pacific Islander) | -1.10 | .269 | -39.72 | [-110.20, 30.76] |
| Child Race (Other) | 2.30 | .021 | 19.55 | [2.91, 36.18] |
| Child Ethnicity | 1.10 | .272 | 2.36 | [-1.85, 6.56] |
| Age | -0.23 | .821 | -0.04 | [-0.37, 0.30] |
| Lead Risk | -0.38 | .701 | -0.21 | [-1.25, 0.84] |
| Intracranial Volume | 37.16 | < .001 | 0.0004 | [0.0004, 0.0004] |
| Family Income (High) × Lead Risk | 0.94 | .347 | 0.62 | [-0.67, 1.91] |
| Family Income (Low) × Lead Risk | -0.50 | .620 | -0.35 | [-1.74, 1.04] |

**Note**: The linear mixed-effects model incorporates testing the statistical significance of coefficients against a *t*-distribution. Family Income was a categorical, effects-coded factor, in which the level “Mid” served as the reference level. Family Income was operationally defined as the self-reported combined family income and partitioned into three levels: Low Income: ≤ $50K; Mid Income: $50K-$100K; High Income: ≥ $100K. Sex was also a categorical factor, effect coded with Male/Female as ‑1/+1. Child Ethnicity was also a categorical factor, effect coded with Hispanic/Non-Hispanic as ‑1/+1. Child Race was also a categorical factor, in which “White” served as the reference level. Age, Maximum Parental Education (i.e., highest education level between parents/caregivers), Lead Risk, and Intracranial Volume were centered continuous factors. The random effects structure included a random intercept for magnetic resonance imaging (MRI) scanner serial number and family identification number. Random effects were restricted to be uncorrelated. Analysis included 8,524 data points. The model accounted for 63.4% of the variance in the data (*R*^2^ = .634, adjusted *R*^2^ = .633).

**Supplementary Table 18. Linear mixed-effects model output for the analysis of lead risk and volume of the lateral ventricles, collapsed across hemispheres.**

|  | *t*(8508) | *p* | *b* | 95% CI |
| --- | --- | --- | --- | --- |
| Intercept | 23.68 | < .001 | 4679.7 | [4292.33, 5067.08] |
| Maximum Parental Education | -0.64 | .524 | -19.92 | [-81.28, 41.43] |
| Family Income (High) | 0.36 | .721 | 17.29 | [-77.49, 112.07] |
| Family Income (Low) | 0.17 | .868 | 8.83 | [-94.95, 112.61] |
| Sex | 1.67 | .096 | 50.08 | [-8.87, 109.02] |
| Child Race (American Indian/Alaska Native) | -0.48 | .629 | -168.10 | [-850.06, 513.87] |
| Child Race (Asian) | -0.55 | .585 | -118.86 | [-545.34, 307.62] |
| Child Race (Black) | 1.60 | .109 | 277.64 | [-61.81, 617.10] |
| Child Race (Native Hawaiian / Pacific Islander) | 0.50 | .616 | 353.14 | [-1026.51, 1732.79] |
| Child Race (Other) | -0.85 | .397 | -141.52 | [-469.10, 186.06] |
| Child Ethnicity | 0.43 | .667 | 18.40 | [-65.39, 102.19] |
| Age | 2.43 | .015 | 8.51 | [1.65, 15.37] |
| Lead Risk | 1.26 | .209 | 13.37 | [-7.50, 34.24] |
| Intracranial Volume | 30.95 | < .001 | 0.01 | [0.01, 0.01] |
| Family Income (High) × Lead Risk | 1.02 | .309 | 13.29 | [-12.29, 38.87] |
| Family Income (Low) × Lead Risk | -0.27 | .789 | -3.77 | [-31.40, 23.86] |

**Note**: The linear mixed-effects model incorporates testing the statistical significance of coefficients against a *t*-distribution. Family Income was a categorical, effects-coded factor, in which the level “Mid” served as the reference level. Family Income was operationally defined as the self-reported combined family income and partitioned into three levels: Low Income: ≤ $50K; Mid Income: $50K-$100K; High Income: ≥ $100K. Sex was also a categorical factor, effect coded with Male/Female as ‑1/+1. Child Ethnicity was also a categorical factor, effect coded with Hispanic/Non-Hispanic as ‑1/+1. Child Race was also a categorical factor, in which “White” served as the reference level. Age, Maximum Parental Education (i.e., highest education level between parents/caregivers), Lead Risk, and Intracranial Volume were centered continuous factors. The random effects structure included a random intercept for magnetic resonance imaging (MRI) scanner serial number and family identification number. Random effects were restricted to be uncorrelated. Analysis included 8,524 data points. The model accounted for 40.3% of the variance in the data (*R*^2^ = .403, adjusted *R*^2^ = .402).

**Supplementary Table 19. Linear mixed-effects model output for the analysis of lead risk and volume of the inferior lateral ventricles, collapsed across hemispheres.**

|  | *t*(8508) | *p* | *b* | 95% CI |
| --- | --- | --- | --- | --- |
| Intercept | 24.77 | < .001 | 309.87 | [285.35, 334.39] |
| Maximum Parental Education | -1.50 | .134 | -2.44 | [-5.63, 0.75] |
| Family Income (High) | 0.83 | .407 | 2.09 | [-2.85, 7.02] |
| Family Income (Low) | 0.08 | .935 | 0.23 | [-5.18, 5.63] |
| Sex | -4.13 | < .001 | -6.49 | [-9.57, -3.41] |
| Child Race (American Indian/Alaska Native) | -0.30 | .762 | -5.48 | [-40.99, 30.04] |
| Child Race (Asian) | 0.94 | .347 | 10.63 | [-11.53, 32.80] |
| Child Race (Black) | -1.63 | .103 | -14.63 | [-32.22, 2.96] |
| Child Race (Native Hawaiian / Pacific Islander) | 0.84 | .402 | 30.43 | [-40.81, 101.67] |
| Child Race (Other) | -1.24 | .216 | -10.71 | [-27.68, 6.25] |
| Child Ethnicity | -2.45 | .014 | -5.47 | [-9.86, -1.09] |
| Age | 1.55 | .122 | 0.28 | [-0.08, 0.64] |
| Lead Risk | 1.03 | .304 | 0.57 | [-0.52, 1.66] |
| Intracranial Volume | 12.91 | < .001 | 0.0002 | [0.0001, 0.0002] |
| Family Income (High) × Lead Risk | 0.14 | .892 | 0.09 | [-1.24, 1.42] |
| Family Income (Low) × Lead Risk | -1.45 | .147 | -1.06 | [-2.50, 0.38] |

**Note**: The linear mixed-effects model incorporates testing the statistical significance of coefficients against a *t*-distribution. Family Income was a categorical, effects-coded factor, in which the level “Mid” served as the reference level. Family Income was operationally defined as the self-reported combined family income and partitioned into three levels: Low Income: ≤ $50K; Mid Income: $50K-$100K; High Income: ≥ $100K. Sex was also a categorical factor, effect coded with Male/Female as ‑1/+1. Child Ethnicity was also a categorical factor, effect coded with Hispanic/Non-Hispanic as ‑1/+1. Child Race was also a categorical factor, in which “White” served as the reference level. Age, Maximum Parental Education (i.e., highest education level between parents/caregivers), Lead Risk, and Intracranial Volume were centered continuous factors. The random effects structure included a random intercept for magnetic resonance imaging (MRI) scanner serial number and family identification number. Random effects were restricted to be uncorrelated. Analysis included 8,524 data points. The model accounted for 30.2% of the variance in the data (*R*^2^ = .302, adjusted *R*^2^ = .300).

**Supplementary Table 20. Linear mixed-effects model output for the analysis of lead risk and volume of the 3^rd^ ventricle.**

|  | *t*(8508) | *p* | *b* | 95% CI |
| --- | --- | --- | --- | --- |
| Intercept | 47.38 | < .001 | 755.63 | [724.36, 786.89] |
| Maximum Parental Education | -1.03 | .305 | -2.65 | [-7.73, 2.42] |
| Family Income (High) | -0.35 | .729 | -1.39 | [-9.22, 6.45] |
| Family Income (Low) | 2.06 | .040 | 9.01 | [0.43, 17.59] |
| Sex | 0.22 | .825 | 0.55 | [-4.30, 5.40] |
| Child Race (American Indian/Alaska Native) | 0.15 | .882 | 4.27 | [-52.07, 60.61] |
| Child Race (Asian) | 1.84 | .065 | 33.17 | [-2.12, 68.47] |
| Child Race (Black) | 1.12 | .262 | 16.11 | [-12.05, 44.26] |
| Child Race (Native Hawaiian / Pacific Islander) | -0.38 | .704 | -22.25 | [-136.96, 92.46] |
| Child Race (Other) | -0.87 | .386 | -12.02 | [-39.20, 15.16] |
| Child Ethnicity | -0.22 | .823 | -0.79 | [-7.70, 6.13] |
| Age | 0.30 | .765 | 0.09 | [-0.48, 0.65] |
| Lead Risk | 1.52 | .129 | 1.34 | [-0.39, 3.06] |
| Intracranial Volume | 31.94 | < .001 | 0.001 | [0.001, 0.001] |
| Family Income (High) × Lead Risk | -0.67 | .503 | -0.72 | [-2.84, 1.39] |
| Family Income (Low) × Lead Risk | -0.62 | .538 | -0.72 | [-3.00, 1.57] |

**Note**: The linear mixed-effects model incorporates testing the statistical significance of coefficients against a *t*-distribution. Family Income was a categorical, effects-coded factor, in which the level “Mid” served as the reference level. Family Income was operationally defined as the self-reported combined family income and partitioned into three levels: Low Income: ≤ $50K; Mid Income: $50K-$100K; High Income: ≥ $100K. Sex was also a categorical factor, effect coded with Male/Female as ‑1/+1. Child Ethnicity was also a categorical factor, effect coded with Hispanic/Non-Hispanic as ‑1/+1. Child Race was also a categorical factor, in which “White” served as the reference level. Age, Maximum Parental Education (i.e., highest education level between parents/caregivers), Lead Risk, and Intracranial Volume were centered continuous factors. The random effects structure included a random intercept for magnetic resonance imaging (MRI) scanner serial number and family identification number. Random effects were restricted to be uncorrelated. Analysis included 8,524 data points. The model accounted for 47.3% of the variance in the data (*R*^2^ = .473, adjusted *R*^2^ = .472).

**Supplementary Table 21. Linear mixed-effects model output for the analysis of lead risk and volume of the 4^th^ ventricle.**

|  | *t*(8508) | *p* | *b* | 95% CI |
| --- | --- | --- | --- | --- |
| Intercept | 46.71 | < .001 | 1793.59 | [1718.33, 1868.86] |
| Maximum Parental Education | 3.72 | < .001 | 25.69 | [12.16, 39.22] |
| Family Income (High) | 2.38 | .017 | 25.32 | [4.49, 46.16] |
| Family Income (Low) | -0.11 | .911 | -1.31 | [-24.15, 21.53] |
| Sex | -8.45 | < .001 | -55.26 | [-68.09, -42.43] |
| Child Race (American Indian/Alaska Native) | -0.75 | .456 | -57.16 | [-207.35, 93.02] |
| Child Race (Asian) | 0.59 | .558 | 28.13 | [-66.06, 122.32] |
| Child Race (Black) | 0.25 | .803 | 9.59 | [-65.72, 84.89] |
| Child Race (Native Hawaiian / Pacific Islander) | 1.02 | .309 | 160.16 | [-148.32, 468.65] |
| Child Race (Other) | -1.47 | .141 | -54.81 | [-127.71, 18.10] |
| Child Ethnicity | -0.92 | .356 | -8.48 | [-26.51, 9.55] |
| Age | 1.71 | .086 | 1.30 | [-0.19, 2.78] |
| Lead Risk | 0.24 | .812 | 0.55 | [-3.97, 5.06] |
| Intracranial Volume | 17.78 | < .001 | 0.001 | [0.001, 0.001] |
| Family Income (High) × Lead Risk | 3.64 | < .001 | 10.45 | [4.82, 16.08] |
| Family Income (Low) × Lead Risk | -0.37 | .711 | -1.15 | [-7.23, 4.93] |

**Note**: The linear mixed-effects model incorporates testing the statistical significance of coefficients against a *t*-distribution. Family Income was a categorical, effects-coded factor, in which the level “Mid” served as the reference level. Family Income was operationally defined as the self-reported combined family income and partitioned into three levels: Low Income: ≤ $50K; Mid Income: $50K-$100K; High Income: ≥ $100K. Sex was also a categorical factor, effect coded with Male/Female as ‑1/+1. Child Ethnicity was also a categorical factor, effect coded with Hispanic/Non-Hispanic as ‑1/+1. Child Race was also a categorical factor, in which “White” served as the reference level. Age, Maximum Parental Education (i.e., highest education level between parents/caregivers), Lead Risk, and Intracranial Volume were centered continuous factors. The random effects structure included a random intercept for magnetic resonance imaging (MRI) scanner serial number and family identification number. Random effects were restricted to be uncorrelated. Analysis included 8,524 data points. The model accounted for 50.3% of the variance in the data (*R*^2^ = .503, adjusted *R*^2^ = .503).

**Supplementary Table 22. Linear mixed-effects model output for the analysis of lead risk and subcortical gray matter volume.**

|  | *t*(8508) | *p* | *b* | 95% CI |
| --- | --- | --- | --- | --- |
| Intercept | 222.48 | < .001 | 60413.53 | [59881.23, 60945.83] |
| Maximum Parental Education | 2.04 | .041 | 71.90 | [2.83, 140.97] |
| Family Income (High) | 1.82 | .068 | 99.39 | [-7.39, 206.17] |
| Family Income (Low) | -2.72 | .007 | -161.93 | [-278.83, -45.04] |
| Sex | -6.51 | < .001 | -216.88 | [-282.16, -151.60] |
| Child Race (American Indian/Alaska Native) | -0.86 | .392 | -333.72 | [-1098.22, 430.78] |
| Child Race (Asian) | 0.33 | .741 | 81.19 | [-400.44, 562.83] |
| Child Race (Black) | -1.04 | .297 | -205.61 | [-591.71, 180.48] |
| Child Race (Native Hawaiian / Pacific Islander) | 0.44 | .662 | 352.84 | [-1227.25, 1932.92] |
| Child Race (Other) | 0.19 | .846 | 36.97 | [-335.69, 409.63] |
| Child Ethnicity | -0.84 | .403 | -40.40 | [-135.01, 54.21] |
| Age | 0.21 | .830 | 0.82 | [-6.65, 8.28] |
| Lead Risk | -1.23 | .220 | -14.77 | [-38.38, 8.83] |
| Intracranial Volume | 113.83 | < .001 | 0.03 | [0.03, 0.03] |
| Family Income (High) × Lead Risk | 0.57 | .567 | 8.43 | [-20.41, 37.26] |
| Family Income (Low) × Lead Risk | 0.63 | .527 | 10.04 | [-21.07, 41.16] |

**Note**: The linear mixed-effects model incorporates testing the statistical significance of coefficients against a *t*-distribution. Family Income was a categorical, effects-coded factor, in which the level “Mid” served as the reference level. Family Income was operationally defined as the self-reported combined family income and partitioned into three levels: Low Income: ≤ $50K; Mid Income: $50K-$100K; High Income: ≥ $100K. Sex was also a categorical factor, effect coded with Male/Female as ‑1/+1. Child Ethnicity was also a categorical factor, effect coded with Hispanic/Non-Hispanic as ‑1/+1. Child Race was also a categorical factor, in which “White” served as the reference level. Age, Maximum Parental Education (i.e., highest education level between parents/caregivers), Lead Risk, and Intracranial Volume were centered continuous factors. The random effects structure included a random intercept for magnetic resonance imaging (MRI) scanner serial number and family identification number. Random effects were restricted to be uncorrelated. Analysis included 8,524 data points. The model accounted for 91.3% of the variance in the data (*R*^2^ = .913, adjusted *R*^2^ = .913).

**Supplementary Table 23. Linear mixed-effects model output for the analysis of lead risk and volume of the posterior corpus callosum, including a Sex × Lead Risk interaction.**

|  | *t*(8509) | *p* | *b* | 95% CI |
| --- | --- | --- | --- | --- |
| Intercept | 90.70 | < .001 | 796.12 | [778.91, 813.32] |
| Maximum Parental Education | 0.95 | .343 | 1.48 | [-1.58, 4.54] |
| Family Income (High) | -0.25 | .799 | -0.59 | [-5.14, 3.96] |
| Family Income (Low) | -0.45 | .656 | -1.18 | [-6.37, 4.01] |
| Sex | 1.99 | .046 | 2.98 | [0.05, 5.91] |
| Child Race (American Indian/Alaska Native) | 0.48 | .629 | 8.41 | [-25.74, 42.55] |
| Child Race (Asian) | 0.73 | .466 | 7.99 | [-13.48, 29.46] |
| Child Race (Black) | -0.01 | .995 | -0.05 | [-17.20, 17.09] |
| Child Race (Native Hawaiian / Pacific Islander) | -0.65 | .518 | -23.28 | [-93.82, 47.25] |
| Child Race (Other) | 0.99 | .321 | 8.43 | [-8.21, 25.07] |
| Child Ethnicity | -1.81 | .070 | -3.79 | [-7.90, 0.31] |
| Age | 7.37 | < .001 | 1.26 | [0.92, 1.59] |
| Lead Risk | -1.98 | .048 | -1.02 | [-2.03, -0.01] |
| Intracranial Volume | 28.67 | < .001 | 0.0003 | [0.0003, 0.0003] |
| Sex × Lead Risk | 0.35 | .727 | 0.15 | [-0.68, 0.98] |

**Note**: The linear mixed-effects model incorporates testing the statistical significance of coefficients against a *t*-distribution. Family Income was a categorical, effects-coded factor, in which the level “Mid” served as the reference level. Family Income was operationally defined as the self-reported combined family income and partitioned into three levels: Low Income: ≤ $50K; Mid Income: $50K-$100K; High Income: ≥ $100K. Sex was also a categorical factor, effect coded with Male/Female as ‑1/+1. Child Ethnicity was also a categorical factor, effect coded with Hispanic/Non-Hispanic as ‑1/+1. Child Race was also a categorical factor, in which “White” served as the reference level. Age, Maximum Parental Education (i.e., highest education level between parents/caregivers), Lead Risk, and Intracranial Volume were centered continuous factors. The random effects structure included a random intercept for magnetic resonance imaging (MRI) scanner serial number and family identification number. Random effects were restricted to be uncorrelated. Analysis included 8,524 data points. The model accounted for 60.3% of the variance in the data (*R*^2^ = .603, adjusted *R*^2^ = .602).

**Supplementary Table 24. Linear mixed-effects model output for the analysis of lead risk and volume of the mid-posterior corpus callosum, including a Sex × Lead Risk interaction.**

|  | *t*(8509) | *p* | *b* | 95% CI |
| --- | --- | --- | --- | --- |
| Intercept | 70.88 | < .001 | 375.96 | [365.57, 386.36] |
| Maximum Parental Education | 0.71 | .476 | 0.68 | [-1.20, 2.56] |
| Family Income (High) | -1.21 | .225 | -1.73 | [-4.52, 1.06] |
| Family Income (Low) | -0.16 | .873 | -0.26 | [-3.45, 2.92] |
| Sex | 1.79 | .073 | 1.66 | [-0.15, 3.48] |
| Child Race (American Indian/Alaska Native) | 1.15 | .250 | 12.34 | [-8.67, 33.36] |
| Child Race (Asian) | -0.53 | .594 | -3.58 | [-16.73, 9.57] |
| Child Race (Black) | -1.40 | .161 | -7.48 | [-17.93, 2.97] |
| Child Race (Native Hawaiian / Pacific Islander) | -0.85 | .395 | -18.57 | [-61.39, 24.26] |
| Child Race (Other) | 1.51 | .131 | 7.82 | [-2.32, 17.97] |
| Child Ethnicity | 0.70 | .486 | 0.89 | [-1.62, 3.40] |
| Age | 7.55 | < .001 | 0.81 | [0.60, 1.02] |
| Lead Risk | -3.24 | .001 | -1.02 | [-1.64, -0.40] |
| Intracranial Volume | 19.23 | < .001 | 0.0001 | [0.0001, 0.0001] |
| Sex × Lead Risk | -0.17 | .867 | -0.04 | [-0.56, 0.47] |

**Note**: The linear mixed-effects model incorporates testing the statistical significance of coefficients against a *t*-distribution. Family Income was a categorical, effects-coded factor, in which the level “Mid” served as the reference level. Family Income was operationally defined as the self-reported combined family income and partitioned into three levels: Low Income: ≤ $50K; Mid Income: $50K-$100K; High Income: ≥ $100K. Sex was also a categorical factor, effect coded with Male/Female as ‑1/+1. Child Ethnicity was also a categorical factor, effect coded with Hispanic/Non-Hispanic as ‑1/+1. Child Race was also a categorical factor, in which “White” served as the reference level. Age, Maximum Parental Education (i.e., highest education level between parents/caregivers), Lead Risk, and Intracranial Volume were centered continuous factors. The random effects structure included a random intercept for magnetic resonance imaging (MRI) scanner serial number and family identification number. Random effects were restricted to be uncorrelated. Analysis included 8,524 data points. The model accounted for 43.0% of the variance in the data (*R*^2^ = .430, adjusted *R*^2^ = .429).

**Supplementary Table 25. Linear mixed-effects model output for the analysis of lead risk and volume of the central corpus callosum, including a Sex × Lead Risk interaction.**

|  | *t*(8509) | *p* | *b* | 95% CI |
| --- | --- | --- | --- | --- |
| Intercept | 66.75 | < .001 | 405.26 | [393.36, 417.16] |
| Maximum Parental Education | -0.65 | .516 | -0.69 | [-2.77, 1.39] |
| Family Income (High) | -1.42 | .157 | -2.24 | [-5.34, 0.86] |
| Family Income (Low) | -0.02 | .981 | -0.04 | [-3.58, 3.49] |
| Sex | 3.30 | .001 | 3.41 | [1.39, 5.44] |
| Child Race (American Indian/Alaska Native) | 0.32 | .748 | 3.82 | [-19.49, 27.13] |
| Child Race (Asian) | 1.63 | .103 | 12.11 | [-2.47, 26.69] |
| Child Race (Black) | 0.70 | .487 | 4.11 | [-7.47, 15.69] |
| Child Race (Native Hawaiian / Pacific Islander) | -1.44 | .149 | -34.80 | [-82.08, 12.48] |
| Child Race (Other) | 1.78 | .076 | 10.17 | [-1.05, 21.39] |
| Child Ethnicity | 1.35 | .178 | 1.94 | [-0.88, 4.76] |
| Age | 4.97 | < .001 | 0.59 | [0.36, 0.83] |
| Lead Risk | -3.36 | .001 | -1.19 | [-1.88, -0.50] |
| Intracranial Volume | 17.61 | < .001 | 0.0001 | [0.0001, 0.0002] |
| Sex × Lead Risk | -0.31 | .755 | -0.09 | [-0.67, 0.48] |

**Note**: The linear mixed-effects model incorporates testing the statistical significance of coefficients against a *t*-distribution. Family Income was a categorical, effects-coded factor, in which the level “Mid” served as the reference level. Family Income was operationally defined as the self-reported combined family income and partitioned into three levels: Low Income: ≤ $50K; Mid Income: $50K-$100K; High Income: ≥ $100K. Sex was also a categorical factor, effect coded with Male/Female as ‑1/+1. Child Ethnicity was also a categorical factor, effect coded with Hispanic/Non-Hispanic as ‑1/+1. Child Race was also a categorical factor, in which “White” served as the reference level. Age, Maximum Parental Education (i.e., highest education level between parents/caregivers), Lead Risk, and Intracranial Volume were centered continuous factors. The random effects structure included a random intercept for magnetic resonance imaging (MRI) scanner serial number and family identification number. Random effects were restricted to be uncorrelated. Analysis included 8,524 data points. The model accounted for 36.9% of the variance in the data (*R*^2^ = .369, adjusted *R*^2^ = .368).

**Supplementary Table 26. Linear mixed-effects model output for the analysis of lead risk and volume of the mid-anterior corpus callosum, including a Sex × Lead Risk interaction.**

|  | *t*(8509) | *p* | *b* | 95% CI |
| --- | --- | --- | --- | --- |
| Intercept | 63.18 | < .001 | 433.82 | [420.36, 447.28] |
| Maximum Parental Education | -2.25 | .024 | -2.71 | [-5.07, -0.35] |
| Family Income (High) | -1.57 | .116 | -2.81 | [-6.32, 0.70] |
| Family Income (Low) | 0.70 | .482 | 1.44 | [-2.57, 5.44] |
| Sex | -0.06 | .954 | -0.07 | [-2.36, 2.23] |
| Child Race (American Indian/Alaska Native) | 0.58 | .564 | 7.77 | [-18.63, 34.17] |
| Child Race (Asian) | 2.54 | .011 | 21.37 | [4.86, 37.87] |
| Child Race (Black) | -1.29 | .197 | -8.62 | [-21.73, 4.49] |
| Child Race (Native Hawaiian / Pacific Islander) | -1.37 | .170 | -37.49 | [-91.02, 16.03] |
| Child Race (Other) | 1.70 | .090 | 11.00 | [-1.70, 23.70] |
| Child Ethnicity | 0.69 | .492 | 1.12 | [-2.08, 4.32] |
| Age | 0.05 | .961 | 0.01 | [-0.26, 0.27] |
| Lead Risk | -3.66 | < .001 | -1.47 | [-2.25, -0.68] |
| Intracranial Volume | 23.68 | < .001 | 0.0002 | [0.0002, 0.0002] |
| Sex × Lead Risk | 0.57 | .570 | 0.19 | [-0.46, 0.84] |

**Note**: The linear mixed-effects model incorporates testing the statistical significance of coefficients against a *t*-distribution. Family Income was a categorical, effects-coded factor, in which the level “Mid” served as the reference level. Family Income was operationally defined as the self-reported combined family income and partitioned into three levels: Low Income: ≤ $50K; Mid Income: $50K-$100K; High Income: ≥ $100K. Sex was also a categorical factor, effect coded with Male/Female as ‑1/+1. Child Ethnicity was also a categorical factor, effect coded with Hispanic/Non-Hispanic as ‑1/+1. Child Race was also a categorical factor, in which “White” served as the reference level. Age, Maximum Parental Education (i.e., highest education level between parents/caregivers), Lead Risk, and Intracranial Volume were centered continuous factors. The random effects structure included a random intercept for magnetic resonance imaging (MRI) scanner serial number and family identification number. Random effects were restricted to be uncorrelated. Analysis included 8,524 data points. The model accounted for 39.9% of the variance in the data (*R*^2^ = .399, adjusted *R*^2^ = .398).

**Supplementary Table 27. Linear mixed-effects model output for the analysis of area deprivation index (ADI) and volume of the thalamus proper, collapsed across hemispheres.**

|  | *t*(8508) | *p* | *b* | 95% CI |
| --- | --- | --- | --- | --- |
| Intercept | 229.61 | < .001 | 7528.28 | [7464.01, 7592.55] |
| Maximum Parental Education | -0.19 | .853 | -1.04 | [-12.04, 9.96] |
| Family Income (High) | -0.61 | .544 | -6.36 | [-26.91, 14.19] |
| Family Income (Low) | -0.51 | .613 | -4.90 | [-23.90, 14.10] |
| Sex | -3.98 | < .001 | -21.05 | [-31.41, -10.70] |
| Child Race (American Indian/Alaska Native) | -0.35 | .724 | -21.77 | [-142.76, 99.21] |
| Child Race (Asian) | -0.43 | .665 | -16.78 | [-92.83, 59.26] |
| Child Race (Black) | -0.31 | .758 | -9.57 | [-70.41, 51.28] |
| Child Race (Native Hawaiian / Pacific Islander) | 0.49 | .624 | 62.04 | [-186.38, 310.47] |
| Child Race (Other) | 0.39 | .696 | 11.72 | [-47.00, 70.44] |
| Child Ethnicity | -0.97 | .334 | -7.29 | [-22.06, 7.48] |
| Age | 6.29 | < .001 | 3.83 | [2.63, 5.02] |
| ADI | -2.31 | .021 | -6.34 | [-11.73, -0.95] |
| Intracranial Volume | 93.76 | < .001 | 0.004 | [0.004, 0.004] |
| Family Income (High) × ADI | -0.41 | .684 | -1.33 | [-7.77, 5.10] |
| Family Income (Low) × ADI | -0.91 | .365 | -2.66 | [-8.42, 3.10] |

**Note**: The linear mixed-effects model incorporates testing the statistical significance of coefficients against a *t*-distribution. Family Income was a categorical, effects-coded factor, in which the level “Mid” served as the reference level. Family Income was operationally defined as the self-reported combined family income and partitioned into three levels: Low Income: ≤ $50K; Mid Income: $50K-$100K; High Income: ≥ $100K. Sex was also a categorical factor, effect coded with Male/Female as ‑1/+1. Child Ethnicity was also a categorical factor, effect coded with Hispanic/Non-Hispanic as ‑1/+1. Child Race was also a categorical factor, in which “White” served as the reference level. Age, Maximum Parental Education (i.e., highest education level between parents/caregivers), Area Deprivation Index (ADI), and Intracranial Volume were centered continuous factors. The random effects structure included a random intercept for magnetic resonance imaging (MRI) scanner serial number and family identification number. Random effects were restricted to be uncorrelated. Analysis included 8,524 data points. The model accounted for 86.0% of the variance in the data (*R*^2^ = .860, adjusted *R*^2^ = .859).

**Supplementary Table 28. Linear mixed-effects model output for the analysis of area deprivation index (ADI) and volume of the caudate, collapsed across hemispheres.**

|  | *t*(8508) | *p* | *b* | 95% CI |
| --- | --- | --- | --- | --- |
| Intercept | 94.97 | < .001 | 4022.76 | [3939.73, 4105.80] |
| Maximum Parental Education | 1.94 | .052 | 10.73 | [-0.11, 21.56] |
| Family Income (High) | -1.19 | .234 | -12.29 | [-32.53, 7.94] |
| Family Income (Low) | -0.47 | .636 | -4.52 | [-23.23, 14.19] |
| Sex | 7.62 | < .001 | 39.43 | [29.29, 49.57] |
| Child Race (American Indian/Alaska Native) | -2.41 | .016 | -146.22 | [-265.02, -27.41] |
| Child Race (Asian) | -0.33 | .743 | -12.53 | [-87.43, 62.37] |
| Child Race (Black) | 1.44 | .149 | 44.20 | [-15.88, 104.28] |
| Child Race (Native Hawaiian / Pacific Islander) | 0.04 | .965 | 5.57 | [-240.09, 251.24] |
| Child Race (Other) | 1.53 | .126 | 45.27 | [-12.67, 103.21] |
| Child Ethnicity | 1.29 | .196 | 9.70 | [-5.00, 24.40] |
| Age | -3.52 | < .001 | -2.08 | [-3.24, -0.92] |
| ADI | -1.93 | .053 | -5.39 | [-10.85, 0.08] |
| Intracranial Volume | 58.45 | < .001 | 0.002 | [0.002, 0.002] |
| Family Income (High) × ADI | -0.90 | .368 | -2.91 | [-9.25, 3.42] |
| Family Income (Low) × ADI | 0.18 | .854 | 0.53 | [-5.14, 6.20] |

**Note**: The linear mixed-effects model incorporates testing the statistical significance of coefficients against a *t*-distribution. Family Income was a categorical, effects-coded factor, in which the level “Mid” served as the reference level. Family Income was operationally defined as the self-reported combined family income and partitioned into three levels: Low Income: ≤ $50K; Mid Income: $50K-$100K; High Income: ≥ $100K. Sex was also a categorical factor, effect coded with Male/Female as ‑1/+1. Child Ethnicity was also a categorical factor, effect coded with Hispanic/Non-Hispanic as ‑1/+1. Child Race was also a categorical factor, in which “White” served as the reference level. Age, Maximum Parental Education (i.e., highest education level between parents/caregivers), Area Deprivation Index (ADI), and Intracranial Volume were centered continuous factors. The random effects structure included a random intercept for magnetic resonance imaging (MRI) scanner serial number and family identification number. Random effects were restricted to be uncorrelated. Analysis included 8,524 data points. The model accounted for 76.4% of the variance in the data (*R*^2^ = .764, adjusted *R*^2^ = .764).

**Supplementary Table 29. Linear mixed-effects model output for the analysis of area deprivation index (ADI) and volume of the putamen, collapsed across hemispheres.**

|  | *t*(8508) | *p* | *b* | 95% CI |
| --- | --- | --- | --- | --- |
| Intercept | 148.94 | < .001 | 5886.4 | [5808.92, 5963.87] |
| Maximum Parental Education | -0.22 | .824 | -1.51 | [-14.79, 11.77] |
| Family Income (High) | -0.68 | .496 | -8.62 | [-33.43, 16.20] |
| Family Income (Low) | -1.62 | .104 | -19.01 | [-41.96, 3.93] |
| Sex | -9.61 | < .001 | -61.08 | [-73.54, -48.63] |
| Child Race (American Indian/Alaska Native) | -1.56 | .118 | -116.45 | [-262.38, 29.47] |
| Child Race (Asian) | 3.17 | .002 | 148.52 | [56.66, 240.38] |
| Child Race (Black) | -1.74 | .081 | -65.44 | [-139.03, 8.16] |
| Child Race (Native Hawaiian / Pacific Islander) | 0.26 | .793 | 40.25 | [-260.72, 341.22] |
| Child Race (Other) | 0.00 | .999 | 0.02 | [-71.02, 71.07] |
| Child Ethnicity | -1.39 | .165 | -12.62 | [-30.44, 5.19] |
| Age | -3.88 | < .001 | -2.83 | [-4.26, -1.40] |
| ADI | -2.43 | .015 | -8.06 | [-14.56, -1.56] |
| Intracranial Volume | 51.02 | < .001 | 0.002 | [0.002, 0.003] |
| Family Income (High) × ADI | -1.92 | .055 | -7.61 | [-15.38, 0.15] |
| Family Income (Low) × ADI | 0.52 | .604 | 1.84 | [-5.12, 8.79] |

**Note**: The linear mixed-effects model incorporates testing the statistical significance of coefficients against a *t*-distribution. Family Income was a categorical, effects-coded factor, in which the level “Mid” served as the reference level. Family Income was operationally defined as the self-reported combined family income and partitioned into three levels: Low Income: ≤ $50K; Mid Income: $50K-$100K; High Income: ≥ $100K. Sex was also a categorical factor, effect coded with Male/Female as ‑1/+1. Child Ethnicity was also a categorical factor, effect coded with Hispanic/Non-Hispanic as ‑1/+1. Child Race was also a categorical factor, in which “White” served as the reference level. Age, Maximum Parental Education (i.e., highest education level between parents/caregivers), Area Deprivation Index (ADI), and Intracranial Volume were centered continuous factors. The random effects structure included a random intercept for magnetic resonance imaging (MRI) scanner serial number and family identification number. Random effects were restricted to be uncorrelated. Analysis included 8,524 data points. The model accounted for 75.3% of the variance in the data (*R*^2^ = .753, adjusted *R*^2^ = .753).

**Supplementary Table 30. Linear mixed-effects model output for the analysis of area deprivation index (ADI) and volume of the pallidum, collapsed across hemispheres.**

|  | *t*(8508) | *p* | *b* | 95% CI |
| --- | --- | --- | --- | --- |
| Intercept | 111.55 | < .001 | 1783.5 | [1752.16, 1814.85] |
| Maximum Parental Education | 1.30 | .195 | 2.65 | [-1.36, 6.66] |
| Family Income (High) | 0.11 | .914 | 0.41 | [-7.06, 7.88] |
| Family Income (Low) | -0.60 | .552 | -2.10 | [-9.01, 4.81] |
| Sex | -10.67 | < .001 | -20.72 | [-24.53, -16.92] |
| Child Race (American Indian/Alaska Native) | 0.05 | .961 | 1.09 | [-42.98, 45.16] |
| Child Race (Asian) | 0.12 | .903 | 1.72 | [-25.88, 29.33] |
| Child Race (Black) | 0.84 | .400 | 9.46 | [-12.56, 31.47] |
| Child Race (Native Hawaiian / Pacific Islander) | 0.07 | .947 | 3.00 | [-86.40, 92.41] |
| Child Race (Other) | -0.32 | .746 | -3.50 | [-24.71, 17.71] |
| Child Ethnicity | -1.76 | .078 | -4.90 | [-10.34, 0.54] |
| Age | -7.52 | < .001 | -1.69 | [-2.14, -1.25] |
| ADI | -2.20 | .028 | -2.27 | [-4.29, -0.25] |
| Intracranial Volume | 53.02 | < .001 | 0.001 | [0.001, 0.001] |
| Family Income (High) × ADI | -1.03 | .301 | -1.23 | [-3.57, 1.11] |
| Family Income (Low) × ADI | 0.32 | .746 | 0.35 | [-1.75, 2.44] |

**Note**: The linear mixed-effects model incorporates testing the statistical significance of coefficients against a *t*-distribution. Family Income was a categorical, effects-coded factor, in which the level “Mid” served as the reference level. Family Income was operationally defined as the self-reported combined family income and partitioned into three levels: Low Income: ≤ $50K; Mid Income: $50K-$100K; High Income: ≥ $100K. Sex was also a categorical factor, effect coded with Male/Female as ‑1/+1. Child Ethnicity was also a categorical factor, effect coded with Hispanic/Non-Hispanic as ‑1/+1. Child Race was also a categorical factor, in which “White” served as the reference level. Age, Maximum Parental Education (i.e., highest education level between parents/caregivers), Area Deprivation Index (ADI), and Intracranial Volume were centered continuous factors. The random effects structure included a random intercept for magnetic resonance imaging (MRI) scanner serial number and family identification number. Random effects were restricted to be uncorrelated. Analysis included 8,524 data points. The model accounted for 64.7% of the variance in the data (*R*^2^ = .647, adjusted *R*^2^ = .647).

**Supplementary Table 31. Linear mixed-effects model output for the analysis of area deprivation index (ADI) and volume of the hippocampus, collapsed across hemispheres.**

|  | *t*(8508) | *p* | *b* | 95% CI |
| --- | --- | --- | --- | --- |
| Intercept | 187.62 | < .001 | 4091.05 | [4048.31, 4133.79] |
| Maximum Parental Education | 2.32 | .021 | 9.20 | [1.41, 16.99] |
| Family Income (High) | 2.07 | .038 | 15.41 | [0.83, 29.98] |
| Family Income (Low) | -1.99 | .047 | -13.66 | [-27.14, -0.18] |
| Sex | -3.39 | .001 | -12.66 | [-19.97, -5.35] |
| Child Race (American Indian/Alaska Native) | 0.66 | .507 | 29.09 | [-56.80, 114.98] |
| Child Race (Asian) | -0.33 | .743 | -9.01 | [-63.00, 44.97] |
| Child Race (Black) | -4.50 | < .001 | -99.19 | [-142.39, -55.99] |
| Child Race (Native Hawaiian / Pacific Islander) | 1.41 | .159 | 127.28 | [-49.68, 304.25] |
| Child Race (Other) | -1.64 | .101 | -34.94 | [-76.73, 6.85] |
| Child Ethnicity | -1.05 | .293 | -5.48 | [-15.71, 4.74] |
| Age | 1.85 | .064 | 0.80 | [-0.05, 1.64] |
| ADI | -1.03 | .303 | -1.91 | [-5.53, 1.72] |
| Intracranial Volume | 62.40 | < .001 | 0.002 | [0.002, 0.002] |
| Family Income (High) × ADI | 0.27 | .785 | 0.63 | [-3.93, 5.19] |
| Family Income (Low) × ADI | -0.62 | .532 | -1.30 | [-5.39, 2.78] |

**Note**: The linear mixed-effects model incorporates testing the statistical significance of coefficients against a *t*-distribution. Family Income was a categorical, effects-coded factor, in which the level “Mid” served as the reference level. Family Income was operationally defined as the self-reported combined family income and partitioned into three levels: Low Income: ≤ $50K; Mid Income: $50K-$100K; High Income: ≥ $100K. Sex was also a categorical factor, effect coded with Male/Female as ‑1/+1. Child Ethnicity was also a categorical factor, effect coded with Hispanic/Non-Hispanic as ‑1/+1. Child Race was also a categorical factor, in which “White” served as the reference level. Age, Maximum Parental Education (i.e., highest education level between parents/caregivers), Area Deprivation Index (ADI), and Intracranial Volume were centered continuous factors. The random effects structure included a random intercept for magnetic resonance imaging (MRI) scanner serial number and family identification number. Random effects were restricted to be uncorrelated. Analysis included 8,524 data points. The model accounted for 78.4% of the variance in the data (*R*^2^ = .784, adjusted *R*^2^ = .784).

**Supplementary Table 32. Linear mixed-effects model output for the analysis of area deprivation index (ADI) and volume of the amygdala, collapsed across hemispheres.**

|  | *t*(8508) | *p* | *b* | 95% CI |
| --- | --- | --- | --- | --- |
| Intercept | 101.17 | < .001 | 1590.17 | [1559.36, 1620.98] |
| Maximum Parental Education | 0.88 | .379 | 1.74 | [-2.14, 5.62] |
| Family Income (High) | 0.82 | .414 | 3.01 | [-4.22, 10.24] |
| Family Income (Low) | -1.01 | .313 | -3.44 | [-10.13, 3.25] |
| Sex | -12.74 | < .001 | -23.97 | [-27.65, -20.28] |
| Child Race (American Indian/Alaska Native) | 1.88 | .060 | 40.86 | [-1.80, 83.52] |
| Child Race (Asian) | 0.98 | .327 | 13.35 | [-13.37, 40.06] |
| Child Race (Black) | -3.29 | .001 | -35.76 | [-57.05, -14.47] |
| Child Race (Native Hawaiian / Pacific Islander) | 0.07 | .940 | 3.29 | [-83.12, 89.70] |
| Child Race (Other) | -1.58 | .113 | -16.58 | [-37.09, 3.93] |
| Child Ethnicity | -1.19 | .235 | -3.19 | [-8.46, 2.08] |
| Age | 1.28 | .201 | 0.28 | [-0.15, 0.71] |
| ADI | -2.69 | .007 | -2.68 | [-4.64, -0.73] |
| Intracranial Volume | 54.89 | < .001 | 0.001 | [0.001, 0.001] |
| Family Income (High) × ADI | -0.35 | .725 | -0.41 | [-2.67, 1.86] |
| Family Income (Low) × ADI | -0.82 | .412 | -0.85 | [-2.88, 1.18] |

**Note**: The linear mixed-effects model incorporates testing the statistical significance of coefficients against a *t*-distribution. Family Income was a categorical, effects-coded factor, in which the level “Mid” served as the reference level. Family Income was operationally defined as the self-reported combined family income and partitioned into three levels: Low Income: ≤ $50K; Mid Income: $50K-$100K; High Income: ≥ $100K. Sex was also a categorical factor, effect coded with Male/Female as ‑1/+1. Child Ethnicity was also a categorical factor, effect coded with Hispanic/Non-Hispanic as ‑1/+1. Child Race was also a categorical factor, in which “White” served as the reference level. Age, Maximum Parental Education (i.e., highest education level between parents/caregivers), Area Deprivation Index (ADI), and Intracranial Volume were centered continuous factors. The random effects structure included a random intercept for magnetic resonance imaging (MRI) scanner serial number and family identification number. Random effects were restricted to be uncorrelated. Analysis included 8,524 data points. The model accounted for 73.0% of the variance in the data (*R*^2^ = .730, adjusted *R*^2^ = .730).

**Supplementary Table 33. Linear mixed-effects model output for the analysis of area deprivation index (ADI) and volume of the accumbens area, collapsed across hemispheres.**

|  | *t*(8508) | *p* | *b* | 95% CI |
| --- | --- | --- | --- | --- |
| Intercept | 57.00 | < .001 | 597.63 | [577.07, 618.18] |
| Maximum Parental Education | -0.41 | .683 | -0.40 | [-2.30, 1.51] |
| Family Income (High) | 0.23 | .815 | 0.42 | [-3.13, 3.98] |
| Family Income (Low) | -0.81 | .415 | -1.37 | [-4.65, 1.92] |
| Sex | -0.44 | .661 | -0.40 | [-2.21, 1.41] |
| Child Race (American Indian/Alaska Native) | 0.90 | .367 | 9.63 | [-11.32, 30.59] |
| Child Race (Asian) | -2.00 | .045 | -13.40 | [-26.53, -0.27] |
| Child Race (Black) | 0.21 | .830 | 1.15 | [-9.32, 11.62] |
| Child Race (Native Hawaiian / Pacific Islander) | 0.61 | .539 | 13.32 | [-29.20, 55.84] |
| Child Race (Other) | -1.41 | .159 | -7.25 | [-17.33, 2.84] |
| Child Ethnicity | -0.14 | .890 | -0.18 | [-2.78, 2.41] |
| Age | -6.40 | < .001 | -0.69 | [-0.90, -0.48] |
| ADI | -2.72 | .007 | -1.34 | [-2.30, -0.37] |
| Intracranial Volume | 53.54 | < .001 | 0.0004 | [0.0004, 0.0004] |
| Family Income (High) × ADI | 0.51 | .612 | 0.29 | [-0.83, 1.40] |
| Family Income (Low) × ADI | -1.07 | .286 | -0.54 | [-1.54, 0.45] |

**Note**: The linear mixed-effects model incorporates testing the statistical significance of coefficients against a *t*-distribution. Family Income was a categorical, effects-coded factor, in which the level “Mid” served as the reference level. Family Income was operationally defined as the self-reported combined family income and partitioned into three levels: Low Income: ≤ $50K; Mid Income: $50K-$100K; High Income: ≥ $100K. Sex was also a categorical factor, effect coded with Male/Female as ‑1/+1. Child Ethnicity was also a categorical factor, effect coded with Hispanic/Non-Hispanic as ‑1/+1. Child Race was also a categorical factor, in which “White” served as the reference level. Age, Maximum Parental Education (i.e., highest education level between parents/caregivers), Area Deprivation Index (ADI), and Intracranial Volume were centered continuous factors. The random effects structure included a random intercept for magnetic resonance imaging (MRI) scanner serial number and family identification number. Random effects were restricted to be uncorrelated. Analysis included 8,524 data points. The model accounted for 67.4% of the variance in the data (*R*^2^ = .674, adjusted *R*^2^ = .674).

**Supplementary Table 34. Linear mixed-effects model output for the analysis of area deprivation index (ADI) and volume of the ventral diencephalon, collapsed across hemispheres.**

|  | *t*(8508) | *p* | *b* | 95% CI |
| --- | --- | --- | --- | --- |
| Intercept | 155.09 | < .001 | 3937.23 | [3887.47, 3987.00] |
| Maximum Parental Education | 1.30 | .192 | 4.31 | [-2.17, 10.78] |
| Family Income (High) | 1.00 | .319 | 6.14 | [-5.94, 18.21] |
| Family Income (Low) | -0.67 | .504 | -3.81 | [-14.98, 7.36] |
| Sex | -2.01 | .045 | -6.28 | [-12.41, -0.14] |
| Child Race (American Indian/Alaska Native) | 0.77 | .444 | 27.78 | [-43.40, 98.97] |
| Child Race (Asian) | -2.77 | .006 | -63.14 | [-107.80, -18.48] |
| Child Race (Black) | 3.84 | < .001 | 69.95 | [34.28, 105.61] |
| Child Race (Native Hawaiian / Pacific Islander) | -1.31 | .192 | -96.61 | [-241.70, 48.48] |
| Child Race (Other) | 1.77 | .077 | 31.02 | [-3.35, 65.39] |
| Child Ethnicity | -0.20 | .844 | -0.88 | [-9.68, 7.91] |
| Age | 10.07 | < .001 | 3.64 | [2.93, 4.35] |
| ADI | -1.54 | .123 | -2.57 | [-5.83, 0.69] |
| Intracranial Volume | 86.10 | < .001 | 0.002 | [0.002, 0.002] |
| Family Income (High) × ADI | 0.79 | .432 | 1.52 | [-2.27, 5.30] |
| Family Income (Low) × ADI | -0.24 | .810 | -0.42 | [-3.81, 2.97] |

**Note**: The linear mixed-effects model incorporates testing the statistical significance of coefficients against a *t*-distribution. Family Income was a categorical, effects-coded factor, in which the level “Mid” served as the reference level. Family Income was operationally defined as the self-reported combined family income and partitioned into three levels: Low Income: ≤ $50K; Mid Income: $50K-$100K; High Income: ≥ $100K. Sex was also a categorical factor, effect coded with Male/Female as ‑1/+1. Child Ethnicity was also a categorical factor, effect coded with Hispanic/Non-Hispanic as ‑1/+1. Child Race was also a categorical factor, in which “White” served as the reference level. Age, Maximum Parental Education (i.e., highest education level between parents/caregivers), Area Deprivation Index (ADI), and Intracranial Volume were centered continuous factors. The random effects structure included a random intercept for magnetic resonance imaging (MRI) scanner serial number and family identification number. Random effects were restricted to be uncorrelated. Analysis included 8,524 data points. The model accounted for 80.8% of the variance in the data (*R*^2^ = .808, adjusted *R*^2^ = .808).

**Supplementary Table 35. Linear mixed-effects model output for the analysis of area deprivation index (ADI) and volume of the cerebellum cortex, collapsed across hemispheres.**

|  | *t*(8508) | *p* | *b* | 95% CI |
| --- | --- | --- | --- | --- |
| Intercept | 140.40 | < .001 | 52914.03 | [52175.26, 53652.81] |
| Maximum Parental Education | 3.50 | < .001 | 179.68 | [79.06, 280.31] |
| Family Income (High) | 0.32 | .746 | 31.05 | [-156.87, 218.98] |
| Family Income (Low) | -1.39 | .166 | -122.74 | [-296.45, 50.97] |
| Sex | -18.78 | < .001 | -893.91 | [-987.19, -800.62] |
| Child Race (American Indian/Alaska Native) | 0.19 | .852 | 104.61 | [-995.82, 1205.04] |
| Child Race (Asian) | -2.11 | .035 | -747.32 | [-1443.16, -51.47] |
| Child Race (Black) | -0.28 | .777 | -81.00 | [-640.36, 478.36] |
| Child Race (Native Hawaiian / Pacific Islander) | -0.90 | .367 | -1055.05 | [-3348.45, 1238.35] |
| Child Race (Other) | 2.16 | .031 | 594.18 | [54.57, 1133.78] |
| Child Ethnicity | 5.88 | < .001 | 408.81 | [272.54, 545.09] |
| Age | -1.60 | .111 | -8.61 | [-19.18, 1.97] |
| ADI | -2.92 | .003 | -75.54 | [-126.19, -24.89] |
| Intracranial Volume | 56.87 | < .001 | 0.02 | [0.02, 0.02] |
| Family Income (High) × ADI | -0.47 | .640 | -14.03 | [-72.84, 44.78] |
| Family Income (Low) × ADI | 1.31 | .190 | 35.20 | [-17.41, 87.81] |

**Note**: The linear mixed-effects model incorporates testing the statistical significance of coefficients against a *t*-distribution. Family Income was a categorical, effects-coded factor, in which the level “Mid” served as the reference level. Family Income was operationally defined as the self-reported combined family income and partitioned into three levels: Low Income: ≤ $50K; Mid Income: $50K-$100K; High Income: ≥ $100K. Sex was also a categorical factor, effect coded with Male/Female as ‑1/+1. Child Ethnicity was also a categorical factor, effect coded with Hispanic/Non-Hispanic as ‑1/+1. Child Race was also a categorical factor, in which “White” served as the reference level. Age, Maximum Parental Education (i.e., highest education level between parents/caregivers), Area Deprivation Index (ADI), and Intracranial Volume were centered continuous factors. The random effects structure included a random intercept for magnetic resonance imaging (MRI) scanner serial number and family identification number. Random effects were restricted to be uncorrelated. Analysis included 8,524 data points. The model accounted for 86.2% of the variance in the data (*R*^2^ = .862, adjusted *R*^2^ = .862).

**Supplementary Table 36. Linear mixed-effects model output for the analysis of area deprivation index (ADI) and volume of cerebellar white matter, collapsed across hemispheres.**

|  | *t*(8508) | *p* | *b* | 95% CI |
| --- | --- | --- | --- | --- |
| Intercept | 133.22 | < .001 | 17090.41 | [16838.94, 17341.89] |
| Maximum Parental Education | 1.93 | .053 | 42.28 | [-0.60, 85.16] |
| Family Income (High) | 0.42 | .675 | 17.16 | [-62.97, 97.29] |
| Family Income (Low) | -1.47 | .141 | -55.63 | [-129.71, 18.45] |
| Sex | -2.84 | .005 | -57.92 | [-97.96, -17.88] |
| Child Race (American Indian/Alaska Native) | 1.10 | .271 | 264.54 | [-206.06, 735.15] |
| Child Race (Asian) | -1.31 | .189 | -199.02 | [-495.74, 97.70] |
| Child Race (Black) | 2.64 | .008 | 321.04 | [83.01, 559.07] |
| Child Race (Native Hawaiian / Pacific Islander) | -0.94 | .350 | -464.98 | [-1439.81, 509.85] |
| Child Race (Other) | 0.72 | .469 | 84.80 | [-144.99, 314.60] |
| Child Ethnicity | 1.59 | .112 | 46.69 | [-10.83, 104.22] |
| Age | 7.18 | < .001 | 16.78 | [12.20, 21.36] |
| ADI | -0.43 | .669 | -4.58 | [-25.60, 16.44] |
| Intracranial Volume | 50.16 | < .001 | 0.01 | [0.01, 0.01] |
| Family Income (High) × ADI | -1.09 | .277 | -13.92 | [-38.99, 11.16] |
| Family Income (Low) × ADI | 0.74 | .457 | 8.52 | [-13.92, 30.97] |

**Note**: The linear mixed-effects model incorporates testing the statistical significance of coefficients against a *t*-distribution. Family Income was a categorical, effects-coded factor, in which the level “Mid” served as the reference level. Family Income was operationally defined as the self-reported combined family income and partitioned into three levels: Low Income: ≤ $50K; Mid Income: $50K-$100K; High Income: ≥ $100K. Sex was also a categorical factor, effect coded with Male/Female as ‑1/+1. Child Ethnicity was also a categorical factor, effect coded with Hispanic/Non-Hispanic as ‑1/+1. Child Race was also a categorical factor, in which “White” served as the reference level. Age, Maximum Parental Education (i.e., highest education level between parents/caregivers), Area Deprivation Index (ADI), and Intracranial Volume were centered continuous factors. The random effects structure included a random intercept for magnetic resonance imaging (MRI) scanner serial number and family identification number. Random effects were restricted to be uncorrelated. Analysis included 8,524 data points. The model accounted for 78.0% of the variance in the data (*R*^2^ = .780, adjusted *R*^2^ = .780).

**Supplementary Table 37. Linear mixed-effects model output for the analysis of area deprivation index (ADI) and volume of the brain stem.**

|  | *t*(8508) | *p* | *b* | 95% CI |
| --- | --- | --- | --- | --- |
| Intercept | 138.85 | < .001 | 19170.79 | [18900.14, 19441.43] |
| Maximum Parental Education | 1.17 | .243 | 21.22 | [-14.40, 56.84] |
| Family Income (High) | 1.15 | .250 | 39.05 | [-27.46, 105.56] |
| Family Income (Low) | -0.86 | .391 | -26.93 | [-88.42, 34.56] |
| Sex | -8.96 | < .001 | -151.74 | [-184.94, -118.54] |
| Child Race (American Indian/Alaska Native) | 1.01 | .314 | 200.53 | [-189.55, 590.61] |
| Child Race (Asian) | -2.29 | .022 | -287.23 | [-533.45, -41.01] |
| Child Race (Black) | 4.05 | < .001 | 408.36 | [210.68, 606.04] |
| Child Race (Native Hawaiian / Pacific Islander) | -1.20 | .229 | -496.48 | [-1305.68, 312.73] |
| Child Race (Other) | 1.23 | .219 | 119.44 | [-71.22, 310.09] |
| Child Ethnicity | -0.67 | .503 | -16.50 | [-64.79, 31.79] |
| Age | 12.65 | < .001 | 24.42 | [20.64, 28.20] |
| ADI | -0.61 | .544 | -5.55 | [-23.50, 12.40] |
| Intracranial Volume | 74.07 | < .001 | 0.01 | [0.01, 0.01] |
| Family Income (High) × ADI | 0.09 | .931 | 0.91 | [-19.90, 21.73] |
| Family Income (Low) × ADI | 0.20 | .842 | 1.90 | [-16.73, 20.53] |

**Note**: The linear mixed-effects model incorporates testing the statistical significance of coefficients against a *t*-distribution. Family Income was a categorical, effects-coded factor, in which the level “Mid” served as the reference level. Family Income was operationally defined as the self-reported combined family income and partitioned into three levels: Low Income: ≤ $50K; Mid Income: $50K-$100K; High Income: ≥ $100K. Sex was also a categorical factor, effect coded with Male/Female as ‑1/+1. Child Ethnicity was also a categorical factor, effect coded with Hispanic/Non-Hispanic as ‑1/+1. Child Race was also a categorical factor, in which “White” served as the reference level. Age, Maximum Parental Education (i.e., highest education level between parents/caregivers), Area Deprivation Index (ADI), and Intracranial Volume were centered continuous factors. The random effects structure included a random intercept for magnetic resonance imaging (MRI) scanner serial number and family identification number. Random effects were restricted to be uncorrelated. Analysis included 8,524 data points. The model accounted for 85.5% of the variance in the data (*R*^2^ = .855, adjusted *R*^2^ = .855).

**Supplementary Table 38. Linear mixed-effects model output for the analysis of area deprivation index (ADI) and volume of the posterior corpus callosum.**

|  | *t*(8508) | *p* | *b* | 95% CI |
| --- | --- | --- | --- | --- |
| Intercept | 90.27 | < .001 | 796.00 | [778.71, 813.28] |
| Maximum Parental Education | 0.94 | .348 | 1.49 | [-1.62, 4.59] |
| Family Income (High) | 0.00 | .998 | -0.01 | [-5.81, 5.80] |
| Family Income (Low) | -0.70 | .485 | -1.91 | [-7.28, 3.45] |
| Sex | 1.97 | .049 | 2.92 | [0.02, 5.83] |
| Child Race (American Indian/Alaska Native) | 0.49 | .627 | 8.47 | [-25.69, 42.62] |
| Child Race (Asian) | 0.75 | .452 | 8.25 | [-13.25, 29.75] |
| Child Race (Black) | -0.06 | .950 | -0.55 | [-17.77, 16.67] |
| Child Race (Native Hawaiian / Pacific Islander) | -0.65 | .516 | -23.36 | [-93.91, 47.19] |
| Child Race (Other) | 0.99 | .322 | 8.42 | [-8.23, 25.07] |
| Child Ethnicity | -1.68 | .094 | -3.51 | [-7.62, 0.60] |
| Age | 7.38 | < .001 | 1.26 | [0.92, 1.59] |
| ADI | -0.49 | .624 | -0.37 | [-1.84, 1.11] |
| Intracranial Volume | 28.64 | < .001 | 0.0003 | [0.0003, 0.0003] |
| Family Income (High) × ADI | 0.23 | .822 | 0.21 | [-1.61, 2.02] |
| Family Income (Low) × ADI | -0.42 | .673 | -0.35 | [-1.98, 1.28] |

**Note**: The linear mixed-effects model incorporates testing the statistical significance of coefficients against a *t*-distribution. Family Income was a categorical, effects-coded factor, in which the level “Mid” served as the reference level. Family Income was operationally defined as the self-reported combined family income and partitioned into three levels: Low Income: ≤ $50K; Mid Income: $50K-$100K; High Income: ≥ $100K. Sex was also a categorical factor, effect coded with Male/Female as ‑1/+1. Child Ethnicity was also a categorical factor, effect coded with Hispanic/Non-Hispanic as ‑1/+1. Child Race was also a categorical factor, in which “White” served as the reference level. Age, Maximum Parental Education (i.e., highest education level between parents/caregivers), Area Deprivation Index (ADI), and Intracranial Volume were centered continuous factors. The random effects structure included a random intercept for magnetic resonance imaging (MRI) scanner serial number and family identification number. Random effects were restricted to be uncorrelated. Analysis included 8,524 data points. The model accounted for 60.3% of the variance in the data (*R*^2^ = .603, adjusted *R*^2^ = .602).

**Supplementary Table 39. Linear mixed-effects model output for the analysis of area deprivation index (ADI) and volume of the mid-posterior corpus callosum.**

|  | *t*(8508) | *p* | *b* | 95% CI |
| --- | --- | --- | --- | --- |
| Intercept | 70.54 | < .001 | 376.44 | [365.98, 386.90] |
| Maximum Parental Education | 0.71 | .476 | 0.69 | [-1.21, 2.60] |
| Family Income (High) | 0.23 | .821 | 0.41 | [-3.15, 3.97] |
| Family Income (Low) | -0.87 | .382 | -1.47 | [-4.76, 1.82] |
| Sex | 1.79 | .073 | 1.65 | [-0.15, 3.45] |
| Child Race (American Indian/Alaska Native) | 1.14 | .256 | 12.18 | [-8.85, 33.20] |
| Child Race (Asian) | -0.49 | .623 | -3.31 | [-16.48, 9.86] |
| Child Race (Black) | -1.42 | .157 | -7.59 | [-18.10, 2.91] |
| Child Race (Native Hawaiian / Pacific Islander) | -0.85 | .393 | -18.66 | [-61.51, 24.19] |
| Child Race (Other) | 1.49 | .135 | 7.74 | [-2.41, 17.89] |
| Child Ethnicity | 0.98 | .329 | 1.25 | [-1.26, 3.77] |
| Age | 7.57 | < .001 | 0.81 | [0.60, 1.02] |
| ADI | -0.80 | .422 | -0.37 | [-1.26, 0.53] |
| Intracranial Volume | 19.13 | < .001 | 0.0001 | [0.0001, 0.0001] |
| Family Income (High) × ADI | 1.87 | .061 | 1.07 | [-0.05, 2.18] |
| Family Income (Low) × ADI | -1.33 | .183 | -0.68 | [-1.68, 0.32] |

**Note**: The linear mixed-effects model incorporates testing the statistical significance of coefficients against a *t*-distribution. Family Income was a categorical, effects-coded factor, in which the level “Mid” served as the reference level. Family Income was operationally defined as the self-reported combined family income and partitioned into three levels: Low Income: ≤ $50K; Mid Income: $50K-$100K; High Income: ≥ $100K. Sex was also a categorical factor, effect coded with Male/Female as ‑1/+1. Child Ethnicity was also a categorical factor, effect coded with Hispanic/Non-Hispanic as ‑1/+1. Child Race was also a categorical factor, in which “White” served as the reference level. Age, Maximum Parental Education (i.e., highest education level between parents/caregivers), Area Deprivation Index (ADI), and Intracranial Volume were centered continuous factors. The random effects structure included a random intercept for magnetic resonance imaging (MRI) scanner serial number and family identification number. Random effects were restricted to be uncorrelated. Analysis included 8,524 data points. The model accounted for 43.0% of the variance in the data (*R*^2^ = .430, adjusted *R*^2^ = .429).

**Supplementary Table 40. Linear mixed-effects model output for the analysis of area deprivation index (ADI) and volume of the central corpus callosum.**

|  | *t*(8508) | *p* | *b* | 95% CI |
| --- | --- | --- | --- | --- |
| Intercept | 66.34 | < .001 | 405.75 | [393.76, 417.74] |
| Maximum Parental Education | -0.64 | .520 | -0.69 | [-2.81, 1.42] |
| Family Income (High) | -0.17 | .861 | -0.35 | [-4.30, 3.60] |
| Family Income (Low) | -0.70 | .485 | -1.30 | [-4.95, 2.35] |
| Sex | 3.33 | .001 | 3.41 | [1.40, 5.42] |
| Child Race (American Indian/Alaska Native) | 0.30 | .764 | 3.58 | [-19.74, 26.90] |
| Child Race (Asian) | 1.67 | .094 | 12.46 | [-2.14, 27.05] |
| Child Race (Black) | 0.67 | .503 | 3.97 | [-7.66, 15.60] |
| Child Race (Native Hawaiian / Pacific Islander) | -1.45 | .147 | -34.96 | [-82.25, 12.34] |
| Child Race (Other) | 1.77 | .076 | 10.16 | [-1.06, 21.38] |
| Child Ethnicity | 1.63 | .103 | 2.35 | [-0.48, 5.18] |
| Age | 5.00 | < .001 | 0.60 | [0.36, 0.83] |
| ADI | -0.81 | .417 | -0.42 | [-1.45, 0.60] |
| Intracranial Volume | 17.50 | < .001 | 0.0001 | [0.0001, 0.0002] |
| Family Income (High) × ADI | 1.53 | .127 | 0.96 | [-0.27, 2.20] |
| Family Income (Low) × ADI | -1.72 | .085 | -0.97 | [-2.08, 0.13] |

**Note**: The linear mixed-effects model incorporates testing the statistical significance of coefficients against a *t*-distribution. Family Income was a categorical, effects-coded factor, in which the level “Mid” served as the reference level. Family Income was operationally defined as the self-reported combined family income and partitioned into three levels: Low Income: ≤ $50K; Mid Income: $50K-$100K; High Income: ≥ $100K. Sex was also a categorical factor, effect coded with Male/Female as ‑1/+1. Child Ethnicity was also a categorical factor, effect coded with Hispanic/Non-Hispanic as ‑1/+1. Child Race was also a categorical factor, in which “White” served as the reference level. Age, Maximum Parental Education (i.e., highest education level between parents/caregivers), Area Deprivation Index (ADI), and Intracranial Volume were centered continuous factors. The random effects structure included a random intercept for magnetic resonance imaging (MRI) scanner serial number and family identification number. Random effects were restricted to be uncorrelated. Analysis included 8,524 data points. The model accounted for 36.8% of the variance in the data (*R*^2^ = .368, adjusted *R*^2^ = .367).

**Supplementary Table 41. Linear mixed-effects model output for the analysis of area deprivation index (ADI) and volume of the mid-anterior corpus callosum.**

|  | *t*(8508) | *p* | *b* | 95% CI |
| --- | --- | --- | --- | --- |
| Intercept | 63.00 | < .001 | 434.42 | [420.91, 447.94] |
| Maximum Parental Education | -2.21 | .027 | -2.70 | [-5.10, -0.31] |
| Family Income (High) | 0.03 | .980 | 0.06 | [-4.41, 4.53] |
| Family Income (Low) | -0.14 | .887 | -0.30 | [-4.44, 3.84] |
| Sex | -0.17 | .869 | -0.19 | [-2.47, 2.08] |
| Child Race (American Indian/Alaska Native) | 0.55 | .584 | 7.39 | [-19.03, 33.80] |
| Child Race (Asian) | 2.60 | .009 | 21.97 | [5.43, 38.50] |
| Child Race (Black) | -1.34 | .179 | -9.02 | [-22.19, 4.15] |
| Child Race (Native Hawaiian / Pacific Islander) | -1.37 | .170 | -37.49 | [-91.04, 16.06] |
| Child Race (Other) | 1.68 | .093 | 10.89 | [-1.82, 23.60] |
| Child Ethnicity | 1.00 | .319 | 1.63 | [-1.58, 4.83] |
| Age | 0.06 | .954 | 0.01 | [-0.26, 0.27] |
| ADI | -0.80 | .421 | -0.48 | [-1.63, 0.68] |
| Intracranial Volume | 23.60 | < .001 | 0.0002 | [0.0002, 0.0002] |
| Family Income (High) × ADI | 1.92 | .055 | 1.37 | [-0.03, 2.77] |
| Family Income (Low) × ADI | -1.40 | .163 | -0.89 | [-2.15, 0.36] |

**Note**: The linear mixed-effects model incorporates testing the statistical significance of coefficients against a *t*-distribution. Family Income was a categorical, effects-coded factor, in which the level “Mid” served as the reference level. Family Income was operationally defined as the self-reported combined family income and partitioned into three levels: Low Income: ≤ $50K; Mid Income: $50K-$100K; High Income: ≥ $100K. Sex was also a categorical factor, effect coded with Male/Female as ‑1/+1. Child Ethnicity was also a categorical factor, effect coded with Hispanic/Non-Hispanic as ‑1/+1. Child Race was also a categorical factor, in which “White” served as the reference level. Age, Maximum Parental Education (i.e., highest education level between parents/caregivers), Area Deprivation Index (ADI), and Intracranial Volume were centered continuous factors. The random effects structure included a random intercept for magnetic resonance imaging (MRI) scanner serial number and family identification number. Random effects were restricted to be uncorrelated. Analysis included 8,524 data points. The model accounted for 39.8% of the variance in the data (*R*^2^ = .398, adjusted *R*^2^ = .397).

**Supplementary Table 42. Linear mixed-effects model output for the analysis of area deprivation index (ADI) and volume of anterior corpus callosum.**

|  | *t*(8508) | *p* | *b* | 95% CI |
| --- | --- | --- | --- | --- |
| Intercept | 79.32 | < .001 | 777.82 | [758.60, 797.04] |
| Maximum Parental Education | -0.22 | .829 | -0.34 | [-3.46, 2.77] |
| Family Income (High) | -0.54 | .592 | -1.59 | [-7.40, 4.23] |
| Family Income (Low) | -0.39 | .697 | -1.07 | [-6.44, 4.31] |
| Sex | 4.24 | < .001 | 6.33 | [3.40, 9.25] |
| Child Race (American Indian/Alaska Native) | -0.76 | .447 | -13.28 | [-47.48, 20.92] |
| Child Race (Asian) | 0.36 | .716 | 4.00 | [-17.52, 25.53] |
| Child Race (Black) | 1.37 | .172 | 12.02 | [-5.22, 29.26] |
| Child Race (Native Hawaiian / Pacific Islander) | -1.12 | .264 | -40.14 | [-110.59, 30.32] |
| Child Race (Other) | 2.33 | .020 | 19.80 | [3.16, 36.43] |
| Child Ethnicity | 1.22 | .223 | 2.61 | [-1.59, 6.81] |
| Age | -0.21 | .835 | -0.04 | [-0.37, 0.30] |
| ADI | 1.20 | .231 | 0.94 | [-0.60, 2.49] |
| Intracranial Volume | 37.20 | < .001 | 0.0004 | [0.0004, 0.0004] |
| Family Income (High) × ADI | -0.35 | .724 | -0.33 | [-2.15, 1.49] |
| Family Income (Low) × ADI | -0.14 | .892 | -0.11 | [-1.74, 1.52] |

**Note**: The linear mixed-effects model incorporates testing the statistical significance of coefficients against a *t*-distribution. Family Income was a categorical, effects-coded factor, in which the level “Mid” served as the reference level. Family Income was operationally defined as the self-reported combined family income and partitioned into three levels: Low Income: ≤ $50K; Mid Income: $50K-$100K; High Income: ≥ $100K. Sex was also a categorical factor, effect coded with Male/Female as ‑1/+1. Child Ethnicity was also a categorical factor, effect coded with Hispanic/Non-Hispanic as ‑1/+1. Child Race was also a categorical factor, in which “White” served as the reference level. Age, Maximum Parental Education (i.e., highest education level between parents/caregivers), Area Deprivation Index (ADI), and Intracranial Volume were centered continuous factors. The random effects structure included a random intercept for magnetic resonance imaging (MRI) scanner serial number and family identification number. Random effects were restricted to be uncorrelated. Analysis included 8,524 data points. The model accounted for 63.4% of the variance in the data (*R*^2^ = .634, adjusted *R*^2^ = .634).

**Supplementary Table 43. Linear mixed-effects model output for the analysis of area deprivation index (ADI) and volume of the lateral ventricles, collapsed across hemispheres.**

|  | *t*(8508) | *p* | *b* | 95% CI |
| --- | --- | --- | --- | --- |
| Intercept | 23.56 | < .001 | 4656.11 | [4268.63, 5043.59] |
| Maximum Parental Education | -0.70 | .483 | -22.17 | [-84.09, 39.75] |
| Family Income (High) | -0.77 | .440 | -45.46 | [-160.91, 69.99] |
| Family Income (Low) | 0.76 | .449 | 41.29 | [-65.55, 148.13] |
| Sex | 1.69 | .092 | 50.68 | [-8.26, 109.62] |
| Child Race (American Indian/Alaska Native) | -0.44 | .661 | -152.57 | [-834.50, 529.35] |
| Child Race (Asian) | -0.62 | .538 | -133.90 | [-560.59, 292.79] |
| Child Race (Black) | 1.63 | .102 | 283.39 | [-56.43, 623.21] |
| Child Race (Native Hawaiian / Pacific Islander) | 0.50 | .617 | 351.46 | [-1027.67, 1730.59] |
| Child Race (Other) | -0.85 | .398 | -141.22 | [-468.74, 186.30] |
| Child Ethnicity | 0.34 | .735 | 14.43 | [-69.29, 98.16] |
| Age | 2.40 | .016 | 8.41 | [1.55, 15.27] |
| ADI | -0.45 | .652 | -7.09 | [-37.90, 23.72] |
| Intracranial Volume | 30.95 | < .001 | 0.01 | [0.01, 0.01] |
| Family Income (High) × ADI | -1.08 | .279 | -19.97 | [-56.15, 16.21] |
| Family Income (Low) × ADI | 0.93 | .350 | 15.46 | [-16.96, 47.89] |

**Note**: The linear mixed-effects model incorporates testing the statistical significance of coefficients against a *t*-distribution. Family Income was a categorical, effects-coded factor, in which the level “Mid” served as the reference level. Family Income was operationally defined as the self-reported combined family income and partitioned into three levels: Low Income: ≤ $50K; Mid Income: $50K-$100K; High Income: ≥ $100K. Sex was also a categorical factor, effect coded with Male/Female as ‑1/+1. Child Ethnicity was also a categorical factor, effect coded with Hispanic/Non-Hispanic as ‑1/+1. Child Race was also a categorical factor, in which “White” served as the reference level. Age, Maximum Parental Education (i.e., highest education level between parents/caregivers), Area Deprivation Index (ADI), and Intracranial Volume were centered continuous factors. The random effects structure included a random intercept for magnetic resonance imaging (MRI) scanner serial number and family identification number. Random effects were restricted to be uncorrelated. Analysis included 8,524 data points. The model accounted for 40.2% of the variance in the data (*R*^2^ = .402, adjusted *R*^2^ = .401).

**Supplementary Table 44. Linear mixed-effects model output for the analysis of area deprivation index (ADI) and volume of the inferior lateral ventricles, collapsed across hemispheres.**

|  | *t*(8508) | *p* | *b* | 95% CI |
| --- | --- | --- | --- | --- |
| Intercept | 24.68 | < .001 | 309.54 | [284.95, 334.13] |
| Maximum Parental Education | -1.45 | .148 | -2.38 | [-5.60, 0.85] |
| Family Income (High) | 0.68 | .499 | 2.07 | [-3.93, 8.08] |
| Family Income (Low) | 0.21 | .832 | 0.60 | [-4.96, 6.16] |
| Sex | -4.08 | < .001 | -6.42 | [-9.50, -3.34] |
| Child Race (American Indian/Alaska Native) | -0.30 | .764 | -5.43 | [-40.95, 30.09] |
| Child Race (Asian) | 0.87 | .382 | 9.89 | [-12.28, 32.07] |
| Child Race (Black) | -1.63 | .104 | -14.62 | [-32.23, 2.99] |
| Child Race (Native Hawaiian / Pacific Islander) | 0.89 | .375 | 32.23 | [-39.00, 103.47] |
| Child Race (Other) | -1.30 | .195 | -11.21 | [-28.17, 5.76] |
| Child Ethnicity | -2.52 | .012 | -5.64 | [-10.02, -1.26] |
| Age | 1.56 | .118 | 0.29 | [-0.07, 0.65] |
| ADI | -0.18 | .860 | -0.15 | [-1.77, 1.48] |
| Intracranial Volume | 12.93 | < .001 | 0.0002 | [0.0001, 0.0002] |
| Family Income (High) × ADI | 0.07 | .942 | 0.07 | [-1.81, 1.95] |
| Family Income (Low) × ADI | 0.01 | .992 | 0.01 | [-1.68, 1.70] |

**Note**: The linear mixed-effects model incorporates testing the statistical significance of coefficients against a *t*-distribution. Family Income was a categorical, effects-coded factor, in which the level “Mid” served as the reference level. Family Income was operationally defined as the self-reported combined family income and partitioned into three levels: Low Income: ≤ $50K; Mid Income: $50K-$100K; High Income: ≥ $100K. Sex was also a categorical factor, effect coded with Male/Female as ‑1/+1. Child Ethnicity was also a categorical factor, effect coded with Hispanic/Non-Hispanic as ‑1/+1. Child Race was also a categorical factor, in which “White” served as the reference level. Age, Maximum Parental Education (i.e., highest education level between parents/caregivers), Area Deprivation Index (ADI), and Intracranial Volume were centered continuous factors. The random effects structure included a random intercept for magnetic resonance imaging (MRI) scanner serial number and family identification number. Random effects were restricted to be uncorrelated. Analysis included 8,524 data points. The model accounted for 30.1% of the variance in the data (*R*^2^ = .301, adjusted *R*^2^ = .300).

**Supplementary Table 45. Linear mixed-effects model output for the analysis of area deprivation index (ADI) and volume of the 3^rd^ ventricle.**

|  | *t*(8508) | *p* | *b* | 95% CI |
| --- | --- | --- | --- | --- |
| Intercept | 47.34 | < .001 | 756.61 | [725.28, 787.94] |
| Maximum Parental Education | -1.15 | .250 | -3.01 | [-8.12, 2.11] |
| Family Income (High) | -0.43 | .668 | -2.09 | [-11.64, 7.46] |
| Family Income (Low) | 2.24 | .025 | 10.10 | [1.27, 18.94] |
| Sex | 0.23 | .815 | 0.58 | [-4.27, 5.43] |
| Child Race (American Indian/Alaska Native) | 0.12 | .906 | 3.40 | [-52.94, 59.73] |
| Child Race (Asian) | 1.77 | .077 | 31.83 | [-3.49, 67.15] |
| Child Race (Black) | 1.27 | .204 | 18.28 | [-9.91, 46.46] |
| Child Race (Native Hawaiian / Pacific Islander) | -0.35 | .726 | -20.52 | [-135.20, 94.16] |
| Child Race (Other) | -0.90 | .368 | -12.49 | [-39.67, 14.68] |
| Child Ethnicity | -0.33 | .741 | -1.17 | [-8.08, 5.74] |
| Age | 0.30 | .766 | 0.09 | [-0.48, 0.65] |
| ADI | -0.34 | .733 | -0.44 | [-2.98, 2.10] |
| Intracranial Volume | 31.93 | < .001 | 0.001 | [0.001, 0.001] |
| Family Income (High) × ADI | -0.08 | .934 | -0.13 | [-3.12, 2.86] |
| Family Income (Low) × ADI | -0.83 | .408 | -1.13 | [-3.81, 1.55] |

**Note**: The linear mixed-effects model incorporates testing the statistical significance of coefficients against a *t*-distribution. Family Income was a categorical, effects-coded factor, in which the level “Mid” served as the reference level. Family Income was operationally defined as the self-reported combined family income and partitioned into three levels: Low Income: ≤ $50K; Mid Income: $50K-$100K; High Income: ≥ $100K. Sex was also a categorical factor, effect coded with Male/Female as ‑1/+1. Child Ethnicity was also a categorical factor, effect coded with Hispanic/Non-Hispanic as ‑1/+1. Child Race was also a categorical factor, in which “White” served as the reference level. Age, Maximum Parental Education (i.e., highest education level between parents/caregivers), Area Deprivation Index (ADI), and Intracranial Volume were centered continuous factors. The random effects structure included a random intercept for magnetic resonance imaging (MRI) scanner serial number and family identification number. Random effects were restricted to be uncorrelated. Analysis included 8,524 data points. The model accounted for 47.3% of the variance in the data (*R*^2^ = .473, adjusted *R*^2^ = .472).

**Supplementary Table 46. Linear mixed-effects model output for the analysis of area deprivation index (ADI) and volume of the 4^th^ ventricle.**

|  | *t*(8508) | *p* | *b* | 95% CI |
| --- | --- | --- | --- | --- |
| Intercept | 46.51 | < .001 | 1787.37 | [1712.03, 1862.71] |
| Maximum Parental Education | 3.43 | .001 | 23.87 | [10.25, 37.50] |
| Family Income (High) | 0.40 | .688 | 5.23 | [-20.26, 30.71] |
| Family Income (Low) | 0.67 | .500 | 8.11 | [-15.46, 31.68] |
| Sex | -8.49 | < .001 | -55.58 | [-68.41, -42.75] |
| Child Race (American Indian/Alaska Native) | -0.67 | .506 | -50.99 | [-201.24, 99.26] |
| Child Race (Asian) | 0.43 | .666 | 20.76 | [-73.60, 115.13] |
| Child Race (Black) | 0.50 | .620 | 19.10 | [-56.36, 94.55] |
| Child Race (Native Hawaiian / Pacific Islander) | 0.97 | .334 | 151.95 | [-156.60, 460.50] |
| Child Race (Other) | -1.44 | .149 | -53.73 | [-126.67, 19.20] |
| Child Ethnicity | -0.65 | .518 | -5.94 | [-23.96, 12.07] |
| Age | 1.62 | .106 | 1.22 | [-0.26, 2.71] |
| ADI | -2.17 | .030 | -7.13 | [-13.56, -0.70] |
| Intracranial Volume | 17.73 | < .001 | 0.001 | [0.001, 0.001] |
| Family Income (High) × ADI | 0.13 | .895 | 0.54 | [-7.44, 8.51] |
| Family Income (Low) × ADI | -1.08 | .279 | -3.95 | [-11.09, 3.20] |

**Note**: The linear mixed-effects model incorporates testing the statistical significance of coefficients against a *t*-distribution. Family Income was a categorical, effects-coded factor, in which the level “Mid” served as the reference level. Family Income was operationally defined as the self-reported combined family income and partitioned into three levels: Low Income: ≤ $50K; Mid Income: $50K-$100K; High Income: ≥ $100K. Sex was also a categorical factor, effect coded with Male/Female as ‑1/+1. Child Ethnicity was also a categorical factor, effect coded with Hispanic/Non-Hispanic as ‑1/+1. Child Race was also a categorical factor, in which “White” served as the reference level. Age, Maximum Parental Education (i.e., highest education level between parents/caregivers), Area Deprivation Index (ADI), and Intracranial Volume were centered continuous factors. The random effects structure included a random intercept for magnetic resonance imaging (MRI) scanner serial number and family identification number. Random effects were restricted to be uncorrelated. Analysis included 8,524 data points. The model accounted for 50.3% of the variance in the data (*R*^2^ = .503, adjusted *R*^2^ = .502).

**Supplementary Table 47. Linear mixed-effects model output for the analysis of area deprivation index (ADI) and subcortical gray matter volume.**

|  | *t*(8508) | *p* | *b* | 95% CI |
| --- | --- | --- | --- | --- |
| Intercept | 224.91 | < .001 | 60387.11 | [59860.79, 60913.44] |
| Maximum Parental Education | 1.42 | .155 | 50.54 | [-19.15, 120.22] |
| Family Income (High) | -0.02 | .982 | -1.53 | [-131.62, 128.56] |
| Family Income (Low) | -1.86 | .063 | -114.07 | [-234.34, 6.20] |
| Sex | -6.55 | < .001 | -217.80 | [-283.03, -152.57] |
| Child Race (American Indian/Alaska Native) | -0.85 | .395 | -331.18 | [-1095.10, 432.74] |
| Child Race (Asian) | 0.22 | .822 | 55.19 | [-426.29, 536.66] |
| Child Race (Black) | -0.79 | .430 | -155.59 | [-541.68, 230.51] |
| Child Race (Native Hawaiian / Pacific Islander) | 0.42 | .673 | 339.96 | [-1238.34, 1918.26] |
| Child Race (Other) | 0.20 | .843 | 37.60 | [-334.72, 409.92] |
| Child Ethnicity | -0.90 | .369 | -43.35 | [-137.85, 51.15] |
| Age | 0.17 | .863 | 0.66 | [-6.81, 8.12] |
| ADI | -3.39 | .001 | -60.70 | [-95.81, -25.59] |
| Intracranial Volume | 113.71 | < .001 | 0.03 | [0.03, 0.03] |
| Family Income (High) × ADI | -1.13 | .260 | -23.42 | [-64.14, 17.31] |
| Family Income (Low) × ADI | -0.21 | .833 | -3.93 | [-40.38, 32.52] |

**Note**: The linear mixed-effects model incorporates testing the statistical significance of coefficients against a *t*-distribution. Family Income was a categorical, effects-coded factor, in which the level “Mid” served as the reference level. Family Income was operationally defined as the self-reported combined family income and partitioned into three levels: Low Income: ≤ $50K; Mid Income: $50K-$100K; High Income: ≥ $100K. Sex was also a categorical factor, effect coded with Male/Female as ‑1/+1. Child Ethnicity was also a categorical factor, effect coded with Hispanic/Non-Hispanic as ‑1/+1. Child Race was also a categorical factor, in which “White” served as the reference level. Age, Maximum Parental Education (i.e., highest education level between parents/caregivers), Area Deprivation Index (ADI), and Intracranial Volume were centered continuous factors. The random effects structure included a random intercept for magnetic resonance imaging (MRI) scanner serial number and family identification number. Random effects were restricted to be uncorrelated. Analysis included 8,524 data points. The model accounted for 91.3% of the variance in the data (*R*^2^ = .913, adjusted *R*^2^ = .912).

**Supplementary Table 48. Linear mixed-effects model output for the analysis of the age-of-housing metric and volume of the thalamus proper, collapsed across hemispheres.**

|  | *t*(8508) | *p* | *b* | 95% CI |
| --- | --- | --- | --- | --- |
| Intercept | 229.73 | < .001 | 7529.31 | [7465.06, 7593.55] |
| Maximum Parental Education | 0.21 | .832 | 1.18 | [-9.70, 12.05] |
| Family Income (High) | 0.35 | .726 | 2.87 | [-13.21, 18.95] |
| Family Income (Low) | -1.10 | .273 | -10.21 | [-28.49, 8.06] |
| Sex | -3.97 | < .001 | -20.97 | [-31.33, -10.61] |
| Child Race (American Indian/Alaska Native) | -0.35 | .724 | -21.82 | [-142.85, 99.20] |
| Child Race (Asian) | -0.30 | .765 | -11.61 | [-87.61, 64.39] |
| Child Race (Black) | -0.55 | .582 | -17.04 | [-77.75, 43.67] |
| Child Race (Native Hawaiian / Pacific Islander) | 0.48 | .632 | 60.78 | [-187.80, 309.37] |
| Child Race (Other) | 0.42 | .677 | 12.49 | [-46.26, 71.23] |
| Child Ethnicity | -0.96 | .336 | -7.24 | [-22.00, 7.51] |
| Age | 6.32 | < .001 | 3.85 | [2.65, 5.04] |
| Age of Housing | 0.06 | .950 | 0.18 | [-5.50, 5.86] |
| Intracranial Volume | 93.81 | < .001 | 0.004 | [0.004, 0.004] |
| Family Income (High) × Age of Housing | 0.48 | .633 | 1.58 | [-4.90, 8.06] |
| Family Income (Low) × Age of Housing | -0.43 | .671 | -1.57 | [-8.79, 5.66] |

**Note**: The linear mixed-effects model incorporates testing the statistical significance of coefficients against a *t*-distribution. Family Income was a categorical, effects-coded factor, in which the level “Mid” served as the reference level. Family Income was operationally defined as the self-reported combined family income and partitioned into three levels: Low Income: ≤ $50K; Mid Income: $50K-$100K; High Income: ≥ $100K. Sex was also a categorical factor, effect coded with Male/Female as ‑1/+1. Child Ethnicity was also a categorical factor, effect coded with Hispanic/Non-Hispanic as ‑1/+1. Child Race was also a categorical factor, in which “White” served as the reference level. Age, Maximum Parental Education (i.e., highest education level between parents/caregivers), Age of Housing, and Intracranial Volume were centered continuous factors. The random effects structure included a random intercept for magnetic resonance imaging (MRI) scanner serial number and family identification number. Random effects were restricted to be uncorrelated. Analysis included 8,524 data points. The model accounted for 85.9% of the variance in the data (*R*^2^ = .859, adjusted *R*^2^ = .859).

**Supplementary Table 49. Linear mixed-effects model output for the analysis of the age-of-housing metric and volume of the caudate, collapsed across hemispheres.**

|  | *t*(8508) | *p* | *b* | 95% CI |
| --- | --- | --- | --- | --- |
| Intercept | 94.82 | < .001 | 4025.29 | [3942.07, 4108.50] |
| Maximum Parental Education | 2.36 | .018 | 12.90 | [2.21, 23.60] |
| Family Income (High) | -0.31 | .759 | -2.48 | [-18.33, 13.37] |
| Family Income (Low) | -1.07 | .284 | -9.84 | [-27.84, 8.17] |
| Sex | 7.64 | < .001 | 39.50 | [29.36, 49.64] |
| Child Race (American Indian/Alaska Native) | -2.40 | .016 | -145.56 | [-264.37, -26.75] |
| Child Race (Asian) | -0.25 | .803 | -9.52 | [-84.38, 65.34] |
| Child Race (Black) | 1.29 | .196 | 39.59 | [-20.36, 99.54] |
| Child Race (Native Hawaiian / Pacific Islander) | 0.04 | .972 | 4.46 | [-241.29, 250.21] |
| Child Race (Other) | 1.54 | .124 | 45.45 | [-12.49, 103.40] |
| Child Ethnicity | 1.39 | .164 | 10.43 | [-4.24, 25.10] |
| Age | -3.48 | .001 | -2.06 | [-3.22, -0.90] |
| Age of Housing | -0.57 | .570 | -1.65 | [-7.35, 4.05] |
| Intracranial Volume | 58.56 | < .001 | 0.002 | [0.002, 0.002] |
| Family Income (High) × Age of Housing | 0.18 | .856 | 0.59 | [-5.80, 6.98] |
| Family Income (Low) × Age of Housing | 0.82 | .413 | 2.97 | [-4.15, 10.09] |

**Note**: The linear mixed-effects model incorporates testing the statistical significance of coefficients against a *t*-distribution. Family Income was a categorical, effects-coded factor, in which the level “Mid” served as the reference level. Family Income was operationally defined as the self-reported combined family income and partitioned into three levels: Low Income: ≤ $50K; Mid Income: $50K-$100K; High Income: ≥ $100K. Sex was also a categorical factor, effect coded with Male/Female as ‑1/+1. Child Ethnicity was also a categorical factor, effect coded with Hispanic/Non-Hispanic as ‑1/+1. Child Race was also a categorical factor, in which “White” served as the reference level. Age, Maximum Parental Education (i.e., highest education level between parents/caregivers), Age of Housing, and Intracranial Volume were centered continuous factors. The random effects structure included a random intercept for magnetic resonance imaging (MRI) scanner serial number and family identification number. Random effects were restricted to be uncorrelated. Analysis included 8,524 data points. The model accounted for 76.4% of the variance in the data (*R*^2^ = .764, adjusted *R*^2^ = .763).

**Supplementary Table 50. Linear mixed-effects model output for the analysis of the age-of-housing metric and volume of the putamen, collapsed across hemispheres.**

|  | *t*(8508) | *p* | *b* | 95% CI |
| --- | --- | --- | --- | --- |
| Intercept | 148.31 | < .001 | 5893.34 | [5815.44, 5971.23] |
| Maximum Parental Education | 0.23 | .818 | 1.54 | [-11.59, 14.67] |
| Family Income (High) | 1.17 | .242 | 11.60 | [-7.82, 31.02] |
| Family Income (Low) | -2.49 | .013 | -28.05 | [-50.11, -5.98] |
| Sex | -9.61 | < .001 | -61.08 | [-73.54, -48.62] |
| Child Race (American Indian/Alaska Native) | -1.57 | .115 | -117.25 | [-263.21, 28.72] |
| Child Race (Asian) | 3.29 | .001 | 154.09 | [62.28, 245.89] |
| Child Race (Black) | -1.86 | .063 | -69.55 | [-142.99, 3.89] |
| Child Race (Native Hawaiian / Pacific Islander) | 0.24 | .810 | 37.01 | [-264.13, 338.15] |
| Child Race (Other) | 0.01 | .993 | 0.33 | [-70.74, 71.39] |
| Child Ethnicity | -1.28 | .199 | -11.67 | [-29.48, 6.14] |
| Age | -3.86 | < .001 | -2.82 | [-4.25, -1.39] |
| Age of Housing | -1.35 | .177 | -4.73 | [-11.60, 2.14] |
| Intracranial Volume | 51.08 | < .001 | 0.002 | [0.002, 0.003] |
| Family Income (High) × Age of Housing | 0.18 | .855 | 0.73 | [-7.09, 8.55] |
| Family Income (Low) × Age of Housing | 0.61 | .539 | 2.73 | [-5.99, 11.45] |

**Note**: The linear mixed-effects model incorporates testing the statistical significance of coefficients against a *t*-distribution. Family Income was a categorical, effects-coded factor, in which the level “Mid” served as the reference level. Family Income was operationally defined as the self-reported combined family income and partitioned into three levels: Low Income: ≤ $50K; Mid Income: $50K-$100K; High Income: ≥ $100K. Sex was also a categorical factor, effect coded with Male/Female as ‑1/+1. Child Ethnicity was also a categorical factor, effect coded with Hispanic/Non-Hispanic as ‑1/+1. Child Race was also a categorical factor, in which “White” served as the reference level. Age, Maximum Parental Education (i.e., highest education level between parents/caregivers), Age of Housing, and Intracranial Volume were centered continuous factors. The random effects structure included a random intercept for magnetic resonance imaging (MRI) scanner serial number and family identification number. Random effects were restricted to be uncorrelated. Analysis included 8,524 data points. The model accounted for 75.3% of the variance in the data (*R*^2^ = .753, adjusted *R*^2^ = .753).

**Supplementary Table 51. Linear mixed-effects model output for the analysis of the age-of-housing metric and volume of the pallidum, collapsed across hemispheres.**

|  | *t*(8508) | *p* | *b* | 95% CI |
| --- | --- | --- | --- | --- |
| Intercept | 111.34 | < .001 | 1784.84 | [1753.42, 1816.27] |
| Maximum Parental Education | 1.72 | .086 | 3.46 | [-0.49, 7.42] |
| Family Income (High) | 1.59 | .112 | 4.74 | [-1.11, 10.60] |
| Family Income (Low) | -1.30 | .194 | -4.41 | [-11.06, 2.25] |
| Sex | -10.66 | < .001 | -20.69 | [-24.50, -16.89] |
| Child Race (American Indian/Alaska Native) | 0.05 | .963 | 1.06 | [-43.02, 45.13] |
| Child Race (Asian) | 0.23 | .818 | 3.24 | [-24.36, 30.84] |
| Child Race (Black) | 0.70 | .483 | 7.85 | [-14.11, 29.82] |
| Child Race (Native Hawaiian / Pacific Islander) | 0.05 | .962 | 2.17 | [-87.27, 91.61] |
| Child Race (Other) | -0.31 | .757 | -3.35 | [-24.57, 17.86] |
| Child Ethnicity | -1.69 | .091 | -4.68 | [-10.11, 0.75] |
| Age | -7.47 | < .001 | -1.68 | [-2.13, -1.24] |
| Age of Housing | -0.58 | .565 | -0.62 | [-2.72, 1.49] |
| Intracranial Volume | 53.13 | < .001 | 0.001 | [0.001, 0.001] |
| Family Income (High) × Age of Housing | 1.07 | .284 | 1.29 | [-1.07, 3.65] |
| Family Income (Low) × Age of Housing | 0.55 | .580 | 0.74 | [-1.89, 3.38] |

**Note**: The linear mixed-effects model incorporates testing the statistical significance of coefficients against a *t*-distribution. Family Income was a categorical, effects-coded factor, in which the level “Mid” served as the reference level. Family Income was operationally defined as the self-reported combined family income and partitioned into three levels: Low Income: ≤ $50K; Mid Income: $50K-$100K; High Income: ≥ $100K. Sex was also a categorical factor, effect coded with Male/Female as ‑1/+1. Child Ethnicity was also a categorical factor, effect coded with Hispanic/Non-Hispanic as ‑1/+1. Child Race was also a categorical factor, in which “White” served as the reference level. Age, Maximum Parental Education (i.e., highest education level between parents/caregivers), Age of Housing, and Intracranial Volume were centered continuous factors. The random effects structure included a random intercept for magnetic resonance imaging (MRI) scanner serial number and family identification number. Random effects were restricted to be uncorrelated. Analysis included 8,524 data points. The model accounted for 64.7% of the variance in the data (*R*^2^ = .647, adjusted *R*^2^ = .647).

**Supplementary Table 52. Linear mixed-effects model output for the analysis of the age-of-housing metric and volume of the hippocampus, collapsed across hemispheres.**

|  | *t*(8508) | *p* | *b* | 95% CI |
| --- | --- | --- | --- | --- |
| Intercept | 188.48 | < .001 | 4089.62 | [4047.08, 4132.15] |
| Maximum Parental Education | 2.59 | .010 | 10.19 | [2.48, 17.90] |
| Family Income (High) | 3.00 | .003 | 17.39 | [6.03, 28.76] |
| Family Income (Low) | -2.46 | .014 | -16.20 | [-29.13, -3.27] |
| Sex | -3.36 | .001 | -12.54 | [-19.85, -5.23] |
| Child Race (American Indian/Alaska Native) | 0.68 | .498 | 29.69 | [-56.18, 115.56] |
| Child Race (Asian) | -0.27 | .789 | -7.34 | [-61.22, 46.54] |
| Child Race (Black) | -4.79 | < .001 | -105.23 | [-148.28, -62.18] |
| Child Race (Native Hawaiian / Pacific Islander) | 1.45 | .148 | 130.68 | [-46.31, 307.66] |
| Child Race (Other) | -1.66 | .097 | -35.41 | [-77.20, 6.38] |
| Child Ethnicity | -1.12 | .264 | -5.81 | [-16.00, 4.38] |
| Age | 1.91 | .056 | 0.82 | [-0.02, 1.67] |
| Age of Housing | 1.65 | .100 | 3.25 | [-0.62, 7.12] |
| Intracranial Volume | 62.50 | < .001 | 0.002 | [0.002, 0.002] |
| Family Income (High) × Age of Housing | 0.14 | .887 | 0.33 | [-4.25, 4.91] |
| Family Income (Low) × Age of Housing | 0.81 | .416 | 2.11 | [-2.98, 7.21] |

**Note**: The linear mixed-effects model incorporates testing the statistical significance of coefficients against a *t*-distribution. Family Income was a categorical, effects-coded factor, in which the level “Mid” served as the reference level. Family Income was operationally defined as the self-reported combined family income and partitioned into three levels: Low Income: ≤ $50K; Mid Income: $50K-$100K; High Income: ≥ $100K. Sex was also a categorical factor, effect coded with Male/Female as ‑1/+1. Child Ethnicity was also a categorical factor, effect coded with Hispanic/Non-Hispanic as ‑1/+1. Child Race was also a categorical factor, in which “White” served as the reference level. Age, Maximum Parental Education (i.e., highest education level between parents/caregivers), Age of Housing, and Intracranial Volume were centered continuous factors. The random effects structure included a random intercept for magnetic resonance imaging (MRI) scanner serial number and family identification number. Random effects were restricted to be uncorrelated. Analysis included 8,524 data points. The model accounted for 78.4% of the variance in the data (*R*^2^ = .784, adjusted *R*^2^ = .784).

**Supplementary Table 53. Linear mixed-effects model output for the analysis of the age-of-housing metric and volume of the amygdala, collapsed across hemispheres.**

|  | *t*(8508) | *p* | *b* | 95% CI |
| --- | --- | --- | --- | --- |
| Intercept | 101.04 | < .001 | 1590.11 | [1559.26, 1620.95] |
| Maximum Parental Education | 1.47 | .142 | 2.87 | [-0.96, 6.69] |
| Family Income (High) | 2.25 | .024 | 6.52 | [0.84, 12.19] |
| Family Income (Low) | -1.77 | .077 | -5.82 | [-12.26, 0.62] |
| Sex | -12.71 | < .001 | -23.91 | [-27.60, -20.23] |
| Child Race (American Indian/Alaska Native) | 1.91 | .057 | 41.49 | [-1.19, 84.17] |
| Child Race (Asian) | 1.10 | .271 | 14.99 | [-11.72, 41.70] |
| Child Race (Black) | -3.65 | < .001 | -39.59 | [-60.84, -18.34] |
| Child Race (Native Hawaiian / Pacific Islander) | 0.08 | .934 | 3.66 | [-82.84, 90.15] |
| Child Race (Other) | -1.58 | .115 | -16.50 | [-37.03, 4.02] |
| Child Ethnicity | -1.09 | .275 | -2.93 | [-8.19, 2.33] |
| Age | 1.34 | .180 | 0.29 | [-0.14, 0.72] |
| Age of Housing | 0.47 | .642 | 0.48 | [-1.55, 2.52] |
| Intracranial Volume | 54.99 | < .001 | 0.001 | [0.001, 0.001] |
| Family Income (High) × Age of Housing | 0.12 | .902 | 0.14 | [-2.14, 2.43] |
| Family Income (Low) × Age of Housing | 0.75 | .453 | 0.98 | [-1.57, 3.53] |

**Note**: The linear mixed-effects model incorporates testing the statistical significance of coefficients against a *t*-distribution. Family Income was a categorical, effects-coded factor, in which the level “Mid” served as the reference level. Family Income was operationally defined as the self-reported combined family income and partitioned into three levels: Low Income: ≤ $50K; Mid Income: $50K-$100K; High Income: ≥ $100K. Sex was also a categorical factor, effect coded with Male/Female as ‑1/+1. Child Ethnicity was also a categorical factor, effect coded with Hispanic/Non-Hispanic as ‑1/+1. Child Race was also a categorical factor, in which “White” served as the reference level. Age, Maximum Parental Education (i.e., highest education level between parents/caregivers), Age of Housing, and Intracranial Volume were centered continuous factors. The random effects structure included a random intercept for magnetic resonance imaging (MRI) scanner serial number and family identification number. Random effects were restricted to be uncorrelated. Analysis included 8,524 data points. The model accounted for 73.0% of the variance in the data (*R*^2^ = .730, adjusted *R*^2^ = .730).

**Supplementary Table 54. Linear mixed-effects model output for the analysis of the age-of-housing metric and volume of the accumbens area, collapsed across hemispheres.**

|  | *t*(8508) | *p* | *b* | 95% CI |
| --- | --- | --- | --- | --- |
| Intercept | 56.64 | < .001 | 597.37 | [576.70, 618.04] |
| Maximum Parental Education | 0.19 | .852 | 0.18 | [-1.70, 2.06] |
| Family Income (High) | 0.81 | .416 | 1.16 | [-1.63, 3.94] |
| Family Income (Low) | -1.32 | .187 | -2.13 | [-5.30, 1.04] |
| Sex | -0.38 | .702 | -0.35 | [-2.16, 1.46] |
| Child Race (American Indian/Alaska Native) | 0.96 | .339 | 10.23 | [-10.73, 31.20] |
| Child Race (Asian) | -1.90 | .057 | -12.73 | [-25.86, 0.40] |
| Child Race (Black) | -0.13 | .897 | -0.69 | [-11.14, 9.76] |
| Child Race (Native Hawaiian / Pacific Islander) | 0.61 | .543 | 13.20 | [-29.36, 55.76] |
| Child Race (Other) | -1.39 | .165 | -7.14 | [-17.24, 2.95] |
| Child Ethnicity | -0.09 | .932 | -0.11 | [-2.70, 2.48] |
| Age | -6.34 | < .001 | -0.68 | [-0.89, -0.47] |
| Age of Housing | -0.43 | .667 | -0.22 | [-1.22, 0.78] |
| Intracranial Volume | 53.69 | < .001 | 0.0004 | [0.0004, 0.0004] |
| Family Income (High) × Age of Housing | 0.16 | .871 | 0.09 | [-1.03, 1.22] |
| Family Income (Low) × Age of Housing | 1.09 | .274 | 0.70 | [-0.55, 1.95] |

**Note**: The linear mixed-effects model incorporates testing the statistical significance of coefficients against a *t*-distribution. Family Income was a categorical, effects-coded factor, in which the level “Mid” served as the reference level. Family Income was operationally defined as the self-reported combined family income and partitioned into three levels: Low Income: ≤ $50K; Mid Income: $50K-$100K; High Income: ≥ $100K. Sex was also a categorical factor, effect coded with Male/Female as ‑1/+1. Child Ethnicity was also a categorical factor, effect coded with Hispanic/Non-Hispanic as ‑1/+1. Child Race was also a categorical factor, in which “White” served as the reference level. Age, Maximum Parental Education (i.e., highest education level between parents/caregivers), Age of Housing, and Intracranial Volume were centered continuous factors. The random effects structure included a random intercept for magnetic resonance imaging (MRI) scanner serial number and family identification number. Random effects were restricted to be uncorrelated. Analysis included 8,524 data points. The model accounted for 67.5% of the variance in the data (*R*^2^ = .675, adjusted *R*^2^ = .674).

**Supplementary Table 55. Linear mixed-effects model output for the analysis of the age-of-housing metric and volume of the ventral diencephalon, collapsed across hemispheres.**

|  | *t*(8508) | *p* | *b* | 95% CI |
| --- | --- | --- | --- | --- |
| Intercept | 155.32 | < .001 | 3935.62 | [3885.95, 3985.29] |
| Maximum Parental Education | 1.68 | .093 | 5.48 | [-0.91, 11.87] |
| Family Income (High) | 1.25 | .210 | 6.05 | [-3.41, 15.52] |
| Family Income (Low) | -1.05 | .294 | -5.76 | [-16.51, 4.99] |
| Sex | -1.97 | .048 | -6.18 | [-12.31, -0.04] |
| Child Race (American Indian/Alaska Native) | 0.79 | .429 | 28.73 | [-42.46, 99.91] |
| Child Race (Asian) | -2.71 | .007 | -61.62 | [-106.26, -16.99] |
| Child Race (Black) | 3.56 | < .001 | 64.61 | [29.02, 100.19] |
| Child Race (Native Hawaiian / Pacific Islander) | -1.28 | .199 | -95.01 | [-240.15, 50.14] |
| Child Race (Other) | 1.78 | .076 | 31.13 | [-3.24, 65.51] |
| Child Ethnicity | -0.13 | .895 | -0.59 | [-9.37, 8.18] |
| Age | 10.13 | < .001 | 3.67 | [2.96, 4.38] |
| Age of Housing | 1.46 | .143 | 2.54 | [-0.86, 5.94] |
| Intracranial Volume | 86.19 | < .001 | 0.002 | [0.002, 0.002] |
| Family Income (High) × Age of Housing | -0.06 | .955 | -0.11 | [-3.92, 3.70] |
| Family Income (Low) × Age of Housing | 0.80 | .425 | 1.73 | [-2.52, 5.99] |

**Note**: The linear mixed-effects model incorporates testing the statistical significance of coefficients against a *t*-distribution. Family Income was a categorical, effects-coded factor, in which the level “Mid” served as the reference level. Family Income was operationally defined as the self-reported combined family income and partitioned into three levels: Low Income: ≤ $50K; Mid Income: $50K-$100K; High Income: ≥ $100K. Sex was also a categorical factor, effect coded with Male/Female as ‑1/+1. Child Ethnicity was also a categorical factor, effect coded with Hispanic/Non-Hispanic as ‑1/+1. Child Race was also a categorical factor, in which “White” served as the reference level. Age, Maximum Parental Education (i.e., highest education level between parents/caregivers), Age of Housing, and Intracranial Volume were centered continuous factors. The random effects structure included a random intercept for magnetic resonance imaging (MRI) scanner serial number and family identification number. Random effects were restricted to be uncorrelated. Analysis included 8,524 data points. The model accounted for 80.8% of the variance in the data (*R*^2^ = .808, adjusted *R*^2^ = .808).

**Supplementary Table 56. Linear mixed-effects model output for the analysis of the age-of-housing metric and volume of the cerebellum cortex, collapsed across hemispheres.**

|  | *t*(8508) | *p* | *b* | 95% CI |
| --- | --- | --- | --- | --- |
| Intercept | 141.40 | < .001 | 52952.99 | [52218.92, 53687.07] |
| Maximum Parental Education | 4.03 | < .001 | 204.02 | [104.67, 303.37] |
| Family Income (High) | 1.54 | .124 | 115.58 | [-31.60, 262.75] |
| Family Income (Low) | -2.23 | .026 | -189.77 | [-356.95, -22.59] |
| Sex | -18.77 | < .001 | -893.39 | [-986.69, -800.09] |
| Child Race (American Indian/Alaska Native) | 0.18 | .858 | 100.57 | [-1000.23, 1201.37] |
| Child Race (Asian) | -1.96 | .050 | -694.71 | [-1390.35, 0.93] |
| Child Race (Black) | -0.46 | .642 | -132.43 | [-690.78, 425.92] |
| Child Race (Native Hawaiian / Pacific Islander) | -0.94 | .349 | -1096.14 | [-3391.07, 1198.79] |
| Child Race (Other) | 2.21 | .027 | 608.23 | [68.40, 1148.07] |
| Child Ethnicity | 6.02 | < .001 | 417.80 | [281.75, 553.84] |
| Age | -1.54 | .124 | -8.31 | [-18.89, 2.28] |
| Age of Housing | 0.09 | .927 | 2.48 | [-50.40, 55.36] |
| Intracranial Volume | 56.97 | < .001 | 0.02 | [0.02, 0.02] |
| Family Income (High) × Age of Housing | 1.50 | .133 | 45.50 | [-13.84, 104.84] |
| Family Income (Low) × Age of Housing | 0.24 | .811 | 8.08 | [-58.01, 74.17] |

**Note**: The linear mixed-effects model incorporates testing the statistical significance of coefficients against a *t*-distribution. Family Income was a categorical, effects-coded factor, in which the level “Mid” served as the reference level. Family Income was operationally defined as the self-reported combined family income and partitioned into three levels: Low Income: ≤ $50K; Mid Income: $50K-$100K; High Income: ≥ $100K. Sex was also a categorical factor, effect coded with Male/Female as ‑1/+1. Child Ethnicity was also a categorical factor, effect coded with Hispanic/Non-Hispanic as ‑1/+1. Child Race was also a categorical factor, in which “White” served as the reference level. Age, Maximum Parental Education (i.e., highest education level between parents/caregivers), Age of Housing, and Intracranial Volume were centered continuous factors. The random effects structure included a random intercept for magnetic resonance imaging (MRI) scanner serial number and family identification number. Random effects were restricted to be uncorrelated. Analysis included 8,524 data points. The model accounted for 86.2% of the variance in the data (*R*^2^ = .862, adjusted *R*^2^ = .862).

**Supplementary Table 57. Linear mixed-effects model output for the analysis of the age-of-housing metric and volume of cerebellar white matter, collapsed across hemispheres.**

|  | *t*(8508) | *p* | *b* | 95% CI |
| --- | --- | --- | --- | --- |
| Intercept | 133.76 | < .001 | 17103.79 | [16853.13, 17354.45] |
| Maximum Parental Education | 2.01 | .045 | 43.41 | [1.05, 85.77] |
| Family Income (High) | 1.49 | .135 | 47.76 | [-14.90, 110.42] |
| Family Income (Low) | -1.88 | .060 | -68.34 | [-139.56, 2.88] |
| Sex | -2.84 | .005 | -58.00 | [-98.04, -17.97] |
| Child Race (American Indian/Alaska Native) | 1.08 | .281 | 258.67 | [-211.89, 729.23] |
| Child Race (Asian) | -1.28 | .201 | -193.25 | [-489.66, 103.16] |
| Child Race (Black) | 2.68 | .007 | 324.92 | [87.50, 562.34] |
| Child Race (Native Hawaiian / Pacific Islander) | -0.94 | .348 | -466.94 | [-1441.97, 508.09] |
| Child Race (Other) | 0.71 | .477 | 83.45 | [-146.35, 313.24] |
| Child Ethnicity | 1.62 | .105 | 47.55 | [-9.87, 104.97] |
| Age | 7.20 | < .001 | 16.82 | [12.24, 21.40] |
| Age of Housing | -0.20 | .842 | -2.26 | [-24.40, 19.89] |
| Intracranial Volume | 50.20 | < .001 | 0.01 | [0.01, 0.01] |
| Family Income (High) × Age of Housing | 0.88 | .381 | 11.29 | [-13.96, 36.54] |
| Family Income (Low) × Age of Housing | 0.32 | .752 | 4.54 | [-23.59, 32.68] |

**Note**: The linear mixed-effects model incorporates testing the statistical significance of coefficients against a *t*-distribution. Family Income was a categorical, effects-coded factor, in which the level “Mid” served as the reference level. Family Income was operationally defined as the self-reported combined family income and partitioned into three levels: Low Income: ≤ $50K; Mid Income: $50K-$100K; High Income: ≥ $100K. Sex was also a categorical factor, effect coded with Male/Female as ‑1/+1. Child Ethnicity was also a categorical factor, effect coded with Hispanic/Non-Hispanic as ‑1/+1. Child Race was also a categorical factor, in which “White” served as the reference level. Age, Maximum Parental Education (i.e., highest education level between parents/caregivers), Age of Housing, and Intracranial Volume were centered continuous factors. The random effects structure included a random intercept for magnetic resonance imaging (MRI) scanner serial number and family identification number. Random effects were restricted to be uncorrelated. Analysis included 8,524 data points. The model accounted for 78.0% of the variance in the data (*R*^2^ = .780, adjusted *R*^2^ = .779).

**Supplementary Table 58. Linear mixed-effects model output for the analysis of the age-of-housing metric and volume of the brain stem.**

|  | *t*(8508) | *p* | *b* | 95% CI |
| --- | --- | --- | --- | --- |
| Intercept | 138.93 | < .001 | 19165.58 | [18895.16, 19436.00] |
| Maximum Parental Education | 1.44 | .149 | 25.85 | [-9.28, 60.99] |
| Family Income (High) | 1.70 | .089 | 45.16 | [-6.90, 97.22] |
| Family Income (Low) | -1.20 | .231 | -36.15 | [-95.28, 22.98] |
| Sex | -8.91 | < .001 | -150.90 | [-184.08, -117.71] |
| Child Race (American Indian/Alaska Native) | 1.03 | .302 | 205.46 | [-184.40, 595.33] |
| Child Race (Asian) | -2.28 | .023 | -285.47 | [-531.40, -39.53] |
| Child Race (Black) | 3.89 | < .001 | 391.01 | [193.88, 588.15] |
| Child Race (Native Hawaiian / Pacific Islander) | -1.17 | .244 | -481.28 | [-1290.24, 327.68] |
| Child Race (Other) | 1.18 | .236 | 115.15 | [-75.40, 305.71] |
| Child Ethnicity | -0.65 | .518 | -15.90 | [-64.07, 32.27] |
| Age | 12.73 | < .001 | 24.57 | [20.79, 28.35] |
| Age of Housing | 0.50 | .620 | 4.73 | [-13.99, 23.44] |
| Intracranial Volume | 74.18 | < .001 | 0.01 | [0.01, 0.01] |
| Family Income (High) × Age of Housing | 0.12 | .902 | 1.31 | [-19.67, 22.30] |
| Family Income (Low) × Age of Housing | 2.32 | .020 | 27.68 | [4.30, 51.06] |

**Note**: The linear mixed-effects model incorporates testing the statistical significance of coefficients against a *t*-distribution. Family Income was a categorical, effects-coded factor, in which the level “Mid” served as the reference level. Family Income was operationally defined as the self-reported combined family income and partitioned into three levels: Low Income: ≤ $50K; Mid Income: $50K-$100K; High Income: ≥ $100K. Sex was also a categorical factor, effect coded with Male/Female as ‑1/+1. Child Ethnicity was also a categorical factor, effect coded with Hispanic/Non-Hispanic as ‑1/+1. Child Race was also a categorical factor, in which “White” served as the reference level. Age, Maximum Parental Education (i.e., highest education level between parents/caregivers), Age of Housing, and Intracranial Volume were centered continuous factors. The random effects structure included a random intercept for magnetic resonance imaging (MRI) scanner serial number and family identification number. Random effects were restricted to be uncorrelated. Analysis included 8,524 data points. The model accounted for 85.5% of the variance in the data (*R*^2^ = .855, adjusted *R*^2^ = .855).

**Supplementary Table 59. Linear mixed-effects model output for the analysis of the age-of-housing metric and volume of the posterior corpus callosum.**

|  | *t*(8508) | *p* | *b* | 95% CI |
| --- | --- | --- | --- | --- |
| Intercept | 90.53 | < .001 | 796.18 | [778.94, 813.42] |
| Maximum Parental Education | 1.02 | .308 | 1.60 | [-1.47, 4.67] |
| Family Income (High) | -0.05 | .962 | -0.11 | [-4.64, 4.42] |
| Family Income (Low) | -0.61 | .543 | -1.60 | [-6.75, 3.55] |
| Sex | 2.01 | .045 | 2.98 | [0.07, 5.88] |
| Child Race (American Indian/Alaska Native) | 0.49 | .621 | 8.61 | [-25.53, 42.75] |
| Child Race (Asian) | 0.79 | .432 | 8.61 | [-12.85, 30.07] |
| Child Race (Black) | -0.01 | .995 | -0.06 | [-17.22, 17.11] |
| Child Race (Native Hawaiian / Pacific Islander) | -0.68 | .496 | -24.52 | [-95.05, 46.02] |
| Child Race (Other) | 1.01 | .313 | 8.56 | [-8.08, 25.20] |
| Child Ethnicity | -1.78 | .075 | -3.73 | [-7.84, 0.37] |
| Age | 7.39 | < .001 | 1.26 | [0.92, 1.59] |
| Age of Housing | -2.38 | .017 | -1.91 | [-3.48, -0.34] |
| Intracranial Volume | 28.72 | < .001 | 0.0003 | [0.0003, 0.0003] |
| Family Income (High) × Age of Housing | 0.93 | .351 | 0.87 | [-0.96, 2.69] |
| Family Income (Low) × Age of Housing | 0.41 | .685 | 0.42 | [-1.61, 2.45] |

**Note**: The linear mixed-effects model incorporates testing the statistical significance of coefficients against a *t*-distribution. Family Income was a categorical, effects-coded factor, in which the level “Mid” served as the reference level. Family Income was operationally defined as the self-reported combined family income and partitioned into three levels: Low Income: ≤ $50K; Mid Income: $50K-$100K; High Income: ≥ $100K. Sex was also a categorical factor, effect coded with Male/Female as ‑1/+1. Child Ethnicity was also a categorical factor, effect coded with Hispanic/Non-Hispanic as ‑1/+1. Child Race was also a categorical factor, in which “White” served as the reference level. Age, Maximum Parental Education (i.e., highest education level between parents/caregivers), Age of Housing, and Intracranial Volume were centered continuous factors. The random effects structure included a random intercept for magnetic resonance imaging (MRI) scanner serial number and family identification number. Random effects were restricted to be uncorrelated. Analysis included 8,524 data points. The model accounted for 60.3% of the variance in the data (*R*^2^ = .603, adjusted *R*^2^ = .602).

**Supplementary Table 60. Linear mixed-effects model output for the analysis of the age-of-housing metric and volume of the mid-posterior corpus callosum.**

|  | *t*(8508) | *p* | *b* | 95% CI |
| --- | --- | --- | --- | --- |
| Intercept | 70.87 | < .001 | 375.71 | [365.32, 386.10] |
| Maximum Parental Education | 0.93 | .354 | 0.89 | [-0.99, 2.78] |
| Family Income (High) | -0.92 | .357 | -1.30 | [-4.08, 1.47] |
| Family Income (Low) | -0.49 | .624 | -0.79 | [-3.95, 2.37] |
| Sex | 1.88 | .061 | 1.72 | [-0.08, 3.53] |
| Child Race (American Indian/Alaska Native) | 1.19 | .233 | 12.78 | [-8.24, 33.80] |
| Child Race (Asian) | -0.48 | .633 | -3.20 | [-16.35, 9.94] |
| Child Race (Black) | -1.51 | .131 | -8.06 | [-18.52, 2.41] |
| Child Race (Native Hawaiian / Pacific Islander) | -0.88 | .379 | -19.21 | [-62.06, 23.63] |
| Child Race (Other) | 1.53 | .127 | 7.91 | [-2.25, 18.06] |
| Child Ethnicity | 0.83 | .405 | 1.06 | [-1.44, 3.57] |
| Age | 7.60 | < .001 | 0.81 | [0.60, 1.02] |
| Age of Housing | -2.71 | .007 | -1.31 | [-2.27, -0.36] |
| Intracranial Volume | 19.31 | < .001 | 0.0001 | [0.0001, 0.0001] |
| Family Income (High) × Age of Housing | 0.05 | .960 | 0.03 | [-1.09, 1.15] |
| Family Income (Low) × Age of Housing | 1.03 | .304 | 0.65 | [-0.59, 1.90] |

**Note**: The linear mixed-effects model incorporates testing the statistical significance of coefficients against a *t*-distribution. Family Income was a categorical, effects-coded factor, in which the level “Mid” served as the reference level. Family Income was operationally defined as the self-reported combined family income and partitioned into three levels: Low Income: ≤ $50K; Mid Income: $50K-$100K; High Income: ≥ $100K. Sex was also a categorical factor, effect coded with Male/Female as ‑1/+1. Child Ethnicity was also a categorical factor, effect coded with Hispanic/Non-Hispanic as ‑1/+1. Child Race was also a categorical factor, in which “White” served as the reference level. Age, Maximum Parental Education (i.e., highest education level between parents/caregivers), Age of Housing, and Intracranial Volume were centered continuous factors. The random effects structure included a random intercept for magnetic resonance imaging (MRI) scanner serial number and family identification number. Random effects were restricted to be uncorrelated. Analysis included 8,524 data points. The model accounted for 43.0% of the variance in the data (*R*^2^ = .430, adjusted *R*^2^ = .429).

**Supplementary Table 61. Linear mixed-effects model output for the analysis of the age-of-housing metric and volume of the central corpus callosum.**

|  | *t*(8508) | *p* | *b* | 95% CI |
| --- | --- | --- | --- | --- |
| Intercept | 66.70 | < .001 | 405.33 | [393.42, 417.24] |
| Maximum Parental Education | -0.55 | .583 | -0.59 | [-2.68, 1.50] |
| Family Income (High) | -1.11 | .267 | -1.75 | [-4.83, 1.34] |
| Family Income (Low) | -0.27 | .785 | -0.49 | [-4.00, 3.02] |
| Sex | 3.40 | .001 | 3.49 | [1.48, 5.49] |
| Child Race (American Indian/Alaska Native) | 0.34 | .732 | 4.08 | [-19.23, 27.39] |
| Child Race (Asian) | 1.71 | .087 | 12.71 | [-1.86, 27.29] |
| Child Race (Black) | 0.69 | .491 | 4.07 | [-7.52, 15.67] |
| Child Race (Native Hawaiian / Pacific Islander) | -1.51 | .132 | -36.35 | [-83.65, 10.95] |
| Child Race (Other) | 1.83 | .068 | 10.46 | [-0.76, 21.68] |
| Child Ethnicity | 1.43 | .153 | 2.06 | [-0.77, 4.88] |
| Age | 5.00 | < .001 | 0.60 | [0.36, 0.83] |
| Age of Housing | -3.30 | .001 | -1.82 | [-2.90, -0.74] |
| Intracranial Volume | 17.66 | < .001 | 0.0001 | [0.0001, 0.0001] |
| Family Income (High) × Age of Housing | 1.13 | .257 | 0.72 | [-0.52, 1.96] |
| Family Income (Low) × Age of Housing | -0.26 | .793 | -0.19 | [-1.57, 1.20] |

**Note**: The linear mixed-effects model incorporates testing the statistical significance of coefficients against a *t*-distribution. Family Income was a categorical, effects-coded factor, in which the level “Mid” served as the reference level. Family Income was operationally defined as the self-reported combined family income and partitioned into three levels: Low Income: ≤ $50K; Mid Income: $50K-$100K; High Income: ≥ $100K. Sex was also a categorical factor, effect coded with Male/Female as ‑1/+1. Child Ethnicity was also a categorical factor, effect coded with Hispanic/Non-Hispanic as ‑1/+1. Child Race was also a categorical factor, in which “White” served as the reference level. Age, Maximum Parental Education (i.e., highest education level between parents/caregivers), Age of Housing, and Intracranial Volume were centered continuous factors. The random effects structure included a random intercept for magnetic resonance imaging (MRI) scanner serial number and family identification number. Random effects were restricted to be uncorrelated. Analysis included 8,524 data points. The model accounted for 36.9% of the variance in the data (*R*^2^ = .369, adjusted *R*^2^ = .368).

**Supplementary Table 62. Linear mixed-effects model output for the analysis of the age-of-housing metric and volume of the mid-anterior corpus callosum.**

|  | *t*(8508) | *p* | *b* | 95% CI |
| --- | --- | --- | --- | --- |
| Intercept | 63.03 | < .001 | 433.7 | [420.21, 447.19] |
| Maximum Parental Education | -2.08 | .038 | -2.51 | [-4.88, -0.14] |
| Family Income (High) | -1.24 | .214 | -2.22 | [-5.71, 1.28] |
| Family Income (Low) | 0.36 | .717 | 0.73 | [-3.24, 4.71] |
| Sex | -0.10 | .919 | -0.12 | [-2.39, 2.16] |
| Child Race (American Indian/Alaska Native) | 0.61 | .543 | 8.19 | [-18.22, 34.60] |
| Child Race (Asian) | 2.63 | .009 | 22.11 | [5.60, 38.61] |
| Child Race (Black) | -1.38 | .169 | -9.21 | [-22.34, 3.92] |
| Child Race (Native Hawaiian / Pacific Islander) | -1.42 | .156 | -38.77 | [-92.32, 14.78] |
| Child Race (Other) | 1.73 | .084 | 11.20 | [-1.50, 23.91] |
| Child Ethnicity | 0.82 | .413 | 1.34 | [-1.86, 4.53] |
| Age | 0.06 | .951 | 0.01 | [-0.26, 0.27] |
| Age of Housing | -3.16 | .002 | -1.98 | [-3.20, -0.75] |
| Intracranial Volume | 23.72 | < .001 | 0.0002 | [0.0002, 0.0002] |
| Family Income (High) × Age of Housing | 0.41 | .679 | 0.30 | [-1.11, 1.70] |
| Family Income (Low) × Age of Housing | 0.44 | .663 | 0.35 | [-1.22, 1.92] |

**Note**: The linear mixed-effects model incorporates testing the statistical significance of coefficients against a *t*-distribution. Family Income was a categorical, effects-coded factor, in which the level “Mid” served as the reference level. Family Income was operationally defined as the self-reported combined family income and partitioned into three levels: Low Income: ≤ $50K; Mid Income: $50K-$100K; High Income: ≥ $100K. Sex was also a categorical factor, effect coded with Male/Female as ‑1/+1. Child Ethnicity was also a categorical factor, effect coded with Hispanic/Non-Hispanic as ‑1/+1. Child Race was also a categorical factor, in which “White” served as the reference level. Age, Maximum Parental Education (i.e., highest education level between parents/caregivers), Age of Housing, and Intracranial Volume were centered continuous factors. The random effects structure included a random intercept for magnetic resonance imaging (MRI) scanner serial number and family identification number. Random effects were restricted to be uncorrelated. Analysis included 8,524 data points. The model accounted for 39.9% of the variance in the data (*R*^2^ = .399, adjusted *R*^2^ = .398).

**Supplementary Table 63. Linear mixed-effects model output for the analysis of the age-of-housing metric and volume of anterior corpus callosum.**

|  | *t*(8508) | *p* | *b* | 95% CI |
| --- | --- | --- | --- | --- |
| Intercept | 79.57 | < .001 | 778.12 | [758.95, 797.29] |
| Maximum Parental Education | -0.50 | .618 | -0.78 | [-3.86, 2.29] |
| Family Income (High) | -0.68 | .498 | -1.57 | [-6.12, 2.98] |
| Family Income (Low) | -0.19 | .850 | -0.50 | [-5.67, 4.67] |
| Sex | 4.24 | < .001 | 6.32 | [3.40, 9.24] |
| Child Race (American Indian/Alaska Native) | -0.78 | .434 | -13.65 | [-47.85, 20.55] |
| Child Race (Asian) | 0.33 | .741 | 3.63 | [-17.88, 25.13] |
| Child Race (Black) | 1.54 | .123 | 13.53 | [-3.67, 30.73] |
| Child Race (Native Hawaiian / Pacific Islander) | -1.12 | .262 | -40.34 | [-110.81, 30.13] |
| Child Race (Other) | 2.32 | .021 | 19.66 | [3.03, 36.30] |
| Child Ethnicity | 1.13 | .257 | 2.42 | [-1.77, 6.62] |
| Age | -0.23 | .819 | -0.04 | [-0.38, 0.30] |
| Age of Housing | -0.47 | .636 | -0.39 | [-2.01, 1.23] |
| Intracranial Volume | 37.17 | < .001 | 0.0004 | [0.0004, 0.0004] |
| Family Income (High) × Age of Housing | 0.87 | .385 | 0.81 | [-1.02, 2.65] |
| Family Income (Low) × Age of Housing | -0.50 | .619 | -0.52 | [-2.56, 1.53] |

**Note**: The linear mixed-effects model incorporates testing the statistical significance of coefficients against a *t*-distribution. Family Income was a categorical, effects-coded factor, in which the level “Mid” served as the reference level. Family Income was operationally defined as the self-reported combined family income and partitioned into three levels: Low Income: ≤ $50K; Mid Income: $50K-$100K; High Income: ≥ $100K. Sex was also a categorical factor, effect coded with Male/Female as ‑1/+1. Child Ethnicity was also a categorical factor, effect coded with Hispanic/Non-Hispanic as ‑1/+1. Child Race was also a categorical factor, in which “White” served as the reference level. Age, Maximum Parental Education (i.e., highest education level between parents/caregivers), Age of Housing, and Intracranial Volume were centered continuous factors. The random effects structure included a random intercept for magnetic resonance imaging (MRI) scanner serial number and family identification number. Random effects were restricted to be uncorrelated. Analysis included 8,524 data points. The model accounted for 63.4% of the variance in the data (*R*^2^ = .634, adjusted *R*^2^ = .633).

**Supplementary Table 64. Linear mixed-effects model output for the analysis of the age-of-housing metric and volume of the lateral ventricles, collapsed across hemispheres.**

|  | *t*(8508) | *p* | *b* | 95% CI |
| --- | --- | --- | --- | --- |
| Intercept | 23.68 | < .001 | 4672.21 | [4285.43, 5059.00] |
| Maximum Parental Education | -0.65 | .518 | -20.15 | [-81.29, 40.98] |
| Family Income (High) | -0.05 | .961 | -2.23 | [-92.71, 88.25] |
| Family Income (Low) | 0.37 | .712 | 19.38 | [-83.44, 122.20] |
| Sex | 1.66 | .096 | 50.01 | [-8.93, 108.94] |
| Child Race (American Indian/Alaska Native) | -0.47 | .639 | -163.02 | [-844.94, 518.90] |
| Child Race (Asian) | -0.58 | .562 | -126.08 | [-552.49, 300.34] |
| Child Race (Black) | 1.62 | .104 | 280.92 | [-58.05, 619.89] |
| Child Race (Native Hawaiian / Pacific Islander) | 0.51 | .612 | 356.84 | [-1022.85, 1736.53] |
| Child Race (Other) | -0.85 | .393 | -142.69 | [-470.27, 184.88] |
| Child Ethnicity | 0.43 | .667 | 18.36 | [-65.23, 101.94] |
| Age | 2.42 | .016 | 8.46 | [1.60, 15.33] |
| Age of Housing | 0.81 | .418 | 13.33 | [-18.92, 45.58] |
| Intracranial Volume | 30.95 | < .001 | 0.01 | [0.01, 0.01] |
| Family Income (High) × Age of Housing | 0.26 | .793 | 4.87 | [-31.56, 41.31] |
| Family Income (Low) × Age of Housing | 0.06 | .950 | 1.31 | [-39.38, 42.00] |

**Note**: The linear mixed-effects model incorporates testing the statistical significance of coefficients against a *t*-distribution. Family Income was a categorical, effects-coded factor, in which the level “Mid” served as the reference level. Family Income was operationally defined as the self-reported combined family income and partitioned into three levels: Low Income: ≤ $50K; Mid Income: $50K-$100K; High Income: ≥ $100K. Sex was also a categorical factor, effect coded with Male/Female as ‑1/+1. Child Ethnicity was also a categorical factor, effect coded with Hispanic/Non-Hispanic as ‑1/+1. Child Race was also a categorical factor, in which “White” served as the reference level. Age, Maximum Parental Education (i.e., highest education level between parents/caregivers), Age of Housing, and Intracranial Volume were centered continuous factors. The random effects structure included a random intercept for magnetic resonance imaging (MRI) scanner serial number and family identification number. Random effects were restricted to be uncorrelated. Analysis included 8,524 data points. The model accounted for 40.3% of the variance in the data (*R*^2^ = .403, adjusted *R*^2^ = .402).

**Supplementary Table 65. Linear mixed-effects model output for the analysis of the age-of-housing metric and volume of the inferior lateral ventricles, collapsed across hemispheres.**

|  | *t*(8508) | *p* | *b* | 95% CI |
| --- | --- | --- | --- | --- |
| Intercept | 24.77 | < .001 | 309.78 | [285.27, 334.29] |
| Maximum Parental Education | -1.53 | .127 | -2.48 | [-5.66, 0.71] |
| Family Income (High) | 0.80 | .422 | 1.93 | [-2.78, 6.64] |
| Family Income (Low) | 0.24 | .812 | 0.65 | [-4.70, 6.00] |
| Sex | -4.12 | < .001 | -6.47 | [-9.55, -3.39] |
| Child Race (American Indian/Alaska Native) | -0.32 | .749 | -5.80 | [-41.30, 29.71] |
| Child Race (Asian) | 0.89 | .372 | 10.09 | [-12.07, 32.25] |
| Child Race (Black) | -1.62 | .106 | -14.48 | [-32.05, 3.08] |
| Child Race (Native Hawaiian / Pacific Islander) | 0.87 | .382 | 31.78 | [-39.45, 103.00] |
| Child Race (Other) | -1.26 | .207 | -10.93 | [-27.88, 6.03] |
| Child Ethnicity | -2.50 | .013 | -5.57 | [-9.94, -1.20] |
| Age | 1.53 | .126 | 0.28 | [-0.08, 0.64] |
| Age of Housing | 0.50 | .614 | 0.44 | [-1.26, 2.13] |
| Intracranial Volume | 12.91 | < .001 | 0.0002 | [0.0001, 0.0002] |
| Family Income (High) × Age of Housing | -0.12 | .901 | -0.12 | [-2.02, 1.78] |
| Family Income (Low) × Age of Housing | -1.57 | .117 | -1.70 | [-3.82, 0.42] |

**Note**: The linear mixed-effects model incorporates testing the statistical significance of coefficients against a *t*-distribution. Family Income was a categorical, effects-coded factor, in which the level “Mid” served as the reference level. Family Income was operationally defined as the self-reported combined family income and partitioned into three levels: Low Income: ≤ $50K; Mid Income: $50K-$100K; High Income: ≥ $100K. Sex was also a categorical factor, effect coded with Male/Female as ‑1/+1. Child Ethnicity was also a categorical factor, effect coded with Hispanic/Non-Hispanic as ‑1/+1. Child Race was also a categorical factor, in which “White” served as the reference level. Age, Maximum Parental Education (i.e., highest education level between parents/caregivers), Age of Housing, and Intracranial Volume were centered continuous factors. The random effects structure included a random intercept for magnetic resonance imaging (MRI) scanner serial number and family identification number. Random effects were restricted to be uncorrelated. Analysis included 8,524 data points. The model accounted for 30.1% of the variance in the data (*R*^2^ = .301, adjusted *R*^2^ = .300).

**Supplementary Table 66. Linear mixed-effects model output for the analysis of the age-of-housing metric and volume of the 3^rd^ ventricle.**

|  | *t*(8508) | *p* | *b* | 95% CI |
| --- | --- | --- | --- | --- |
| Intercept | 47.46 | < .001 | 755.88 | [724.66, 787.10] |
| Maximum Parental Education | -1.08 | .280 | -2.79 | [-7.84, 2.27] |
| Family Income (High) | -0.31 | .754 | -1.20 | [-8.67, 6.28] |
| Family Income (Low) | 2.20 | .028 | 9.54 | [1.04, 18.03] |
| Sex | 0.21 | .834 | 0.52 | [-4.33, 5.37] |
| Child Race (American Indian/Alaska Native) | 0.12 | .904 | 3.47 | [-52.86, 59.79] |
| Child Race (Asian) | 1.79 | .074 | 32.14 | [-3.14, 67.43] |
| Child Race (Black) | 1.14 | .256 | 16.30 | [-11.81, 44.41] |
| Child Race (Native Hawaiian / Pacific Islander) | -0.33 | .741 | -19.37 | [-134.06, 95.32] |
| Child Race (Other) | -0.90 | .368 | -12.48 | [-39.65, 14.69] |
| Child Ethnicity | -0.28 | .780 | -0.98 | [-7.88, 5.91] |
| Age | 0.29 | .776 | 0.08 | [-0.48, 0.64] |
| Age of Housing | 1.25 | .210 | 1.70 | [-0.96, 4.36] |
| Intracranial Volume | 31.92 | < .001 | 0.001 | [0.001, 0.001] |
| Family Income (High) × Age of Housing | -1.14 | .253 | -1.76 | [-4.77, 1.26] |
| Family Income (Low) × Age of Housing | -0.75 | .454 | -1.28 | [-4.64, 2.08] |

**Note**: The linear mixed-effects model incorporates testing the statistical significance of coefficients against a *t*-distribution. Family Income was a categorical, effects-coded factor, in which the level “Mid” served as the reference level. Family Income was operationally defined as the self-reported combined family income and partitioned into three levels: Low Income: ≤ $50K; Mid Income: $50K-$100K; High Income: ≥ $100K. Sex was also a categorical factor, effect coded with Male/Female as ‑1/+1. Child Ethnicity was also a categorical factor, effect coded with Hispanic/Non-Hispanic as ‑1/+1. Child Race was also a categorical factor, in which “White” served as the reference level. Age, Maximum Parental Education (i.e., highest education level between parents/caregivers), Age of Housing, and Intracranial Volume were centered continuous factors. The random effects structure included a random intercept for magnetic resonance imaging (MRI) scanner serial number and family identification number. Random effects were restricted to be uncorrelated. Analysis included 8,524 data points. The model accounted for 47.3% of the variance in the data (*R*^2^ = .473, adjusted *R*^2^ = .473).

**Supplementary Table 67. Linear mixed-effects model output for the analysis of the age-of-housing metric and volume of the 4^th^ ventricle.**

|  | *t*(8508) | *p* | *b* | 95% CI |
| --- | --- | --- | --- | --- |
| Intercept | 46.64 | < .001 | 1786.82 | [1711.72, 1861.92] |
| Maximum Parental Education | 3.86 | < .001 | 26.56 | [13.08, 40.05] |
| Family Income (High) | 1.46 | .145 | 14.78 | [-5.12, 34.68] |
| Family Income (Low) | 0.08 | .938 | 0.90 | [-21.73, 23.54] |
| Sex | -8.43 | < .001 | -55.18 | [-68.01, -42.35] |
| Child Race (American Indian/Alaska Native) | -0.67 | .505 | -51.09 | [-201.32, 99.13] |
| Child Race (Asian) | 0.59 | .555 | 28.35 | [-65.87, 122.56] |
| Child Race (Black) | 0.18 | .857 | 6.91 | [-68.31, 82.13] |
| Child Race (Native Hawaiian / Pacific Islander) | 0.98 | .328 | 154.07 | [-154.53, 462.68] |
| Child Race (Other) | -1.45 | .148 | -53.87 | [-126.81, 19.06] |
| Child Ethnicity | -0.80 | .425 | -7.33 | [-25.31, 10.66] |
| Age | 1.71 | .087 | 1.30 | [-0.19, 2.78] |
| Age of Housing | 0.65 | .512 | 2.29 | [-4.57, 9.15] |
| Intracranial Volume | 17.79 | < .001 | 0.001 | [0.001, 0.001] |
| Family Income (High) × Age of Housing | 1.87 | .062 | 7.63 | [-0.38, 15.65] |
| Family Income (Low) × Age of Housing | 0.63 | .531 | 2.85 | [-6.08, 11.78] |

**Note**: The linear mixed-effects model incorporates testing the statistical significance of coefficients against a *t*-distribution. Family Income was a categorical, effects-coded factor, in which the level “Mid” served as the reference level. Family Income was operationally defined as the self-reported combined family income and partitioned into three levels: Low Income: ≤ $50K; Mid Income: $50K-$100K; High Income: ≥ $100K. Sex was also a categorical factor, effect coded with Male/Female as ‑1/+1. Child Ethnicity was also a categorical factor, effect coded with Hispanic/Non-Hispanic as ‑1/+1. Child Race was also a categorical factor, in which “White” served as the reference level. Age, Maximum Parental Education (i.e., highest education level between parents/caregivers), Age of Housing, and Intracranial Volume were centered continuous factors. The random effects structure included a random intercept for magnetic resonance imaging (MRI) scanner serial number and family identification number. Random effects were restricted to be uncorrelated. Analysis included 8,524 data points. The model accounted for 50.3% of the variance in the data (*R*^2^ = .503, adjusted *R*^2^ = .502).

**Supplementary Table 68. Linear mixed-effects model output for the analysis of the age-of-housing metric and subcortical gray matter volume.**

|  | *t*(8508) | *p* | *b* | 95% CI |
| --- | --- | --- | --- | --- |
| Intercept | 223.03 | < .001 | 60404.52 | [59873.62, 60935.43] |
| Maximum Parental Education | 2.13 | .033 | 74.79 | [5.98, 143.61] |
| Family Income (High) | 1.87 | .061 | 97.34 | [-4.62, 199.29] |
| Family Income (Low) | -2.91 | .004 | -172.18 | [-287.99, -56.37] |
| Sex | -6.51 | < .001 | -216.80 | [-282.06, -151.53] |
| Child Race (American Indian/Alaska Native) | -0.83 | .407 | -323.30 | [-1087.69, 441.09] |
| Child Race (Asian) | 0.38 | .708 | 92.15 | [-389.36, 573.66] |
| Child Race (Black) | -1.12 | .263 | -220.20 | [-605.71, 165.31] |
| Child Race (Native Hawaiian / Pacific Islander) | 0.41 | .679 | 333.65 | [-1246.27, 1913.57] |
| Child Race (Other) | 0.21 | .832 | 40.25 | [-332.36, 412.85] |
| Child Ethnicity | -0.75 | .451 | -36.27 | [-130.65, 58.12] |
| Age | 0.23 | .815 | 0.89 | [-6.58, 8.36] |
| Age of Housing | -0.20 | .845 | -3.65 | [-40.30, 33.00] |
| Intracranial Volume | 113.83 | < .001 | 0.03 | [0.03, 0.03] |
| Family Income (High) × Age of Housing | 0.37 | .714 | 7.69 | [-33.40, 48.79] |
| Family Income (Low) × Age of Housing | 0.99 | .324 | 23.04 | [-22.76, 68.84] |

**Note**: The linear mixed-effects model incorporates testing the statistical significance of coefficients against a *t*-distribution. Family Income was a categorical, effects-coded factor, in which the level “Mid” served as the reference level. Family Income was operationally defined as the self-reported combined family income and partitioned into three levels: Low Income: ≤ $50K; Mid Income: $50K-$100K; High Income: ≥ $100K. Sex was also a categorical factor, effect coded with Male/Female as ‑1/+1. Child Ethnicity was also a categorical factor, effect coded with Hispanic/Non-Hispanic as ‑1/+1. Child Race was also a categorical factor, in which “White” served as the reference level. Age, Maximum Parental Education (i.e., highest education level between parents/caregivers), Age of Housing, and Intracranial Volume were centered continuous factors. The random effects structure included a random intercept for magnetic resonance imaging (MRI) scanner serial number and family identification number. Random effects were restricted to be uncorrelated. Analysis included 8,524 data points. The model accounted for 91.3% of the variance in the data (*R*^2^ = .913, adjusted *R*^2^ = .913).

**Supplementary Table 69. Linear mixed-effects model output for the analysis of posterior corpus callosum volume and scores on the dimensional change card sort test (NIH Toolbox).**

|  | *t*(8511) | *p* | *b* | 95% CI |
| --- | --- | --- | --- | --- |
| Intercept | 150.78 | < .001 | 90.24 | [89.06, 91.41] |
| Maximum Parental Education | 7.96 | < .001 | 0.88 | [0.66, 1.10] |
| Family Income (High) | 3.26 | .001 | 0.53 | [0.21, 0.85] |
| Family Income (Low) | -4.92 | < .001 | -0.91 | [-1.28, -0.55] |
| Sex | 7.62 | < .001 | 0.73 | [0.54, 0.92] |
| Child Race (American Indian/Alaska Native) | -1.46 | .145 | -1.82 | [-4.27, 0.63] |
| Child Race (Asian) | 3.33 | .001 | 2.57 | [1.06, 4.09] |
| Child Race (Black) | -2.21 | .027 | -1.34 | [-2.53, -0.15] |
| Child Race (Native Hawaiian / Pacific Islander) | -1.04 | .298 | -2.58 | [-7.43, 2.27] |
| Child Race (Other) | 2.43 | .015 | 1.44 | [0.28, 2.60] |
| Child Ethnicity | 1.48 | .140 | 0.22 | [-0.07, 0.50] |
| Age | 17.98 | < .001 | 0.23 | [0.21, 0.26] |
| Posterior Corpus Callosum Volume | 4.18 | < .001 | 0.003 | [0.002, 0.005] |

**Note**: The linear mixed-effects model incorporates testing the statistical significance of coefficients against a *t*-distribution. Family Income was a categorical, effects-coded factor, in which the level “Mid” served as the reference level. Family Income was operationally defined as the self-reported combined family income and partitioned into three levels: Low Income: ≤ $50K; Mid Income: $50K-$100K; High Income: ≥ $100K. Sex was also a categorical factor, effect coded with Male/Female as ‑1/+1. Child Ethnicity was also a categorical factor, effect coded with Hispanic/Non-Hispanic as ‑1/+1. Child Race was also a categorical factor, in which “White” served as the reference level. Age, Maximum Parental Education (i.e., highest education level between parents/caregivers), and Posterior Corpus Callosum Volume were centered continuous factors. The random effects structure included a random intercept for study site identification number and family identification number. Random effects were restricted to be uncorrelated. Analysis included 8,524 data points. The model accounted for 21.5% of the variance in the data (*R*^2^ = .215, adjusted *R*^2^ = .214).

**Supplementary Table 70. Linear mixed-effects model output for the analysis of posterior corpus callosum volume and scores on the** **flanker inhibitory control and attention test (NIH Toolbox).**

|  | *t*(8511) | *p* | *b* | 95% CI |
| --- | --- | --- | --- | --- |
| Intercept | 160.69 | < .001 | 93.82 | [92.68, 94.97] |
| Maximum Parental Education | 6.26 | < .001 | 0.67 | [0.46, 0.88] |
| Family Income (High) | 3.55 | < .001 | 0.56 | [0.25, 0.87] |
| Family Income (Low) | -4.50 | < .001 | -0.81 | [-1.16, -0.46] |
| Sex | -1.21 | .228 | -0.11 | [-0.30, 0.07] |
| Child Race (American Indian/Alaska Native) | -1.06 | .289 | -1.28 | [-3.66, 1.09] |
| Child Race (Asian) | 2.43 | .015 | 1.82 | [0.35, 3.29] |
| Child Race (Black) | -4.66 | < .001 | -2.74 | [-3.89, -1.58] |
| Child Race (Native Hawaiian / Pacific Islander) | 1.21 | .228 | 2.88 | [-1.80, 7.55] |
| Child Race (Other) | -0.57 | .565 | -0.33 | [-1.45, 0.79] |
| Child Ethnicity | 0.51 | .612 | 0.07 | [-0.21, 0.35] |
| Age | 17.38 | < .001 | 0.22 | [0.19, 0.24] |
| Posterior Corpus Callosum Volume | 2.71 | .007 | 0.002 | [0.001, 0.003] |

**Note**: The linear mixed-effects model incorporates testing the statistical significance of coefficients against a *t*-distribution. Family Income was a categorical, effects-coded factor, in which the level “Mid” served as the reference level. Family Income was operationally defined as the self-reported combined family income and partitioned into three levels: Low Income: ≤ $50K; Mid Income: $50K-$100K; High Income: ≥ $100K. Sex was also a categorical factor, effect coded with Male/Female as ‑1/+1. Child Ethnicity was also a categorical factor, effect coded with Hispanic/Non-Hispanic as ‑1/+1. Child Race was also a categorical factor, in which “White” served as the reference level. Age, Maximum Parental Education (i.e., highest education level between parents/caregivers), and Posterior Corpus Callosum Volume were centered continuous factors. The random effects structure included a random intercept for study site identification number and family identification number. Random effects were restricted to be uncorrelated. Analysis included 8,524 data points. The model accounted for 16.2% of the variance in the data (*R*^2^ = .162, adjusted *R*^2^ = .161).

**Supplementary Table 71. Linear mixed-effects model output for the analysis of posterior corpus callosum volume and scores on the list sorting working memory test (NIH Toolbox).**

|  | *t*(8511) | *p* | *b* | 95% CI |
| --- | --- | --- | --- | --- |
| Intercept | 123.00 | < .001 | 93.33 | [91.84, 94.82] |
| Maximum Parental Education | 13.05 | < .001 | 1.83 | [1.55, 2.10] |
| Family Income (High) | 5.27 | < .001 | 1.09 | [0.68, 1.49] |
| Family Income (Low) | -5.77 | < .001 | -1.35 | [-1.81, -0.89] |
| Sex | -2.67 | .008 | -0.32 | [-0.56, -0.09] |
| Child Race (American Indian/Alaska Native) | -0.38 | .701 | -0.61 | [-3.70, 2.49] |
| Child Race (Asian) | 0.97 | .330 | 0.95 | [-0.97, 2.87] |
| Child Race (Black) | -4.58 | < .001 | -3.52 | [-5.03, -2.02] |
| Child Race (Native Hawaiian / Pacific Islander) | 0.47 | .641 | 1.46 | [-4.69, 7.62] |
| Child Race (Other) | 0.59 | .554 | 0.44 | [-1.03, 1.91] |
| Child Ethnicity | 2.70 | .007 | 0.50 | [0.14, 0.86] |
| Age | 13.84 | < .001 | 0.22 | [0.19, 0.26] |
| Posterior Corpus Callosum Volume | 1.53 | .127 | 0.001 | [-0.0004, 0.003] |

**Note**: The linear mixed-effects model incorporates testing the statistical significance of coefficients against a *t*-distribution. Family Income was a categorical, effects-coded factor, in which the level “Mid” served as the reference level. Family Income was operationally defined as the self-reported combined family income and partitioned into three levels: Low Income: ≤ $50K; Mid Income: $50K-$100K; High Income: ≥ $100K. Sex was also a categorical factor, effect coded with Male/Female as ‑1/+1. Child Ethnicity was also a categorical factor, effect coded with Hispanic/Non-Hispanic as ‑1/+1. Child Race was also a categorical factor, in which “White” served as the reference level. Age, Maximum Parental Education (i.e., highest education level between parents/caregivers), and Posterior Corpus Callosum Volume were centered continuous factors. The random effects structure included a random intercept for study site identification number and family identification number. Random effects were restricted to be uncorrelated. Analysis included 8,524 data points. The model accounted for 27.1% of the variance in the data (*R*^2^ = .271, adjusted *R*^2^ = .270).

**Supplementary Table 72. Linear mixed-effects model output for the analysis of posterior corpus callosum volume and scores on the pattern comparison processing speed test (NIH Toolbox).**

|  | *t*(8511) | *p* | *b* | 95% CI |
| --- | --- | --- | --- | --- |
| Intercept | 86.88 | < .001 | 87.77 | [85.78, 89.75] |
| Maximum Parental Education | 4.22 | < .001 | 0.75 | [0.40, 1.09] |
| Family Income (High) | 0.39 | .700 | 0.10 | [-0.41, 0.61] |
| Family Income (Low) | -1.44 | .151 | -0.43 | [-1.01, 0.16] |
| Sex | 6.53 | < .001 | 1.00 | [0.70, 1.30] |
| Child Race (American Indian/Alaska Native) | -1.85 | .065 | -3.68 | [-7.59, 0.23] |
| Child Race (Asian) | 2.42 | .015 | 3.00 | [0.57, 5.42] |
| Child Race (Black) | -2.59 | .010 | -2.52 | [-4.42, -0.61] |
| Child Race (Native Hawaiian / Pacific Islander) | 0.74 | .461 | 2.92 | [-4.83, 10.66] |
| Child Race (Other) | 0.09 | .927 | 0.09 | [-1.76, 1.94] |
| Child Ethnicity | 0.53 | .595 | 0.13 | [-0.34, 0.60] |
| Age | 20.59 | < .001 | 0.42 | [0.38, 0.46] |
| Posterior Corpus Callosum Volume | 2.98 | .003 | 0.004 | [0.001, 0.01] |

**Note**: The linear mixed-effects model incorporates testing the statistical significance of coefficients against a *t*-distribution. Family Income was a categorical, effects-coded factor, in which the level “Mid” served as the reference level. Family Income was operationally defined as the self-reported combined family income and partitioned into three levels: Low Income: ≤ $50K; Mid Income: $50K-$100K; High Income: ≥ $100K. Sex was also a categorical factor, effect coded with Male/Female as ‑1/+1. Child Ethnicity was also a categorical factor, effect coded with Hispanic/Non-Hispanic as ‑1/+1. Child Race was also a categorical factor, in which “White” served as the reference level. Age, Maximum Parental Education (i.e., highest education level between parents/caregivers), and Posterior Corpus Callosum Volume were centered continuous factors. The random effects structure included a random intercept for study site identification number and family identification number. Random effects were restricted to be uncorrelated. Analysis included 8,524 data points. The model accounted for 19.4% of the variance in the data (*R*^2^ = .194, adjusted *R*^2^ = .193).

**Supplementary Table 73. Linear mixed-effects model output for the analysis of posterior corpus callosum volume and scores on the picture sequence memory test (NIH Toolbox).**

|  | *t*(8511) | *p* | *b* | 95% CI |
| --- | --- | --- | --- | --- |
| Intercept | 127.60 | < .001 | 101.41 | [99.85, 102.97] |
| Maximum Parental Education | 6.84 | < .001 | 1.01 | [0.72, 1.30] |
| Family Income (High) | 4.27 | < .001 | 0.93 | [0.50, 1.35] |
| Family Income (Low) | -3.45 | .001 | -0.85 | [-1.33, -0.37] |
| Sex | 6.04 | < .001 | 0.77 | [0.52, 1.02] |
| Child Race (American Indian/Alaska Native) | 0.00 | .997 | -0.01 | [-3.26, 3.25] |
| Child Race (Asian) | 1.22 | .221 | 1.26 | [-0.76, 3.28] |
| Child Race (Black) | -5.11 | < .001 | -4.15 | [-5.74, -2.56] |
| Child Race (Native Hawaiian / Pacific Islander) | 0.47 | .638 | 1.57 | [-4.96, 8.09] |
| Child Race (Other) | 0.33 | .740 | 0.26 | [-1.29, 1.82] |
| Child Ethnicity | 0.24 | .810 | 0.05 | [-0.33, 0.42] |
| Age | 10.78 | < .001 | 0.18 | [0.15, 0.22] |
| Posterior Corpus Callosum Volume | 0.34 | .732 | 0.0003 | [-0.002, 0.002] |

**Note**: The linear mixed-effects model incorporates testing the statistical significance of coefficients against a *t*-distribution. Family Income was a categorical, effects-coded factor, in which the level “Mid” served as the reference level. Family Income was operationally defined as the self-reported combined family income and partitioned into three levels: Low Income: ≤ $50K; Mid Income: $50K-$100K; High Income: ≥ $100K. Sex was also a categorical factor, effect coded with Male/Female as ‑1/+1. Child Ethnicity was also a categorical factor, effect coded with Hispanic/Non-Hispanic as ‑1/+1. Child Race was also a categorical factor, in which “White” served as the reference level. Age, Maximum Parental Education (i.e., highest education level between parents/caregivers), and Posterior Corpus Callosum Volume were centered continuous factors. The random effects structure included a random intercept for study site identification number and family identification number. Random effects were restricted to be uncorrelated. Analysis included 8,524 data points. The model accounted for 24.2% of the variance in the data (*R*^2^ = .242, adjusted *R*^2^ = .241).

**Supplementary Table 74. Linear mixed-effects model output for the analysis of posterior corpus callosum volume and scores on the oral reading recognition test (NIH Toolbox).**

|  | *t*(8511) | *p* | *b* | 95% CI |
| --- | --- | --- | --- | --- |
| Intercept | 193.57 | < .001 | 89.21 | [88.31, 90.11] |
| Maximum Parental Education | 16.53 | < .001 | 1.30 | [1.14, 1.45] |
| Family Income (High) | 6.00 | < .001 | 0.70 | [0.47, 0.92] |
| Family Income (Low) | -8.12 | < .001 | -1.07 | [-1.33, -0.81] |
| Sex | 1.29 | .196 | 0.09 | [-0.04, 0.22] |
| Child Race (American Indian/Alaska Native) | -0.57 | .571 | -0.50 | [-2.23, 1.23] |
| Child Race (Asian) | 2.62 | .009 | 1.45 | [0.36, 2.53] |
| Child Race (Black) | -5.09 | < .001 | -2.23 | [-3.09, -1.37] |
| Child Race (Native Hawaiian / Pacific Islander) | 0.68 | .493 | 1.23 | [-2.29, 4.75] |
| Child Race (Other) | 0.12 | .902 | 0.05 | [-0.78, 0.89] |
| Child Ethnicity | 2.57 | .010 | 0.27 | [0.06, 0.48] |
| Age | 23.20 | < .001 | 0.20 | [0.19, 0.22] |
| Posterior Corpus Callosum Volume | 3.25 | .001 | 0.002 | [0.001, 0.003] |

**Note**: The linear mixed-effects model incorporates testing the statistical significance of coefficients against a *t*-distribution. Family Income was a categorical, effects-coded factor, in which the level “Mid” served as the reference level. Family Income was operationally defined as the self-reported combined family income and partitioned into three levels: Low Income: ≤ $50K; Mid Income: $50K-$100K; High Income: ≥ $100K. Sex was also a categorical factor, effect coded with Male/Female as ‑1/+1. Child Ethnicity was also a categorical factor, effect coded with Hispanic/Non-Hispanic as ‑1/+1. Child Race was also a categorical factor, in which “White” served as the reference level. Age, Maximum Parental Education (i.e., highest education level between parents/caregivers), and Posterior Corpus Callosum Volume were centered continuous factors. The random effects structure included a random intercept for study site identification number and family identification number. Random effects were restricted to be uncorrelated. Analysis included 8,524 data points. The model accounted for 52.8% of the variance in the data (*R*^2^ = .528, adjusted *R*^2^ = .527).

**Supplementary Table 75. Linear mixed-effects model output for the analysis of posterior corpus callosum volume and scores on the picture vocabulary test (NIH Toolbox).**

|  | *t*(8511) | *p* | *b* | 95% CI |
| --- | --- | --- | --- | --- |
| Intercept | 161.86 | < .001 | 79.52 | [78.55, 80.48] |
| Maximum Parental Education | 18.87 | < .001 | 1.63 | [1.46, 1.80] |
| Family Income (High) | 6.84 | < .001 | 0.87 | [0.62, 1.12] |
| Family Income (Low) | -8.22 | < .001 | -1.19 | [-1.48, -0.91] |
| Sex | -1.25 | .212 | -0.09 | [-0.24, 0.05] |
| Child Race (American Indian/Alaska Native) | -0.50 | .616 | -0.49 | [-2.39, 1.42] |
| Child Race (Asian) | 2.89 | .004 | 1.75 | [0.56, 2.95] |
| Child Race (Black) | -3.55 | < .001 | -1.71 | [-2.65, -0.76] |
| Child Race (Native Hawaiian / Pacific Islander) | -2.41 | .016 | -4.75 | [-8.61, -0.89] |
| Child Race (Other) | 4.75 | < .001 | 2.22 | [1.30, 3.14] |
| Child Ethnicity | 8.80 | < .001 | 1.03 | [0.80, 1.26] |
| Age | 27.07 | < .001 | 0.27 | [0.25, 0.28] |
| Posterior Corpus Callosum Volume | 5.34 | < .001 | 0.003 | [0.002, 0.004] |

**Note**: The linear mixed-effects model incorporates testing the statistical significance of coefficients against a *t*-distribution. Family Income was a categorical, effects-coded factor, in which the level “Mid” served as the reference level. Family Income was operationally defined as the self-reported combined family income and partitioned into three levels: Low Income: ≤ $50K; Mid Income: $50K-$100K; High Income: ≥ $100K. Sex was also a categorical factor, effect coded with Male/Female as ‑1/+1. Child Ethnicity was also a categorical factor, effect coded with Hispanic/Non-Hispanic as ‑1/+1. Child Race was also a categorical factor, in which “White” served as the reference level. Age, Maximum Parental Education (i.e., highest education level between parents/caregivers), and Posterior Corpus Callosum Volume were centered continuous factors. The random effects structure included a random intercept for study site identification number and family identification number. Random effects were restricted to be uncorrelated. Analysis included 8,524 data points. The model accounted for 56.2% of the variance in the data (*R*^2^ = .562, adjusted *R*^2^ = .561).

**Supplementary Table 76. Linear mixed-effects model output for the analysis of mid-posterior corpus callosum volume and scores on the dimensional change card sort test (NIH Toolbox).**

|  | *t*(8511) | *p* | *b* | 95% CI |
| --- | --- | --- | --- | --- |
| Intercept | 150.25 | < .001 | 90.26 | [89.08, 91.44] |
| Maximum Parental Education | 8.00 | < .001 | 0.89 | [0.67, 1.10] |
| Family Income (High) | 3.30 | .001 | 0.54 | [0.22, 0.86] |
| Family Income (Low) | -4.94 | < .001 | -0.92 | [-1.28, -0.55] |
| Sex | 7.44 | < .001 | 0.71 | [0.52, 0.90] |
| Child Race (American Indian/Alaska Native) | -1.50 | .135 | -1.87 | [-4.32, 0.58] |
| Child Race (Asian) | 3.36 | .001 | 2.60 | [1.08, 4.11] |
| Child Race (Black) | -2.16 | .031 | -1.31 | [-2.50, -0.12] |
| Child Race (Native Hawaiian / Pacific Islander) | -1.02 | .306 | -2.53 | [-7.38, 2.32] |
| Child Race (Other) | 2.41 | .016 | 1.43 | [0.27, 2.59] |
| Child Ethnicity | 1.42 | .155 | 0.21 | [-0.08, 0.50] |
| Age | 18.01 | < .001 | 0.23 | [0.21, 0.26] |
| Mid-Posterior Corpus Callosum Volume | 3.89 | < .001 | 0.005 | [0.002, 0.01] |

**Note**: The linear mixed-effects model incorporates testing the statistical significance of coefficients against a *t*-distribution. Family Income was a categorical, effects-coded factor, in which the level “Mid” served as the reference level. Family Income was operationally defined as the self-reported combined family income and partitioned into three levels: Low Income: ≤ $50K; Mid Income: $50K-$100K; High Income: ≥ $100K. Sex was also a categorical factor, effect coded with Male/Female as ‑1/+1. Child Ethnicity was also a categorical factor, effect coded with Hispanic/Non-Hispanic as ‑1/+1. Child Race was also a categorical factor, in which “White” served as the reference level. Age, Maximum Parental Education (i.e., highest education level between parents/caregivers), and Mid-Posterior Corpus Callosum Volume were centered continuous factors. The random effects structure included a random intercept for study site identification number and family identification number. Random effects were restricted to be uncorrelated. Analysis included 8,524 data points. The model accounted for 21.7% of the variance in the data (*R*^2^ = .217, adjusted *R*^2^ = .216).

**Supplementary Table 77. Linear mixed-effects model output for the analysis of mid-posterior corpus callosum volume and scores on the flanker inhibitory control and attention test (NIH Toolbox).**

|  | *t*(8511) | *p* | *b* | 95% CI |
| --- | --- | --- | --- | --- |
| Intercept | 160.38 | < .001 | 93.86 | [92.71, 95.00] |
| Maximum Parental Education | 6.26 | < .001 | 0.67 | [0.46, 0.88] |
| Family Income (High) | 3.58 | < .001 | 0.57 | [0.26, 0.88] |
| Family Income (Low) | -4.50 | < .001 | -0.81 | [-1.16, -0.46] |
| Sex | -1.27 | .203 | -0.12 | [-0.30, 0.06] |
| Child Race (American Indian/Alaska Native) | -1.10 | .271 | -1.33 | [-3.70, 1.04] |
| Child Race (Asian) | 2.46 | .014 | 1.84 | [0.38, 3.31] |
| Child Race (Black) | -4.60 | < .001 | -2.70 | [-3.85, -1.55] |
| Child Race (Native Hawaiian / Pacific Islander) | 1.23 | .220 | 2.93 | [-1.75, 7.60] |
| Child Race (Other) | -0.61 | .544 | -0.35 | [-1.47, 0.77] |
| Child Ethnicity | 0.44 | .659 | 0.06 | [-0.22, 0.34] |
| Age | 17.32 | < .001 | 0.22 | [0.19, 0.24] |
| Mid-Posterior Corpus Callosum Volume | 3.50 | < .001 | 0.004 | [0.002, 0.01] |

**Note**: The linear mixed-effects model incorporates testing the statistical significance of coefficients against a *t*-distribution. Family Income was a categorical, effects-coded factor, in which the level “Mid” served as the reference level. Family Income was operationally defined as the self-reported combined family income and partitioned into three levels: Low Income: ≤ $50K; Mid Income: $50K-$100K; High Income: ≥ $100K. Sex was also a categorical factor, effect coded with Male/Female as ‑1/+1. Child Ethnicity was also a categorical factor, effect coded with Hispanic/Non-Hispanic as ‑1/+1. Child Race was also a categorical factor, in which “White” served as the reference level. Age, Maximum Parental Education (i.e., highest education level between parents/caregivers), and Mid-Posterior Corpus Callosum Volume were centered continuous factors. The random effects structure included a random intercept for study site identification number and family identification number. Random effects were restricted to be uncorrelated. Analysis included 8,524 data points. The model accounted for 16.2% of the variance in the data (*R*^2^ = .162, adjusted *R*^2^ = .161).

**Supplementary Table 78. Linear mixed-effects model output for the analysis of mid-posterior corpus callosum volume and scores on the list sorting working memory test (NIH Toolbox).**

|  | *t*(8511) | *p* | *b* | 95% CI |
| --- | --- | --- | --- | --- |
| Intercept | 122.94 | < .001 | 93.31 | [91.83, 94.80] |
| Maximum Parental Education | 13.09 | < .001 | 1.83 | [1.56, 2.11] |
| Family Income (High) | 5.29 | < .001 | 1.09 | [0.69, 1.50] |
| Family Income (Low) | -5.81 | < .001 | -1.36 | [-1.82, -0.90] |
| Sex | -2.88 | .004 | -0.35 | [-0.58, -0.11] |
| Child Race (American Indian/Alaska Native) | -0.38 | .701 | -0.61 | [-3.70, 2.49] |
| Child Race (Asian) | 0.97 | .330 | 0.95 | [-0.97, 2.87] |
| Child Race (Black) | -4.60 | < .001 | -3.54 | [-5.05, -2.03] |
| Child Race (Native Hawaiian / Pacific Islander) | 0.47 | .641 | 1.46 | [-4.69, 7.62] |
| Child Race (Other) | 0.61 | .543 | 0.46 | [-1.01, 1.93] |
| Child Ethnicity | 2.71 | .007 | 0.50 | [0.14, 0.87] |
| Age | 13.97 | < .001 | 0.23 | [0.19, 0.26] |
| Mid-Posterior Corpus Callosum Volume | 0.19 | .850 | 0.0003 | [-0.003, 0.003] |

**Note**: The linear mixed-effects model incorporates testing the statistical significance of coefficients against a *t*-distribution. Family Income was a categorical, effects-coded factor, in which the level “Mid” served as the reference level. Family Income was operationally defined as the self-reported combined family income and partitioned into three levels: Low Income: ≤ $50K; Mid Income: $50K-$100K; High Income: ≥ $100K. Sex was also a categorical factor, effect coded with Male/Female as ‑1/+1. Child Ethnicity was also a categorical factor, effect coded with Hispanic/Non-Hispanic as ‑1/+1. Child Race was also a categorical factor, in which “White” served as the reference level. Age, Maximum Parental Education (i.e., highest education level between parents/caregivers), and Mid-Posterior Corpus Callosum Volume were centered continuous factors. The random effects structure included a random intercept for study site identification number and family identification number. Random effects were restricted to be uncorrelated. Analysis included 8,524 data points. The model accounted for 27.1% of the variance in the data (*R*^2^ = .271, adjusted *R*^2^ = .270).

**Supplementary Table 79. Linear mixed-effects model output for the analysis of mid-posterior corpus callosum volume and scores on the pattern comparison processing speed test (NIH Toolbox).**

|  | *t*(8511) | *p* | *b* | 95% CI |
| --- | --- | --- | --- | --- |
| Intercept | 86.58 | < .001 | 87.81 | [85.82, 89.79] |
| Maximum Parental Education | 4.24 | < .001 | 0.75 | [0.40, 1.10] |
| Family Income (High) | 0.42 | .677 | 0.11 | [-0.40, 0.62] |
| Family Income (Low) | -1.45 | .148 | -0.43 | [-1.01, 0.15] |
| Sex | 6.44 | < .001 | 0.98 | [0.68, 1.28] |
| Child Race (American Indian/Alaska Native) | -1.88 | .060 | -3.74 | [-7.65, 0.16] |
| Child Race (Asian) | 2.44 | .015 | 3.03 | [0.60, 5.45] |
| Child Race (Black) | -2.54 | .011 | -2.47 | [-4.38, -0.57] |
| Child Race (Native Hawaiian / Pacific Islander) | 0.75 | .451 | 2.98 | [-4.76, 10.73] |
| Child Race (Other) | 0.07 | .943 | 0.07 | [-1.78, 1.92] |
| Child Ethnicity | 0.48 | .634 | 0.11 | [-0.36, 0.59] |
| Age | 20.58 | < .001 | 0.42 | [0.38, 0.46] |
| Mid-Posterior Corpus Callosum Volume | 3.21 | .001 | 0.01 | [0.003, 0.01] |

**Note**: The linear mixed-effects model incorporates testing the statistical significance of coefficients against a *t*-distribution. Family Income was a categorical, effects-coded factor, in which the level “Mid” served as the reference level. Family Income was operationally defined as the self-reported combined family income and partitioned into three levels: Low Income: ≤ $50K; Mid Income: $50K-$100K; High Income: ≥ $100K. Sex was also a categorical factor, effect coded with Male/Female as ‑1/+1. Child Ethnicity was also a categorical factor, effect coded with Hispanic/Non-Hispanic as ‑1/+1. Child Race was also a categorical factor, in which “White” served as the reference level. Age, Maximum Parental Education (i.e., highest education level between parents/caregivers), and Mid-Posterior Corpus Callosum Volume were centered continuous factors. The random effects structure included a random intercept for study site identification number and family identification number. Random effects were restricted to be uncorrelated. Analysis included 8,524 data points. The model accounted for 19.3% of the variance in the data (*R*^2^ = .193, adjusted *R*^2^ = .192).

**Supplementary Table 80. Linear mixed-effects model output for the analysis of mid-posterior corpus callosum volume and scores on the picture sequence memory test (NIH Toolbox).**

|  | *t*(8511) | *p* | *b* | 95% CI |
| --- | --- | --- | --- | --- |
| Intercept | 127.61 | < .001 | 101.37 | [99.82, 102.93] |
| Maximum Parental Education | 6.88 | < .001 | 1.01 | [0.73, 1.30] |
| Family Income (High) | 4.29 | < .001 | 0.93 | [0.50, 1.35] |
| Family Income (Low) | -3.48 | < .001 | -0.86 | [-1.34, -0.38] |
| Sex | 5.91 | < .001 | 0.75 | [0.50, 1.00] |
| Child Race (American Indian/Alaska Native) | 0.01 | .991 | 0.02 | [-3.24, 3.27] |
| Child Race (Asian) | 1.21 | .228 | 1.24 | [-0.78, 3.26] |
| Child Race (Black) | -5.15 | < .001 | -4.19 | [-5.78, -2.59] |
| Child Race (Native Hawaiian / Pacific Islander) | 0.46 | .643 | 1.54 | [-4.98, 8.06] |
| Child Race (Other) | 0.36 | .718 | 0.29 | [-1.27, 1.84] |
| Child Ethnicity | 0.28 | .780 | 0.05 | [-0.32, 0.43] |
| Age | 10.94 | < .001 | 0.18 | [0.15, 0.22] |
| Mid-Posterior Corpus Callosum Volume | -1.32 | .187 | -0.002 | [-0.01, 0.001] |

**Note**: The linear mixed-effects model incorporates testing the statistical significance of coefficients against a *t*-distribution. Family Income was a categorical, effects-coded factor, in which the level “Mid” served as the reference level. Family Income was operationally defined as the self-reported combined family income and partitioned into three levels: Low Income: ≤ $50K; Mid Income: $50K-$100K; High Income: ≥ $100K. Sex was also a categorical factor, effect coded with Male/Female as ‑1/+1. Child Ethnicity was also a categorical factor, effect coded with Hispanic/Non-Hispanic as ‑1/+1. Child Race was also a categorical factor, in which “White” served as the reference level. Age, Maximum Parental Education (i.e., highest education level between parents/caregivers), and Mid-Posterior Corpus Callosum Volume were centered continuous factors. The random effects structure included a random intercept for study site identification number and family identification number. Random effects were restricted to be uncorrelated. Analysis included 8,524 data points. The model accounted for 24.2% of the variance in the data (*R*^2^ = .242, adjusted *R*^2^ = .241).

**Supplementary Table 81. Linear mixed-effects model output for the analysis of mid-posterior corpus callosum volume and scores on the oral reading recognition test (NIH Toolbox).**

|  | *t*(8511) | *p* | *b* | 95% CI |
| --- | --- | --- | --- | --- |
| Intercept | 193.55 | < .001 | 89.22 | [88.32, 90.13] |
| Maximum Parental Education | 16.56 | < .001 | 1.30 | [1.15, 1.45] |
| Family Income (High) | 6.05 | < .001 | 0.70 | [0.47, 0.93] |
| Family Income (Low) | -8.15 | < .001 | -1.07 | [-1.33, -0.82] |
| Sex | 1.13 | .257 | 0.08 | [-0.06, 0.21] |
| Child Race (American Indian/Alaska Native) | -0.60 | .552 | -0.52 | [-2.25, 1.20] |
| Child Race (Asian) | 2.65 | .008 | 1.46 | [0.38, 2.55] |
| Child Race (Black) | -5.06 | < .001 | -2.22 | [-3.07, -1.36] |
| Child Race (Native Hawaiian / Pacific Islander) | 0.70 | .485 | 1.26 | [-2.27, 4.78] |
| Child Race (Other) | 0.11 | .915 | 0.05 | [-0.79, 0.88] |
| Child Ethnicity | 2.50 | .012 | 0.27 | [0.06, 0.48] |
| Age | 23.22 | < .001 | 0.20 | [0.19, 0.22] |
| Mid-Posterior Corpus Callosum Volume | 3.18 | .001 | 0.003 | [0.001, 0.004] |

**Note**: The linear mixed-effects model incorporates testing the statistical significance of coefficients against a *t*-distribution. Family Income was a categorical, effects-coded factor, in which the level “Mid” served as the reference level. Family Income was operationally defined as the self-reported combined family income and partitioned into three levels: Low Income: ≤ $50K; Mid Income: $50K-$100K; High Income: ≥ $100K. Sex was also a categorical factor, effect coded with Male/Female as ‑1/+1. Child Ethnicity was also a categorical factor, effect coded with Hispanic/Non-Hispanic as ‑1/+1. Child Race was also a categorical factor, in which “White” served as the reference level. Age, Maximum Parental Education (i.e., highest education level between parents/caregivers), and Mid-Posterior Corpus Callosum Volume were centered continuous factors. The random effects structure included a random intercept for study site identification number and family identification number. Random effects were restricted to be uncorrelated. Analysis included 8,524 data points. The model accounted for 52.8% of the variance in the data (*R*^2^ = .528, adjusted *R*^2^ = .528).

**Supplementary Table 82. Linear mixed-effects model output for the analysis of mid-posterior corpus callosum volume and scores on the picture vocabulary test (NIH Toolbox).**

|  | *t*(8511) | *p* | *b* | 95% CI |
| --- | --- | --- | --- | --- |
| Intercept | 161.80 | < .001 | 79.55 | [78.58, 80.51] |
| Maximum Parental Education | 18.91 | < .001 | 1.64 | [1.47, 1.81] |
| Family Income (High) | 6.91 | < .001 | 0.88 | [0.63, 1.13] |
| Family Income (Low) | -8.25 | < .001 | -1.20 | [-1.48, -0.91] |
| Sex | -1.53 | .126 | -0.11 | [-0.26, 0.03] |
| Child Race (American Indian/Alaska Native) | -0.55 | .583 | -0.53 | [-2.44, 1.37] |
| Child Race (Asian) | 2.94 | .003 | 1.79 | [0.59, 2.98] |
| Child Race (Black) | -3.49 | < .001 | -1.68 | [-2.62, -0.74] |
| Child Race (Native Hawaiian / Pacific Islander) | -2.39 | .017 | -4.70 | [-8.56, -0.84] |
| Child Race (Other) | 4.72 | < .001 | 2.21 | [1.29, 3.13] |
| Child Ethnicity | 8.70 | < .001 | 1.02 | [0.79, 1.25] |
| Age | 27.10 | < .001 | 0.27 | [0.25, 0.28] |
| Mid-Posterior Corpus Callosum Volume | 5.19 | < .001 | 0.01 | [0.003, 0.01] |

**Note**: The linear mixed-effects model incorporates testing the statistical significance of coefficients against a *t*-distribution. Family Income was a categorical, effects-coded factor, in which the level “Mid” served as the reference level. Family Income was operationally defined as the self-reported combined family income and partitioned into three levels: Low Income: ≤ $50K; Mid Income: $50K-$100K; High Income: ≥ $100K. Sex was also a categorical factor, effect coded with Male/Female as ‑1/+1. Child Ethnicity was also a categorical factor, effect coded with Hispanic/Non-Hispanic as ‑1/+1. Child Race was also a categorical factor, in which “White” served as the reference level. Age, Maximum Parental Education (i.e., highest education level between parents/caregivers), and Mid-Posterior Corpus Callosum Volume were centered continuous factors. The random effects structure included a random intercept for study site identification number and family identification number. Random effects were restricted to be uncorrelated. Analysis included 8,524 data points. The model accounted for 56.2% of the variance in the data (*R*^2^ = .562, adjusted *R*^2^ = .562).

**Supplementary Table 83. Linear mixed-effects model output for the analysis of central corpus callosum volume and scores on the dimensional change card sort test (NIH Toolbox).**

|  | *t*(8511) | *p* | *b* | 95% CI |
| --- | --- | --- | --- | --- |
| Intercept | 150.24 | < .001 | 90.22 | [89.05, 91.40] |
| Maximum Parental Education | 8.07 | < .001 | 0.89 | [0.68, 1.11] |
| Family Income (High) | 3.31 | .001 | 0.54 | [0.22, 0.86] |
| Family Income (Low) | -4.97 | < .001 | -0.92 | [-1.29, -0.56] |
| Sex | 7.26 | < .001 | 0.69 | [0.51, 0.88] |
| Child Race (American Indian/Alaska Native) | -1.46 | .145 | -1.82 | [-4.27, 0.63] |
| Child Race (Asian) | 3.28 | .001 | 2.54 | [1.03, 4.06] |
| Child Race (Black) | -2.26 | .024 | -1.38 | [-2.57, -0.18] |
| Child Race (Native Hawaiian / Pacific Islander) | -1.01 | .311 | -2.51 | [-7.37, 2.34] |
| Child Race (Other) | 2.43 | .015 | 1.44 | [0.28, 2.60] |
| Child Ethnicity | 1.43 | .152 | 0.21 | [-0.08, 0.50] |
| Age | 18.22 | < .001 | 0.23 | [0.21, 0.26] |
| Central Corpus Callosum Volume | 2.53 | .011 | 0.003 | [0.001, 0.01] |

**Note**: The linear mixed-effects model incorporates testing the statistical significance of coefficients against a *t*-distribution. Family Income was a categorical, effects-coded factor, in which the level “Mid” served as the reference level. Family Income was operationally defined as the self-reported combined family income and partitioned into three levels: Low Income: ≤ $50K; Mid Income: $50K-$100K; High Income: ≥ $100K. Sex was also a categorical factor, effect coded with Male/Female as ‑1/+1. Child Ethnicity was also a categorical factor, effect coded with Hispanic/Non-Hispanic as ‑1/+1. Child Race was also a categorical factor, in which “White” served as the reference level. Age, Maximum Parental Education (i.e., highest education level between parents/caregivers), and Central Corpus Callosum Volume were centered continuous factors. The random effects structure included a random intercept for study site identification number and family identification number. Random effects were restricted to be uncorrelated. Analysis included 8,524 data points. The model accounted for 21.6% of the variance in the data (*R*^2^ = .216, adjusted *R*^2^ = .214).

**Supplementary Table 84. Linear mixed-effects model output for the analysis of central corpus callosum volume and scores on the flanker inhibitory control and attention test (NIH Toolbox).**

|  | *t*(8511) | *p* | *b* | 95% CI |
| --- | --- | --- | --- | --- |
| Intercept | 160.47 | < .001 | 93.81 | [92.67, 94.96] |
| Maximum Parental Education | 6.33 | < .001 | 0.68 | [0.47, 0.89] |
| Family Income (High) | 3.59 | < .001 | 0.57 | [0.26, 0.88] |
| Family Income (Low) | -4.54 | < .001 | -0.82 | [-1.17, -0.47] |
| Sex | -1.51 | .132 | -0.14 | [-0.32, 0.04] |
| Child Race (American Indian/Alaska Native) | -1.06 | .288 | -1.29 | [-3.66, 1.09] |
| Child Race (Asian) | 2.41 | .016 | 1.81 | [0.34, 3.28] |
| Child Race (Black) | -4.70 | < .001 | -2.76 | [-3.91, -1.61] |
| Child Race (Native Hawaiian / Pacific Islander) | 1.22 | .223 | 2.91 | [-1.77, 7.59] |
| Child Race (Other) | -0.57 | .570 | -0.32 | [-1.45, 0.80] |
| Child Ethnicity | 0.48 | .631 | 0.07 | [-0.21, 0.35] |
| Age | 17.58 | < .001 | 0.22 | [0.19, 0.24] |
| Central Corpus Callosum Volume | 1.27 | .206 | 0.001 | [-0.001, 0.004] |

**Note**: The linear mixed-effects model incorporates testing the statistical significance of coefficients against a *t*-distribution. Family Income was a categorical, effects-coded factor, in which the level “Mid” served as the reference level. Family Income was operationally defined as the self-reported combined family income and partitioned into three levels: Low Income: ≤ $50K; Mid Income: $50K-$100K; High Income: ≥ $100K. Sex was also a categorical factor, effect coded with Male/Female as ‑1/+1. Child Ethnicity was also a categorical factor, effect coded with Hispanic/Non-Hispanic as ‑1/+1. Child Race was also a categorical factor, in which “White” served as the reference level. Age, Maximum Parental Education (i.e., highest education level between parents/caregivers), and Central Corpus Callosum Volume were centered continuous factors. The random effects structure included a random intercept for study site identification number and family identification number. Random effects were restricted to be uncorrelated. Analysis included 8,524 data points. The model accounted for 16.1% of the variance in the data (*R*^2^ = .161, adjusted *R*^2^ = .160).

**Supplementary Table 85. Linear mixed-effects model output for the analysis of central corpus callosum volume and scores on the list sorting working memory test (NIH Toolbox).**

|  | *t*(8511) | *p* | *b* | 95% CI |
| --- | --- | --- | --- | --- |
| Intercept | 122.94 | < .001 | 93.32 | [91.83, 94.81] |
| Maximum Parental Education | 13.10 | < .001 | 1.83 | [1.56, 2.11] |
| Family Income (High) | 5.29 | < .001 | 1.09 | [0.69, 1.50] |
| Family Income (Low) | -5.80 | < .001 | -1.36 | [-1.82, -0.90] |
| Sex | -2.86 | .004 | -0.34 | [-0.58, -0.11] |
| Child Race (American Indian/Alaska Native) | -0.38 | .701 | -0.61 | [-3.70, 2.49] |
| Child Race (Asian) | 0.96 | .335 | 0.94 | [-0.97, 2.86] |
| Child Race (Black) | -4.60 | < .001 | -3.54 | [-5.05, -2.03] |
| Child Race (Native Hawaiian / Pacific Islander) | 0.47 | .636 | 1.49 | [-4.67, 7.65] |
| Child Race (Other) | 0.60 | .550 | 0.45 | [-1.02, 1.92] |
| Child Ethnicity | 2.69 | .007 | 0.50 | [0.13, 0.86] |
| Age | 13.97 | < .001 | 0.23 | [0.19, 0.26] |
| Central Corpus Callosum Volume | 0.66 | .512 | 0.001 | [-0.002, 0.004] |

**Note**: The linear mixed-effects model incorporates testing the statistical significance of coefficients against a *t*-distribution. Family Income was a categorical, effects-coded factor, in which the level “Mid” served as the reference level. Family Income was operationally defined as the self-reported combined family income and partitioned into three levels: Low Income: ≤ $50K; Mid Income: $50K-$100K; High Income: ≥ $100K. Sex was also a categorical factor, effect coded with Male/Female as ‑1/+1. Child Ethnicity was also a categorical factor, effect coded with Hispanic/Non-Hispanic as ‑1/+1. Child Race was also a categorical factor, in which “White” served as the reference level. Age, Maximum Parental Education (i.e., highest education level between parents/caregivers), and Central Corpus Callosum Volume were centered continuous factors. The random effects structure included a random intercept for study site identification number and family identification number. Random effects were restricted to be uncorrelated. Analysis included 8,524 data points. The model accounted for 27.1% of the variance in the data (*R*^2^ = .271, adjusted *R*^2^ = .270).

**Supplementary Table 86. Linear mixed-effects model output for the analysis of central corpus callosum volume and scores on the pattern comparison processing speed test (NIH Toolbox).**

|  | *t*(8511) | *p* | *b* | 95% CI |
| --- | --- | --- | --- | --- |
| Intercept | 86.73 | < .001 | 87.74 | [85.76, 89.72] |
| Maximum Parental Education | 4.31 | < .001 | 0.76 | [0.42, 1.11] |
| Family Income (High) | 0.43 | .668 | 0.11 | [-0.40, 0.62] |
| Family Income (Low) | -1.49 | .136 | -0.44 | [-1.02, 0.14] |
| Sex | 6.24 | < .001 | 0.95 | [0.65, 1.25] |
| Child Race (American Indian/Alaska Native) | -1.85 | .065 | -3.68 | [-7.59, 0.23] |
| Child Race (Asian) | 2.40 | .016 | 2.97 | [0.55, 5.40] |
| Child Race (Black) | -2.63 | .008 | -2.56 | [-4.47, -0.66] |
| Child Race (Native Hawaiian / Pacific Islander) | 0.75 | .453 | 2.97 | [-4.79, 10.72] |
| Child Race (Other) | 0.10 | .918 | 0.10 | [-1.76, 1.95] |
| Child Ethnicity | 0.50 | .615 | 0.12 | [-0.35, 0.59] |
| Age | 20.82 | < .001 | 0.42 | [0.38, 0.46] |
| Central Corpus Callosum Volume | 1.24 | .216 | 0.002 | [-0.001, 0.01] |

**Note**: The linear mixed-effects model incorporates testing the statistical significance of coefficients against a *t*-distribution. Family Income was a categorical, effects-coded factor, in which the level “Mid” served as the reference level. Family Income was operationally defined as the self-reported combined family income and partitioned into three levels: Low Income: ≤ $50K; Mid Income: $50K-$100K; High Income: ≥ $100K. Sex was also a categorical factor, effect coded with Male/Female as ‑1/+1. Child Ethnicity was also a categorical factor, effect coded with Hispanic/Non-Hispanic as ‑1/+1. Child Race was also a categorical factor, in which “White” served as the reference level. Age, Maximum Parental Education (i.e., highest education level between parents/caregivers), and Central Corpus Callosum Volume were centered continuous factors. The random effects structure included a random intercept for study site identification number and family identification number. Random effects were restricted to be uncorrelated. Analysis included 8,524 data points. The model accounted for 19.3% of the variance in the data (*R*^2^ = .193, adjusted *R*^2^ = .192).

**Supplementary Table 87. Linear mixed-effects model output for the analysis of central corpus callosum volume and scores on the picture sequence memory test (NIH Toolbox).**

|  | *t*(8511) | *p* | *b* | 95% CI |
| --- | --- | --- | --- | --- |
| Intercept | 127.60 | < .001 | 101.40 | [99.84, 102.96] |
| Maximum Parental Education | 6.85 | < .001 | 1.01 | [0.72, 1.30] |
| Family Income (High) | 4.28 | < .001 | 0.93 | [0.50, 1.35] |
| Family Income (Low) | -3.46 | .001 | -0.85 | [-1.34, -0.37] |
| Sex | 6.02 | < .001 | 0.76 | [0.51, 1.01] |
| Child Race (American Indian/Alaska Native) | 0.00 | .998 | -0.004 | [-3.26, 3.25] |
| Child Race (Asian) | 1.23 | .220 | 1.26 | [-0.76, 3.28] |
| Child Race (Black) | -5.12 | < .001 | -4.16 | [-5.75, -2.56] |
| Child Race (Native Hawaiian / Pacific Islander) | 0.47 | .641 | 1.55 | [-4.97, 8.08] |
| Child Race (Other) | 0.34 | .732 | 0.27 | [-1.28, 1.83] |
| Child Ethnicity | 0.25 | .800 | 0.05 | [-0.33, 0.43] |
| Age | 10.86 | < .001 | 0.18 | [0.15, 0.22] |
| Central Corpus Callosum Volume | -0.31 | .760 | -0.0005 | [-0.003, 0.002] |

**Note**: The linear mixed-effects model incorporates testing the statistical significance of coefficients against a *t*-distribution. Family Income was a categorical, effects-coded factor, in which the level “Mid” served as the reference level. Family Income was operationally defined as the self-reported combined family income and partitioned into three levels: Low Income: ≤ $50K; Mid Income: $50K-$100K; High Income: ≥ $100K. Sex was also a categorical factor, effect coded with Male/Female as ‑1/+1. Child Ethnicity was also a categorical factor, effect coded with Hispanic/Non-Hispanic as ‑1/+1. Child Race was also a categorical factor, in which “White” served as the reference level. Age, Maximum Parental Education (i.e., highest education level between parents/caregivers), and Central Corpus Callosum Volume were centered continuous factors. The random effects structure included a random intercept for study site identification number and family identification number. Random effects were restricted to be uncorrelated. Analysis included 8,524 data points. The model accounted for 24.2% of the variance in the data (*R*^2^ = .242, adjusted *R*^2^ = .241).

**Supplementary Table 88. Linear mixed-effects model output for the analysis of central corpus callosum volume and scores on the oral reading recognition test (NIH Toolbox).**

|  | *t*(8511) | *p* | *b* | 95% CI |
| --- | --- | --- | --- | --- |
| Intercept | 193.63 | < .001 | 89.20 | [88.30, 90.10] |
| Maximum Parental Education | 16.62 | < .001 | 1.31 | [1.15, 1.46] |
| Family Income (High) | 6.05 | < .001 | 0.70 | [0.47, 0.93] |
| Family Income (Low) | -8.17 | < .001 | -1.08 | [-1.34, -0.82] |
| Sex | 0.96 | .338 | 0.06 | [-0.07, 0.19] |
| Child Race (American Indian/Alaska Native) | -0.56 | .573 | -0.50 | [-2.23, 1.23] |
| Child Race (Asian) | 2.60 | .009 | 1.43 | [0.35, 2.52] |
| Child Race (Black) | -5.14 | < .001 | -2.25 | [-3.11, -1.39] |
| Child Race (Native Hawaiian / Pacific Islander) | 0.70 | .485 | 1.26 | [-2.27, 4.78] |
| Child Race (Other) | 0.13 | .896 | 0.06 | [-0.78, 0.89] |
| Child Ethnicity | 2.52 | .012 | 0.27 | [0.06, 0.48] |
| Age | 23.44 | < .001 | 0.21 | [0.19, 0.22] |
| Central Corpus Callosum Volume | 1.68 | .094 | 0.001 | [-0.0002, 0.003] |

**Note**: The linear mixed-effects model incorporates testing the statistical significance of coefficients against a *t*-distribution. Family Income was a categorical, effects-coded factor, in which the level “Mid” served as the reference level. Family Income was operationally defined as the self-reported combined family income and partitioned into three levels: Low Income: ≤ $50K; Mid Income: $50K-$100K; High Income: ≥ $100K. Sex was also a categorical factor, effect coded with Male/Female as ‑1/+1. Child Ethnicity was also a categorical factor, effect coded with Hispanic/Non-Hispanic as ‑1/+1. Child Race was also a categorical factor, in which “White” served as the reference level. Age, Maximum Parental Education (i.e., highest education level between parents/caregivers), and Central Corpus Callosum Volume were centered continuous factors. The random effects structure included a random intercept for study site identification number and family identification number. Random effects were restricted to be uncorrelated. Analysis included 8,524 data points. The model accounted for 52.7% of the variance in the data (*R*^2^ = .527, adjusted *R*^2^ = .527).

**Supplementary Table 89. Linear mixed-effects model output for the analysis of central corpus callosum volume and scores on the picture vocabulary test (NIH Toolbox).**

|  | *t*(8511) | *p* | *b* | 95% CI |
| --- | --- | --- | --- | --- |
| Intercept | 161.51 | < .001 | 79.50 | [78.54, 80.47] |
| Maximum Parental Education | 18.99 | < .001 | 1.65 | [1.48, 1.82] |
| Family Income (High) | 6.92 | < .001 | 0.88 | [0.63, 1.14] |
| Family Income (Low) | -8.29 | < .001 | -1.20 | [-1.49, -0.92] |
| Sex | -1.80 | .072 | -0.13 | [-0.28, 0.01] |
| Child Race (American Indian/Alaska Native) | -0.50 | .619 | -0.48 | [-2.39, 1.42] |
| Child Race (Asian) | 2.84 | .004 | 1.73 | [0.54, 2.92] |
| Child Race (Black) | -3.62 | < .001 | -1.74 | [-2.68, -0.80] |
| Child Race (Native Hawaiian / Pacific Islander) | -2.38 | .017 | -4.69 | [-8.56, -0.82] |
| Child Race (Other) | 4.75 | < .001 | 2.22 | [1.31, 3.14] |
| Child Ethnicity | 8.71 | < .001 | 1.02 | [0.79, 1.25] |
| Age | 27.42 | < .001 | 0.27 | [0.25, 0.29] |
| Central Corpus Callosum Volume | 3.05 | .002 | 0.003 | [0.001, 0.004] |

**Note**: The linear mixed-effects model incorporates testing the statistical significance of coefficients against a *t*-distribution. Family Income was a categorical, effects-coded factor, in which the level “Mid” served as the reference level. Family Income was operationally defined as the self-reported combined family income and partitioned into three levels: Low Income: ≤ $50K; Mid Income: $50K-$100K; High Income: ≥ $100K. Sex was also a categorical factor, effect coded with Male/Female as ‑1/+1. Child Ethnicity was also a categorical factor, effect coded with Hispanic/Non-Hispanic as ‑1/+1. Child Race was also a categorical factor, in which “White” served as the reference level. Age, Maximum Parental Education (i.e., highest education level between parents/caregivers), and Central Corpus Callosum Volume were centered continuous factors. The random effects structure included a random intercept for study site identification number and family identification number. Random effects were restricted to be uncorrelated. Analysis included 8,524 data points. The model accounted for 56.3% of the variance in the data (*R*^2^ = .563, adjusted *R*^2^ = .562).

**Supplementary Table 90. Linear mixed-effects model output for the analysis of mid-anterior corpus callosum volume and scores on the dimensional change card sort test (NIH Toolbox).**

|  | *t*(8511) | *p* | *b* | 95% CI |
| --- | --- | --- | --- | --- |
| Intercept | 150.51 | < .001 | 90.21 | [89.04, 91.39] |
| Maximum Parental Education | 8.10 | < .001 | 0.90 | [0.68, 1.12] |
| Family Income (High) | 3.32 | .001 | 0.54 | [0.22, 0.86] |
| Family Income (Low) | -5.00 | < .001 | -0.93 | [-1.29, -0.56] |
| Sex | 7.31 | < .001 | 0.70 | [0.52, 0.89] |
| Child Race (American Indian/Alaska Native) | -1.46 | .144 | -1.83 | [-4.28, 0.62] |
| Child Race (Asian) | 3.28 | .001 | 2.54 | [1.02, 4.06] |
| Child Race (Black) | -2.23 | .026 | -1.35 | [-2.54, -0.16] |
| Child Race (Native Hawaiian / Pacific Islander) | -1.02 | .306 | -2.53 | [-7.39, 2.32] |
| Child Race (Other) | 2.44 | .015 | 1.45 | [0.29, 2.61] |
| Child Ethnicity | 1.44 | .150 | 0.21 | [-0.08, 0.50] |
| Age | 18.40 | < .001 | 0.23 | [0.21, 0.26] |
| Mid-Anterior Corpus Callosum Volume | 1.90 | .058 | 0.002 | [-0.0001, 0.004] |

**Note**: The linear mixed-effects model incorporates testing the statistical significance of coefficients against a *t*-distribution. Family Income was a categorical, effects-coded factor, in which the level “Mid” served as the reference level. Family Income was operationally defined as the self-reported combined family income and partitioned into three levels: Low Income: ≤ $50K; Mid Income: $50K-$100K; High Income: ≥ $100K. Sex was also a categorical factor, effect coded with Male/Female as ‑1/+1. Child Ethnicity was also a categorical factor, effect coded with Hispanic/Non-Hispanic as ‑1/+1. Child Race was also a categorical factor, in which “White” served as the reference level. Age, Maximum Parental Education (i.e., highest education level between parents/caregivers), and Mid-Anterior Corpus Callosum Volume were centered continuous factors. The random effects structure included a random intercept for study site identification number and family identification number. Random effects were restricted to be uncorrelated. Analysis included 8,524 data points. The model accounted for 21.5% of the variance in the data (*R*^2^ = .215, adjusted *R*^2^ = .214).

**Supplementary Table 91. Linear mixed-effects model output for the analysis of mid-anterior corpus callosum volume and scores on the flanker inhibitory control and attention test (NIH Toolbox).**

|  | *t*(8511) | *p* | *b* | 95% CI |
| --- | --- | --- | --- | --- |
| Intercept | 160.54 | < .001 | 93.81 | [92.66, 94.95] |
| Maximum Parental Education | 6.35 | < .001 | 0.68 | [0.47, 0.89] |
| Family Income (High) | 3.59 | < .001 | 0.57 | [0.26, 0.88] |
| Family Income (Low) | -4.55 | < .001 | -0.82 | [-1.17, -0.47] |
| Sex | -1.42 | .157 | -0.13 | [-0.32, 0.05] |
| Child Race (American Indian/Alaska Native) | -1.06 | .287 | -1.29 | [-3.66, 1.08] |
| Child Race (Asian) | 2.40 | .016 | 1.8 | [0.33, 3.27] |
| Child Race (Black) | -4.67 | < .001 | -2.74 | [-3.89, -1.59] |
| Child Race (Native Hawaiian / Pacific Islander) | 1.22 | .224 | 2.90 | [-1.77, 7.58] |
| Child Race (Other) | -0.56 | .572 | -0.32 | [-1.44, 0.80] |
| Child Ethnicity | 0.48 | .629 | 0.07 | [-0.21, 0.35] |
| Age | 17.68 | < .001 | 0.22 | [0.20, 0.24] |
| Mid-Anterior Corpus Callosum Volume | 1.14 | .253 | 0.001 | [-0.001, 0.003] |

**Note**: The linear mixed-effects model incorporates testing the statistical significance of coefficients against a *t*-distribution. Family Income was a categorical, effects-coded factor, in which the level “Mid” served as the reference level. Family Income was operationally defined as the self-reported combined family income and partitioned into three levels: Low Income: ≤ $50K; Mid Income: $50K-$100K; High Income: ≥ $100K. Sex was also a categorical factor, effect coded with Male/Female as ‑1/+1. Child Ethnicity was also a categorical factor, effect coded with Hispanic/Non-Hispanic as ‑1/+1. Child Race was also a categorical factor, in which “White” served as the reference level. Age, Maximum Parental Education (i.e., highest education level between parents/caregivers), and Mid-Anterior Corpus Callosum Volume were centered continuous factors. The random effects structure included a random intercept for study site identification number and family identification number. Random effects were restricted to be uncorrelated. Analysis included 8,524 data points. The model accounted for 16.1% of the variance in the data (*R*^2^ = .161, adjusted *R*^2^ = .160).

**Supplementary Table 92. Linear mixed-effects model output for the analysis of mid-anterior corpus callosum volume and scores on the list sorting working memory test (NIH Toolbox).**

|  | *t*(8511) | *p* | *b* | 95% CI |
| --- | --- | --- | --- | --- |
| Intercept | 123.00 | < .001 | 93.30 | [91.81, 94.78] |
| Maximum Parental Education | 13.10 | < .001 | 1.83 | [1.56, 2.11] |
| Family Income (High) | 5.29 | < .001 | 1.09 | [0.69, 1.50] |
| Family Income (Low) | -5.82 | < .001 | -1.37 | [-1.83, -0.91] |
| Sex | -2.99 | .003 | -0.36 | [-0.60, -0.13] |
| Child Race (American Indian/Alaska Native) | -0.38 | .705 | -0.60 | [-3.69, 2.50] |
| Child Race (Asian) | 0.99 | .323 | 0.97 | [-0.95, 2.89] |
| Child Race (Black) | -4.63 | < .001 | -3.56 | [-5.07, -2.06] |
| Child Race (Native Hawaiian / Pacific Islander) | 0.46 | .648 | 1.43 | [-4.73, 7.59] |
| Child Race (Other) | 0.63 | .529 | 0.47 | [-1.00, 1.94] |
| Child Ethnicity | 2.74 | .006 | 0.51 | [0.15, 0.87] |
| Age | 14.06 | < .001 | 0.23 | [0.19, 0.26] |
| Mid-Anterior Corpus Callosum Volume | -0.82 | .411 | -0.001 | [-0.003, 0.001] |

**Note**: The linear mixed-effects model incorporates testing the statistical significance of coefficients against a *t*-distribution. Family Income was a categorical, effects-coded factor, in which the level “Mid” served as the reference level. Family Income was operationally defined as the self-reported combined family income and partitioned into three levels: Low Income: ≤ $50K; Mid Income: $50K-$100K; High Income: ≥ $100K. Sex was also a categorical factor, effect coded with Male/Female as ‑1/+1. Child Ethnicity was also a categorical factor, effect coded with Hispanic/Non-Hispanic as ‑1/+1. Child Race was also a categorical factor, in which “White” served as the reference level. Age, Maximum Parental Education (i.e., highest education level between parents/caregivers), and Mid-Anterior Corpus Callosum Volume were centered continuous factors. The random effects structure included a random intercept for study site identification number and family identification number. Random effects were restricted to be uncorrelated. Analysis included 8,524 data points. The model accounted for 27.1% of the variance in the data (*R*^2^ = .271, adjusted *R*^2^ = .270).

**Supplementary Table 93. Linear mixed-effects model output for the analysis of mid-anterior corpus callosum volume and scores on the pattern comparison processing speed test (NIH Toolbox).**

|  | *t*(8511) | *p* | *b* | 95% CI |
| --- | --- | --- | --- | --- |
| Intercept | 86.76 | < .001 | 87.75 | [85.77, 89.73] |
| Maximum Parental Education | 4.33 | < .001 | 0.77 | [0.42, 1.11] |
| Family Income (High) | 0.43 | .670 | 0.11 | [-0.40, 0.62] |
| Family Income (Low) | -1.48 | .138 | -0.44 | [-1.02, 0.14] |
| Sex | 6.40 | < .001 | 0.98 | [0.68, 1.28] |
| Child Race (American Indian/Alaska Native) | -1.85 | .064 | -3.69 | [-7.60, 0.21] |
| Child Race (Asian) | 2.37 | .018 | 2.94 | [0.51, 5.37] |
| Child Race (Black) | -2.58 | .010 | -2.51 | [-4.41, -0.60] |
| Child Race (Native Hawaiian / Pacific Islander) | 0.76 | .449 | 3.00 | [-4.76, 10.75] |
| Child Race (Other) | 0.09 | .931 | 0.08 | [-1.77, 1.93] |
| Child Ethnicity | 0.48 | .629 | 0.12 | [-0.36, 0.59] |
| Age | 20.91 | < .001 | 0.43 | [0.39, 0.47] |
| Mid-Anterior Corpus Callosum Volume | 2.03 | .042 | 0.003 | [0.0001, 0.01] |

**Note**: The linear mixed-effects model incorporates testing the statistical significance of coefficients against a *t*-distribution. Family Income was a categorical, effects-coded factor, in which the level “Mid” served as the reference level. Family Income was operationally defined as the self-reported combined family income and partitioned into three levels: Low Income: ≤ $50K; Mid Income: $50K-$100K; High Income: ≥ $100K. Sex was also a categorical factor, effect coded with Male/Female as ‑1/+1. Child Ethnicity was also a categorical factor, effect coded with Hispanic/Non-Hispanic as ‑1/+1. Child Race was also a categorical factor, in which “White” served as the reference level. Age, Maximum Parental Education (i.e., highest education level between parents/caregivers), and Mid-Anterior Corpus Callosum Volume were centered continuous factors. The random effects structure included a random intercept for study site identification number and family identification number. Random effects were restricted to be uncorrelated. Analysis included 8,524 data points. The model accounted for 19.3% of the variance in the data (*R*^2^ = .193, adjusted *R*^2^ = .192).

**Supplementary Table 94. Linear mixed-effects model output for the analysis of mid-anterior corpus callosum volume and scores on the picture sequence memory test (NIH Toolbox).**

|  | *t*(8511) | *p* | *b* | 95% CI |
| --- | --- | --- | --- | --- |
| Intercept | 127.63 | < .001 | 101.38 | [99.82, 102.94] |
| Maximum Parental Education | 6.85 | < .001 | 1.01 | [0.72, 1.30] |
| Family Income (High) | 4.28 | < .001 | 0.93 | [0.50, 1.35] |
| Family Income (Low) | -3.48 | .001 | -0.86 | [-1.34, -0.37] |
| Sex | 5.78 | < .001 | 0.74 | [0.49, 0.99] |
| Child Race (American Indian/Alaska Native) | 0.00 | .997 | 0.01 | [-3.25, 3.26] |
| Child Race (Asian) | 1.25 | .211 | 1.29 | [-0.73, 3.31] |
| Child Race (Black) | -5.16 | < .001 | -4.19 | [-5.78, -2.60] |
| Child Race (Native Hawaiian / Pacific Islander) | 0.45 | .650 | 1.51 | [-5.01, 8.03] |
| Child Race (Other) | 0.37 | .712 | 0.29 | [-1.26, 1.85] |
| Child Ethnicity | 0.30 | .766 | 0.06 | [-0.32, 0.44] |
| Age | 10.89 | < .001 | 0.18 | [0.15, 0.22] |
| Mid-Anterior Corpus Callosum Volume | -1.54 | .125 | -0.002 | [-0.005, 0.001] |

**Note**: The linear mixed-effects model incorporates testing the statistical significance of coefficients against a *t*-distribution. Family Income was a categorical, effects-coded factor, in which the level “Mid” served as the reference level. Family Income was operationally defined as the self-reported combined family income and partitioned into three levels: Low Income: ≤ $50K; Mid Income: $50K-$100K; High Income: ≥ $100K. Sex was also a categorical factor, effect coded with Male/Female as ‑1/+1. Child Ethnicity was also a categorical factor, effect coded with Hispanic/Non-Hispanic as ‑1/+1. Child Race was also a categorical factor, in which “White” served as the reference level. Age, Maximum Parental Education (i.e., highest education level between parents/caregivers), and Mid-Anterior Corpus Callosum Volume were centered continuous factors. The random effects structure included a random intercept for study site identification number and family identification number. Random effects were restricted to be uncorrelated. Analysis included 8,524 data points. The model accounted for 24.2% of the variance in the data (*R*^2^ = .242, adjusted *R*^2^ = .241).

**Supplementary Table 95. Linear mixed-effects model output for the analysis of mid-anterior corpus callosum volume and scores on the oral reading recognition test (NIH Toolbox).**

|  | *t*(8511) | *p* | *b* | 95% CI |
| --- | --- | --- | --- | --- |
| Intercept | 193.83 | < .001 | 89.19 | [88.28, 90.09] |
| Maximum Parental Education | 16.64 | < .001 | 1.31 | [1.15, 1.46] |
| Family Income (High) | 6.05 | < .001 | 0.70 | [0.47, 0.93] |
| Family Income (Low) | -8.19 | < .001 | -1.08 | [-1.34, -0.82] |
| Sex | 0.90 | .367 | 0.06 | [-0.07, 0.19] |
| Child Race (American Indian/Alaska Native) | -0.56 | .574 | -0.50 | [-2.22, 1.23] |
| Child Race (Asian) | 2.61 | .009 | 1.44 | [0.36, 2.53] |
| Child Race (Black) | -5.13 | < .001 | -2.25 | [-3.11, -1.39] |
| Child Race (Native Hawaiian / Pacific Islander) | 0.68 | .496 | 1.23 | [-2.30, 4.75] |
| Child Race (Other) | 0.16 | .875 | 0.07 | [-0.77, 0.90] |
| Child Ethnicity | 2.56 | .011 | 0.27 | [0.06, 0.48] |
| Age | 23.59 | < .001 | 0.21 | [0.19, 0.22] |
| Mid-Anterior Corpus Callosum Volume | 0.39 | .698 | 0.0003 | [-0.001, 0.002] |

**Note**: The linear mixed-effects model incorporates testing the statistical significance of coefficients against a *t*-distribution. Family Income was a categorical, effects-coded factor, in which the level “Mid” served as the reference level. Family Income was operationally defined as the self-reported combined family income and partitioned into three levels: Low Income: ≤ $50K; Mid Income: $50K-$100K; High Income: ≥ $100K. Sex was also a categorical factor, effect coded with Male/Female as ‑1/+1. Child Ethnicity was also a categorical factor, effect coded with Hispanic/Non-Hispanic as ‑1/+1. Child Race was also a categorical factor, in which “White” served as the reference level. Age, Maximum Parental Education (i.e., highest education level between parents/caregivers), and Mid-Anterior Corpus Callosum Volume were centered continuous factors. The random effects structure included a random intercept for study site identification number and family identification number. Random effects were restricted to be uncorrelated. Analysis included 8,524 data points. The model accounted for 52.7% of the variance in the data (*R*^2^ = .527, adjusted *R*^2^ = .526).

**Supplementary Table 96. Linear mixed-effects model output for the analysis of mid-anterior corpus callosum volume and scores on the picture vocabulary test (NIH Toolbox).**

|  | *t*(8511) | *p* | *b* | 95% CI |
| --- | --- | --- | --- | --- |
| Intercept | 161.71 | < .001 | 79.48 | [78.52, 80.45] |
| Maximum Parental Education | 19.02 | < .001 | 1.65 | [1.48, 1.82] |
| Family Income (High) | 6.91 | < .001 | 0.88 | [0.63, 1.13] |
| Family Income (Low) | -8.32 | < .001 | -1.21 | [-1.49, -0.92] |
| Sex | -1.78 | .075 | -0.13 | [-0.28, 0.01] |
| Child Race (American Indian/Alaska Native) | -0.50 | .620 | -0.48 | [-2.39, 1.43] |
| Child Race (Asian) | 2.86 | .004 | 1.74 | [0.55, 2.93] |
| Child Race (Black) | -3.59 | < .001 | -1.73 | [-2.67, -0.78] |
| Child Race (Native Hawaiian / Pacific Islander) | -2.40 | .016 | -4.74 | [-8.61, -0.87] |
| Child Race (Other) | 4.78 | < .001 | 2.24 | [1.32, 3.16] |
| Child Ethnicity | 8.76 | < .001 | 1.03 | [0.80, 1.26] |
| Age | 27.63 | < .001 | 0.27 | [0.25, 0.29] |
| Mid-Anterior Corpus Callosum Volume | 1.40 | .161 | 0.001 | [-0.0004, 0.003] |

**Note**: The linear mixed-effects model incorporates testing the statistical significance of coefficients against a *t*-distribution. Family Income was a categorical, effects-coded factor, in which the level “Mid” served as the reference level. Family Income was operationally defined as the self-reported combined family income and partitioned into three levels: Low Income: ≤ $50K; Mid Income: $50K-$100K; High Income: ≥ $100K. Sex was also a categorical factor, effect coded with Male/Female as ‑1/+1. Child Ethnicity was also a categorical factor, effect coded with Hispanic/Non-Hispanic as ‑1/+1. Child Race was also a categorical factor, in which “White” served as the reference level. Age, Maximum Parental Education (i.e., highest education level between parents/caregivers), and Mid-Anterior Corpus Callosum Volume were centered continuous factors. The random effects structure included a random intercept for study site identification number and family identification number. Random effects were restricted to be uncorrelated. Analysis included 8,524 data points. The model accounted for 56.3% of the variance in the data (*R*^2^ = .563, adjusted *R*^2^ = .562).

**Supplementary Table 97. Linear mixed-effects model output for the analysis of anterior corpus callosum volume and scores on the dimensional change card sort test (NIH Toolbox).**

|  | *t*(8511) | *p* | *b* | 95% CI |
| --- | --- | --- | --- | --- |
| Intercept | 150.53 | < .001 | 90.25 | [89.07, 91.42] |
| Maximum Parental Education | 8.05 | < .001 | 0.89 | [0.67, 1.11] |
| Family Income (High) | 3.30 | .001 | 0.54 | [0.22, 0.86] |
| Family Income (Low) | -4.98 | < .001 | -0.92 | [-1.29, -0.56] |
| Sex | 7.36 | < .001 | 0.71 | [0.52, 0.90] |
| Child Race (American Indian/Alaska Native) | -1.43 | .152 | -1.79 | [-4.24, 0.66] |
| Child Race (Asian) | 3.34 | .001 | 2.58 | [1.07, 4.10] |
| Child Race (Black) | -2.27 | .023 | -1.38 | [-2.57, -0.19] |
| Child Race (Native Hawaiian / Pacific Islander) | -1.03 | .302 | -2.56 | [-7.41, 2.30] |
| Child Race (Other) | 2.42 | .015 | 1.44 | [0.28, 2.60] |
| Child Ethnicity | 1.40 | .161 | 0.21 | [-0.08, 0.49] |
| Age | 18.37 | < .001 | 0.23 | [0.21, 0.26] |
| Anterior Corpus Callosum Volume | 2.06 | .039 | 0.002 | [0.0001, 0.003] |

**Note**: The linear mixed-effects model incorporates testing the statistical significance of coefficients against a *t*-distribution. Family Income was a categorical, effects-coded factor, in which the level “Mid” served as the reference level. Family Income was operationally defined as the self-reported combined family income and partitioned into three levels: Low Income: ≤ $50K; Mid Income: $50K-$100K; High Income: ≥ $100K. Sex was also a categorical factor, effect coded with Male/Female as ‑1/+1. Child Ethnicity was also a categorical factor, effect coded with Hispanic/Non-Hispanic as ‑1/+1. Child Race was also a categorical factor, in which “White” served as the reference level. Age, Maximum Parental Education (i.e., highest education level between parents/caregivers), and Anterior Corpus Callosum Volume were centered continuous factors. The random effects structure included a random intercept for study site identification number and family identification number. Random effects were restricted to be uncorrelated. Analysis included 8,524 data points. The model accounted for 21.5% of the variance in the data (*R*^2^ = .215, adjusted *R*^2^ = .214).

**Supplementary Table 98. Linear mixed-effects model output for the analysis of anterior corpus callosum volume and scores on the flanker inhibitory control and attention test (NIH Toolbox).**

|  | *t*(8511) | *p* | *b* | 95% CI |
| --- | --- | --- | --- | --- |
| Intercept | 160.50 | < .001 | 93.82 | [92.68, 94.97] |
| Maximum Parental Education | 6.32 | < .001 | 0.68 | [0.47, 0.89] |
| Family Income (High) | 3.58 | < .001 | 0.57 | [0.26, 0.88] |
| Family Income (Low) | -4.54 | < .001 | -0.82 | [-1.17, -0.46] |
| Sex | -1.40 | .162 | -0.13 | [-0.32, 0.05] |
| Child Race (American Indian/Alaska Native) | -1.05 | .295 | -1.27 | [-3.64, 1.11] |
| Child Race (Asian) | 2.44 | .015 | 1.83 | [0.36, 3.30] |
| Child Race (Black) | -4.70 | < .001 | -2.76 | [-3.91, -1.61] |
| Child Race (Native Hawaiian / Pacific Islander) | 1.21 | .227 | 2.89 | [-1.79, 7.56] |
| Child Race (Other) | -0.57 | .569 | -0.33 | [-1.45, 0.80] |
| Child Ethnicity | 0.47 | .637 | 0.07 | [-0.21, 0.35] |
| Age | 17.67 | < .001 | 0.22 | [0.20, 0.24] |
| Anterior Corpus Callosum Volume | 1.06 | .289 | 0.001 | [-0.001, 0.002] |

**Note**: The linear mixed-effects model incorporates testing the statistical significance of coefficients against a *t*-distribution. Family Income was a categorical, effects-coded factor, in which the level “Mid” served as the reference level. Family Income was operationally defined as the self-reported combined family income and partitioned into three levels: Low Income: ≤ $50K; Mid Income: $50K-$100K; High Income: ≥ $100K. Sex was also a categorical factor, effect coded with Male/Female as ‑1/+1. Child Ethnicity was also a categorical factor, effect coded with Hispanic/Non-Hispanic as ‑1/+1. Child Race was also a categorical factor, in which “White” served as the reference level. Age, Maximum Parental Education (i.e., highest education level between parents/caregivers), and Anterior Corpus Callosum Volume were centered continuous factors. The random effects structure included a random intercept for study site identification number and family identification number. Random effects were restricted to be uncorrelated. Analysis included 8,524 data points. The model accounted for 16.2% of the variance in the data (*R*^2^ = .162, adjusted *R*^2^ = .160).

**Supplementary Table 99. Linear mixed-effects model output for the analysis of anterior corpus callosum volume and scores on the list sorting working memory test (NIH Toolbox).**

|  | *t*(8511) | *p* | *b* | 95% CI |
| --- | --- | --- | --- | --- |
| Intercept | 122.89 | < .001 | 93.33 | [91.84, 94.82] |
| Maximum Parental Education | 13.09 | < .001 | 1.83 | [1.56, 2.11] |
| Family Income (High) | 5.28 | < .001 | 1.09 | [0.69, 1.49] |
| Family Income (Low) | -5.79 | < .001 | -1.36 | [-1.82, -0.90] |
| Sex | -2.78 | .005 | -0.34 | [-0.58, -0.10] |
| Child Race (American Indian/Alaska Native) | -0.38 | .707 | -0.59 | [-3.69, 2.50] |
| Child Race (Asian) | 0.98 | .329 | 0.96 | [-0.96, 2.88] |
| Child Race (Black) | -4.60 | < .001 | -3.54 | [-5.05, -2.03] |
| Child Race (Native Hawaiian / Pacific Islander) | 0.47 | .640 | 1.47 | [-4.69, 7.63] |
| Child Race (Other) | 0.60 | .551 | 0.45 | [-1.02, 1.92] |
| Child Ethnicity | 2.68 | .007 | 0.50 | [0.13, 0.86] |
| Age | 14.03 | < .001 | 0.23 | [0.19, 0.26] |
| Anterior Corpus Callosum Volume | 0.55 | .579 | 0.001 | [-0.001, 0.002] |

**Note**: The linear mixed-effects model incorporates testing the statistical significance of coefficients against a *t*-distribution. Family Income was a categorical, effects-coded factor, in which the level “Mid” served as the reference level. Family Income was operationally defined as the self-reported combined family income and partitioned into three levels: Low Income: ≤ $50K; Mid Income: $50K-$100K; High Income: ≥ $100K. Sex was also a categorical factor, effect coded with Male/Female as ‑1/+1. Child Ethnicity was also a categorical factor, effect coded with Hispanic/Non-Hispanic as ‑1/+1. Child Race was also a categorical factor, in which “White” served as the reference level. Age, Maximum Parental Education (i.e., highest education level between parents/caregivers), and Anterior Corpus Callosum Volume were centered continuous factors. The random effects structure included a random intercept for study site identification number and family identification number. Random effects were restricted to be uncorrelated. Analysis included 8,524 data points. The model accounted for 27.1% of the variance in the data (*R*^2^ = .271, adjusted *R*^2^ = .270).

**Supplementary Table 100. Linear mixed-effects model output for the analysis of anterior corpus callosum volume and scores on the pattern comparison processing speed test (NIH Toolbox).**

|  | *t*(8511) | *p* | *b* | 95% CI |
| --- | --- | --- | --- | --- |
| Intercept | 86.86 | < .001 | 87.77 | [85.79, 89.75] |
| Maximum Parental Education | 4.29 | < .001 | 0.76 | [0.41, 1.11] |
| Family Income (High) | 0.41 | .678 | 0.11 | [-0.40, 0.62] |
| Family Income (Low) | -1.48 | .139 | -0.44 | [-1.02, 0.14] |
| Sex | 6.31 | < .001 | 0.97 | [0.67, 1.27] |
| Child Race (American Indian/Alaska Native) | -1.83 | .067 | -3.65 | [-7.56, 0.26] |
| Child Race (Asian) | 2.43 | .015 | 3.01 | [0.58, 5.44] |
| Child Race (Black) | -2.63 | .008 | -2.56 | [-4.47, -0.66] |
| Child Race (Native Hawaiian / Pacific Islander) | 0.74 | .458 | 2.93 | [-4.82, 10.68] |
| Child Race (Other) | 0.09 | .926 | 0.09 | [-1.77, 1.94] |
| Child Ethnicity | 0.48 | .630 | 0.12 | [-0.36, 0.59] |
| Age | 20.90 | < .001 | 0.43 | [0.39, 0.47] |
| Anterior Corpus Callosum Volume | 1.35 | .177 | 0.002 | [-0.001, 0.004] |

**Note**: The linear mixed-effects model incorporates testing the statistical significance of coefficients against a *t*-distribution. Family Income was a categorical, effects-coded factor, in which the level “Mid” served as the reference level. Family Income was operationally defined as the self-reported combined family income and partitioned into three levels: Low Income: ≤ $50K; Mid Income: $50K-$100K; High Income: ≥ $100K. Sex was also a categorical factor, effect coded with Male/Female as ‑1/+1. Child Ethnicity was also a categorical factor, effect coded with Hispanic/Non-Hispanic as ‑1/+1. Child Race was also a categorical factor, in which “White” served as the reference level. Age, Maximum Parental Education (i.e., highest education level between parents/caregivers), and Anterior Corpus Callosum Volume were centered continuous factors. The random effects structure included a random intercept for study site identification number and family identification number. Random effects were restricted to be uncorrelated. Analysis included 8,524 data points. The model accounted for 19.3% of the variance in the data (*R*^2^ = .193, adjusted *R*^2^ = .192).

**Supplementary Table 101. Linear mixed-effects model output for the analysis of anterior corpus callosum volume and scores on the picture sequence memory test (NIH Toolbox).**

|  | *t*(8511) | *p* | *b* | 95% CI |
| --- | --- | --- | --- | --- |
| Intercept | 127.45 | < .001 | 101.37 | [99.81, 102.93] |
| Maximum Parental Education | 6.87 | < .001 | 1.01 | [0.72, 1.30] |
| Family Income (High) | 4.29 | < .001 | 0.93 | [0.51, 1.35] |
| Family Income (Low) | -3.48 | .001 | -0.86 | [-1.34, -0.37] |
| Sex | 5.83 | < .001 | 0.75 | [0.49, 1.00] |
| Child Race (American Indian/Alaska Native) | -0.01 | .990 | -0.02 | [-3.28, 3.23] |
| Child Race (Asian) | 1.21 | .224 | 1.25 | [-0.77, 3.27] |
| Child Race (Black) | -5.12 | < .001 | -4.16 | [-5.75, -2.57] |
| Child Race (Native Hawaiian / Pacific Islander) | 0.47 | .642 | 1.55 | [-4.97, 8.07] |
| Child Race (Other) | 0.36 | .718 | 0.29 | [-1.27, 1.84] |
| Child Ethnicity | 0.29 | .775 | 0.06 | [-0.32, 0.43] |
| Age | 10.88 | < .001 | 0.18 | [0.15, 0.22] |
| Anterior Corpus Callosum Volume | -0.92 | .359 | -0.001 | [-0.003, 0.001] |

**Note**: The linear mixed-effects model incorporates testing the statistical significance of coefficients against a *t*-distribution. Family Income was a categorical, effects-coded factor, in which the level “Mid” served as the reference level. Family Income was operationally defined as the self-reported combined family income and partitioned into three levels: Low Income: ≤ $50K; Mid Income: $50K-$100K; High Income: ≥ $100K. Sex was also a categorical factor, effect coded with Male/Female as ‑1/+1. Child Ethnicity was also a categorical factor, effect coded with Hispanic/Non-Hispanic as ‑1/+1. Child Race was also a categorical factor, in which “White” served as the reference level. Age, Maximum Parental Education (i.e., highest education level between parents/caregivers), and Anterior Corpus Callosum Volume were centered continuous factors. The random effects structure included a random intercept for study site identification number and family identification number. Random effects were restricted to be uncorrelated. Analysis included 8,524 data points. The model accounted for 24.2% of the variance in the data (*R*^2^ = .242, adjusted *R*^2^ = .241).

**Supplementary Table 102. Linear mixed-effects model output for the analysis of anterior corpus callosum volume and scores on the oral reading recognition test (NIH Toolbox).**

|  | *t*(8511) | *p* | *b* | 95% CI |
| --- | --- | --- | --- | --- |
| Intercept | 193.64 | < .001 | 89.20 | [88.30, 90.11] |
| Maximum Parental Education | 16.61 | < .001 | 1.30 | [1.15, 1.46] |
| Family Income (High) | 6.03 | < .001 | 0.70 | [0.47, 0.93] |
| Family Income (Low) | -8.17 | < .001 | -1.08 | [-1.34, -0.82] |
| Sex | 1.01 | .313 | 0.07 | [-0.06, 0.20] |
| Child Race (American Indian/Alaska Native) | -0.55 | .583 | -0.48 | [-2.21, 1.24] |
| Child Race (Asian) | 2.63 | .009 | 1.45 | [0.37, 2.53] |
| Child Race (Black) | -5.14 | < .001 | -2.25 | [-3.11, -1.39] |
| Child Race (Native Hawaiian / Pacific Islander) | 0.68 | .494 | 1.23 | [-2.30, 4.76] |
| Child Race (Other) | 0.14 | .891 | 0.06 | [-0.78, 0.89] |
| Child Ethnicity | 2.53 | .012 | 0.27 | [0.06, 0.48] |
| Age | 23.57 | < .001 | 0.21 | [0.19, 0.22] |
| Anterior Corpus Callosum Volume | 1.03 | .301 | 0.001 | [-0.0005, 0.002] |

**Note**: The linear mixed-effects model incorporates testing the statistical significance of coefficients against a *t*-distribution. Family Income was a categorical, effects-coded factor, in which the level “Mid” served as the reference level. Family Income was operationally defined as the self-reported combined family income and partitioned into three levels: Low Income: ≤ $50K; Mid Income: $50K-$100K; High Income: ≥ $100K. Sex was also a categorical factor, effect coded with Male/Female as ‑1/+1. Child Ethnicity was also a categorical factor, effect coded with Hispanic/Non-Hispanic as ‑1/+1. Child Race was also a categorical factor, in which “White” served as the reference level. Age, Maximum Parental Education (i.e., highest education level between parents/caregivers), and Anterior Corpus Callosum Volume were centered continuous factors. The random effects structure included a random intercept for study site identification number and family identification number. Random effects were restricted to be uncorrelated. Analysis included 8,524 data points. The model accounted for 52.7% of the variance in the data (*R*^2^ = .527, adjusted *R*^2^ = .527).

**Supplementary Table 103. Linear mixed-effects model output for the analysis of anterior corpus callosum volume and scores on the picture vocabulary test (NIH Toolbox).**

|  | *t*(8511) | *p* | *b* | 95% CI |
| --- | --- | --- | --- | --- |
| Intercept | 161.46 | < .001 | 79.53 | [78.56, 80.49] |
| Maximum Parental Education | 18.95 | < .001 | 1.64 | [1.47, 1.81] |
| Family Income (High) | 6.87 | < .001 | 0.88 | [0.63, 1.13] |
| Family Income (Low) | -8.26 | < .001 | -1.20 | [-1.49, -0.92] |
| Sex | -1.52 | .129 | -0.11 | [-0.26, 0.03] |
| Child Race (American Indian/Alaska Native) | -0.46 | .645 | -0.45 | [-2.36, 1.46] |
| Child Race (Asian) | 2.90 | .004 | 1.77 | [0.57, 2.96] |
| Child Race (Black) | -3.61 | < .001 | -1.74 | [-2.68, -0.80] |
| Child Race (Native Hawaiian / Pacific Islander) | -2.40 | .016 | -4.74 | [-8.61, -0.87] |
| Child Race (Other) | 4.73 | < .001 | 2.22 | [1.30, 3.14] |
| Child Ethnicity | 8.68 | < .001 | 1.02 | [0.79, 1.25] |
| Age | 27.60 | < .001 | 0.27 | [0.25, 0.29] |
| Anterior Corpus Callosum Volume | 2.87 | .004 | 0.002 | [0.001, 0.003] |

**Note**: The linear mixed-effects model incorporates testing the statistical significance of coefficients against a *t*-distribution. Family Income was a categorical, effects-coded factor, in which the level “Mid” served as the reference level. Family Income was operationally defined as the self-reported combined family income and partitioned into three levels: Low Income: ≤ $50K; Mid Income: $50K-$100K; High Income: ≥ $100K. Sex was also a categorical factor, effect coded with Male/Female as ‑1/+1. Child Ethnicity was also a categorical factor, effect coded with Hispanic/Non-Hispanic as ‑1/+1. Child Race was also a categorical factor, in which “White” served as the reference level. Age, Maximum Parental Education (i.e., highest education level between parents/caregivers), and Anterior Corpus Callosum Volume were centered continuous factors. The random effects structure included a random intercept for study site identification number and family identification number. Random effects were restricted to be uncorrelated. Analysis included 8,524 data points. The model accounted for 56.4% of the variance in the data (*R*^2^ = .564, adjusted *R*^2^ = .563).

**Supplementary Table 104. Linear mixed-effects model output for the analysis of lead risk and scores on the dimensional change card sort test (NIH Toolbox).**

|  | *t*(8511) | *p* | *b* | 95% CI |
| --- | --- | --- | --- | --- |
| Intercept | 150.48 | < .001 | 90.21 | [89.03, 91.38] |
| Maximum Parental Education | 8.02 | < .001 | 0.89 | [0.67, 1.11] |
| Family Income (High) | 3.13 | .002 | 0.52 | [0.19, 0.84] |
| Family Income (Low) | -4.75 | < .001 | -0.90 | [-1.26, -0.53] |
| Sex | 7.11 | < .001 | 0.68 | [0.49, 0.87] |
| Child Race (American Indian/Alaska Native) | -1.46 | .144 | -1.83 | [-4.28, 0.62] |
| Child Race (Asian) | 3.29 | .001 | 2.55 | [1.03, 4.07] |
| Child Race (Black) | -2.19 | .028 | -1.34 | [-2.53, -0.14] |
| Child Race (Native Hawaiian / Pacific Islander) | -1.04 | .296 | -2.59 | [-7.44, 2.27] |
| Child Race (Other) | 2.48 | .013 | 1.47 | [0.31, 2.63] |
| Child Ethnicity | 1.43 | .153 | 0.21 | [-0.08, 0.50] |
| Age | 18.41 | < .001 | 0.23 | [0.21, 0.26] |
| Lead Risk | -1.22 | .224 | -0.04 | [-0.11, 0.03] |

**Note**: This model corresponds to the total effect of lead risk on performance in the dimensional change card sort test, as part of the analyses conducted to investigate indirect associations between lead risk and cognition. The linear mixed-effects model incorporates testing the statistical significance of coefficients against a *t*-distribution. Family Income was a categorical, effects-coded factor, in which the level “Mid” served as the reference level. Family Income was operationally defined as the self-reported combined family income and partitioned into three levels: Low Income: ≤ $50K; Mid Income: $50K-$100K; High Income: ≥ $100K. Sex was also a categorical factor, effect coded with Male/Female as ‑1/+1. Child Ethnicity was also a categorical factor, effect coded with Hispanic/Non-Hispanic as ‑1/+1. Child Race was also a categorical factor, in which “White” served as the reference level. Age, Maximum Parental Education (i.e., highest education level between parents/caregivers), and Lead Risk were centered continuous factors. The random effects structure included a random intercept for study site identification number and family identification number. Random effects were restricted to be uncorrelated. Analysis included 8,524 data points. The model accounted for 21.6% of the variance in the data (*R*^2^ = .216, adjusted *R*^2^ = .215).

**Supplementary Table 105. Linear mixed-effects model output for the analysis of lead risk and scores on the picture vocabulary test (NIH Toolbox).**

|  | *t*(8511) | *p* | *b* | 95% CI |
| --- | --- | --- | --- | --- |
| Intercept | 162.40 | < .001 | 79.45 | [78.49, 80.41] |
| Maximum Parental Education | 19.08 | < .001 | 1.66 | [1.49, 1.83] |
| Family Income (High) | 7.09 | < .001 | 0.92 | [0.66, 1.17] |
| Family Income (Low) | -8.51 | < .001 | -1.25 | [-1.54, -0.97] |
| Sex | -1.98 | .048 | -0.15 | [-0.29, -0.001] |
| Child Race (American Indian/Alaska Native) | -0.48 | .631 | -0.47 | [-2.38, 1.44] |
| Child Race (Asian) | 2.93 | .003 | 1.79 | [0.59, 2.98] |
| Child Race (Black) | -3.75 | < .001 | -1.81 | [-2.75, -0.86] |
| Child Race (Native Hawaiian / Pacific Islander) | -2.42 | .016 | -4.77 | [-8.64, -0.90] |
| Child Race (Other) | 4.82 | < .001 | 2.26 | [1.34, 3.18] |
| Child Ethnicity | 8.89 | < .001 | 1.04 | [0.81, 1.27] |
| Age | 27.67 | < .001 | 0.27 | [0.25, 0.29] |
| Lead Risk | 1.70 | .089 | 0.05 | [-0.01, 0.11] |

**Note**: This model corresponds to the total effect of lead risk on performance in the picture vocabulary test, as part of the analyses conducted to investigate indirect associations between lead risk and cognition. The linear mixed-effects model incorporates testing the statistical significance of coefficients against a *t*-distribution. Family Income was a categorical, effects-coded factor, in which the level “Mid” served as the reference level. Family Income was operationally defined as the self-reported combined family income and partitioned into three levels: Low Income: ≤ $50K; Mid Income: $50K-$100K; High Income: ≥ $100K. Sex was also a categorical factor, effect coded with Male/Female as ‑1/+1. Child Ethnicity was also a categorical factor, effect coded with Hispanic/Non-Hispanic as ‑1/+1. Child Race was also a categorical factor, in which “White” served as the reference level. Age, Maximum Parental Education (i.e., highest education level between parents/caregivers), and Lead Risk were centered continuous factors. The random effects structure included a random intercept for study site identification number and family identification number. Random effects were restricted to be uncorrelated. Analysis included 8,524 data points. The model accounted for 56.3% of the variance in the data (*R*^2^ = .563, adjusted *R*^2^ = .562).

**Supplementary Table 106. Linear mixed-effects model output for the analysis of lead risk and scores on the oral reading recognition test (NIH Toolbox).**

|  | *t*(8511) | *p* | *b* | 95% CI |
| --- | --- | --- | --- | --- |
| Intercept | 194.42 | < .001 | 89.18 | [88.28, 90.08] |
| Maximum Parental Education | 16.66 | < .001 | 1.31 | [1.16, 1.46] |
| Family Income (High) | 6.10 | < .001 | 0.72 | [0.49, 0.95] |
| Family Income (Low) | -8.21 | < .001 | -1.10 | [-1.36, -0.84] |
| Sex | 0.86 | .389 | 0.06 | [-0.07, 0.19] |
| Child Race (American Indian/Alaska Native) | -0.56 | .577 | -0.49 | [-2.22, 1.24] |
| Child Race (Asian) | 2.65 | .008 | 1.46 | [0.38, 2.55] |
| Child Race (Black) | -5.19 | < .001 | -2.28 | [-3.14, -1.42] |
| Child Race (Native Hawaiian / Pacific Islander) | 0.68 | .498 | 1.22 | [-2.31, 4.74] |
| Child Race (Other) | 0.17 | .868 | 0.07 | [-0.76, 0.91] |
| Child Ethnicity | 2.60 | .009 | 0.28 | [0.07, 0.49] |
| Age | 23.60 | < .001 | 0.21 | [0.19, 0.22] |
| Lead Risk | 0.82 | .414 | 0.02 | [-0.03, 0.07] |

**Note**: This model corresponds to the total effect of lead risk on performance in the oral reading recognition test, as part of the analyses conducted to investigate indirect associations between lead risk and cognition. The linear mixed-effects model incorporates testing the statistical significance of coefficients against a *t*-distribution. Family Income was a categorical, effects-coded factor, in which the level “Mid” served as the reference level. Family Income was operationally defined as the self-reported combined family income and partitioned into three levels: Low Income: ≤ $50K; Mid Income: $50K-$100K; High Income: ≥ $100K. Sex was also a categorical factor, effect coded with Male/Female as ‑1/+1. Child Ethnicity was also a categorical factor, effect coded with Hispanic/Non-Hispanic as ‑1/+1. Child Race was also a categorical factor, in which “White” served as the reference level. Age, Maximum Parental Education (i.e., highest education level between parents/caregivers), and Lead Risk were centered continuous factors. The random effects structure included a random intercept for study site identification number and family identification number. Random effects were restricted to be uncorrelated. Analysis included 8,524 data points. The model accounted for 52.7% of the variance in the data (*R*^2^ = .527, adjusted *R*^2^ = .526).

**Supplementary Table 107. Linear mixed-effects model output for the analysis of lead risk and scores on the pattern comparison processing speed test (NIH Toolbox).**

|  | *t*(8511) | *p* | *b* | 95% CI |
| --- | --- | --- | --- | --- |
| Intercept | 86.67 | < .001 | 87.76 | [85.77, 89.74] |
| Maximum Parental Education | 4.21 | < .001 | 0.75 | [0.40, 1.09] |
| Family Income (High) | 0.12 | .901 | 0.03 | [-0.48, 0.55] |
| Family Income (Low) | -1.13 | .259 | -0.34 | [-0.93, 0.25] |
| Sex | 6.17 | < .001 | 0.94 | [0.64, 1.24] |
| Child Race (American Indian/Alaska Native) | -1.85 | .064 | -3.69 | [-7.60, 0.21] |
| Child Race (Asian) | 2.36 | .018 | 2.93 | [0.50, 5.35] |
| Child Race (Black) | -2.48 | .013 | -2.42 | [-4.33, -0.51] |
| Child Race (Native Hawaiian / Pacific Islander) | 0.73 | .464 | 2.90 | [-4.85, 10.65] |
| Child Race (Other) | 0.12 | .901 | 0.12 | [-1.73, 1.97] |
| Child Ethnicity | 0.42 | .672 | 0.10 | [-0.37, 0.57] |
| Age | 20.91 | < .001 | 0.43 | [0.39, 0.47] |
| Lead Risk | -2.12 | .034 | -0.13 | [-0.24, -0.01] |

**Note**: This model corresponds to the total effect of lead risk on performance in the pattern comparison processing speed test, as part of the analyses conducted to investigate indirect associations between lead risk and cognition. The linear mixed-effects model incorporates testing the statistical significance of coefficients against a *t*-distribution. Family Income was a categorical, effects-coded factor, in which the level “Mid” served as the reference level. Family Income was operationally defined as the self-reported combined family income and partitioned into three levels: Low Income: ≤ $50K; Mid Income: $50K-$100K; High Income: ≥ $100K. Sex was also a categorical factor, effect coded with Male/Female as ‑1/+1. Child Ethnicity was also a categorical factor, effect coded with Hispanic/Non-Hispanic as ‑1/+1. Child Race was also a categorical factor, in which “White” served as the reference level. Age, Maximum Parental Education (i.e., highest education level between parents/caregivers), and Lead Risk were centered continuous factors. The random effects structure included a random intercept for study site identification number and family identification number. Random effects were restricted to be uncorrelated. Analysis included 8,524 data points. The model accounted for 19.3% of the variance in the data (*R*^2^ = .193, adjusted *R*^2^ = .192).

**Supplementary Table 108. Linear mixed-effects model output for the analysis of lead risk and scores on the flanker inhibitory control and attention test (NIH Toolbox).**

|  | *t*(8511) | *p* | *b* | 95% CI |
| --- | --- | --- | --- | --- |
| Intercept | 160.76 | < .001 | 93.81 | [92.67, 94.95] |
| Maximum Parental Education | 6.28 | < .001 | 0.68 | [0.46, 0.89] |
| Family Income (High) | 3.41 | .001 | 0.54 | [0.23, 0.86] |
| Family Income (Low) | -4.32 | < .001 | -0.79 | [-1.15, -0.43] |
| Sex | -1.59 | .112 | -0.15 | [-0.33, 0.03] |
| Child Race (American Indian/Alaska Native) | -1.06 | .287 | -1.29 | [-3.66, 1.09] |
| Child Race (Asian) | 2.41 | .016 | 1.80 | [0.33, 3.27] |
| Child Race (Black) | -4.62 | < .001 | -2.72 | [-3.87, -1.57] |
| Child Race (Native Hawaiian / Pacific Islander) | 1.20 | .229 | 2.87 | [-1.81, 7.55] |
| Child Race (Other) | -0.54 | .586 | -0.31 | [-1.43, 0.81] |
| Child Ethnicity | 0.45 | .650 | 0.07 | [-0.22, 0.35] |
| Age | 17.69 | < .001 | 0.22 | [0.20, 0.24] |
| Lead Risk | -1.12 | .263 | -0.04 | [-0.11, 0.03] |

**Note**: This model corresponds to the total effect of lead risk on performance in the flanker inhibitory control and attention test, as part of the analyses conducted to investigate indirect associations between lead risk and cognition. The linear mixed-effects model incorporates testing the statistical significance of coefficients against a *t*-distribution. Family Income was a categorical, effects-coded factor, in which the level “Mid” served as the reference level. Family Income was operationally defined as the self-reported combined family income and partitioned into three levels: Low Income: ≤ $50K; Mid Income: $50K-$100K; High Income: ≥ $100K. Sex was also a categorical factor, effect coded with Male/Female as ‑1/+1. Child Ethnicity was also a categorical factor, effect coded with Hispanic/Non-Hispanic as ‑1/+1. Child Race was also a categorical factor, in which “White” served as the reference level. Age, Maximum Parental Education (i.e., highest education level between parents/caregivers), and Lead Risk were centered continuous factors. The random effects structure included a random intercept for study site identification number and family identification number. Random effects were restricted to be uncorrelated. Analysis included 8,524 data points. The model accounted for 16.1% of the variance in the data (*R*^2^ = .161, adjusted *R*^2^ = .160).

**Supplementary Table 109. Linear mixed-effects model output for the analysis of lead risk and volume of the mid-anterior corpus callosum.**

|  | *t*(8511) | *p* | *b* | 95% CI |
| --- | --- | --- | --- | --- |
| Intercept | 56.88 | < .001 | 424.57 | [409.94, 439.20] |
| Maximum Parental Education | -0.56 | .577 | -0.69 | [-3.12, 1.74] |
| Family Income (High) | -0.38 | .701 | -0.71 | [-4.33, 2.92] |
| Family Income (Low) | -0.48 | .633 | -1.01 | [-5.14, 3.13] |
| Sex | -12.69 | < .001 | -13.32 | [-15.38, -11.26] |
| Child Race (American Indian/Alaska Native) | 0.47 | .640 | 6.50 | [-20.77, 33.77] |
| Child Race (Asian) | 1.89 | .058 | 16.49 | [-0.58, 33.55] |
| Child Race (Black) | -2.45 | .014 | -16.90 | [-30.43, -3.36] |
| Child Race (Native Hawaiian / Pacific Islander) | -1.06 | .287 | -30.05 | [-85.42, 25.31] |
| Child Race (Other) | 1.90 | .057 | 12.75 | [-0.38, 25.88] |
| Child Ethnicity | 2.16 | .030 | 3.67 | [0.35, 6.99] |
| Age | 1.37 | .171 | 0.19 | [-0.08, 0.46] |
| Lead Risk | -3.61 | < .001 | -1.50 | [-2.31, -0.69] |

**Note**: This model corresponds to the association between lead risk and volume of the mid-anterior corpus callosum, as part of the analyses conducted to investigate indirect associations between lead risk and cognition. The linear mixed-effects model incorporates testing the statistical significance of coefficients against a *t*-distribution. Family Income was a categorical, effects-coded factor, in which the level “Mid” served as the reference level. Family Income was operationally defined as the self-reported combined family income and partitioned into three levels: Low Income: ≤ $50K; Mid Income: $50K-$100K; High Income: ≥ $100K. Sex was also a categorical factor, effect coded with Male/Female as ‑1/+1. Child Ethnicity was also a categorical factor, effect coded with Hispanic/Non-Hispanic as ‑1/+1. Child Race was also a categorical factor, in which “White” served as the reference level. Age, Maximum Parental Education (i.e., highest education level between parents/caregivers), and Lead Risk were centered continuous factors. The random effects structure included a random intercept for study site identification number and family identification number. Random effects were restricted to be uncorrelated. Analysis included 8,524 data points. The model accounted for 35.0% of the variance in the data (*R*^2^ = .350, adjusted *R*^2^ = .349).

**Supplementary Table 110. Linear mixed-effects model output for the analysis of lead risk and volume of the central corpus callosum.**

|  | *t*(8511) | *p* | *b* | 95% CI |
| --- | --- | --- | --- | --- |
| Intercept | 57.83 | < .001 | 399.87 | [386.32, 413.43] |
| Maximum Parental Education | 0.62 | .535 | 0.67 | [-1.44, 2.78] |
| Family Income (High) | -0.54 | .590 | -0.87 | [-4.02, 2.29] |
| Family Income (Low) | -0.86 | .391 | -1.57 | [-5.17, 2.02] |
| Sex | -5.66 | < .001 | -5.17 | [-6.95, -3.38] |
| Child Race (American Indian/Alaska Native) | 0.28 | .781 | 3.37 | [-20.33, 27.06] |
| Child Race (Asian) | 1.25 | .210 | 9.48 | [-5.36, 24.31] |
| Child Race (Black) | -0.33 | .740 | -1.99 | [-13.76, 9.77] |
| Child Race (Native Hawaiian / Pacific Islander) | -1.22 | .223 | -29.92 | [-78.01, 18.16] |
| Child Race (Other) | 1.95 | .051 | 11.37 | [-0.03, 22.78] |
| Child Ethnicity | 2.34 | .019 | 3.47 | [0.57, 6.37] |
| Age | 5.83 | < .001 | 0.71 | [0.47, 0.94] |
| Lead Risk | -3.22 | .001 | -1.17 | [-1.88, -0.46] |

**Note**: This model corresponds to the association between lead risk and volume of the central corpus callosum, as part of the analyses conducted to investigate indirect associations between lead risk and cognition. The linear mixed-effects model incorporates testing the statistical significance of coefficients against a *t*-distribution. Family Income was a categorical, effects-coded factor, in which the level “Mid” served as the reference level. Family Income was operationally defined as the self-reported combined family income and partitioned into three levels: Low Income: ≤ $50K; Mid Income: $50K-$100K; High Income: ≥ $100K. Sex was also a categorical factor, effect coded with Male/Female as ‑1/+1. Child Ethnicity was also a categorical factor, effect coded with Hispanic/Non-Hispanic as ‑1/+1. Child Race was also a categorical factor, in which “White” served as the reference level. Age, Maximum Parental Education (i.e., highest education level between parents/caregivers), and Lead Risk were centered continuous factors. The random effects structure included a random intercept for study site identification number and family identification number. Random effects were restricted to be uncorrelated. Analysis included 8,524 data points. The model accounted for 34.3% of the variance in the data (*R*^2^ = .343, adjusted *R*^2^ = .342).

**Supplementary Table 111. Linear mixed-effects model output for the analysis of lead risk and volume of the mid-posterior corpus callosum.**

|  | *t*(8511) | *p* | *b* | 95% CI |
| --- | --- | --- | --- | --- |
| Intercept | 62.22 | < .001 | 370.45 | [358.78, 382.12] |
| Maximum Parental Education | 2.08 | .038 | 2.03 | [0.11, 3.94] |
| Family Income (High) | -0.26 | .792 | -0.38 | [-3.24, 2.47] |
| Family Income (Low) | -1.06 | .287 | -1.77 | [-5.02, 1.49] |
| Sex | -8.13 | < .001 | -6.69 | [-8.31, -5.08] |
| Child Race (American Indian/Alaska Native) | 1.06 | .287 | 11.65 | [-9.80, 33.10] |
| Child Race (Asian) | -0.91 | .361 | -6.27 | [-19.71, 7.18] |
| Child Race (Black) | -2.34 | .019 | -12.78 | [-23.46, -2.10] |
| Child Race (Native Hawaiian / Pacific Islander) | -0.64 | .524 | -14.22 | [-57.97, 29.53] |
| Child Race (Other) | 1.70 | .088 | 9.01 | [-1.35, 19.37] |
| Child Ethnicity | 1.79 | .074 | 2.38 | [-0.23, 5.00] |
| Age | 8.48 | < .001 | 0.92 | [0.71, 1.14] |
| Lead Risk | -3.08 | .002 | -1.01 | [-1.65, -0.37] |

**Note**: This model corresponds to the association between lead risk and volume of the mid-posterior corpus callosum, as part of the analyses conducted to investigate indirect associations between lead risk and cognition. The linear mixed-effects model incorporates testing the statistical significance of coefficients against a *t*-distribution. Family Income was a categorical, effects-coded factor, in which the level “Mid” served as the reference level. Family Income was operationally defined as the self-reported combined family income and partitioned into three levels: Low Income: ≤ $50K; Mid Income: $50K-$100K; High Income: ≥ $100K. Sex was also a categorical factor, effect coded with Male/Female as ‑1/+1. Child Ethnicity was also a categorical factor, effect coded with Hispanic/Non-Hispanic as ‑1/+1. Child Race was also a categorical factor, in which “White” served as the reference level. Age, Maximum Parental Education (i.e., highest education level between parents/caregivers), and Lead Risk were centered continuous factors. The random effects structure included a random intercept for study site identification number and family identification number. Random effects were restricted to be uncorrelated. Analysis included 8,524 data points. The model accounted for 40.9% of the variance in the data (*R*^2^ = .409, adjusted *R*^2^ = .408).

**Supplementary Table 112. Linear mixed-effects model output for the analysis of lead risk, mid-posterior corpus callosal volume, central corpus callosal volume, and scores on the dimensional change card sort test (NIH Toolbox).**

|  | *t*(8509) | *p* | *b* | 95% CI |
| --- | --- | --- | --- | --- |
| Intercept | 150.08 | < .001 | 90.27 | [89.09, 91.45] |
| Maximum Parental Education | 7.93 | < .001 | 0.88 | [0.66, 1.10] |
| Family Income (High) | 3.12 | .002 | 0.51 | [0.19, 0.84] |
| Family Income (Low) | -4.69 | < .001 | -0.88 | [-1.25, -0.51] |
| Sex | 7.43 | < .001 | 0.71 | [0.52, 0.90] |
| Child Race (American Indian/Alaska Native) | -1.50 | .133 | -1.87 | [-4.32, 0.57] |
| Child Race (Asian) | 3.33 | .001 | 2.58 | [1.06, 4.09] |
| Child Race (Black) | -2.08 | .038 | -1.27 | [-2.46, -0.07] |
| Child Race (Native Hawaiian / Pacific Islander) | -1.02 | .307 | -2.53 | [-7.38, 2.32] |
| Child Race (Other) | 2.41 | .016 | 1.43 | [0.27, 2.59] |
| Child Ethnicity | 1.37 | .172 | 0.20 | [-0.09, 0.49] |
| Age | 17.99 | < .001 | 0.23 | [0.21, 0.26] |
| Lead Risk | -1.09 | .278 | -0.04 | [-0.11, 0.03] |
| Mid-Posterior Corpus Callosum Volume | 2.94 | .003 | 0.005 | [0.002, 0.01] |
| Central Corpus Callosum Volume | 0.16 | .870 | 0.0002 | [-0.003, 0.003] |

**Note**: This model corresponds to the direct effect of lead risk on performance in the dimensional change card sort test, controlling for mid-posterior and central corpus callosal volume, as part of the analyses conducted to investigate indirect associations between lead risk and cognition. The linear mixed-effects model incorporates testing the statistical significance of coefficients against a *t*-distribution. Family Income was a categorical, effects-coded factor, in which the level “Mid” served as the reference level. Family Income was operationally defined as the self-reported combined family income and partitioned into three levels: Low Income: ≤ $50K; Mid Income: $50K-$100K; High Income: ≥ $100K. Sex was also a categorical factor, effect coded with Male/Female as ‑1/+1. Child Ethnicity was also a categorical factor, effect coded with Hispanic/Non-Hispanic as ‑1/+1. Child Race was also a categorical factor, in which “White” served as the reference level. Age, Maximum Parental Education (i.e., highest education level between parents/caregivers), Lead Risk, Mid-Posterior Corpus Callosum Volume, and Central Corpus Callosum Volume were centered continuous factors. The random effects structure included a random intercept for study site identification number and family identification number. Random effects were restricted to be uncorrelated. Analysis included 8,524 data points. The model accounted for 21.7% of the variance in the data (*R*^2^ = .217, adjusted *R*^2^ = .216).

**Supplementary Table 113. Linear mixed-effects model output for the analysis of lead risk, mid-posterior corpus callosal volume, central corpus callosal volume, and scores on the picture vocabulary test (NIH Toolbox).**

|  | *t*(8509) | *p* | *b* | 95% CI |
| --- | --- | --- | --- | --- |
| Intercept | 162.57 | < .001 | 79.52 | [78.57, 80.48] |
| Maximum Parental Education | 18.99 | < .001 | 1.65 | [1.48, 1.81] |
| Family Income (High) | 7.12 | < .001 | 0.92 | [0.66, 1.17] |
| Family Income (Low) | -8.46 | < .001 | -1.24 | [-1.53, -0.96] |
| Sex | -1.51 | .130 | -0.11 | [-0.26, 0.03] |
| Child Race (American Indian/Alaska Native) | -0.54 | .589 | -0.53 | [-2.43, 1.38] |
| Child Race (Asian) | 3.00 | .003 | 1.82 | [0.63, 3.01] |
| Child Race (Black) | -3.62 | < .001 | -1.74 | [-2.69, -0.80] |
| Child Race (Native Hawaiian / Pacific Islander) | -2.39 | .017 | -4.70 | [-8.56, -0.84] |
| Child Race (Other) | 4.73 | < .001 | 2.21 | [1.30, 3.13] |
| Child Ethnicity | 8.80 | < .001 | 1.03 | [0.80, 1.26] |
| Age | 27.11 | < .001 | 0.27 | [0.25, 0.28] |
| Lead Risk | 1.88 | .061 | 0.05 | [-0.002, 0.11] |
| Mid-Posterior Corpus Callosum Volume | 4.23 | < .001 | 0.01 | [0.003, 0.01] |
| Central Corpus Callosum Volume | -0.12 | .902 | -0.0001 | [-0.002, 0.002] |

**Note**: This model corresponds to the direct effect of lead risk on performance in the picture vocabulary test, controlling for mid-posterior and central corpus callosal volume, as part of the analyses conducted to investigate indirect associations between lead risk and cognition. The linear mixed-effects model incorporates testing the statistical significance of coefficients against a *t*-distribution. Family Income was a categorical, effects-coded factor, in which the level “Mid” served as the reference level. Family Income was operationally defined as the self-reported combined family income and partitioned into three levels: Low Income: ≤ $50K; Mid Income: $50K-$100K; High Income: ≥ $100K. Sex was also a categorical factor, effect coded with Male/Female as ‑1/+1. Child Ethnicity was also a categorical factor, effect coded with Hispanic/Non-Hispanic as ‑1/+1. Child Race was also a categorical factor, in which “White” served as the reference level. Age, Maximum Parental Education (i.e., highest education level between parents/caregivers), Lead Risk, Mid-Posterior Corpus Callosum Volume, and Central Corpus Callosum Volume were centered continuous factors. The random effects structure included a random intercept for study site identification number and family identification number. Random effects were restricted to be uncorrelated. Analysis included 8,524 data points. The model accounted for 56.1% of the variance in the data (*R*^2^ = .561, adjusted *R*^2^ = .561).

**Supplementary Table 114. Linear mixed-effects model output for the analysis of lead risk, mid-posterior corpus callosal volume, and scores on the oral reading recognition test (NIH Toolbox).**

|  | *t*(8510) | *p* | *b* | 95% CI |
| --- | --- | --- | --- | --- |
| Intercept | 194.13 | < .001 | 89.22 | [88.32, 90.12] |
| Maximum Parental Education | 16.59 | < .001 | 1.30 | [1.15, 1.46] |
| Family Income (High) | 6.11 | < .001 | 0.72 | [0.49, 0.95] |
| Family Income (Low) | -8.18 | < .001 | -1.09 | [-1.36, -0.83] |
| Sex | 1.14 | .254 | 0.08 | [-0.05, 0.21] |
| Child Race (American Indian/Alaska Native) | -0.59 | .553 | -0.52 | [-2.25, 1.21] |
| Child Race (Asian) | 2.68 | .007 | 1.48 | [0.40, 2.56] |
| Child Race (Black) | -5.11 | < .001 | -2.24 | [-3.10, -1.38] |
| Child Race (Native Hawaiian / Pacific Islander) | 0.70 | .485 | 1.26 | [-2.27, 4.78] |
| Child Race (Other) | 0.11 | .914 | 0.05 | [-0.79, 0.88] |
| Child Ethnicity | 2.54 | .011 | 0.27 | [0.06, 0.48] |
| Age | 23.23 | < .001 | 0.20 | [0.19, 0.22] |
| Lead Risk | 0.92 | .360 | 0.02 | [-0.03, 0.08] |
| Mid-Posterior Corpus Callosum Volume | 3.21 | .001 | 0.003 | [0.001, 0.01] |

**Note**: This model corresponds to the direct effect of lead risk on performance in the oral reading recognition test, controlling for mid-posterior corpus callosal volume, as part of the analyses conducted to investigate indirect associations between lead risk and cognition. The linear mixed-effects model incorporates testing the statistical significance of coefficients against a *t*-distribution. Family Income was a categorical, effects-coded factor, in which the level “Mid” served as the reference level. Family Income was operationally defined as the self-reported combined family income and partitioned into three levels: Low Income: ≤ $50K; Mid Income: $50K-$100K; High Income: ≥ $100K. Sex was also a categorical factor, effect coded with Male/Female as ‑1/+1. Child Ethnicity was also a categorical factor, effect coded with Hispanic/Non-Hispanic as ‑1/+1. Child Race was also a categorical factor, in which “White” served as the reference level. Age, Maximum Parental Education (i.e., highest education level between parents/caregivers), Lead Risk, and Mid-Posterior Corpus Callosum Volume were centered continuous factors. The random effects structure included a random intercept for study site identification number and family identification number. Random effects were restricted to be uncorrelated. Analysis included 8,524 data points. The model accounted for 52.8% of the variance in the data (*R*^2^ = .528, adjusted *R*^2^ = .528).

**Supplementary Table 115. Linear mixed-effects model output for the analysis of lead risk, mid-posterior corpus callosal volume, mid-anterior corpus callosal volume, and scores on the pattern comparison processing speed test (NIH Toolbox).**

|  | *t*(8509) | *p* | *b* | 95% CI |
| --- | --- | --- | --- | --- |
| Intercept | 86.33 | < .001 | 87.84 | [85.85, 89.84] |
| Maximum Parental Education | 4.14 | < .001 | 0.73 | [0.39, 1.08] |
| Family Income (High) | 0.12 | .903 | 0.03 | [-0.48, 0.55] |
| Family Income (Low) | -1.08 | .279 | -0.33 | [-0.92, 0.26] |
| Sex | 6.40 | < .001 | 0.98 | [0.68, 1.28] |
| Child Race (American Indian/Alaska Native) | -1.89 | .059 | -3.76 | [-7.66, 0.15] |
| Child Race (Asian) | 2.38 | .017 | 2.95 | [0.52, 5.38] |
| Child Race (Black) | -2.39 | .017 | -2.33 | [-4.24, -0.42] |
| Child Race (Native Hawaiian / Pacific Islander) | 0.75 | .451 | 2.98 | [-4.76, 10.72] |
| Child Race (Other) | 0.07 | .947 | 0.06 | [-1.79, 1.91] |
| Child Ethnicity | 0.37 | .712 | 0.09 | [-0.38, 0.56] |
| Age | 20.54 | < .001 | 0.42 | [0.38, 0.46] |
| Lead Risk | -2.02 | .043 | -0.12 | [-0.23, -0.004] |
| Mid-Posterior Corpus Callosum Volume | 2.47 | .014 | 0.01 | [0.001, 0.01] |
| Mid-Anterior Corpus Callosum Volume | 0.12 | .901 | 0.0002 | [-0.004, 0.004] |

**Note**: This model corresponds to the direct effect of lead risk on performance in the pattern comparison processing speed test, controlling for mid-posterior and mid-anterior corpus callosal volume, as part of the analyses conducted to investigate indirect associations between lead risk and cognition. The linear mixed-effects model incorporates testing the statistical significance of coefficients against a *t*-distribution. Family Income was a categorical, effects-coded factor, in which the level “Mid” served as the reference level. Family Income was operationally defined as the self-reported combined family income and partitioned into three levels: Low Income: ≤ $50K; Mid Income: $50K-$100K; High Income: ≥ $100K. Sex was also a categorical factor, effect coded with Male/Female as ‑1/+1. Child Ethnicity was also a categorical factor, effect coded with Hispanic/Non-Hispanic as ‑1/+1. Child Race was also a categorical factor, in which “White” served as the reference level. Age, Maximum Parental Education (i.e., highest education level between parents/caregivers), Lead Risk, Mid-Posterior Corpus Callosum Volume, and Mid-Anterior Corpus Callosum Volume were centered continuous factors. The random effects structure included a random intercept for study site identification number and family identification number. Random effects were restricted to be uncorrelated. Analysis included 8,524 data points. The model accounted for 19.3% of the variance in the data (*R*^2^ = .193, adjusted *R*^2^ = .192).

**Supplementary Table 116. Linear mixed-effects model output for the analysis of lead risk, mid-posterior corpus callosal volume, and scores on the flanker inhibitory control and attention test (NIH Toolbox).**

|  | *t*(8510) | *p* | *b* | 95% CI |
| --- | --- | --- | --- | --- |
| Intercept | 160.45 | < .001 | 93.87 | [92.72, 95.01] |
| Maximum Parental Education | 6.20 | < .001 | 0.67 | [0.46, 0.88] |
| Family Income (High) | 3.41 | .001 | 0.54 | [0.23, 0.86] |
| Family Income (Low) | -4.27 | < .001 | -0.78 | [-1.14, -0.42] |
| Sex | -1.28 | .199 | -0.12 | [-0.30, 0.06] |
| Child Race (American Indian/Alaska Native) | -1.10 | .269 | -1.34 | [-3.71, 1.03] |
| Child Race (Asian) | 2.44 | .015 | 1.83 | [0.36, 3.30] |
| Child Race (Black) | -4.53 | < .001 | -2.66 | [-3.82, -1.51] |
| Child Race (Native Hawaiian / Pacific Islander) | 1.23 | .220 | 2.93 | [-1.75, 7.60] |
| Child Race (Other) | -0.61 | .543 | -0.35 | [-1.47, 0.77] |
| Child Ethnicity | 0.39 | .700 | 0.06 | [-0.23, 0.34] |
| Age | 17.31 | < .001 | 0.22 | [0.19, 0.24] |
| Lead Risk | -0.99 | .323 | -0.03 | [-0.10, 0.03] |
| Mid-Posterior Corpus Callosum Volume | 3.46 | .001 | 0.004 | [0.002, 0.01] |

**Note**: This model corresponds to the direct effect of lead risk on performance in the flanker inhibitory control and attention test, controlling for mid-posterior corpus callosal volume, as part of the analyses conducted to investigate indirect associations between lead risk and cognition. The linear mixed-effects model incorporates testing the statistical significance of coefficients against a *t*-distribution. Family Income was a categorical, effects-coded factor, in which the level “Mid” served as the reference level. Family Income was operationally defined as the self-reported combined family income and partitioned into three levels: Low Income: ≤ $50K; Mid Income: $50K-$100K; High Income: ≥ $100K. Sex was also a categorical factor, effect coded with Male/Female as ‑1/+1. Child Ethnicity was also a categorical factor, effect coded with Hispanic/Non-Hispanic as ‑1/+1. Child Race was also a categorical factor, in which “White” served as the reference level. Age, Maximum Parental Education (i.e., highest education level between parents/caregivers), Lead Risk, and Mid-Posterior Corpus Callosum Volume were centered continuous factors. The random effects structure included a random intercept for study site identification number and family identification number. Random effects were restricted to be uncorrelated. Analysis included 8,524 data points. The model accounted for 16.2% of the variance in the data (*R*^2^ = .162, adjusted *R*^2^ = .161).

**Supplementary Table 117. Linear mixed-effects model output for the analysis of lead risk, area deprivation index (ADI), and volume of the thalamus proper, collapsed across hemispheres.**

|  | *t*(8505) | *p* | *b* | 95% CI |
| --- | --- | --- | --- | --- |
| Intercept | 229.34 | < .001 | 7527.65 | [7463.30, 7591.99] |
| Maximum Parental Education | -0.17 | .866 | -0.95 | [-12.00, 10.10] |
| Family Income (High) | -0.54 | .587 | -5.76 | [-26.55, 15.03] |
| Family Income (Low) | -0.58 | .561 | -5.68 | [-24.83, 13.47] |
| Sex | -3.98 | < .001 | -21.01 | [-31.37, -10.65] |
| Child Race (American Indian/Alaska Native) | -0.35 | .726 | -21.61 | [-142.62, 99.40] |
| Child Race (Asian) | -0.44 | .663 | -16.89 | [-92.96, 59.18] |
| Child Race (Black) | -0.34 | .733 | -10.59 | [-71.54, 50.36] |
| Child Race (Native Hawaiian / Pacific Islander) | 0.50 | .617 | 63.45 | [-185.10, 312.00] |
| Child Race (Other) | 0.38 | .704 | 11.38 | [-47.37, 70.13] |
| Child Ethnicity | -0.93 | .352 | -7.05 | [-21.89, 7.79] |
| Age | 6.30 | < .001 | 3.84 | [2.64, 5.03] |
| Lead Risk | 0.56 | .575 | 1.16 | [-2.90, 5.22] |
| ADI | -2.34 | .020 | -6.98 | [-12.84, -1.12] |
| Intracranial Volume | 93.74 | < .001 | 0.004 | [0.004, 0.004] |
| Family Income (High) × Lead Risk | -0.03 | .974 | -0.08 | [-4.82, 4.66] |
| Family Income (Low) × Lead Risk | 0.31 | .758 | 0.84 | [-4.51, 6.19] |
| Family Income (High) × ADI | -0.34 | .731 | -1.16 | [-7.78, 5.46] |
| Family Income (Low) × ADI | -0.98 | .327 | -3.11 | [-9.33, 3.11] |

**Note**: The linear mixed-effects model incorporates testing the statistical significance of coefficients against a *t*-distribution. Family Income was a categorical, effects-coded factor, in which the level “Mid” served as the reference level. Family Income was operationally defined as the self-reported combined family income and partitioned into three levels: Low Income: ≤ $50K; Mid Income: $50K-$100K; High Income: ≥ $100K. Sex was also a categorical factor, effect coded with Male/Female as ‑1/+1. Child Ethnicity was also a categorical factor, effect coded with Hispanic/Non-Hispanic as ‑1/+1. Child Race was also a categorical factor, in which “White” served as the reference level. Age, Maximum Parental Education (i.e., highest education level between parents/caregivers), Lead Risk, Area Deprivation Index (ADI), and Intracranial Volume were centered continuous factors. The random effects structure included a random intercept for magnetic resonance imaging (MRI) scanner serial number and family identification number. Random effects were restricted to be uncorrelated. Analysis included 8,524 data points. The model accounted for 86.0% of the variance in the data (*R*^2^ = .860, adjusted *R*^2^ = .859).

**Supplementary Table 118. Linear mixed-effects model output for the analysis of lead risk, area deprivation index (ADI), and volume of the caudate, collapsed across hemispheres.**

|  | *t*(8505) | *p* | *b* | 95% CI |
| --- | --- | --- | --- | --- |
| Intercept | 94.84 | < .001 | 4024.80 | [3941.61, 4107.99] |
| Maximum Parental Education | 1.92 | .055 | 10.66 | [-0.23, 21.54] |
| Family Income (High) | -1.10 | .269 | -11.53 | [-32.00, 8.93] |
| Family Income (Low) | -0.41 | .683 | -3.93 | [-22.78, 14.92] |
| Sex | 7.63 | < .001 | 39.47 | [29.33, 49.62] |
| Child Race (American Indian/Alaska Native) | -2.43 | .015 | -147.53 | [-266.35, -28.71] |
| Child Race (Asian) | -0.34 | .737 | -12.83 | [-87.76, 62.10] |
| Child Race (Black) | 1.48 | .139 | 45.43 | [-14.75, 105.61] |
| Child Race (Native Hawaiian / Pacific Islander) | 0.05 | .957 | 6.75 | [-239.00, 252.51] |
| Child Race (Other) | 1.53 | .127 | 45.11 | [-12.85, 103.07] |
| Child Ethnicity | 1.17 | .240 | 8.84 | [-5.92, 23.61] |
| Age | -3.51 | < .001 | -2.08 | [-3.24, -0.92] |
| Lead Risk | -1.04 | .297 | -2.16 | [-6.24, 1.91] |
| ADI | -1.31 | .190 | -4.00 | [-9.99, 1.99] |
| Intracranial Volume | 58.46 | < .001 | 0.002 | [0.002, 0.002] |
| Family Income (High) × Lead Risk | 0.96 | .337 | 2.28 | [-2.38, 6.95] |
| Family Income (Low) × Lead Risk | -0.11 | .912 | -0.30 | [-5.56, 4.97] |
| Family Income (High) × ADI | -1.15 | .252 | -3.81 | [-10.33, 2.71] |
| Family Income (Low) × ADI | 0.19 | .847 | 0.60 | [-5.52, 6.72] |

**Note**: The linear mixed-effects model incorporates testing the statistical significance of coefficients against a *t*-distribution. Family Income was a categorical, effects-coded factor, in which the level “Mid” served as the reference level. Family Income was operationally defined as the self-reported combined family income and partitioned into three levels: Low Income: ≤ $50K; Mid Income: $50K-$100K; High Income: ≥ $100K. Sex was also a categorical factor, effect coded with Male/Female as ‑1/+1. Child Ethnicity was also a categorical factor, effect coded with Hispanic/Non-Hispanic as ‑1/+1. Child Race was also a categorical factor, in which “White” served as the reference level. Age, Maximum Parental Education (i.e., highest education level between parents/caregivers), Lead Risk, Area Deprivation Index (ADI), and Intracranial Volume were centered continuous factors. The random effects structure included a random intercept for magnetic resonance imaging (MRI) scanner serial number and family identification number. Random effects were restricted to be uncorrelated. Analysis included 8,524 data points. The model accounted for 76.4% of the variance in the data (*R*^2^ = .764, adjusted *R*^2^ = .763).

**Supplementary Table 119. Linear mixed-effects model output for the analysis of lead risk, area deprivation index (ADI), and volume of the putamen, collapsed across hemispheres.**

|  | *t*(8505) | *p* | *b* | 95% CI |
| --- | --- | --- | --- | --- |
| Intercept | 148.62 | < .001 | 5886.22 | [5808.58, 5963.85] |
| Maximum Parental Education | -0.09 | .930 | -0.60 | [-13.93, 12.73] |
| Family Income (High) | -0.68 | .499 | -8.66 | [-33.76, 16.43] |
| Family Income (Low) | -1.57 | .116 | -18.52 | [-41.63, 4.60] |
| Sex | -9.57 | < .001 | -60.80 | [-73.26, -48.35] |
| Child Race (American Indian/Alaska Native) | -1.57 | .117 | -116.76 | [-262.68, 29.16] |
| Child Race (Asian) | 3.12 | .002 | 146.10 | [54.23, 237.97] |
| Child Race (Black) | -1.77 | .077 | -66.48 | [-140.18, 7.22] |
| Child Race (Native Hawaiian / Pacific Islander) | 0.31 | .754 | 48.16 | [-252.87, 349.18] |
| Child Race (Other) | -0.05 | .962 | -1.72 | [-72.77, 69.33] |
| Child Ethnicity | -1.43 | .152 | -13.10 | [-31.00, 4.80] |
| Age | -3.85 | < .001 | -2.81 | [-4.24, -1.38] |
| Lead Risk | -1.12 | .264 | -2.79 | [-7.69, 2.11] |
| ADI | -1.83 | .067 | -6.60 | [-13.66, 0.47] |
| Intracranial Volume | 51.06 | < .001 | 0.002 | [0.002, 0.003] |
| Family Income (High) × Lead Risk | -0.001 | .999 | -0.003 | [-5.72, 5.71] |
| Family Income (Low) × Lead Risk | 1.50 | .134 | 4.94 | [-1.51, 11.39] |
| Family Income (High) × ADI | -1.94 | .053 | -7.90 | [-15.90, 0.09] |
| Family Income (Low) × ADI | -0.07 | .944 | -0.27 | [-7.77, 7.23] |

**Note**: The linear mixed-effects model incorporates testing the statistical significance of coefficients against a *t*-distribution. Family Income was a categorical, effects-coded factor, in which the level “Mid” served as the reference level. Family Income was operationally defined as the self-reported combined family income and partitioned into three levels: Low Income: ≤ $50K; Mid Income: $50K-$100K; High Income: ≥ $100K. Sex was also a categorical factor, effect coded with Male/Female as ‑1/+1. Child Ethnicity was also a categorical factor, effect coded with Hispanic/Non-Hispanic as ‑1/+1. Child Race was also a categorical factor, in which “White” served as the reference level. Age, Maximum Parental Education (i.e., highest education level between parents/caregivers), Lead Risk, Area Deprivation Index (ADI), and Intracranial Volume were centered continuous factors. The random effects structure included a random intercept for magnetic resonance imaging (MRI) scanner serial number and family identification number. Random effects were restricted to be uncorrelated. Analysis included 8,524 data points. The model accounted for 75.3% of the variance in the data (*R*^2^ = .753, adjusted *R*^2^ = .753).

**Supplementary Table 120. Linear mixed-effects model output for the analysis of lead risk, area deprivation index (ADI), and volume of the pallidum, collapsed across hemispheres.**

|  | *t*(8505) | *p* | *b* | 95% CI |
| --- | --- | --- | --- | --- |
| Intercept | 111.54 | < .001 | 1783.94 | [1752.59, 1815.29] |
| Maximum Parental Education | 1.30 | .193 | 2.67 | [-1.35, 6.69] |
| Family Income (High) | 0.32 | .750 | 1.23 | [-6.33, 8.78] |
| Family Income (Low) | -0.67 | .500 | -2.39 | [-9.36, 4.57] |
| Sex | -10.64 | < .001 | -20.67 | [-24.47, -16.86] |
| Child Race (American Indian/Alaska Native) | 0.02 | .984 | 0.46 | [-43.62, 44.53] |
| Child Race (Asian) | 0.11 | .910 | 1.59 | [-26.03, 29.20] |
| Child Race (Black) | 0.83 | .408 | 9.31 | [-12.74, 31.35] |
| Child Race (Native Hawaiian / Pacific Islander) | 0.10 | .919 | 4.66 | [-84.77, 94.08] |
| Child Race (Other) | -0.35 | .723 | -3.84 | [-25.05, 17.38] |
| Child Ethnicity | -1.85 | .065 | -5.15 | [-10.62, 0.31] |
| Age | -7.48 | < .001 | -1.69 | [-2.13, -1.24] |
| Lead Risk | -0.18 | .856 | -0.14 | [-1.64, 1.37] |
| ADI | -1.89 | .059 | -2.14 | [-4.35, 0.08] |
| Intracranial Volume | 53.03 | < .001 | 0.001 | [0.001, 0.001] |
| Family Income (High) × Lead Risk | 1.18 | .239 | 1.03 | [-0.69, 2.76] |
| Family Income (Low) × Lead Risk | 0.49 | .621 | 0.49 | [-1.46, 2.44] |
| Family Income (High) × ADI | -1.25 | .210 | -1.54 | [-3.95, 0.87] |
| Family Income (Low) × ADI | 0.04 | .970 | 0.04 | [-2.22, 2.31] |

**Note**: The linear mixed-effects model incorporates testing the statistical significance of coefficients against a *t*-distribution. Family Income was a categorical, effects-coded factor, in which the level “Mid” served as the reference level. Family Income was operationally defined as the self-reported combined family income and partitioned into three levels: Low Income: ≤ $50K; Mid Income: $50K-$100K; High Income: ≥ $100K. Sex was also a categorical factor, effect coded with Male/Female as ‑1/+1. Child Ethnicity was also a categorical factor, effect coded with Hispanic/Non-Hispanic as ‑1/+1. Child Race was also a categorical factor, in which “White” served as the reference level. Age, Maximum Parental Education (i.e., highest education level between parents/caregivers), Lead Risk, Area Deprivation Index (ADI), and Intracranial Volume were centered continuous factors. The random effects structure included a random intercept for magnetic resonance imaging (MRI) scanner serial number and family identification number. Random effects were restricted to be uncorrelated. Analysis included 8,524 data points. The model accounted for 64.7% of the variance in the data (*R*^2^ = .647, adjusted *R*^2^ = .646).

**Supplementary Table 121. Linear mixed-effects model output for the analysis of lead risk, area deprivation index (ADI), and volume of the hippocampus, collapsed across hemispheres.**

|  | *t*(8505) | *p* | *b* | 95% CI |
| --- | --- | --- | --- | --- |
| Intercept | 187.66 | < .001 | 4089.28 | [4046.56, 4132.00] |
| Maximum Parental Education | 2.41 | .016 | 9.61 | [1.79, 17.43] |
| Family Income (High) | 2.21 | .027 | 16.66 | [1.91, 31.41] |
| Family Income (Low) | -2.23 | .026 | -15.44 | [-29.02, -1.86] |
| Sex | -3.35 | .001 | -12.50 | [-19.81, -5.19] |
| Child Race (American Indian/Alaska Native) | 0.69 | .491 | 30.18 | [-55.71, 116.07] |
| Child Race (Asian) | -0.36 | .717 | -9.98 | [-63.96, 44.00] |
| Child Race (Black) | -4.62 | < .001 | -101.83 | [-145.08, -58.58] |
| Child Race (Native Hawaiian / Pacific Islander) | 1.45 | .147 | 130.92 | [-46.08, 307.91] |
| Child Race (Other) | -1.68 | .093 | -35.88 | [-77.68, 5.92] |
| Child Ethnicity | -0.90 | .366 | -4.72 | [-14.98, 5.53] |
| Age | 1.89 | .059 | 0.81 | [-0.03, 1.66] |
| Lead Risk | 1.79 | .073 | 2.54 | [-0.24, 5.32] |
| ADI | -1.60 | .110 | -3.16 | [-7.02, 0.71] |
| Intracranial Volume | 62.53 | < .001 | 0.002 | [0.002, 0.002] |
| Family Income (High) × Lead Risk | -0.32 | .751 | -0.54 | [-3.89, 2.81] |
| Family Income (Low) × Lead Risk | 1.32 | .186 | 2.55 | [-1.23, 6.33] |
| Family Income (High) × ADI | 0.49 | .626 | 1.17 | [-3.53, 5.86] |
| Family Income (Low) × ADI | -1.16 | .247 | -2.60 | [-7.01, 1.80] |

**Note**: The linear mixed-effects model incorporates testing the statistical significance of coefficients against a *t*-distribution. Family Income was a categorical, effects-coded factor, in which the level “Mid” served as the reference level. Family Income was operationally defined as the self-reported combined family income and partitioned into three levels: Low Income: ≤ $50K; Mid Income: $50K-$100K; High Income: ≥ $100K. Sex was also a categorical factor, effect coded with Male/Female as ‑1/+1. Child Ethnicity was also a categorical factor, effect coded with Hispanic/Non-Hispanic as ‑1/+1. Child Race was also a categorical factor, in which “White” served as the reference level. Age, Maximum Parental Education (i.e., highest education level between parents/caregivers), Lead Risk, Area Deprivation Index (ADI), and Intracranial Volume were centered continuous factors. The random effects structure included a random intercept for magnetic resonance imaging (MRI) scanner serial number and family identification number. Random effects were restricted to be uncorrelated. Analysis included 8,524 data points. The model accounted for 78.4% of the variance in the data (*R*^2^ = .784, adjusted *R*^2^ = .784).

**Supplementary Table 122. Linear mixed-effects model output for the analysis of lead risk, area deprivation index (ADI), and volume of the amygdala, collapsed across hemispheres.**

|  | *t*(8505) | *p* | *b* | 95% CI |
| --- | --- | --- | --- | --- |
| Intercept | 101.23 | < .001 | 1589.20 | [1558.43, 1619.97] |
| Maximum Parental Education | 0.95 | .340 | 1.89 | [-2.00, 5.79] |
| Family Income (High) | 0.83 | .407 | 3.09 | [-4.22, 10.40] |
| Family Income (Low) | -1.15 | .249 | -3.97 | [-10.70, 2.77] |
| Sex | -12.71 | < .001 | -23.92 | [-27.61, -20.23] |
| Child Race (American Indian/Alaska Native) | 1.89 | .058 | 41.24 | [-1.43, 83.90] |
| Child Race (Asian) | 0.96 | .335 | 13.14 | [-13.58, 39.86] |
| Child Race (Black) | -3.38 | .001 | -36.75 | [-58.07, -15.43] |
| Child Race (Native Hawaiian / Pacific Islander) | 0.10 | .918 | 4.55 | [-81.88, 90.99] |
| Child Race (Other) | -1.61 | .106 | -16.90 | [-37.41, 3.62] |
| Child Ethnicity | -1.06 | .288 | -2.87 | [-8.16, 2.42] |
| Age | 1.31 | .191 | 0.29 | [-0.14, 0.71] |
| Lead Risk | 1.27 | .204 | 0.94 | [-0.51, 2.40] |
| ADI | -3.00 | .003 | -3.29 | [-5.43, -1.14] |
| Intracranial Volume | 54.88 | < .001 | 0.001 | [0.001, 0.001] |
| Family Income (High) × Lead Risk | -0.84 | .400 | -0.72 | [-2.39, 0.95] |
| Family Income (Low) × Lead Risk | 1.13 | .257 | 1.09 | [-0.80, 2.98] |
| Family Income (High) × ADI | -0.07 | .946 | -0.08 | [-2.41, 2.25] |
| Family Income (Low) × ADI | -1.21 | .228 | -1.35 | [-3.54, 0.84] |

**Note**: The linear mixed-effects model incorporates testing the statistical significance of coefficients against a *t*-distribution. Family Income was a categorical, effects-coded factor, in which the level “Mid” served as the reference level. Family Income was operationally defined as the self-reported combined family income and partitioned into three levels: Low Income: ≤ $50K; Mid Income: $50K-$100K; High Income: ≥ $100K. Sex was also a categorical factor, effect coded with Male/Female as ‑1/+1. Child Ethnicity was also a categorical factor, effect coded with Hispanic/Non-Hispanic as ‑1/+1. Child Race was also a categorical factor, in which “White” served as the reference level. Age, Maximum Parental Education (i.e., highest education level between parents/caregivers), Lead Risk, Area Deprivation Index (ADI), and Intracranial Volume were centered continuous factors. The random effects structure included a random intercept for magnetic resonance imaging (MRI) scanner serial number and family identification number. Random effects were restricted to be uncorrelated. Analysis included 8,524 data points. The model accounted for 73.0% of the variance in the data (*R*^2^ = .730, adjusted *R*^2^ = .729).

**Supplementary Table 123. Linear mixed-effects model output for the analysis of lead risk, area deprivation index (ADI), and volume of the accumbens area, collapsed across hemispheres.**

|  | *t*(8505) | *p* | *b* | 95% CI |
| --- | --- | --- | --- | --- |
| Intercept | 57.00 | < .001 | 597.44 | [576.89, 617.98] |
| Maximum Parental Education | -0.29 | .771 | -0.28 | [-2.20, 1.63] |
| Family Income (High) | 0.30 | .762 | 0.56 | [-3.04, 4.15] |
| Family Income (Low) | -0.89 | .375 | -1.50 | [-4.81, 1.81] |
| Sex | -0.39 | .694 | -0.36 | [-2.17, 1.45] |
| Child Race (American Indian/Alaska Native) | 0.90 | .368 | 9.64 | [-11.32, 30.59] |
| Child Race (Asian) | -2.04 | .041 | -13.68 | [-26.81, -0.54] |
| Child Race (Black) | 0.15 | .881 | 0.80 | [-9.69, 11.29] |
| Child Race (Native Hawaiian / Pacific Islander) | 0.67 | .506 | 14.45 | [-28.09, 56.99] |
| Child Race (Other) | -1.46 | .145 | -7.50 | [-17.59, 2.59] |
| Child Ethnicity | -0.12 | .901 | -0.17 | [-2.77, 2.44] |
| Age | -6.36 | < .001 | -0.68 | [-0.89, -0.47] |
| Lead Risk | 0.01 | .990 | 0.005 | [-0.71, 0.72] |
| ADI | -2.50 | .012 | -1.35 | [-2.41, -0.29] |
| Intracranial Volume | 53.56 | < .001 | 0.0004 | [0.0004, 0.0004] |
| Family Income (High) × Lead Risk | -0.09 | .928 | -0.04 | [-0.86, 0.78] |
| Family Income (Low) × Lead Risk | 1.51 | .130 | 0.72 | [-0.21, 1.64] |
| Family Income (High) × ADI | 0.52 | .600 | 0.31 | [-0.84, 1.45] |
| Family Income (Low) × ADI | -1.58 | .113 | -0.87 | [-1.95, 0.21] |

**Note**: The linear mixed-effects model incorporates testing the statistical significance of coefficients against a *t*-distribution. Family Income was a categorical, effects-coded factor, in which the level “Mid” served as the reference level. Family Income was operationally defined as the self-reported combined family income and partitioned into three levels: Low Income: ≤ $50K; Mid Income: $50K-$100K; High Income: ≥ $100K. Sex was also a categorical factor, effect coded with Male/Female as ‑1/+1. Child Ethnicity was also a categorical factor, effect coded with Hispanic/Non-Hispanic as ‑1/+1. Child Race was also a categorical factor, in which “White” served as the reference level. Age, Maximum Parental Education (i.e., highest education level between parents/caregivers), Lead Risk, Area Deprivation Index (ADI), and Intracranial Volume were centered continuous factors. The random effects structure included a random intercept for magnetic resonance imaging (MRI) scanner serial number and family identification number. Random effects were restricted to be uncorrelated. Analysis included 8,524 data points. The model accounted for 67.5% of the variance in the data (*R*^2^ = .675, adjusted *R*^2^ = .674).

**Supplementary Table 124. Linear mixed-effects model output for the analysis of lead risk, area deprivation index (ADI), and volume of the ventral diencephalon, collapsed across hemispheres.**

|  | *t*(8505) | *p* | *b* | 95% CI |
| --- | --- | --- | --- | --- |
| Intercept | 155.67 | < .001 | 3936.10 | [3886.54, 3985.67] |
| Maximum Parental Education | 1.32 | .188 | 4.36 | [-2.14, 10.86] |
| Family Income (High) | 1.20 | .231 | 7.47 | [-4.74, 19.68] |
| Family Income (Low) | -0.93 | .352 | -5.34 | [-16.60, 5.91] |
| Sex | -1.98 | .048 | -6.19 | [-12.32, -0.06] |
| Child Race (American Indian/Alaska Native) | 0.76 | .447 | 27.59 | [-43.59, 98.78] |
| Child Race (Asian) | -2.76 | .006 | -63.00 | [-107.67, -18.33] |
| Child Race (Black) | 3.74 | < .001 | 68.17 | [32.45, 103.88] |
| Child Race (Native Hawaiian / Pacific Islander) | -1.27 | .204 | -93.95 | [-239.06, 51.16] |
| Child Race (Other) | 1.73 | .083 | 30.41 | [-3.96, 64.79] |
| Child Ethnicity | -0.13 | .900 | -0.56 | [-9.39, 8.27] |
| Age | 10.11 | < .001 | 3.66 | [2.95, 4.37] |
| Lead Risk | 1.86 | .063 | 2.30 | [-0.13, 4.73] |
| ADI | -2.15 | .032 | -3.92 | [-7.49, -0.34] |
| Intracranial Volume | 86.07 | < .001 | 0.002 | [0.002, 0.002] |
| Family Income (High) × Lead Risk | 0.13 | .894 | 0.19 | [-2.60, 2.98] |
| Family Income (Low) × Lead Risk | 0.84 | .403 | 1.34 | [-1.80, 4.49] |
| Family Income (High) × ADI | 0.88 | .381 | 1.74 | [-2.15, 5.64] |
| Family Income (Low) × ADI | -0.63 | .527 | -1.18 | [-4.84, 2.48] |

**Note**: The linear mixed-effects model incorporates testing the statistical significance of coefficients against a *t*-distribution. Family Income was a categorical, effects-coded factor, in which the level “Mid” served as the reference level. Family Income was operationally defined as the self-reported combined family income and partitioned into three levels: Low Income: ≤ $50K; Mid Income: $50K-$100K; High Income: ≥ $100K. Sex was also a categorical factor, effect coded with Male/Female as ‑1/+1. Child Ethnicity was also a categorical factor, effect coded with Hispanic/Non-Hispanic as ‑1/+1. Child Race was also a categorical factor, in which “White” served as the reference level. Age, Maximum Parental Education (i.e., highest education level between parents/caregivers), Lead Risk, Area Deprivation Index (ADI), and Intracranial Volume were centered continuous factors. The random effects structure included a random intercept for magnetic resonance imaging (MRI) scanner serial number and family identification number. Random effects were restricted to be uncorrelated. Analysis included 8,524 data points. The model accounted for 80.8% of the variance in the data (*R*^2^ = .808, adjusted *R*^2^ = .808).

**Supplementary Table 125. Linear mixed-effects model output for the analysis of lead risk, area deprivation index (ADI), and volume of the cerebellum cortex, collapsed across hemispheres.**

|  | *t*(8505) | *p* | *b* | 95% CI |
| --- | --- | --- | --- | --- |
| Intercept | 140.33 | < .001 | 52930.23 | [52190.87, 53669.59] |
| Maximum Parental Education | 3.40 | .001 | 175.14 | [74.14, 276.14] |
| Family Income (High) | 0.65 | .517 | 62.81 | [-127.21, 252.83] |
| Family Income (Low) | -1.55 | .121 | -138.54 | [-313.54, 36.47] |
| Sex | -18.77 | < .001 | -893.08 | [-986.37, -799.79] |
| Child Race (American Indian/Alaska Native) | 0.15 | .884 | 82.10 | [-1018.26, 1182.46] |
| Child Race (Asian) | -2.08 | .038 | -738.00 | [-1433.93, -42.06] |
| Child Race (Black) | -0.29 | .771 | -83.27 | [-643.41, 476.87] |
| Child Race (Native Hawaiian / Pacific Islander) | -0.88 | .378 | -1032.38 | [-3326.14, 1261.37] |
| Child Race (Other) | 2.14 | .032 | 590.06 | [50.37, 1129.74] |
| Child Ethnicity | 5.76 | < .001 | 401.86 | [265.02, 538.70] |
| Age | -1.56 | .118 | -8.43 | [-19.00, 2.15] |
| Lead Risk | 0.70 | .484 | 13.47 | [-24.28, 51.22] |
| ADI | -2.87 | .004 | -81.09 | [-136.56, -25.61] |
| Intracranial Volume | 56.86 | < .001 | 0.02 | [0.02, 0.02] |
| Family Income (High) × Lead Risk | 1.75 | .080 | 38.65 | [-4.68, 81.98] |
| Family Income (Low) × Lead Risk | -0.33 | .742 | -8.21 | [-57.08, 40.66] |
| Family Income (High) × ADI | -0.76 | .446 | -23.52 | [-84.03, 36.99] |
| Family Income (Low) × ADI | 1.21 | .226 | 35.10 | [-21.67, 91.86] |

**Note**: The linear mixed-effects model incorporates testing the statistical significance of coefficients against a *t*-distribution. Family Income was a categorical, effects-coded factor, in which the level “Mid” served as the reference level. Family Income was operationally defined as the self-reported combined family income and partitioned into three levels: Low Income: ≤ $50K; Mid Income: $50K-$100K; High Income: ≥ $100K. Sex was also a categorical factor, effect coded with Male/Female as ‑1/+1. Child Ethnicity was also a categorical factor, effect coded with Hispanic/Non-Hispanic as ‑1/+1. Child Race was also a categorical factor, in which “White” served as the reference level. Age, Maximum Parental Education (i.e., highest education level between parents/caregivers), Lead Risk, Area Deprivation Index (ADI), and Intracranial Volume were centered continuous factors. The random effects structure included a random intercept for magnetic resonance imaging (MRI) scanner serial number and family identification number. Random effects were restricted to be uncorrelated. Analysis included 8,524 data points. The model accounted for 86.2% of the variance in the data (*R*^2^ = .862, adjusted *R*^2^ = .862).

**Supplementary Table 126. Linear mixed-effects model output for the analysis of lead risk, area deprivation index (ADI), and volume of cerebellar white matter, collapsed across hemispheres.**

|  | *t*(8505) | *p* | *b* | 95% CI |
| --- | --- | --- | --- | --- |
| Intercept | 133.01 | < .001 | 17092.08 | [16840.19, 17343.97] |
| Maximum Parental Education | 1.96 | .050 | 42.98 | [-0.08, 86.04] |
| Family Income (High) | 0.50 | .615 | 20.79 | [-60.26, 101.85] |
| Family Income (Low) | -1.49 | .135 | -56.90 | [-131.55, 17.76] |
| Sex | -2.81 | .005 | -57.47 | [-97.52, -17.42] |
| Child Race (American Indian/Alaska Native) | 1.09 | .276 | 261.74 | [-208.96, 732.43] |
| Child Race (Asian) | -1.33 | .184 | -201.29 | [-498.11, 95.53] |
| Child Race (Black) | 2.63 | .009 | 319.33 | [80.91, 557.75] |
| Child Race (Native Hawaiian / Pacific Islander) | -0.91 | .364 | -452.06 | [-1427.27, 523.15] |
| Child Race (Other) | 0.70 | .484 | 82.06 | [-147.83, 311.94] |
| Child Ethnicity | 1.54 | .124 | 45.42 | [-12.38, 103.22] |
| Age | 7.20 | < .001 | 16.83 | [12.25, 21.41] |
| Lead Risk | -0.25 | .806 | -1.99 | [-17.82, 13.85] |
| ADI | -0.28 | .777 | -3.30 | [-26.16, 19.55] |
| Intracranial Volume | 50.17 | < .001 | 0.01 | [0.01, 0.01] |
| Family Income (High) × Lead Risk | 0.46 | .644 | 4.35 | [-14.11, 22.82] |
| Family Income (Low) × Lead Risk | 0.52 | .601 | 5.56 | [-15.27, 26.39] |
| Family Income (High) × ADI | -1.16 | .244 | -15.33 | [-41.14, 10.47] |
| Family Income (Low) × ADI | 0.46 | .643 | 5.73 | [-18.49, 29.95] |

**Note**: The linear mixed-effects model incorporates testing the statistical significance of coefficients against a *t*-distribution. Family Income was a categorical, effects-coded factor, in which the level “Mid” served as the reference level. Family Income was operationally defined as the self-reported combined family income and partitioned into three levels: Low Income: ≤ $50K; Mid Income: $50K-$100K; High Income: ≥ $100K. Sex was also a categorical factor, effect coded with Male/Female as ‑1/+1. Child Ethnicity was also a categorical factor, effect coded with Hispanic/Non-Hispanic as ‑1/+1. Child Race was also a categorical factor, in which “White” served as the reference level. Age, Maximum Parental Education (i.e., highest education level between parents/caregivers), Lead Risk, Area Deprivation Index (ADI), and Intracranial Volume were centered continuous factors. The random effects structure included a random intercept for magnetic resonance imaging (MRI) scanner serial number and family identification number. Random effects were restricted to be uncorrelated. Analysis included 8,524 data points. The model accounted for 78.0% of the variance in the data (*R*^2^ = .780, adjusted *R*^2^ = .779).

**Supplementary Table 127. Linear mixed-effects model output for the analysis of lead risk, area deprivation index (ADI), and volume of the brain stem.**

|  | *t*(8505) | *p* | *b* | 95% CI |
| --- | --- | --- | --- | --- |
| Intercept | 139.00 | < .001 | 19164.70 | [18894.42, 19434.98] |
| Maximum Parental Education | 1.33 | .185 | 24.20 | [-11.55, 59.95] |
| Family Income (High) | 1.31 | .189 | 45.08 | [-22.16, 112.32] |
| Family Income (Low) | -1.05 | .292 | -33.29 | [-95.22, 28.64] |
| Sex | -8.89 | < .001 | -150.52 | [-183.72, -117.32] |
| Child Race (American Indian/Alaska Native) | 1.00 | .315 | 199.89 | [-190.10, 589.88] |
| Child Race (Asian) | -2.34 | .019 | -294.26 | [-540.48, -48.05] |
| Child Race (Black) | 3.92 | < .001 | 396.21 | [198.29, 594.13] |
| Child Race (Native Hawaiian / Pacific Islander) | -1.12 | .264 | -461.03 | [-1270.20, 348.14] |
| Child Race (Other) | 1.14 | .252 | 111.35 | [-79.30, 302.00] |
| Child Ethnicity | -0.63 | .527 | -15.63 | [-64.12, 32.85] |
| Age | 12.73 | < .001 | 24.56 | [20.77, 28.34] |
| Lead Risk | 0.58 | .563 | 3.95 | [-9.42, 17.32] |
| ADI | -0.81 | .419 | -8.11 | [-27.78, 11.55] |
| Intracranial Volume | 74.11 | < .001 | 0.01 | [0.01, 0.01] |
| Family Income (High) × Lead Risk | -0.02 | .981 | -0.18 | [-15.52, 15.15] |
| Family Income (Low) × Lead Risk | 2.34 | .019 | 20.63 | [3.33, 37.94] |
| Family Income (High) × ADI | 0.15 | .881 | 1.64 | [-19.78, 23.06] |
| Family Income (Low) × ADI | -0.76 | .447 | -7.80 | [-27.90, 12.30] |

**Note**: The linear mixed-effects model incorporates testing the statistical significance of coefficients against a *t*-distribution. Family Income was a categorical, effects-coded factor, in which the level “Mid” served as the reference level. Family Income was operationally defined as the self-reported combined family income and partitioned into three levels: Low Income: ≤ $50K; Mid Income: $50K-$100K; High Income: ≥ $100K. Sex was also a categorical factor, effect coded with Male/Female as ‑1/+1. Child Ethnicity was also a categorical factor, effect coded with Hispanic/Non-Hispanic as ‑1/+1. Child Race was also a categorical factor, in which “White” served as the reference level. Age, Maximum Parental Education (i.e., highest education level between parents/caregivers), Lead Risk, Area Deprivation Index (ADI), and Intracranial Volume were centered continuous factors. The random effects structure included a random intercept for magnetic resonance imaging (MRI) scanner serial number and family identification number. Random effects were restricted to be uncorrelated. Analysis included 8,524 data points. The model accounted for 85.5% of the variance in the data (*R*^2^ = .855, adjusted *R*^2^ = .855).

**Supplementary Table 128. Linear mixed-effects model output for the analysis of lead risk, area deprivation index (ADI), and volume of the posterior corpus callosum.**

|  | *t*(8505) | *p* | *b* | 95% CI |
| --- | --- | --- | --- | --- |
| Intercept | 90.23 | < .001 | 796.36 | [779.06, 813.66] |
| Maximum Parental Education | 1.01 | .311 | 1.61 | [-1.50, 4.73] |
| Family Income (High) | -0.02 | .986 | -0.05 | [-5.92, 5.82] |
| Family Income (Low) | -0.56 | .573 | -1.55 | [-6.96, 3.85] |
| Sex | 1.99 | .046 | 2.96 | [0.05, 5.86] |
| Child Race (American Indian/Alaska Native) | 0.46 | .644 | 8.06 | [-26.09, 42.22] |
| Child Race (Asian) | 0.72 | .472 | 7.88 | [-13.62, 29.38] |
| Child Race (Black) | -0.04 | .966 | -0.38 | [-17.62, 16.87] |
| Child Race (Native Hawaiian / Pacific Islander) | -0.61 | .543 | -21.88 | [-92.43, 48.68] |
| Child Race (Other) | 0.96 | .338 | 8.13 | [-8.51, 24.78] |
| Child Ethnicity | -1.81 | .070 | -3.82 | [-7.95, 0.31] |
| Age | 7.40 | < .001 | 1.26 | [0.93, 1.60] |
| Lead Risk | -1.91 | .056 | -1.10 | [-2.22, 0.03] |
| ADI | 0.24 | .809 | 0.20 | [-1.39, 1.78] |
| Intracranial Volume | 28.68 | < .001 | 0.0003 | [0.0003, 0.0003] |
| Family Income (High) × Lead Risk | 0.50 | .615 | 0.34 | [-0.99, 1.68] |
| Family Income (Low) × Lead Risk | 0.96 | .338 | 0.74 | [-0.77, 2.24] |
| Family Income (High) × ADI | -0.02 | .981 | -0.02 | [-1.89, 1.85] |
| Family Income (Low) × ADI | -0.72 | .468 | -0.65 | [-2.40, 1.11] |

**Note**: The linear mixed-effects model incorporates testing the statistical significance of coefficients against a *t*-distribution. Family Income was a categorical, effects-coded factor, in which the level “Mid” served as the reference level. Family Income was operationally defined as the self-reported combined family income and partitioned into three levels: Low Income: ≤ $50K; Mid Income: $50K-$100K; High Income: ≥ $100K. Sex was also a categorical factor, effect coded with Male/Female as ‑1/+1. Child Ethnicity was also a categorical factor, effect coded with Hispanic/Non-Hispanic as ‑1/+1. Child Race was also a categorical factor, in which “White” served as the reference level. Age, Maximum Parental Education (i.e., highest education level between parents/caregivers), Lead Risk, Area Deprivation Index (ADI), and Intracranial Volume were centered continuous factors. The random effects structure included a random intercept for magnetic resonance imaging (MRI) scanner serial number and family identification number. Random effects were restricted to be uncorrelated. Analysis included 8,524 data points. The model accounted for 60.2% of the variance in the data (*R*^2^ = .602, adjusted *R*^2^ = .601).

**Supplementary Table 129. Linear mixed-effects model output for the analysis of lead risk, area deprivation index (ADI), and volume of the mid-posterior corpus callosum.**

|  | *t*(8505) | *p* | *b* | 95% CI |
| --- | --- | --- | --- | --- |
| Intercept | 70.67 | < .001 | 376.56 | [366.11, 387.01] |
| Maximum Parental Education | 0.85 | .396 | 0.83 | [-1.08, 2.74] |
| Family Income (High) | 0.07 | .946 | 0.12 | [-3.47, 3.72] |
| Family Income (Low) | -0.63 | .532 | -1.06 | [-4.37, 2.26] |
| Sex | 1.85 | .065 | 1.70 | [-0.10, 3.50] |
| Child Race (American Indian/Alaska Native) | 1.12 | .264 | 11.99 | [-9.03, 33.00] |
| Child Race (Asian) | -0.55 | .584 | -3.68 | [-16.84, 9.48] |
| Child Race (Black) | -1.39 | .164 | -7.46 | [-17.97, 3.05] |
| Child Race (Native Hawaiian / Pacific Islander) | -0.80 | .423 | -17.52 | [-60.35, 25.31] |
| Child Race (Other) | 1.45 | .147 | 7.51 | [-2.64, 17.66] |
| Child Ethnicity | 0.82 | .411 | 1.06 | [-1.46, 3.58] |
| Age | 7.59 | < .001 | 0.81 | [0.60, 1.02] |
| Lead Risk | -2.73 | .006 | -0.95 | [-1.64, -0.27] |
| ADI | 0.18 | .854 | 0.09 | [-0.87, 1.05] |
| Intracranial Volume | 19.26 | < .001 | 0.0001 | [0.0001, 0.0001] |
| Family Income (High) × Lead Risk | -0.10 | .923 | -0.04 | [-0.86, 0.78] |
| Family Income (Low) × Lead Risk | 1.52 | .128 | 0.72 | [-0.21, 1.64] |
| Family Income (High) × ADI | 1.64 | .101 | 0.96 | [-0.19, 2.11] |
| Family Income (Low) × ADI | -1.72 | .086 | -0.94 | [-2.02, 0.13] |

**Note**: The linear mixed-effects model incorporates testing the statistical significance of coefficients against a *t*-distribution. Family Income was a categorical, effects-coded factor, in which the level “Mid” served as the reference level. Family Income was operationally defined as the self-reported combined family income and partitioned into three levels: Low Income: ≤ $50K; Mid Income: $50K-$100K; High Income: ≥ $100K. Sex was also a categorical factor, effect coded with Male/Female as ‑1/+1. Child Ethnicity was also a categorical factor, effect coded with Hispanic/Non-Hispanic as ‑1/+1. Child Race was also a categorical factor, in which “White” served as the reference level. Age, Maximum Parental Education (i.e., highest education level between parents/caregivers), Lead Risk, Area Deprivation Index (ADI), and Intracranial Volume were centered continuous factors. The random effects structure included a random intercept for magnetic resonance imaging (MRI) scanner serial number and family identification number. Random effects were restricted to be uncorrelated. Analysis included 8,524 data points. The model accounted for 43.0% of the variance in the data (*R*^2^ = .430, adjusted *R*^2^ = .429).

**Supplementary Table 130. Linear mixed-effects model output for the analysis of lead risk, area deprivation index (ADI), and volume of the central corpus callosum.**

|  | *t*(8505) | *p* | *b* | 95% CI |
| --- | --- | --- | --- | --- |
| Intercept | 66.57 | < .001 | 406.28 | [394.32, 418.24] |
| Maximum Parental Education | -0.60 | .551 | -0.65 | [-2.77, 1.48] |
| Family Income (High) | -0.29 | .774 | -0.58 | [-4.58, 3.41] |
| Family Income (Low) | -0.39 | .693 | -0.74 | [-4.42, 2.94] |
| Sex | 3.35 | .001 | 3.44 | [1.43, 5.44] |
| Child Race (American Indian/Alaska Native) | 0.27 | .786 | 3.23 | [-20.09, 26.54] |
| Child Race (Asian) | 1.64 | .102 | 12.19 | [-2.40, 26.78] |
| Child Race (Black) | 0.77 | .443 | 4.56 | [-7.08, 16.20] |
| Child Race (Native Hawaiian / Pacific Islander) | -1.43 | .153 | -34.51 | [-81.80, 12.78] |
| Child Race (Other) | 1.76 | .078 | 10.10 | [-1.13, 21.32] |
| Child Ethnicity | 1.41 | .160 | 2.04 | [-0.80, 4.88] |
| Age | 5.00 | < .001 | 0.60 | [0.36, 0.83] |
| Lead Risk | -3.12 | .002 | -1.23 | [-2.01, -0.46] |
| ADI | 0.44 | .663 | 0.25 | [-0.86, 1.35] |
| Intracranial Volume | 17.60 | < .001 | 0.0001 | [0.0001, 0.0002] |
| Family Income (High) × Lead Risk | 0.71 | .476 | 0.33 | [-0.58, 1.24] |
| Family Income (Low) × Lead Risk | 0.28 | .777 | 0.15 | [-0.88, 1.18] |
| Family Income (High) × ADI | 1.10 | .270 | 0.72 | [-0.56, 1.99] |
| Family Income (Low) × ADI | -1.63 | .103 | -0.99 | [-2.19, 0.20] |

**Note**: The linear mixed-effects model incorporates testing the statistical significance of coefficients against a *t*-distribution. Family Income was a categorical, effects-coded factor, in which the level “Mid” served as the reference level. Family Income was operationally defined as the self-reported combined family income and partitioned into three levels: Low Income: ≤ $50K; Mid Income: $50K-$100K; High Income: ≥ $100K. Sex was also a categorical factor, effect coded with Male/Female as ‑1/+1. Child Ethnicity was also a categorical factor, effect coded with Hispanic/Non-Hispanic as ‑1/+1. Child Race was also a categorical factor, in which “White” served as the reference level. Age, Maximum Parental Education (i.e., highest education level between parents/caregivers), Lead Risk, Area Deprivation Index (ADI), and Intracranial Volume were centered continuous factors. The random effects structure included a random intercept for magnetic resonance imaging (MRI) scanner serial number and family identification number. Random effects were restricted to be uncorrelated. Analysis included 8,524 data points. The model accounted for 36.9% of the variance in the data (*R*^2^ = .369, adjusted *R*^2^ = .368).

**Supplementary Table 131. Linear mixed-effects model output for the analysis of lead risk, area deprivation index (ADI), and volume of the mid-anterior corpus callosum.**

|  | *t*(8505) | *p* | *b* | 95% CI |
| --- | --- | --- | --- | --- |
| Intercept | 63.05 | < .001 | 434.90 | [421.38, 448.42] |
| Maximum Parental Education | -2.12 | .034 | -2.60 | [-5.00, -0.20] |
| Family Income (High) | -0.15 | .879 | -0.35 | [-4.87, 4.17] |
| Family Income (Low) | 0.19 | .850 | 0.40 | [-3.77, 4.57] |
| Sex | -0.15 | .878 | -0.18 | [-2.45, 2.10] |
| Child Race (American Indian/Alaska Native) | 0.53 | .598 | 7.11 | [-19.30, 33.51] |
| Child Race (Asian) | 2.56 | .010 | 21.60 | [5.08, 38.13] |
| Child Race (Black) | -1.26 | .208 | -8.46 | [-21.65, 4.72] |
| Child Race (Native Hawaiian / Pacific Islander) | -1.35 | .178 | -36.81 | [-90.34, 16.72] |
| Child Race (Other) | 1.66 | .096 | 10.78 | [-1.92, 23.48] |
| Child Ethnicity | 0.79 | .432 | 1.29 | [-1.93, 4.50] |
| Age | 0.05 | .959 | 0.01 | [-0.26, 0.27] |
| Lead Risk | -3.33 | .001 | -1.49 | [-2.37, -0.61] |
| ADI | 0.49 | .626 | 0.31 | [-0.94, 1.56] |
| Intracranial Volume | 23.67 | < .001 | 0.0002 | [0.0002, 0.0002] |
| Family Income (High) × Lead Risk | 0.32 | .750 | 0.17 | [-0.86, 1.20] |
| Family Income (Low) × Lead Risk | 0.63 | .529 | 0.37 | [-0.79, 1.54] |
| Family Income (High) × ADI | 1.55 | .122 | 1.14 | [-0.30, 2.58] |
| Family Income (Low) × ADI | -1.43 | .152 | -0.99 | [-2.34, 0.36] |

**Note**: The linear mixed-effects model incorporates testing the statistical significance of coefficients against a *t*-distribution. Family Income was a categorical, effects-coded factor, in which the level “Mid” served as the reference level. Family Income was operationally defined as the self-reported combined family income and partitioned into three levels: Low Income: ≤ $50K; Mid Income: $50K-$100K; High Income: ≥ $100K. Sex was also a categorical factor, effect coded with Male/Female as ‑1/+1. Child Ethnicity was also a categorical factor, effect coded with Hispanic/Non-Hispanic as ‑1/+1. Child Race was also a categorical factor, in which “White” served as the reference level. Age, Maximum Parental Education (i.e., highest education level between parents/caregivers), Lead Risk, Area Deprivation Index (ADI), and Intracranial Volume were centered continuous factors. The random effects structure included a random intercept for magnetic resonance imaging (MRI) scanner serial number and family identification number. Random effects were restricted to be uncorrelated. Analysis included 8,524 data points. The model accounted for 39.9% of the variance in the data (*R*^2^ = .399, adjusted *R*^2^ = .398).

**Supplementary Table 132. Linear mixed-effects model output for the analysis of lead risk, area deprivation index (ADI), and volume of the anterior corpus callosum.**

|  | *t*(8505) | *p* | *b* | 95% CI |
| --- | --- | --- | --- | --- |
| Intercept | 79.25 | < .001 | 778.61 | [759.35, 797.86] |
| Maximum Parental Education | -0.28 | .781 | -0.44 | [-3.57, 2.68] |
| Family Income (High) | -0.43 | .667 | -1.29 | [-7.17, 4.59] |
| Family Income (Low) | -0.32 | .752 | -0.87 | [-6.29, 4.55] |
| Sex | 4.24 | < .001 | 6.32 | [3.40, 9.25] |
| Child Race (American Indian/Alaska Native) | -0.79 | .428 | -13.82 | [-48.02, 20.38] |
| Child Race (Asian) | 0.37 | .709 | 4.10 | [-17.43, 25.63] |
| Child Race (Black) | 1.43 | .154 | 12.57 | [-4.70, 29.84] |
| Child Race (Native Hawaiian / Pacific Islander) | -1.12 | .263 | -40.21 | [-110.68, 30.26] |
| Child Race (Other) | 2.34 | .019 | 19.86 | [3.22, 36.50] |
| Child Ethnicity | 1.07 | .286 | 2.30 | [-1.92, 6.52] |
| Age | -0.21 | .834 | -0.04 | [-0.37, 0.30] |
| Lead Risk | -1.07 | .286 | -0.63 | [-1.79, 0.53] |
| ADI | 1.57 | .116 | 1.35 | [-0.33, 3.04] |
| Intracranial Volume | 37.20 | < .001 | 0.0004 | [0.0004, 0.0004] |
| Family Income (High) × Lead Risk | 1.30 | .193 | 0.89 | [-0.45, 2.23] |
| Family Income (Low) × Lead Risk | -0.61 | .540 | -0.47 | [-1.99, 1.04] |
| Family Income (High) × ADI | -0.69 | .489 | -0.66 | [-2.54, 1.21] |
| Family Income (Low) × ADI | 0.08 | .939 | 0.07 | [-1.69, 1.83] |

**Note**: The linear mixed-effects model incorporates testing the statistical significance of coefficients against a *t*-distribution. Family Income was a categorical, effects-coded factor, in which the level “Mid” served as the reference level. Family Income was operationally defined as the self-reported combined family income and partitioned into three levels: Low Income: ≤ $50K; Mid Income: $50K-$100K; High Income: ≥ $100K. Sex was also a categorical factor, effect coded with Male/Female as ‑1/+1. Child Ethnicity was also a categorical factor, effect coded with Hispanic/Non-Hispanic as ‑1/+1. Child Race was also a categorical factor, in which “White” served as the reference level. Age, Maximum Parental Education (i.e., highest education level between parents/caregivers), Lead Risk, Area Deprivation Index (ADI), and Intracranial Volume were centered continuous factors. The random effects structure included a random intercept for magnetic resonance imaging (MRI) scanner serial number and family identification number. Random effects were restricted to be uncorrelated. Analysis included 8,524 data points. The model accounted for 63.4% of the variance in the data (*R*^2^ = .634, adjusted *R*^2^ = .633).

**Supplementary Table 133. Linear mixed-effects model output for the analysis of lead risk, area deprivation index (ADI), and volume of the lateral ventricles, collapsed across hemispheres.**

|  | *t*(8505) | *p* | *b* | 95% CI |
| --- | --- | --- | --- | --- |
| Intercept | 23.57 | < .001 | 4659.79 | [4272.19, 5047.39] |
| Maximum Parental Education | -0.79 | .430 | -25.01 | [-87.17, 37.14] |
| Family Income (High) | -0.50 | .620 | -29.50 | [-146.27, 87.26] |
| Family Income (Low) | 0.55 | .581 | 30.30 | [-77.32, 137.93] |
| Sex | 1.69 | .092 | 50.73 | [-8.22, 109.67] |
| Child Race (American Indian/Alaska Native) | -0.46 | .643 | -161.33 | [-843.22, 520.56] |
| Child Race (Asian) | -0.58 | .561 | -126.69 | [-553.41, 300.03] |
| Child Race (Black) | 1.61 | .108 | 279.29 | [-60.98, 619.56] |
| Child Race (Native Hawaiian / Pacific Islander) | 0.51 | .613 | 355.62 | [-1023.54, 1734.78] |
| Child Race (Other) | -0.85 | .396 | -141.79 | [-469.32, 185.74] |
| Child Ethnicity | 0.31 | .753 | 13.50 | [-70.60, 97.60] |
| Age | 2.43 | .015 | 8.49 | [1.63, 15.35] |
| Lead Risk | 1.30 | .194 | 15.27 | [-7.78, 38.33] |
| ADI | -0.86 | .390 | -14.75 | [-48.38, 18.89] |
| Intracranial Volume | 30.92 | < .001 | 0.01 | [0.01, 0.01] |
| Family Income (High) × Lead Risk | 1.13 | .257 | 15.41 | [-11.24, 42.05] |
| Family Income (Low) × Lead Risk | -0.43 | .664 | -6.67 | [-36.77, 23.44] |
| Family Income (High) × ADI | -1.19 | .234 | -22.63 | [-59.87, 14.62] |
| Family Income (Low) × ADI | 0.92 | .358 | 16.43 | [-18.59, 51.45] |

**Note**: The linear mixed-effects model incorporates testing the statistical significance of coefficients against a *t*-distribution. Family Income was a categorical, effects-coded factor, in which the level “Mid” served as the reference level. Family Income was operationally defined as the self-reported combined family income and partitioned into three levels: Low Income: ≤ $50K; Mid Income: $50K-$100K; High Income: ≥ $100K. Sex was also a categorical factor, effect coded with Male/Female as ‑1/+1. Child Ethnicity was also a categorical factor, effect coded with Hispanic/Non-Hispanic as ‑1/+1. Child Race was also a categorical factor, in which “White” served as the reference level. Age, Maximum Parental Education (i.e., highest education level between parents/caregivers), Lead Risk, Area Deprivation Index (ADI), and Intracranial Volume were centered continuous factors. The random effects structure included a random intercept for magnetic resonance imaging (MRI) scanner serial number and family identification number. Random effects were restricted to be uncorrelated. Analysis included 8,524 data points. The model accounted for 40.2% of the variance in the data (*R*^2^ = .402, adjusted *R*^2^ = .400).

**Supplementary Table 134. Linear mixed-effects model output for the analysis of lead risk, area deprivation index (ADI), and volume of the inferior lateral ventricles, collapsed across hemispheres.**

|  | *t*(8505) | *p* | *b* | 95% CI |
| --- | --- | --- | --- | --- |
| Intercept | 24.74 | < .001 | 309.57 | [285.04, 334.10] |
| Maximum Parental Education | -1.58 | .114 | -2.61 | [-5.85, 0.63] |
| Family Income (High) | 0.68 | .498 | 2.10 | [-3.97, 8.18] |
| Family Income (Low) | 0.15 | .877 | 0.44 | [-5.16, 6.04] |
| Sex | -4.13 | < .001 | -6.49 | [-9.58, -3.41] |
| Child Race (American Indian/Alaska Native) | -0.30 | .765 | -5.42 | [-40.94, 30.09] |
| Child Race (Asian) | 0.93 | .352 | 10.54 | [-11.64, 32.72] |
| Child Race (Black) | -1.60 | .109 | -14.41 | [-32.05, 3.23] |
| Child Race (Native Hawaiian / Pacific Islander) | 0.84 | .402 | 30.43 | [-40.81, 101.67] |
| Child Race (Other) | -1.25 | .212 | -10.80 | [-27.77, 6.16] |
| Child Ethnicity | -2.47 | .014 | -5.55 | [-9.95, -1.15] |
| Age | 1.54 | .124 | 0.28 | [-0.08, 0.64] |
| Lead Risk | 1.16 | .245 | 0.72 | [-0.49, 1.93] |
| ADI | -0.60 | .546 | -0.55 | [-2.33, 1.23] |
| Intracranial Volume | 12.87 | < .001 | 0.0002 | [0.0001, 0.0002] |
| Family Income (High) × Lead Risk | 0.03 | .975 | 0.02 | [-1.37, 1.41] |
| Family Income (Low) × Lead Risk | -1.51 | .132 | -1.21 | [-2.77, 0.36] |
| Family Income (High) × ADI | 0.14 | .890 | 0.14 | [-1.80, 2.08] |
| Family Income (Low) × ADI | 0.56 | .573 | 0.52 | [-1.30, 2.35] |

**Note**: The linear mixed-effects model incorporates testing the statistical significance of coefficients against a *t*-distribution. Family Income was a categorical, effects-coded factor, in which the level “Mid” served as the reference level. Family Income was operationally defined as the self-reported combined family income and partitioned into three levels: Low Income: ≤ $50K; Mid Income: $50K-$100K; High Income: ≥ $100K. Sex was also a categorical factor, effect coded with Male/Female as ‑1/+1. Child Ethnicity was also a categorical factor, effect coded with Hispanic/Non-Hispanic as ‑1/+1. Child Race was also a categorical factor, in which “White” served as the reference level. Age, Maximum Parental Education (i.e., highest education level between parents/caregivers), Lead Risk, Area Deprivation Index (ADI), and Intracranial Volume were centered continuous factors. The random effects structure included a random intercept for magnetic resonance imaging (MRI) scanner serial number and family identification number. Random effects were restricted to be uncorrelated. Analysis included 8,524 data points. The model accounted for 30.2% of the variance in the data (*R*^2^ = .302, adjusted *R*^2^ = .300).

**Supplementary Table 135. Linear mixed-effects model output for the analysis of lead risk, area deprivation index (ADI), and volume of the 3^rd^ ventricle.**

|  | *t*(8505) | *p* | *b* | 95% CI |
| --- | --- | --- | --- | --- |
| Intercept | 47.45 | < .001 | 755.47 | [724.26, 786.68] |
| Maximum Parental Education | -1.17 | .242 | -3.07 | [-8.21, 2.07] |
| Family Income (High) | -0.44 | .662 | -2.15 | [-11.81, 7.51] |
| Family Income (Low) | 2.08 | .038 | 9.44 | [0.54, 18.34] |
| Sex | 0.21 | .834 | 0.52 | [-4.33, 5.37] |
| Child Race (American Indian/Alaska Native) | 0.14 | .887 | 4.10 | [-52.24, 60.43] |
| Child Race (Asian) | 1.79 | .073 | 32.28 | [-3.04, 67.61] |
| Child Race (Black) | 1.21 | .227 | 17.41 | [-10.82, 45.63] |
| Child Race (Native Hawaiian / Pacific Islander) | -0.37 | .713 | -21.54 | [-136.23, 93.16] |
| Child Race (Other) | -0.89 | .374 | -12.32 | [-39.50, 14.87] |
| Child Ethnicity | -0.18 | .856 | -0.64 | [-7.58, 6.30] |
| Age | 0.29 | .770 | 0.08 | [-0.48, 0.65] |
| Lead Risk | 1.88 | .060 | 1.82 | [-0.08, 3.73] |
| ADI | -1.08 | .280 | -1.52 | [-4.29, 1.24] |
| Intracranial Volume | 31.88 | < .001 | 0.001 | [0.001, 0.001] |
| Family Income (High) × Lead Risk | -0.90 | .369 | -1.01 | [-3.21, 1.19] |
| Family Income (Low) × Lead Risk | -0.16 | .871 | -0.21 | [-2.69, 2.28] |
| Family Income (High) × ADI | 0.24 | .811 | 0.38 | [-2.70, 3.45] |
| Family Income (Low) × ADI | -0.72 | .472 | -1.06 | [-3.95, 1.83] |

**Note**: The linear mixed-effects model incorporates testing the statistical significance of coefficients against a *t*-distribution. Family Income was a categorical, effects-coded factor, in which the level “Mid” served as the reference level. Family Income was operationally defined as the self-reported combined family income and partitioned into three levels: Low Income: ≤ $50K; Mid Income: $50K-$100K; High Income: ≥ $100K. Sex was also a categorical factor, effect coded with Male/Female as ‑1/+1. Child Ethnicity was also a categorical factor, effect coded with Hispanic/Non-Hispanic as ‑1/+1. Child Race was also a categorical factor, in which “White” served as the reference level. Age, Maximum Parental Education (i.e., highest education level between parents/caregivers), Lead Risk, Area Deprivation Index (ADI), and Intracranial Volume were centered continuous factors. The random effects structure included a random intercept for magnetic resonance imaging (MRI) scanner serial number and family identification number. Random effects were restricted to be uncorrelated. Analysis included 8,524 data points. The model accounted for 47.3% of the variance in the data (*R*^2^ = .473, adjusted *R*^2^ = .471).

**Supplementary Table 136. Linear mixed-effects model output for the analysis of lead risk, area deprivation index (ADI), and volume of the 4^th^ ventricle.**

|  | *t*(8505) | *p* | *b* | 95% CI |
| --- | --- | --- | --- | --- |
| Intercept | 46.64 | < .001 | 1790.75 | [1715.48, 1866.01] |
| Maximum Parental Education | 3.38 | .001 | 23.59 | [9.92, 37.26] |
| Family Income (High) | 1.05 | .291 | 13.86 | [-11.90, 39.62] |
| Family Income (Low) | 0.30 | .766 | 3.59 | [-20.13, 27.32] |
| Sex | -8.45 | < .001 | -55.26 | [-68.08, -42.44] |
| Child Race (American Indian/Alaska Native) | -0.73 | .467 | -55.68 | [-205.79, 94.42] |
| Child Race (Asian) | 0.43 | .666 | 20.78 | [-73.48, 115.05] |
| Child Race (Black) | 0.44 | .659 | 16.98 | [-58.50, 92.45] |
| Child Race (Native Hawaiian / Pacific Islander) | 1.03 | .301 | 162.66 | [-145.60, 470.91] |
| Child Race (Other) | -1.50 | .133 | -55.88 | [-128.75, 16.99] |
| Child Ethnicity | -0.79 | .428 | -7.30 | [-25.37, 10.76] |
| Age | 1.70 | .089 | 1.28 | [-0.20, 2.77] |
| Lead Risk | 1.09 | .274 | 2.74 | [-2.17, 7.64] |
| ADI | -2.24 | .025 | -7.87 | [-14.77, -0.97] |
| Intracranial Volume | 17.74 | < .001 | 0.001 | [0.001, 0.001] |
| Family Income (High) × Lead Risk | 3.16 | .002 | 9.45 | [3.59, 15.31] |
| Family Income (Low) × Lead Risk | 0.55 | .582 | 1.86 | [-4.75, 8.47] |
| Family Income (High) × ADI | -0.42 | .676 | -1.75 | [-9.95, 6.45] |
| Family Income (Low) × ADI | -1.46 | .143 | -5.75 | [-13.45, 1.95] |

**Note**: The linear mixed-effects model incorporates testing the statistical significance of coefficients against a *t*-distribution. Family Income was a categorical, effects-coded factor, in which the level “Mid” served as the reference level. Family Income was operationally defined as the self-reported combined family income and partitioned into three levels: Low Income: ≤ $50K; Mid Income: $50K-$100K; High Income: ≥ $100K. Sex was also a categorical factor, effect coded with Male/Female as ‑1/+1. Child Ethnicity was also a categorical factor, effect coded with Hispanic/Non-Hispanic as ‑1/+1. Child Race was also a categorical factor, in which “White” served as the reference level. Age, Maximum Parental Education (i.e., highest education level between parents/caregivers), Lead Risk, Area Deprivation Index (ADI), and Intracranial Volume were centered continuous factors. The random effects structure included a random intercept for magnetic resonance imaging (MRI) scanner serial number and family identification number. Random effects were restricted to be uncorrelated. Analysis included 8,524 data points. The model accounted for 50.3% of the variance in the data (*R*^2^ = .503, adjusted *R*^2^ = .501).

**Supplementary Table 137. Linear mixed-effects model output for the analysis of lead risk, area deprivation index (ADI), and subcortical gray matter volume.**

|  | *t*(8505) | *p* | *b* | 95% CI |
| --- | --- | --- | --- | --- |
| Intercept | 224.91 | < .001 | 60383.38 | [59857.10, 60909.67] |
| Maximum Parental Education | 1.51 | .132 | 53.82 | [-16.13, 123.78] |
| Family Income (High) | 0.10 | .917 | 6.99 | [-124.57, 138.56] |
| Family Income (Low) | -1.95 | .051 | -120.63 | [-241.81, 0.56] |
| Sex | -6.50 | < .001 | -216.32 | [-281.56, -151.07] |
| Child Race (American Indian/Alaska Native) | -0.86 | .391 | -334.11 | [-1098.11, 429.89] |
| Child Race (Asian) | 0.19 | .849 | 46.76 | [-434.86, 528.38] |
| Child Race (Black) | -0.85 | .395 | -167.71 | [-554.42, 219.00] |
| Child Race (Native Hawaiian / Pacific Islander) | 0.48 | .635 | 382.70 | [-1196.16, 1961.57] |
| Child Race (Other) | 0.15 | .883 | 28.01 | [-344.44, 400.45] |
| Child Ethnicity | -0.90 | .366 | -43.77 | [-138.67, 51.14] |
| Age | 0.22 | .830 | 0.82 | [-6.64, 8.28] |
| Lead Risk | 0.10 | .921 | 1.33 | [-24.83, 27.49] |
| ADI | -3.14 | .002 | -61.56 | [-100.04, -23.08] |
| Intracranial Volume | 113.72 | < .001 | 0.03 | [0.03, 0.03] |
| Family Income (High) × Lead Risk | 0.25 | .802 | 3.84 | [-26.17, 33.85] |
| Family Income (Low) × Lead Risk | 1.35 | .177 | 23.31 | [-10.56, 57.18] |
| Family Income (High) × ADI | -1.13 | .259 | -24.12 | [-66.03, 17.80] |
| Family Income (Low) × ADI | -0.75 | .454 | -15.04 | [-54.38, 24.30] |

**Note**: The linear mixed-effects model incorporates testing the statistical significance of coefficients against a *t*-distribution. Family Income was a categorical, effects-coded factor, in which the level “Mid” served as the reference level. Family Income was operationally defined as the self-reported combined family income and partitioned into three levels: Low Income: ≤ $50K; Mid Income: $50K-$100K; High Income: ≥ $100K. Sex was also a categorical factor, effect coded with Male/Female as ‑1/+1. Child Ethnicity was also a categorical factor, effect coded with Hispanic/Non-Hispanic as ‑1/+1. Child Race was also a categorical factor, in which “White” served as the reference level. Age, Maximum Parental Education (i.e., highest education level between parents/caregivers), Lead Risk, Area Deprivation Index (ADI), and Intracranial Volume were centered continuous factors. The random effects structure included a random intercept for magnetic resonance imaging (MRI) scanner serial number and family identification number. Random effects were restricted to be uncorrelated. Analysis included 8,524 data points. The model accounted for 91.3% of the variance in the data (*R*^2^ = .913, adjusted *R*^2^ = .913).
