# Supplementary material for: Risk of lead exposure, subcortical brain structure, and cognition in a large cohort of 9- to 10-year-old children
Source: PLoS One. 2021 Oct 14;16(10):e0258469. doi: 10.1371/journal.pone.0258469 (PMC8516269; doi:10.1371/journal.pone.0258469)
Supplement: Attachment — Submitted filename: ResponseToReviewers_22Jan2021.docx [file pone.0258469.s002.docx]

**Additional Editor (E) Comments:**

1. **The paper is well-written, although sometimes a little too terse, and more information would be helpful for the reader.**
   a. Thank you to the editor for this suggestion. A series of details have been added throughout the manuscript, as described throughout the rest of the responses below.
2. **The authors refer to secondary analyses in which ADI replaces lead risk. What is the association between the lead risk score and ADI? What happens if the authors include both the lead risk score and ADI in the same analyses instead of replacing lead risk with ADI?**
   a. We have added a sentence to the Results section noting the correlation between ADI and both lead risk and housing age. We have also respecified the ADI and housing-age analyses as “sensitivity” analyses, rather than “secondary” analyses. Additionally, per this recommendation, we re-ran the regression analyses with both ADI*Family Income and Lead Risk*Family Income interactions in the same model. The significant associations between lead risk and mid-anterior, central, and mid-posterior corpus callosal volumes continued to pass FDR correction. Accordingly, the following was added to the “Accounting for Neighborhood Disadvantage” section: “*Further, when ADI and lead risk (and their interactions with family income) were included in the same model, the FDR-corrected associations between lead risk and mid-anterior (r_p_ = ‑0.036 [‑0.047, ‑0.025]), central (r_p_ = ‑0.034 [‑0.045, ‑0.023]), and mid-posterior corpus callosal volumes (r_p_ = ‑0.030 [‑0.040, ‑0.019]) were maintained (q < .05), as were the FDR-corrected associations between ADI and subcortical gray-matter (r_p_ = ‑0.034 [‑0.045, ‑0.023]), amygdalar (r_p_ = ‑0.033 [‑0.043, ‑0.022]), and cerebellar cortical volumes (r_p_ = ‑0.031 [‑0.042, ‑0.020]) (q < .05) (Tables 117-137 in S1 Appendix”* (**p. 18, Lines 412-419**). Accordingly, the corresponding statistical output tables have been added to Appendix S1 of the supporting information.
3. **The findings in Table 2 indicate larger ventricles in the presence of greater lead—the need to control for smaller ICV is important and should probably be included in the findings presented in Table and in the text.**
   a. We’d like to thank the editor for noting the importance of ICV in these analyses. As specified in the Methods sections (**pp. 8-10**), the analyses in which the criterion was subcortical volume included ICV as a covariate, even for the non-significant associations of lead risk or ADI (such as the associations with ventricular volume). For the reader’s benefit, we have added the following sentence to the **Table 2 caption**: “Analyses controlled for age, sex, parental education, race, ethnicity, family income, intracranial volume, and the interaction between family income and either lead risk or ADI.”
4. **The major limitation of the paper is the potential overlap between lead risk including housing age vs. ADI, a metric of neighborhood SES disadvantage. Although the authors attempt to discriminate between them, this is not entirely convincing. Another limitation that should be repeated in the Conclusions is that these findings are based on a community-related risk rather than on individual risk.**
   a. A paragraph has been added to the newly included “Limitations and Future Directions” section describing relationships between ADI, lead risk, and housing age, as well as community-level versus individual-level risk (**pp. 25-27**).
5. **Lines 130+ and later 314+ and wherever referring to these tests: The names of the cognitive tests used should be cited properly by specifying name of test and capitalizing words appropriately (e.g., Picture Vocabulary Test; Flanker test, etc.)**
   a. This edit has been made throughout the manuscript, and we are appreciative of the editor’s recommendation.
6. **Line 185 Why wasn’t ICV included as a covariate? Figure 1 indicates that ICV was a covariate. There appears to be an inconsistency here—please explain or correct.**
   a. We thank the editor for raising this point. In the first set of analyses of lead risk and brain volume, in order to determine which regional volumes were associated with lead risk (i.e., in which the criterion for analysis was regional volume), we controlled for ICV, allowing us to separate whether greater lead risk was uniquely associated with subcortical volumes. Not including ICV in these analyses would have basically prevented us from knowing if lead risk was associated with smaller regional volumes or just smaller overall brain size. However, in these subsequent analyses, the ultimate criterion was cognitive test score, in that we tested whether the association between lead risk and cognitive test score was mediated by subcortical volume. In the total and direct effect testing of the association between lead risk and cognitive score, we did not control for ICV, in accordance with our past research. Thus, in order to accurately conduct mediation analyses in which the models to measure the indirect effect only included the additional mediator variable rather than a mediator variable *and* a new covariate (i.e., ICV), we did not control for ICV in these analyses. We have added a sentence to **page 10**: “*Accordingly, because these analyses ultimately tested whether the association between lead risk and cognitive test score was mediated by subcortical volume, in which the criterion was cognitive test score and not subcortical volume, ICV was not included as a covariate for these analyses of cognitive performance.*”
7. **Title and in lines 364 and elsewhere in Discussion, where age range is given: change “9-10-year-old” to “9- to 10-year-old”.**
   a. This edit has been made in title and throughout the discussion in accordance with the editor’s recommendation.
8. **Lines 440-441 needs to add not only “how” but “when” lead exposure occurs. The authors acknowledge that they could not distinguish between pre- and postnatal exposure and thus timing of exposure should be repeated here.**
   a. On **pages 25-27**, we have divided the final paragraphs into “Limitations & Future Directions” and “Conclusion” sections. The “Limitations and Future Directions” section has also been expanded upon and now includes discussion of the “how” and “when” of lead exposure, per the editor’s request (**pp. 25-27**)

**Comments to the Author: Reviewer #1 (R1)**

1. **Abstract: Page 7, line 52: “The association of lead exposure risk on cognitive performance…” should rather read “The association of lead exposure risk with cognitive performance…”**
   a. This sentence was edited in accordance with the reviewer’s recommendation (**Abstract**).
2. **Methods, Page 11, line 111: “Ethnical” should be corrected to “ethical”.**
   a. This word was corrected in accordance with the reviewer’s correction (**p. 6, Line 113**).
3. **It would be helpful for the reader for the authors to give a summary of the data collection methods (NIH toolbox, and MRI acquisition and processing), even though these have been described elsewhere.**
   a. Additional details have been added about the specific versions used for the NIH Toolbox, and a general description of cross-task data collection methods for the NIH Toolbox has been added (**pp. 7-8**). Additional details have also been provided for the MRI acquisition and processing (**pp. 7-8**). We also provided an additional link to an ABCD-Study-provided PDF that outlines all the neuroimaging parameters across the scanners used at different sites.
4. **Since this was a multisite study, it would be helpful to know how comparable the data (both in terms of acquisition and outcome measurements) was across the different sites, particularly given that the primary lead exposure risk measure was, as I understand it, dependent to some extent on geographical location. Were the exposure risk metrics similarly distributed across the cohorts at the different sites?**
   a. We have added specification that all data-collection sites follow similar experimental protocols, with an added citation of Auchter et al. (2018) from a special issue of *Developmental Cognitive Neuroscience* on the ABCD Study. The reviewer also noted geographic differences in lead-risk scores. Because we have previously published site-by-site distributions, which we specified in the manuscript, we have added the following sentence to the Methods section: “*Briefly, for some sites, the lead-risk scores were uniformly distributed, while other sites showed modalities at either lower, intermediate, and/or higher lead-risk scores, thereby reflecting geographic differences in risk of exposure [39]*” (**p. 6, Lines 121-124**).
5. **What was the rationale behind the use of the specific confounders included in the analyses? Were these confirmed in the current cohort to be associated with lead exposure risk and/or the outcomes used?**
   a. We have included the following sentence in the Methods section addressing the rationale for including the specific covariates used in analyses: “*In accordance with previous research analyzing associations between brain structure and socioeconomic and/or environmental conditions [28, 45, 46], we controlled for children’s age, sex, race, ethnicity, maximum parental education, and family income.*” (**p. 8, Lines 255-256**)
6. **The authors state that they controlled for ICV. Was this associated with lead exposure risk in this cohort?**
   a. The primary reason for controlling for ICV initially was for the sake of the subcortical volume analyses. Specifically, it was important to know whether any differences in subcortical volume were due to differences in brain size (here, operationally defined as ICV). However, we thank the reviewer for the suggestion to look at how ICV was correlated with lead risk. Accordingly, in our Methods section, we included the following: “*Analyses of subcortical data also controlled for intracranial volume (ICV), which was inversely associated with both lead risk, Spearman’s rho (ρ) = ‑0.11, p < .001, and ADI, ρ = ‑0.11, p < .001.*” (**p. 8, Lines 258-260**)
7. **Results, Table 1: the authors state that the children in the analyses did not differ appreciably in key sociodemographic indicators compared to the entire ABCD cohort (page 16, lines 205-206). Was this determined by statistically comparing the two? If so the statistical results should be presented in the table for each indicator.**
   a. We thank the reviewer for this comment and the careful reading/review of our manuscript. In our manuscript, our statement that the demographic indicators in the entire cohort vs. the analyzed sample “did not differ appreciably” was based on observed comparisons between the proportions of participants of each demographic within each factor (e.g., sex, ethnicity). Traditionally, comparing the distribution of participants across different levels of a factor is done through chi-squared goodness-of-fit tests, which works particularly well for samples that are smaller than the sample analyzed here. More specifically, chi-squared tests are very sensitive to sample size, in that, with larger and larger sample sizes, it is very easy to find statistically significant distributional differences across a factor even though such differences, proportionately, are not meaningful. For example, when running chi-squared tests on the demographic indicators in Table 1, the tests for all factors (except sex) are statistically significant, indicating that the observed counts (i.e., the “Sample with Complete Data” column in Table 1) substantially differ from predicted counts (i.e., if 8,524 participants were proportioned in accordance with the percentages data in the “Release 2.0.1” column). However, the percentages (%) / proportion data tell a different story, as they are quite similar between the two columns.

To illustrate via worked-out example using the ethnicity data, there were 1,640 Hispanic participants and 6,884 non-Hispanic participants in the analyzed sample (i.e., the observed counts). 20.3% of ABCD is Hispanic, while 78.4% is not Hispanic. However, of the participants with non-missing data for ethnicity, 20.6% are Hispanic; 79.4% are not Hispanic. If that same distribution across ABCD was present in the analyzed sample, then we would predict there to be 1,753 Hispanic participants and 6,772 non-Hispanic participants (i.e., the predicted counts). Chi-squared tests would then reveal significant differences between the observed and predicted counts, *p* = .003. However, the differences with respect to proportion Hispanic versus not Hispanic are quite similar. Indeed, if we were to scale the counts by a factor of 100 (i.e., all counts divided by 100) and re-run the chi-squared tests, then the observed and predicted counts do not differ, *p* = .763. I contrast, if we were to multiply all of the original counts by 100, then there would be even greater differences between the observed and predicted counts, *p* < 10e-200. Thus, despite equivalent proportional distributions in all cases, larger samples are vulnerable to statistically significant differences that are not meaningful. Further, after additional research and review of the literature, we were unable to find any sample-size corrections for chi-squared tests for large samples (i.e., instead, many statistical tests instead have corrections for small samples). Accordingly, we believe that including statistical comparisons (e.g., chi-squared tests) for Table 1 would not be informative to the reader and may even cause greater confusion, as they would not reflect the actual proportional distributions, which more effectively capture demographical distribution. Therefore, we have maintained our original statement in the manuscript, “Children included in our analyses did not appreciably differ in key sociodemographic indicators compared with the entire ABCD cohort (Table 1).” (**p. 11, Lines 310-311**).

8. **Discussion: The findings are placed in the context of the previous literature, both of lead exposure and of exposure to other neurotoxic substances, and their implications are clearly discussed. Some study limitations are addressed although the possibility of other toxic exposures known to affect brain volume (particularly pre-natally) is not specifically mentioned and should be addressed.**
   a. As described above, we have split up our discussion to now have a “Limitations and Future Directions” section (**pp. 25-27**). From our initial submission, our manuscript includes a paragraph discussing how exposure to other neurotoxicants is associated with brain structure. This passage includes exposure that occurs both pre- and postnatally. In this revision, we have substantially expanded on our discussion of limitations/future directions, including passages on exposures to other neurotoxicants and environments that may synergistically influence brain and cognitive development.
