# Supplementary material for: Risk of lead exposure, subcortical brain structure, and cognition in a large cohort of 9- to 10-year-old children
Source: PLoS One. 2021 Oct 14;16(10):e0258469. doi: 10.1371/journal.pone.0258469 (PMC8516269; doi:10.1371/journal.pone.0258469)
Supplement: Attachment — Submitted filename: Response to Reviewers 13 Apr 2021.docx [file pone.0258469.s003.docx]

**Editor**

1. **There continue to be important questions raised about the resubmission, as indicated by the questions and comments by the additional reviewer, particularly regarding whether there is a direct measure of blood or urine lead, even for a subset of the ABCD sample, available. This would strengthen the conclusion that it is lead and not other aspects of the poor housing that are actually responsible for the outcomes reported here. The relations between ADI and both lead risk and housing age that I inquired about were apparently not so strong as to suggest that the ADI and lead risk are one and the same and suggest that they were making relatively independent contributions. However, these analyses were based on lead “risk” and not a direct measure of lead exposure. As noted by the second reviewer, concluding that lead is the cause of the effects is overstated. Please respond to all of the points raised by this reviewer and modify the manuscript accordingly.**
   a. We agree that the neighborhood-level lead risk measure is only a surrogate marker for lead exposure. We reviewed the manuscript and replaced “effect” with “associations”. We also verified that we refer to “risk” of exposure rather than actual exposure throughout the manuscript.

**Reviewer #2**

1. **The primary concern with the manuscript is therefore that lead exposure is modeled rather than measured. Given that the confidence with which it is written with lead as the cause is very overstated. It needs to be tempered, particularly since the need for an MR scanner means that ABCD children are overrepresented as living in urban areas with more advanced health care systems and these urban areas may be, by definition, higher in lead risk. Lead may certainly be playing a role, but other factors likely are as well (perhaps synergistically with lead since the model likely captures multiple toxic exposures associated with poor, segregated housing).**
   b. We appreciate the reviewer’s comment and agree that we are using risk scores in contrast to blood lead concentrations. Indeed, the Methods section includes the sentence, “*Currently, there are no blood-lead data for ABCD participants*” (**p. 6, Lines 108-109**). Still, we believe our analyses are informative and provide insight into the relationships between neurocognitive development and factors associated with neurotoxicant exposure. Accordingly, our Discussion also includes the following sentence: “*There are no endogenous lead-exposure data yet in ABCD, but our study suggests that lead-exposure risk is associated with corpus callosal morphology*” (**p. 22, Lines 421-423**).

We also added the following sentence to the Methods section: “*Lead-risk scores were previously shown to be highly associated with childhood lead exposure in children (Marshall et al., 2020; D. C. Wheeler, Jones, Schootman, & Nelson, 2019). For instance, we previously validated that these lead-risk scores were positively associated with both the rate of elevated blood-lead levels across 13 states and 2 cities at the census-tract level and the geometric mean of census-tract-level blood-lead levels in the state of Maryland (Marshall et al., 2020)*” (**p. 6, Lines 119-123**).

Further, as noted by the reviewer, it is unlikely that the ABCD participants were only exposed to lead. Accordingly, our Discussion now includes the following sentences: “*As individuals are rarely exposed to isolated chemicals (but to mixtures of chemicals) (Cory-Slechta, 2005), the incorporation of multiple data sources reflecting ‘mixtures’ of environmental health disparities may offer substantial insight into the collective and synergistic factors to target in environmental remediation interventions. Accordingly, upon collection of bodily lead data in ABCD, our future research will involve analyses of both chemical and ‘environmental-disparity mixtures’ to both study developmental trajectories more comprehensively and evaluate the relative strengths of the associations between adolescent development and other sources of disparity (e.g., air pollution, residential segregation)*” (**p. 27, Lines 541-548**).

As noted by the reviewer, the ABCD data collection sites were predominantly in urban areas. Still, the recruitment areas for these sites do well to encompass a non-negligible proportion of 9-to-10-year-olds in the US, which we have noted in an added sentence to the Discussion: “*Similarly, even though ABCD’s data collection sites are primarily in metropolitan areas, the recruitment areas of these 21 sites represent at least 20% of the 9-to-10-year-old US population (Garavan et al., 2018)*” (**p. 26, Lines 510-512**).

As noted in E.1, we thoroughly reviewed the manuscript and have altered the wording of any claims that imply causation, specifically using words such as “associations” rather than “effects, and we have also reviewed the manuscript and have edited any wording to clarify that these analyses involve “risk” of exposure rather than actual exposure as measured by blood or urine collection (or other biospecimens).

2. **Has the model been tested in a subset of ABCD children? A percentage of them must have been screened for blood lead by their physician. Is there a correlation in that subset? Was that sort of validation attempted?**
   a. We thank the reviewer for this comment, as it pertains to one of our future goals of our research. Indeed, one passage of our discussion is, “*There are no endogenous lead-exposure data yet in ABCD, but our study suggests that lead-exposure risk is associated with corpus callosal morphology*” (**p. 22, Lines 421-423**).

We now have a sentence in our Discussion that reads, “*The ABCD Study does not yet have endogenous lead-exposure data in its participants, but it has been actively collecting address histories for its participants since birth, which, when completed, will facilitate understanding of the critical developmental periods of lead neurotoxicity vulnerability*” (**pp. 25-26, Lines 502-508**).

We have also added a sentence to the Conclusion section: “*ABCD is also exploring the collection and analysis of blood lead levels to gauge how well they are correlated with neighborhood-level lead-risk estimates*” (**p. 27, Lines 556-558**).

Thus, we have noted that further research in this domain will allow us to validate these lead-risk scores against current blood lead levels in ABCD children and past lead levels from deciduous tooth analysis. The reviewer also commented on past lead screening by physicians. The ABCD Study does not yet have such data, but we have added the following sentence to our Conclusion: “*ABCD is also working to incorporate electronic health records as part of its dataset, which may also help elucidate these relationships via past lead screening results*” (**pp. 27-28, Lines 558-559**).

3. **While it is possible that lead risk is the cause of the results, in the absence of a measure of lead exposure and without some form of validating the model in ABCD population, this is very speculative. The way it is written ascribes too much of the findings to Lead and not to the correlates of poor, older housing which likely can’t be disentangled easily, especially without a measurement of blood lead. The wording should be more tempered.**
   a. Please see responses to E.1 and R2.1. We agree that neighborhood level of risk is an imperfect measure of lead exposure. Still, numerous studies have consistently shown that age of housing is a significant predictor of lead exposure using blood lead concentrations (e.g., Akkus & Ozdenerol, 2014; Benson et al., 2017; Berg, Kuhn, & Van Dyke, 2017; Binns et al., 1994; Gibson, Fisher, Clonch, MacDonald, & Cook, 2020; Haan, Gerson, & Zishka, 1996; Haley & Talbot, 2004; Kaufmann, Clouse, Olson, & Matte, 2000; Lanphear, Byrd, Auinger, & Schaffer, 1998; Litaker, Kippes, Gallagher, & O'Connor, 2000; Miranda, Dolinoy, & Overstreet, 2002; Pirkle et al., 1998; Sargent, Bailey, Simon, Blake, & Dalton, 1997; Sargent et al., 1995; Schultz, Morara, Buxton, & Weintraub, 2017; W. Wheeler & Brown, 2013).
4. **Many of the studies listed as representing low level lead poisoning took place in the 1980’s and 1990s when low level was defined as <10 ug/dL which is not the case for today. By today’s standards, these studies would represent highly exposed populations. Please revise this text to note that the definition of low-level lead poisoning was higher then.**
   a. We appreciate the reviewer’s concern regarding how it is important to properly contextualize our data. With regard to the reviewer’s comment regarding low versus high levels of lead poisoning, our specific discussion of the relationships between “low levels” of exposure and cognition stem from 2 relatively recent papers, one from 2012 and the other from 2018, respectively: “… *even at low levels of exposure, IQ was shown to be associated with concurrent blood-lead levels in 7- to 14-year-olds (Lucchini et al., 2012; Menezes-Filho et al., 2018)*” (**p. 25, Lines 494-495**).

To address the considerable declines in blood lead levels over the past several decades and that the criterion for elevated exposure has similarly decreased, while also reflecting evidence that blood lead levels are still relatively high in some children in the United States, we have added a sentence to the Discussion: “*Indeed, while average blood lead levels have substantially declined over the past several decades, a 2021 study has estimated that nearly 400,000 1-to-11-year-olds in 2011-2016 had blood-lead levels exceeding the CDC’s reference level of 5 µg/dL (Egan, Cornwell, Courtney, & Ettinger, 2021)*” (**p. 24, Lines 458-461**).

5. **Is there an address history that allows for adjusting for moves? If not, add that to limitations.**
   a. We thank the reviewer for this comment. The ABCD Study is working to compile address histories for its nearly 12,000 participants. Accordingly, the Limitations section includes the following sentence: “*The ABCD Study does not yet have endogenous lead-exposure data in its participants, but it has been actively collecting address histories for its participants since birth, which, when completed, will facilitate understanding of the critical developmental periods of lead neurotoxicity vulnerability*” (**pp. 25-26, Lines 502-508**).

Further, the Conclusion now includes the following sentences: “*ABCD is also exploring the collection and analysis of blood lead levels to gauge how well* *they are correlated with neighborhood-level lead-risk estimates. ABCD is also working to incorporate electronic health records as part of its dataset, which may also help elucidate these relationships via past lead screening results*” (**pp. 27-28, Lines 554-557**). Specifically, while the ABCD Study is working to include full address histories for its participants, this has not yet been completed given the rigor and effort to complete this task. Even though research has shown that characteristics individuals’ neighborhoods are often relatively stable over time, even when participants move (Knighton, 2018), and even across generations (van Ham, Hedman, Manley, Coulter, & Östh, 2014), we have now noted in the manuscript our intention to analyze such address histories once these data are made available.

6. **Was the NIH toolbox administered concurrent to the MRI? If so, they are cross sectional. That would violate the assumptions of causal mediation analysis.**
   a. The reviewer noted that mediational analyses of cross-sectional data are not viable given such cross-sectional design. As described by Fairchild and McDaniel (2017), this guideline is generally true, but it is possible to run mediational analyses on cross-sectional data given the analytical design. Specifically, cross-sectional mediational analyses are possible if the correct temporal order is established and that alternative temporal orderings are ruled out (Fairchild & McDaniel, 2017). We have edited our Limitations section to now include the following sentence: “*Further, while the potential for reverse causation is inherent to cross-sectional studies, thereby limiting causal inference, it is unlikely that poor cognitive performance elicits altered brain structure, or that altered brain structure induces risk factors of lead exposure here, thus supporting the temporal ordering within our cross-sectional mediational analyses (lead risk 🡪 brain structure 🡪 cognitive performance) (Fairchild & McDaniel, 2017)*” (**p. 26, Lines 510-514**).
7. **This sentence has to be amended “In the current report, the primary residential addresses of 9- to 10-year-old children were used to derive community-based risk estimates of lead exposure, which our past research has shown are valid proxies of exposure [28]. Stating that the authors published a study previously in ABCD using this model is not the same as validation of a model. (lines 478-480). If the lead exposure data were validated in that paper, please state how that was done, why you aren’t showing it in this paper and what the correlation of the model was with blood lead? Otherwise remove this sentence or revise it taking "valid proxies" out.**

As also noted in R2.1, we have also added the following sentence to the Methods section to further describe the lead-risk model developed by Rad Cunningham at the Washington State Department of Health: “*Lead-risk scores were previously shown to be highly associated with childhood lead exposure in children (Marshall et al., 2020; D. C. Wheeler et al., 2019). For instance, we previously validated that these lead-risk scores were positively associated with both the rate of elevated blood-lead levels across 13 states and 2 cities at the census-tract level and the geometric mean of census-tract-level blood-lead levels in the state of Maryland (Marshall et al., 2020)*” (**p. 6, Lines 119-123**). Because these data have been published elsewhere, we chose to provide more detail to describe what we had done previously without replicating the previous report.

**References**

Akkus, C., & Ozdenerol, E. (2014). Exploring childhood lead exposure through GIS: A review of the recent literature. *International Journal of Environmental Research and Public Health, 11*, 6314-6334. doi:10.3390/ijerph110606314

Benson, S. M., Talbott, E. O., Brink, L. L., Wu, C., Sharma, R. K., & Marsh, G. M. (2017). Environmental lead and childhood blood lead levels in US children: NHANES, 1999-2006. *Archives of Environmental & Occupational Health, 72*(2), 70-78. doi:10.1080/19338244.2016.1157454

Berg, K., Kuhn, S., & Van Dyke, M. (2017). Spatial surveillance of childhood lead exposure in a targeted screening state: An application of generalized additive models in Denver, Colorado. *Journal of Public Health Management & Practice, 23*, S79-S92.

Binns, H. J., LeBailly, S. A., Poncher, J., Kinsella, R., Saunders, S. E., & Pediatric Practice Research Group. (1994). Is there lead in the suburbs? Risk assessment in Chicago suburban pediatric practices. *Pediatrics, 93*(2), 164-171.

Cory-Slechta, D. A. (2005). Studying toxicants as single chemicals: Does this strategy adequately identify neurotoxic risk? *NeuroToxicology, 26*(4), 491-510. doi:10.1016/j.neuro.2004.12.007

Egan, K. B., Cornwell, C. R., Courtney, J. G., & Ettinger, A. S. (2021). Blood lead levels in U.S. children ages 1-11 years, 1976-2016. *Environmental Health Perspectives, 129*(3), 037003. doi:10.1289/EHP7932

Fairchild, A. J., & McDaniel, H. L. (2017). Best (but oft-forgotten) practices: mediation analysis. *American Journal of Clinical Nutrition, 105*, 1259-1271. doi:10.3945/ajcn.117.152546

Garavan, H., Bartsch, H., Conway, K., Decastro, A., Goldstein, R. Z., Heeringa, S., . . . Zahs, D. (2018). Recruiting the ABCD sample: Design considerations and procedures. *Developmental Cognitive Neuroscience, 32*, 16-22. doi:10.1016/j.dcn.2018.04.004

Gibson, J. M., Fisher, M., Clonch, A., MacDonald, J. M., & Cook, P. J. (2020). Children drinking private well water have higher blood lead than those with city water. *Proceedings of the National Academy of Sciences, 117*(29), 16898-16907. doi:10.1073/pnas.2002729117

Haan, M. N., Gerson, M., & Zishka, B. A. (1996). Identification of children at risk of lead poisoning: An evaluation of routine pediatric blood lead screening in an HMO-insured population. *Pediatrics, 97*(1), 79-83.

Haley, V. B., & Talbot, T. O. (2004). Geographic analysis of blood lead levels in New York state children born 1994-1997. *Environmental Health Perspectives, 112*, 1577-1582. doi:10.1289/ehp.7053

Kaufmann, R. B., Clouse, T. L., Olson, D. R., & Matte, T. D. (2000). Elevated blood lead levels and blood lead screening among US children aged one to five years: 1988-1994. *Pediatrics, 106*, e79.

Knighton, A. J. (2018). Is a patient's current address of record a reasonable measure of neighborhood deprivation exposure? A case for the use of point in time measures of residence in clinical care. *Health Equity, 2*, 62-69. doi:10.1089/heq.2017.0005

Lanphear, B. P., Byrd, R. S., Auinger, P., & Schaffer, S. J. (1998). Community characteristics associated with elevated blood lead levels in children. *Pediatrics, 101*(2), 264-271.

Litaker, D., Kippes, C. M., Gallagher, T. E., & O'Connor, M. E. (2000). Targeting lead screening: The Ohio lead risk score. *Pediatrics, 106*(5), e69.

Lucchini, R. G., Zoni, S., Guazzetti, S., Bontempi, E., Micheletti, S., Broberg, K., . . . Smith, D. R. (2012). Inverse association of intellectual function with very low blood lead but not with manganese exposure in Italian adolescents. *Environmental Research, 118*, 65-71. doi:10.1016/j.envres.2012.08.003

Marshall, A. T., Betts, S., Kan, E. C., McConnell, R., Lanphear, B. P., & Sowell, E. R. (2020). Association of lead-exposure risk and family income with childhood brain outcomes. *Nature Medicine, 26*, 91-97. doi:10.1038/s41591-019-0713-y

Menezes-Filho, J. A., Carvalho, C. F., Rodrigues, J. L. G., Araújo, C. F. S., dos Santos, N. R., Lima, C. S., . . . Mergler, D. (2018). Environmental co-exposure to lead and manganese and intellectual deficit in school-aged children. *International Journal of Environmental Research and Public Health, 15*, 2418. doi:10.3390/ijerph15112418

Miranda, M. L., Dolinoy, D. C., & Overstreet, M. A. (2002). Mapping for prevention: GIS models for directing childhood lead poisoning prevention programs. *Environmental Health Perspectives, 110*, 947-953.

Pirkle, J. L., Kaufmann, R. B., Brody, D. J., Hickman, T., Gunter, E. W., & Paschal, D. C. (1998). Exposure of the U.S. population to lead, 1991-1994. *Environmental Health Perspectives, 106*(11), 745-790. doi:10.1289/ehp.98106745

Sargent, J. D., Bailey, A., Simon, P., Blake, M., & Dalton, M. A. (1997). Census tract analysis of lead exposure in Rhode Island children. *Environmental Research, 74*, 159-168.

Sargent, J. D., Brown, M. J., Freeman, J. L., Bailey, A., Goodman, D., & Freeman, D. H. (1995). Childhood lead poisoning in Massachusetts communities: Its association with sociodemographic and housing characteristics. *American Journal of Public Health, 85*, 528-534.

Schultz, B. D., Morara, M., Buxton, B. E., & Weintraub, M. (2017). Predicting blood-lead levels amoung U.S. children at the census tract level. *Environmental Justice, 10*, 129-136. doi:10.1089/env.2017.0005

van Ham, M., Hedman, L., Manley, D., Coulter, R., & Östh, J. (2014). Intergenerational transmission of neighbourhood poverty: an analysis of neighbourhood histories of individuals. *Transactions of the Institute of British Geographers, 39*, 402-417. doi:10.1111/tran.12040

Wheeler, D. C., Jones, R. M., Schootman, M., & Nelson, E. J. (2019). Explaining variation in elevated blood lead levels among children in Minnesota using neighborhood socioeconomic variables. *Science of the Total Environment, 650*, 970-977. doi:10.1016/j.scitotenv.2018.09.088

Wheeler, W., & Brown, M. J. (2013). Blood lead levels in children aged 1-5 years - United States, 1999-2010. *Morbidity and Mortality Weekly Report, 62*(13), 245-248.
