# Supplementary material for: Risk of lead exposure, subcortical brain structure, and cognition in a large cohort of 9- to 10-year-old children
Source: PLoS One. 2021 Oct 14;16(10):e0258469. doi: 10.1371/journal.pone.0258469 (PMC8516269; doi:10.1371/journal.pone.0258469)
Supplement: Attachment — Submitted filename: Response to Reviewers 16Sept2021.docx [file pone.0258469.s004.docx]

**Editor**

1. **The interest in and significance of research on effects of lead exposure is major and has many implications for public health. This paper focuses on lead risk but lacks direct biological measures of lead exposure. This important limitation results in the authors’ needing to expand on this limitation rather than concluding with lead exposure (not lead risk) being the primary cause of the developmental problems.  Moreover, the sentence in the Conclusion is incorrect: "Lead exposure may mediate certain aspects of cognitive functioning by diminishing subcortical brain structure, including the anterior splenium (i.e., mid-posterior corpus callosum)." Lead is not the mediator in this sentence but the predictor; regional brain volume is the mediator." This is correctly stated in a sentence towards the end of the previous paragraph.**
   a. We thank the editor for making this comment, as that sentence in the Conclusion of the Abstract was indeed misworded. Accordingly, the Conclusion of the Abstract was edited and now reads, “*Environmental factors related to the risk of lead exposure may be associated with certain aspects of cognitive functioning via diminished subcortical brain structure, including the anterior splenium (i.e., mid-posterior corpus callosum).*” Additionally, in the Conclusion section at the end of the Discussion, the final sentence now reads, “*Until then, this study, which uses neighborhood-level lead-exposure risk, provides potential evidence that cognitive deficits from low-level lead toxicity (and its related environmental factors) may operate by diminishing subcortical brain structure [28]*” (**pp. 28-29, Lines 567-570**).
2. **One reviewer has concluded that the paper is acceptable now. However, the other reviewer points out some important limitations that still need to be addressed. Even though the authors adjusted for environmental factors, statistical control may still be inadequate, particularly given the absence of a biological measure of lead. Thus, as pointed out by the second reviewer, in their discussion, the authors need to embed lead risk into the larger environmental risk factors that contribute to the outcome, of which lead is only one. The authors should, therefore, modify the discussion and conclusions to emphasize the combination of factors that impact on the public health of the children.**
   a. As also described in R2.1, the primary revisions regarded contextualizing risk of lead exposure within other environmental variables. We thank the editor and Reviewer #2 for addressing this comment, as we agree that risk of lead exposure does not operate in a vacuum but is associated with other socioeconomic and demographic factors. Accordingly, we have edited the Limitations section to include the following paragraph, which we believe effectively addresses the greater context of lead exposure: “*As individuals are rarely exposed to isolated chemicals (but to mixtures of chemicals) [104], the incorporation of multiple data sources reflecting “mixtures” of environmental health disparities may also offer substantial insight into the collective and synergistic factors to target in environmental remediation interventions. Simply, the risk of lead exposure does not exist in a vacuum but is associated with past and current practices that have differentially subjected children to such risks. For example, the burden of lead-exposure’s effects is typically greatest in children in the lowest SES families [105-108], a glaring example of environmental injustice [105, 109]. Similarly, Black and Hispanic children tend to have greater mean blood lead levels than white children [5, 110-112] and are more likely than white children to live in homes or regions with greater risks of lead exposure [113-115]. Further, lead-poisoning rates (and, thus, children’s blood-lead levels) are associated with multiple community-level factors [39], including value and age of houses, poverty rates, population density, and percentage of the population who are Black or Hispanic [1, 116], signifying racial residential segregation as a potential explanatory mechanism for lead exposure disparities [117]. While the data in the current manuscript may reflect differences in lead exposure, these differences would then ultimately be due to disparate conditions that initially elicited such differences, thereby focusing any potential intervention efforts on the originating disparities. Indeed, recent research has shown that soil-lead concentrations tended to be elevated in samples taken from historically redlined neighborhoods compared to those in “best” and “desirable” neighborhoods, per zone designations by the 1930’s Home Owners’ Loan Corporation [118]. Ultimately, because we do not currently have endogenous lead-exposure data in our participants, the results related to risk of lead exposure may be alternatively explained (at least partially) by these other systemic and environmental factors. Accordingly, upon collection of bodily lead data and additional geocoded data in ABCD, our future research will involve analyses of both chemical and “environmental-disparity mixtures” to both study developmental trajectories more comprehensively and evaluate the relative strengths of the associations between adolescent development and other sources of disparity (e.g., air pollution, residential segregation)”* (**pp. 27-28, Lines 530-556**). Further, throughout the discussion, we have also edited sentences to not only describe lead-exposure risk, but also its related environmental factors that may also account for our results (**p. 22, Lines 427-428; p. 24, Line 468**).

**Reviewer #2**

1. **Lead-risk scores were previously shown to be highly associated with childhood lead exposure in children (Marshall et al., 2020; D. C. Wheeler, Jones, Schootman, & Nelson, 2019). For instance, we previously validated that these lead-risk scores were positively associated with both the rate of elevated blood-lead levels across 13 states and 2 cities at the census-tract level and the geometric mean of census-tract-level blood-lead levels in the state of Maryland (Marshall et al., 2020)” (p. 6, Lines 119-123) What is described above is an ecological analysis and not a validation study (i.e. the children in the analysis cited for validation may or may not be the children screened for lead). More to the point- the authors need consideration of the issues that correlate with lead exposure that may be playing a bigger role than lead. The reader can decide if they believe the results are due to lead or other correlates of poor housing. You haven’t proven it is lead, and with the limitations you have on measuring lead exposure, can never prove it is lead. Just be transparent about that. It may be lead in part, I don’t dispute that, but there is a lack of consideration of all the issues that go with poor housing- especially racism, economic stress and educational quality, just to name a few. This paper needs greater discussion on the role that segregated housing and redlining is playing as I believe those issues are severely understated and the narrow focus on lead exposure does a disservice to children. Focusing on lead poisoning also misses the big picture which is structural racism. The bulk of these findings are due to the combination of issues (redlining, economic injustice and social stress) and not just lead. In addition, lead poisoning didn’t cause redlining and segregation, it is a consequence of it. Fixing lead exposure won’t fix segregation and its many toxic correlates. Fixing segregation will fix the larger issues, including lead exposure and will have much greater public health impact than just focusing on lead exposure. The discussion should address these issues as give them greater weight.**
   a. We appreciate the reviewer’s comment on validation studies versus ecological studies. Accordingly, when we first introduce the lead-risk and area-deprivation indices in the Methods section, we have altered the wording of the sentence describing the validation of the lead-risk scores. Specifically, the sentence now reads, “*For instance, we previously showed that these lead-risk scores were positively associated with both the rate of elevated blood-lead levels across 13 states and 2 cities at the census-tract level and the geometric mean of census-tract-level blood-lead levels in the state of Maryland [28]”* (**p. 6, Line 125**). Further, as described in the response to E.2, we have edited the Limitations section to include the following paragraph, which we believe effectively addresses the greater context of lead exposure: “*As individuals are rarely exposed to isolated chemicals (but to mixtures of chemicals) [104], the incorporation of multiple data sources reflecting “mixtures” of environmental health disparities may also offer substantial insight into the collective and synergistic factors to target in environmental remediation interventions. Simply, the risk of lead exposure does not exist in a vacuum but is associated with past and current practices that have differentially subjected children to such risks. For example, the burden of lead-exposure’s effects is typically greatest in children in the lowest SES families [105-108], a glaring example of environmental injustice [105, 109]. Similarly, Black and Hispanic children tend to have greater mean blood lead levels than white children [5, 110-112] and are more likely than white children to live in homes or regions with greater risks of lead exposure [113-115]. Further, lead-poisoning rates (and, thus, children’s blood-lead levels) are associated with multiple community-level factors [39], including value and age of houses, poverty rates, population density, and percentage of the population who are Black or Hispanic [1, 116], signifying racial residential segregation as a potential explanatory mechanism for lead exposure disparities [117]. While the data in the current manuscript may reflect differences in lead exposure, these differences would then ultimately be due to disparate conditions that initially elicited such differences, thereby focusing any potential intervention efforts on the originating disparities. Indeed, recent research has shown that soil-lead concentrations tended to be elevated in samples taken from historically redlined neighborhoods compared to those in “best” and “desirable” neighborhoods, per zone designations by the 1930’s Home Owners’ Loan Corporation [118]. Ultimately, because we do not currently have endogenous lead-exposure data in our participants, the results related to risk of lead exposure may be alternatively explained (at least partially) by these other systemic and environmental factors. Accordingly, upon collection of bodily lead data and additional geocoded data in ABCD, our future research will involve analyses of both chemical and “environmental-disparity mixtures” to both study developmental trajectories more comprehensively and evaluate the relative strengths of the associations between adolescent development and other sources of disparity (e.g., air pollution, residential segregation)*” (**pp. 27-28, Lines 530-556**). Further, throughout the discussion, we have also edited sentences to not only describe lead-exposure risk, but also its related environmental factors that may also account for our results (**p. 22, Lines 427-428; p. 24, Line 468**).
